# A New Generation of Sumanene‐Based AIEgens for the Effective Recognition of Metal Cations in Solutions Containing 95 vol % of Water

**DOI:** 10.1002/chem.202500705

**Published:** 2025-04-14

**Authors:** Jakub S. Cyniak, Hidehiro Sakurai, Artur Kasprzak

**Affiliations:** ^1^ Faculty of Chemistry Warsaw University of Technology Noakowskiego Str. 3 00-664 Warsaw Poland; ^2^ Division of Applied Chemistry Graduate School of Engineering Osaka University, 2-1 Yamadaoka Suita 565-0871 Osaka Japan; ^3^ Innovative Catalysis Science Division Institute for Open and Transdisciplinary Research Initiatives (ICS-OTRI) Osaka University, Suita Osaka 565-0871 Japan

**Keywords:** buckybowls, receptors, sumanene, aggregation-induced emission, cations detection

## Abstract

In recent years, the application of sumanene derivatives for the optical detection of metal cations was demonstrated. Unfortunately, known sumanene‐based receptors enable the detection process in purely organic solutions or in aqueous media containing not lower than 50 vol % of organic solvent. Designing easy‐to‐synthesize sumanene‐based optical receptors able to effectively recognize metal cations in aqueous solutions containing a slight volume fraction of organic solvent remained an important and vital challenge. In this work, we show that water‐insoluble sumanene receptors composed of only carbon and hydrogen atoms enable the effective detection of cesium (Cs^+^) or lithium (Li^+^) cations in solutions containing 95 vol % of water. Their key feature is related to the exhibition of an aggregation‐induced emission (AIE) effect. We discovered that the designed sumanene receptors exhibit excellent detection parameters expressed by Stern‐Volmer constant values at the level from 10^8^ to 10^10^ M^−1^. This work also shows that by simple modification of the sumanene receptor structure, it is possible to drastically change its detection preference from large Cs^+^ cations to small Li^+^ cations. The highest sensitivity of the designed receptors was concluded for Na^+^ or Li^+^, depending on the receptor structure. This work opens new avenues in designing sumanene‐based optical receptors.

## Graphical abstract

Easy‐to‐synthesize and aggregation‐induced emission (AIE) active sumanene‐based receptors were synthesized and successfully applied to the effective recognition of cesium or lithium cations in solutions containing 95 vol % of water.

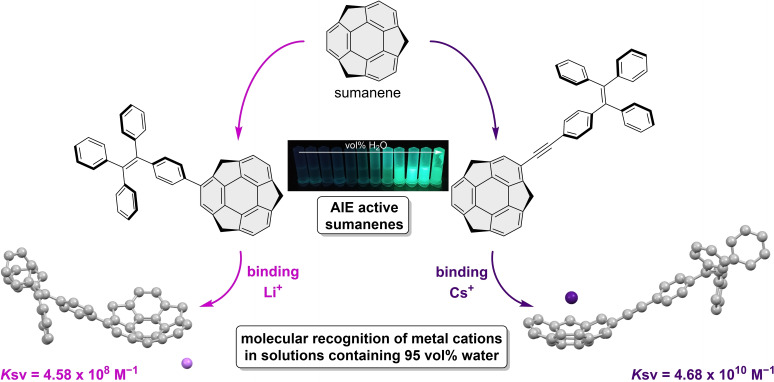

## Introduction

**Figure 1:**
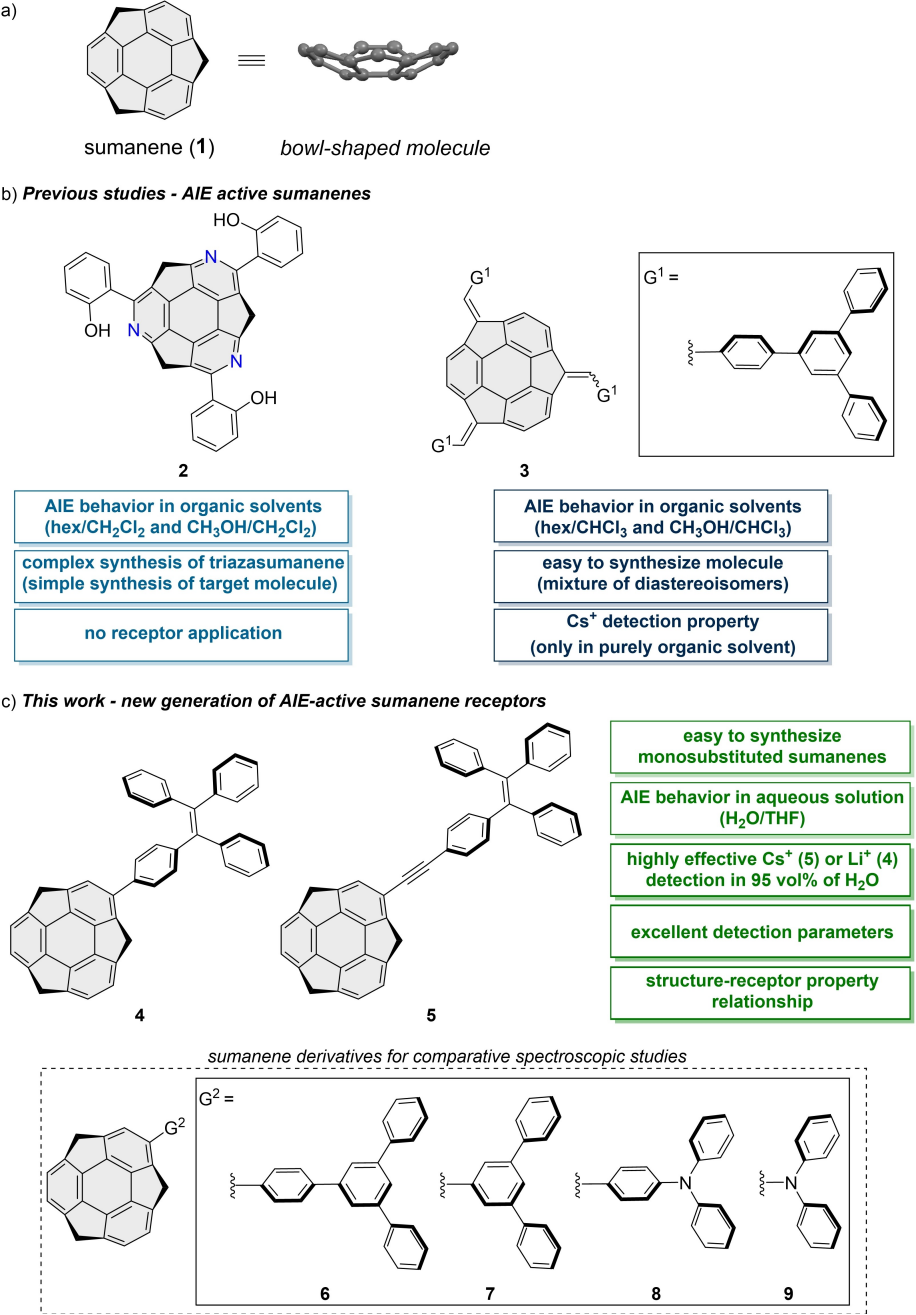
(**a**) Structure of sumanene (**1**), graphical presentation of (**b**) reported sumanene‐based AIEgens together with their key features and (**c**) aims and contents of this work.

Bowl‐shaped polyaromatic hydrocarbons (buckybowls) constitute an important class of molecules, featuring various interesting properties and functions.[^1^, ^2^] Sumanene (**1**, Figure 1a), a curved C_60_ fullerene fragment, is a flagship example belonging to the class of buckybowls. It was first synthesized in 2003, more than 30 years after corannulene (another C_60_ fullerene fragment).[^3^, ^4^] Through the last 21 years, it was discovered that sumanene offers many exciting possibilities to organic, supramolecular, and materials chemists, related to the unique physicochemical properties resulting from curvature in its structure.[^5^, ^6^, ^7^] Sumanene and its derivatives have been considered excellent materials for the creation of various functional organic molecules. Many important reports dealing with the design of, *e. g*., sumanene‐based metal‐organic capsules,^[8]^ frameworks and supramolecular polymers,[^9^, ^10^] and dielectric materials,[^11^, ^12^, ^13^, ^14^] were recently published. Examples of the application of functionalized sumanenes as molecular receptors of metal cations were published by our groups during the last 7 years.[^15^, ^16^, ^17^, ^18^, ^19^, ^20^, ^21^, ^22^, ^23^, ^24^] Sumanene core plays a key role in terms of providing molecular recognition phenomena and the nature of this recognition is cation‐π interaction at sumanene's neutral state.[^25^, ^26^, ^27^] Most of the reports deal with the site‐selective detection of cesium cations (Cs^+^).[^15^, ^16^, ^17^, ^18^, ^19^, ^20^, ^21^, ^22^, ^23^] Additionally, one report demonstrated the unexpected possibility of lithium cation (Li^+^) detection with bis(terpyridine)–ruthenium(II) complexes containing a sumanene motif.^[24]^

Achievement of the detection process with sumanene derivatives in aqueous solutions containing the lowest volume of organic solvent possible is desirable. However, to date, detection of metal cations by sumanene receptors was achieved in purely organic medium or aqueous solutions containing not lower than 50 vol % of organic solvent (tetrahydrofuran (THF) or acetonitrile). The need to use such a relatively high vol % of organic solvent was related to the insolubility of sumanene derivatives in water and their relatively low solubility in aqueous solutions containing an organic solvent, even at the concentrations at the level of 10^−5^ M. Potential introduction of functionalities improving the solubility of sumanene in water, such as hydroxyl or amino groups, might be considered as one of the solutions. However, such modifications could cause an undesirable lowering effectiveness and/or selectivity of the metal cations detection by sumanene, related to the possible interfering effect of introduced functionalities. Additionally, increasing the vol % of water in solution could also lower the sumanene‐cation binding process parameters, predominantly due to the strong hydration of metal cations what is undesirable in terms of this molecular recognition phenomenon.

Molecules featuring aggregation‐induced emission (AIE) behavior (so‐called AIEgens) have been widely studied in recent years, toward their applications in, *e. g*., organic light‐emitting diodes, fluorescent probes, or molecular receptors.[^28^, ^29^, ^30^] The literature points on two examples of known AIE‐active sumanene derivatives. The first one (**2**, Figure 1b), synthesized in 2017 in several steps, features the triazasumanene core.^[31]^ Compound **2** exhibited an excited‐state intramolecular proton transfer process (ESIPT) together with AIE behavior. The second example of sumanene‐based AIEgen (**3**, Figure 1b) was synthesized (2021) in one step from sumanene using a condensation reaction.^[17]^ The obtained mixture of diastereoisomers of **3** was used for spectrofluorimetric detection of Cs^+^; however, it was used in a purely organic solution.

Inspired by the attractive field of AIEgens chemistry, here, we report on the design of a new generation of easy‐to‐synthesize and AIE‐active sumanenes monosubstituted with 1,1,2,2‐tetraphenylethylene units (**4**–**5**, Figure 1c). Despite compounds **4**–**5** being water‐insoluble, as well as being composed of only carbon (C) and hydrogen (H) atoms, we found that it is possible to use **4**–**5** for the effective spectrofluorimetric detection of metal cations in aqueous solutions containing 95 vol % water and only 5 vol % of THF. Additionally, for the first time in the buckybowls science we present that by simply tuning the structure of the AIE‐active sumanene receptor, it is possible to drastically change its detection preference from the recognition of Cs^+^ (receptor **5**) to Li^+^ (receptor **4**). Notably, we show that sumanene receptors **4**–**5** featuring turn‐off fluorescence behaviors are characterized by excellent values of Stern‐Volmer constants (*K*_sv_) at the level from 10^8^ to 10^10^ M^−1^. Our assays also include comparative studies on the photophysical properties of sumanenes monosubstituted with other non‐planar polyaromatic skeletons, including 1,3,5‐triphenylbenzene (**6**), 3,5‐diphenylbenzene (**7**), triphenylamine or diphenylamine (**8**–**9**; see all structures in Figure 1c).

## Results and Discussion

**Scheme 1:**
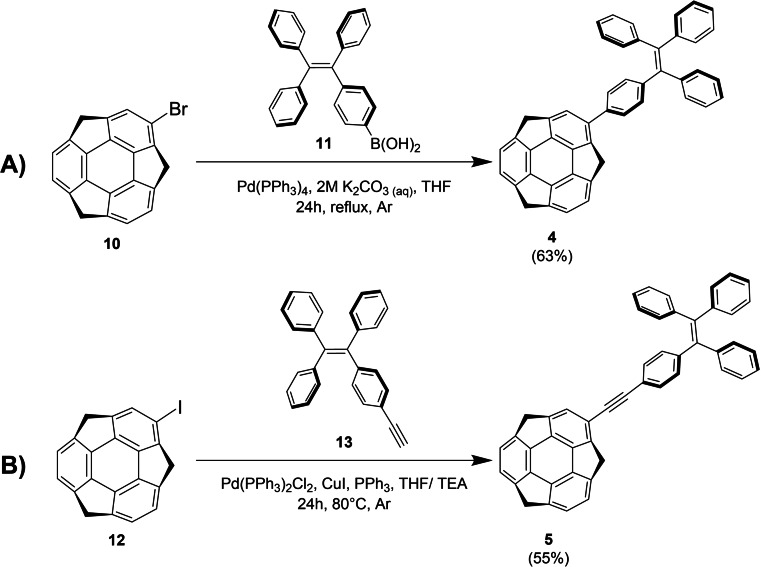
Synthesis of compounds **4**–**5**.

Refer to Supporting Information (SI), Section S1, for the full experimental details on the synthesis of **4**–**9**. In brief, target sumanene derivatives **4** and **5** bearing the 1,1,2,2‐tetraphenylethylene units were synthesized with good yields from 2‐bromosumanene (**10**)[^20^, ^32^] or 2‐iodosumanene (**12**),^[33]^ respectively, in one‐step reactions (Scheme 1). Compound **4** was synthesized (63 %) through Suzuki‐Miyaura cross‐coupling between **10** and 4‐(1,2,2‐triphenylvinyl)phenylboronic acid (**11**) with the usage of tetrakis(triphenylphosphine)palladium(0). Compound **5** was obtained (55 %) through Sonogashira cross‐coupling reaction between **12** and (2‐(4‐ethynylphenyl)ethene‐1,1,2‐triyl)tribenzene (**13**). Reaction was performed in THF/triethylamine (TEA) mixture (1/4 *v*/*v*) in the presence of copper(I) iodide, triphenylphosphine and bis(triphenylphosphine)palladium(II) dichloride. Additionally, compounds **6**–**8** were synthesized by means of Suzuki‐Miyaura cross‐coupling starting from **10** and respective boronic acids or their pinacol esters, whereas compound **9** was obtained employing the Ullman cross‐coupling with **12** and *N*,*N*‐diphenylamine as starting materials (refer to SI, Scheme S1 in Subsection S1.2, for synthesis pathways of **6**–**9**). Density functional theory (DFT) computed structures of compounds **4**–**9** using Gaussian software^[34]^ with B3LYP functional^[35]^ and 6‐311++G(d,p) basis set^[36]^ supported that the substituents introduced into the sumanene skeleton were non‐planar and constituted twisted polycyclic aromatic hydrocarbons (refer to SI, Section S6 for the DFT‐computed structures of **4–9**). Additionally, according to our computations compound **7** featured lower bowl depth (1.122 Å) than **1**, **4**–**6** and **8**–**9** (1.143–1.150 Å; Table S5, SI).

Refer to SI, Sections S1‐S3, for the full characterization data on **4**–**9**. The successful synthesis of **4**–**9** was confirmed with NMR spectroscopy and high‐resolution mass spectrometry (HRMS). In brief, regarding NMR characteristics, compounds **4**–**9** featured characteristic signals coming from the sumanene skeleton, that is H_Ar_ (C_Ar_), H_benzylic,exo_ and H_benzylic,endo_ (C_benzylic_), as well as introduced polyaromatic units in the aromatic region. Ultimate confirmation on the isolation of **4**–**9** was achieved with HRMS since the experimental isotopic patterns for **4**–**9** were well‐matched with the computed ones.

**Figure 2:**
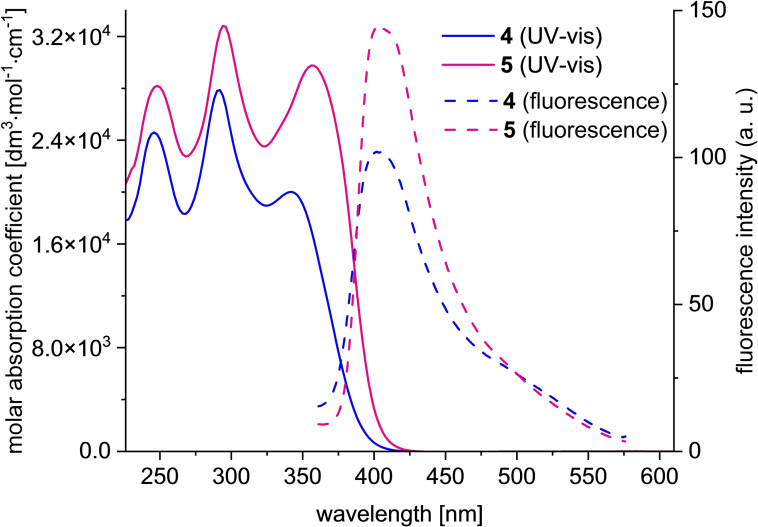
UV‐vis (*C*=2×10^−5^ M, THF) and fluorescence spectra (*C*=2×10^−5^ M, THF, *λ*_ex,**4**_=292 nm, *λ*_ex,**5**_=294 nm) of **4** and **5**.

The fundamental photophysical properties of **4**–**9** were studied with UV‐vis and fluorescence spectroscopies. The spectra of target compounds **4**–**5** are presented in Figure 2, whereas the full spectroscopic data for **4**–**9** are presented in SI, Section S4. The UV‐vis spectra of **4**–**5** featured three major absorption maxima (*λ*_max_) located at 246–248 nm, 292–294 nm, and 341–358 nm (Figure 2). Interestingly, among the compounds **4**–**9**, only **4**–**5** featured the last *λ*_max_ featuring significant molar absorption coefficient (*ϵ*) value at the level of 10^4^ dm^3^×mol^−1^×cm^−1^. The *λ*_max_ of 341–358 nm for **4**–**5** was also significantly (*ca*. 60–80 nm) red‐shifted in comparison to native sumanene (*ca*. 280 nm^[37]^). Time‐dependent‐DFT (TD‐DFT) computations (B3LYP/6‐311++G(d,p)/IEFPCM(THF)^[38]^) demonstrated that compound **5** featured a narrower HOMO–LUMO energy gap (3.45 eV) in comparison to **4** (3.80 eV; Figure S76, SI). This resulted in the red shift in *λ*_max_ for **5** in comparison to **4**, both in the experimental and predicted UV‐vis spectra, *i. e*., 341 nm (predicted 347 nm) and 358 nm (predicted 365 nm) for **4** and **5**, respectively. The narrower HOMO–LUMO energy gap for **5** and observed red‐shift suggested more extended π‐conjugation in comparison to **4**, what could be reasoned with the presence of the ethynyl bridge between sumanene and 1,1,2,2‐tetraphenylethyleneunits in **5**.^[33]^ TD‐DFT computations also supported the observed increase in the *ϵ* value for **5** (2.97×10^4^ dm^3^×mol^−1^×cm^−1^ for *λ*_max_=358 nm) in comparison to **4** (2.00×10^4^ dm^3^×mol^−1^×cm ^−1^ for *λ*_max_=341 nm). Compounds **4**–**9** were found to be blue light emitters since their fluorescence spectra featured emission maxima (*λ*_em_) between 390–430 nm (see Figure 2 for the fluorescence spectra of target compounds **4**–**5**, *λ*_em_ = 402–404 nm; for the spectra of **6**–**9**, refer to Figure S26, SI). Notably, *λ*_em_ for the **4**–**9** were red‐shifted in comparison to the sumanene spectrum (*λ*_em_≈380 nm^[37]^), further revealing π‐conjugation for these derivatives.

**Figure 3:**
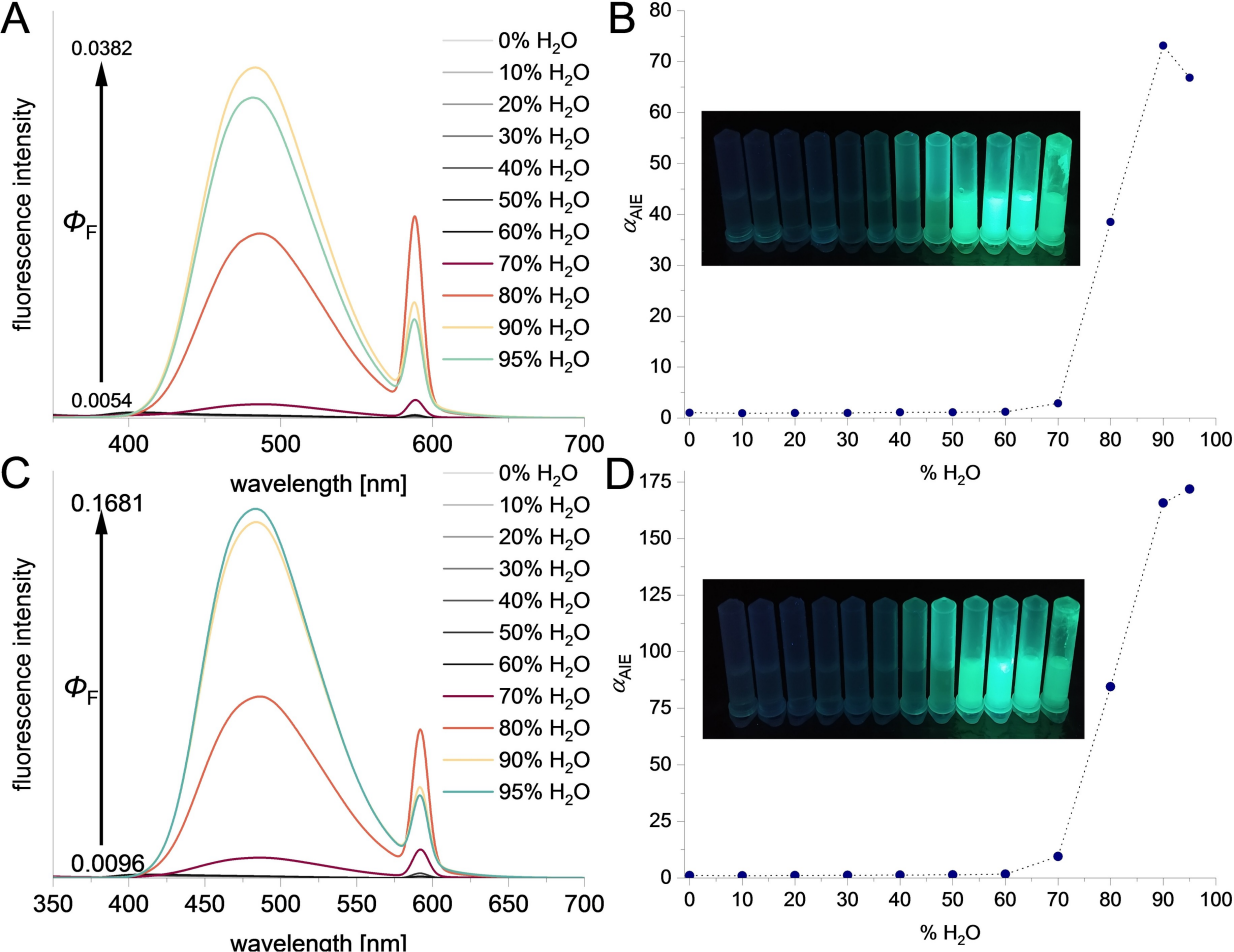
**A**) Fluorescence spectra of **4** (*C*_**4**_=2×10^−5^ M, *λ*_ex_=292 nm) in different H_2_O/THF solvent systems (difference in the *Φ*_F_ is also shown); **B**) Fluorescence intensity dependence on the H_2_O content (vol %) in the sample, samples upon the irradiation with UV‐light (*λ*_ex_=365 nm); **C**) Fluorescence spectra of **5** (*C*_**5**_=2×10^−5^ M, *λ*_ex_=294 nm) in different H_2_O/THF solvent systems (difference in the *Φ*_F_ is also shown); **D**) fluorescence intensity dependence for **5** on the H_2_O content (vol %) in the sample, photograph of samples upon the irradiation with UV‐light (*λ*_ex_=365 nm).

Next, the fluorescence spectra of **4**–**9** were measured in the mixtures of THF (good solvent) and H_2_O (poor solvent) with increasing amounts of vol % of H_2_O in the sample to check potential AIE behavior for these molecules. Sumanene derivatives substituted with a 1,1,2,2‐tetraphenylethyleneskeleton (compounds **4**–**5**) were found to feature exclusive AIE behavior (Figure 3) among tested compounds **4**–**9** (refer to SI, Section S4, for a summary of spectra for **4**–**9**). For **4**–**5**, fluorescence intensity did not change much up to 70 vol % of H_2_O. For higher vol % of H_2_O, a significant increase in fluorescence intensity was observed. This resulted in the improvement (from 7‐ to 16‐fold increase) of the fluorescence quantum yield (*Φ*_F_), from 0.0054‐0.0096 for solutions of **4**–**5** in THF to 0.0382–0.1681 for their aggregates (relative method; refer to SI, Section S1, for the details of estimation of *Φ*_F_). This clear AIE behavior for **4**–**5** could be also observed with the naked eye (Figure 3). The maximal AIE factor (*α*_AIE_)^[39]^ for **4** and **5** equaled 80 and 170, respectively, pointing to the attractive fluorescence enhancement for these molecules. Interestingly, the fluorescence spectra of aggregates of **4**–**5** featured red shift (*ca*. 80 nm, *λ*_em_ = 482–483 nm) in comparison to the spectra measured in THF (*λ*_em_=402–404 nm), what is also in a good agreement with the findings from the solid‐state fluorescence spectra of **4**–**5** (*λ*_em_=471–473 nm; Figures S34–S35, SI). Dynamic light scattering (DLS) studies revealed that the increase of fluorescence intensity for **4**–**5** was accompanied by the decrease of the mean hydrodynamic diameter of particles (Figures S36–S38, SI). Aggregates of **5** in solution containing 70 vol %, 80 vol %, and 95 vol % of H_2_O featured mean hydrodynamic diameter of particles forming the sample of 472.6 nm, 408.9 nm, and 127.7 nm, respectively. Considering that the formation of aggregates was induced by the addition of polar solvent (water) to the non‐polar organic molecules (sumanene derivatives **4** and **5**) in THF, the influence of solvophobic effects during the formation of aggregates might be considered as important factor in terms of resultant size of aggregates. Scanning Electron Microscopy (SEM) studies with aggregated **4** and **5** revealed the formation of spherical particles with diameter of *ca*. 1 nm (Figure S39, SI).

Subsequently, the receptor properties of AIE‐active sumanene derivatives **4**–**5** were tested toward the recognition of s‐block monovalent metal cations, namely lithium (Li^+^), sodium (Na^+^), potassium (K^+^), rubidium (Rb^+^), and cesium (Cs^+^). Considering the results of our AIE studies, spectrofluorimetric titrations were performed in the solvent system containing 95 vol % of water and 5 vol % of THF. Refer to SI, Sections S1 and S5, for the full data on those receptor studies. Note that on the contrary to the previous studies in solution (homogeneous samples) with optical receptors based on monosubstituted sumanenes,^[15,20][24]^ the herein examined detection process relies on the non‐covalent interactions between aggregated sumanene derivatives **4**–**5** and introduced analyte (metal cation), thus, this molecular recognition phenomenon is a quasi‐heterogeneous process.

**Figure 4:**
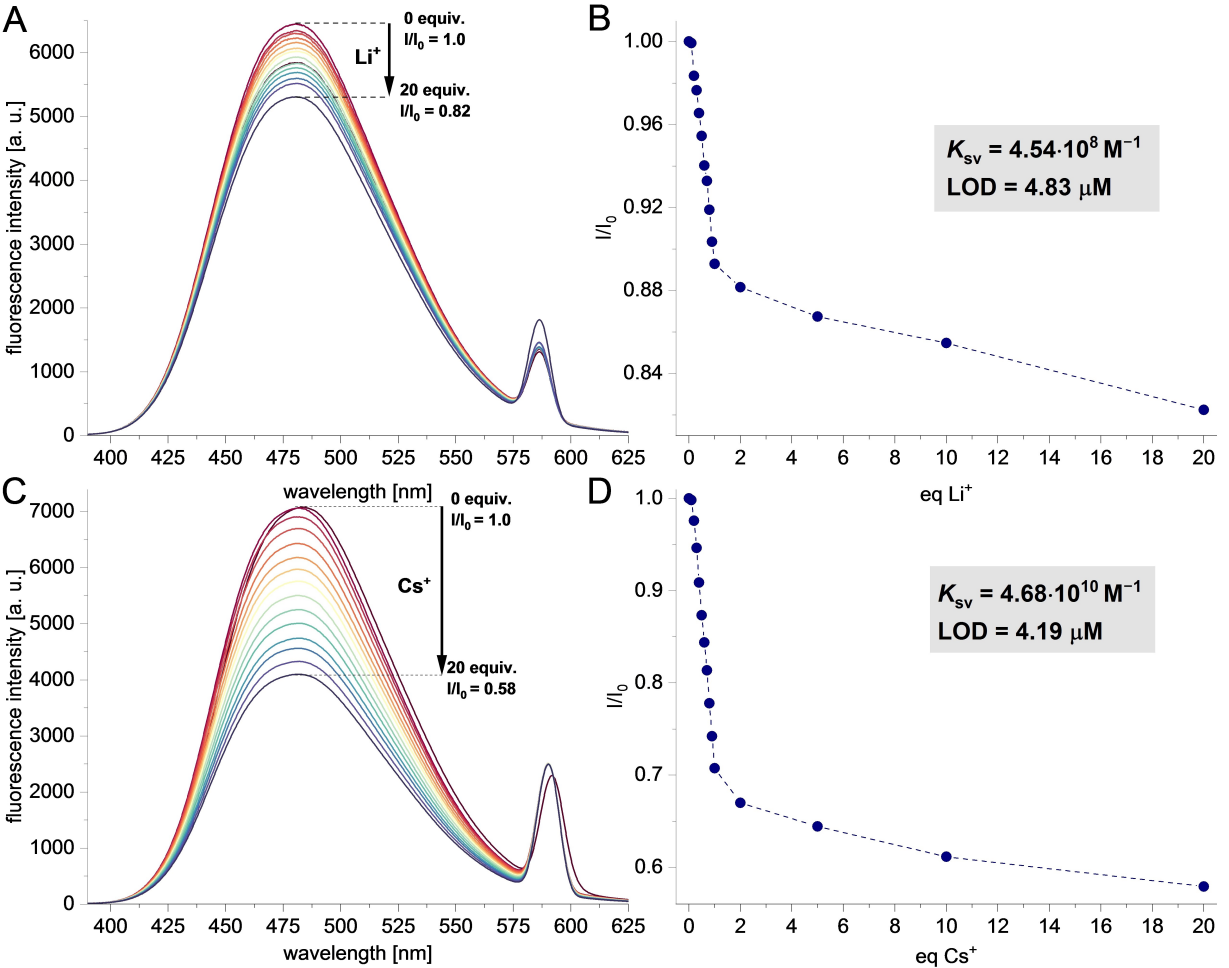
**A**) Fluorescence spectra of aggregated **4** in the presence of various molar equivalents of Li^+^ (H_2_O/THF 95 : 5 *v*/*v*, *C*_**4**_=2×10^−5^ M, *λ*_ex_=292 nm); **B**) Titration curve on the interactions between aggregated **4** and Li ^+^ (*K_sv_* and LOD values are also provided); **C**) Fluorescence spectra of aggregated **5** in the presence of various molar equivalents of Cs^+^ (H_2_O/THF 95 : 5 *v*/*v*, *C*_**5**_=2×10^−5^ M, *λ*_ex_=294 nm); **D**) Titration curve on the interactions between aggregated **4** and Cs^+^ (*K_sv_* and LOD values are also provided); *I*_0_ and *I* denote the emission intensity of the sumanene derivative without the presence of the metal cation and in the presence of the metal cation (*λ*_em_=480 nm), respectively.

**Figure 5:**
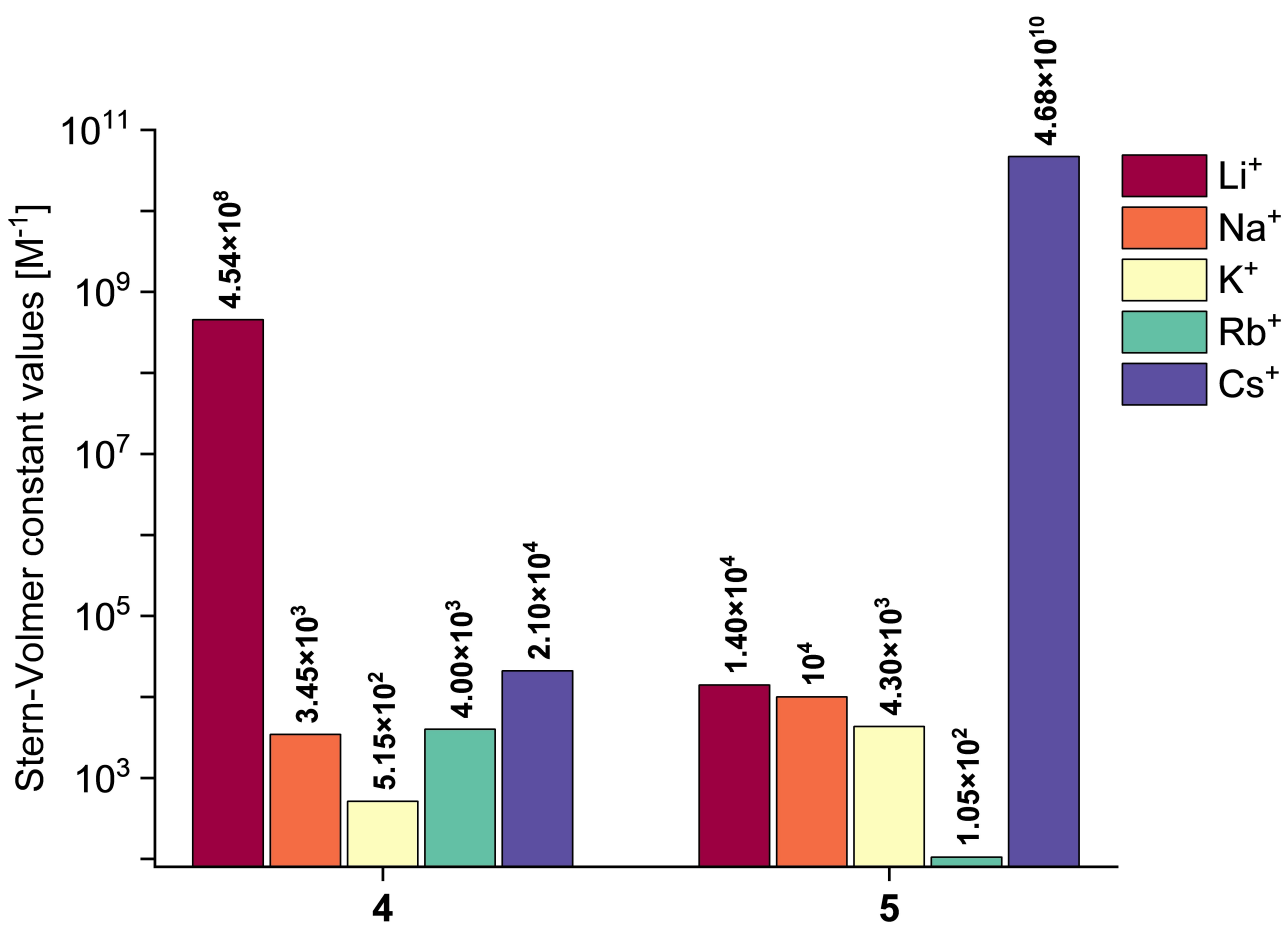
Graphical comparison of the Stern‐Volmer constant values for the interactions between **4**–**5** and tested metal cations.

Receptor **4** and **5** exhibited an effective turn‐off fluorescence behavior upon the presence of increasing amounts (molar equivalents) of Li^+^ and Cs^+^ cations, respectively (Figure 4). This feature was ascribed to a plausible existence of a photo‐induced electron transfer (PET) with the inclusion of 1,1,2,2‐tetraphenylethylene unit in **4** and **5** structure and metal cation.^[40]^ Time‐course emission spectra assays in the presence of 1 molar equiv. of the respective cation revealed that emission intensity dropped constantly upon the addition of the cation, and the plateau of fluorescence intensity was observed after ca. 480 s and 235 s for receptor **4** (Li^+^) and **5** (Cs^+^), respectively (Figures S60‐S61, SI). It means that the recognition process with **4**–**5** was characterized by a satisfactory response time. Stern‐Volmer constant (*K*_SV_) values were very high and equaled 4.54×10^8^ M^−1^ and 4.68×10^10^ M^−1^ for the interactions between **4** and Li^+^ and between **5** and Cs^+^, respectively (Figures S40‐S59, SI). For comparison, *K*_SV_ values for the interactions with other cations were significantly decreased, namely from ten thousand to a million times lower (see Figure 5). It means that the designed receptors feature excellent parameters toward the detection of Li^+^ (receptor **4**) or Cs^+^ (receptor **5**). The limit of detection (LOD) values for **4**–**5** were 4.83–4.18 μM. Data collected from fluorescent titrations were also analyzed using Bindfit program,[^41^, ^42^, ^43^] which suggested that the dynamic formation of 1 : 1 cation‐π complexes for the studied systems is the most likely (see SI, Figures S62–S63 and Tables S3‐S4). The association constant values (estimated with Bindfit) for the **4**‐Li^+^ and **5**‐Cs^+^ interactions were at the level of 10^5^ M^−1^ based on the global fitting to the 1 : 1 model. The 1 : 1 stoichiometry of the formed complexes was further supported by the continuous variation method (Job's plot) analyses[^44^, ^45^] (see SI, Section S5), as well as additional control **4**‐Li^+^ and **5**‐Cs^+^ titrations with changed host (**4** or **5**) concentration (see SI, Tables S5–S6).[^41^, ^42^, ^43^]

Additionally, analysis of the fluorescence quenching efficiency for the studied systems was used for concluding sensitivity of receptors **4**–**5** toward detection of tested alkali metal cations. Fluorescence quenching efficiency (taken for the titration endpoint) was found to be (from highest to lowest) Na^+^>Cs^+^>K^+^>Rb^+^>Li^+^ and Li^+^>Cs^+^>Rb^+^>Na^+^= K^+^ for receptor **4** and **5**, respectively (see SI, Figures S67–S68). It means that the highest sensitivity of detection was observed for **4**‐Na^+^ and **5**‐Li^+^ systems. We hypothesized that for these systems there might have been the most significant changes in the conformation and/or geometry of the receptor during the non‐covalent binding process of alkali metal cation, what caused the most intense changes in the fluorescence of the receptor in these cases.

Notably, while the Cs^+^ recognition property of several classes of sumanene derivatives has been reported,[^15^, ^16^, ^17^, ^18^, ^19^, ^20^, ^21^, ^22^, ^23^] there is only one example of the sumanene‐based optical probe with an unexpected Li^+^ detection feature^[24]^ (Table 1). Known sumanene‐based receptors able to bind Cs^+^ also differ significantly in terms of their chemical structure. The reported sumanene‐tethered Li^+^ receptor featured a different structural motif with ruthenium(II) complexed by the pincer ligand attached to a sumanene skeleton. On the other hand, the structural difference between herein reported receptors **4**–**5** featuring different metal cation recognition preference was very slight, namely the presence (**5**) or absence (**4**) of the ethynyl bridge between sumanene and 1,1,2,2‐tetraphenylethylene skeletons. It means that for the first time in buckybowls science by simple change in the receptor structure it was possible to tune its detection preference. The observed Li^+^ binding preference for compound **4** was further corroborated by control competition experiments. These experiments involved the initial addition of an excess of either Li^+^ or Cs^+^ to a solution of compound **4**, followed by titration with the alternate cation (Cs^+^ or Li^+^). According to these experiments, titration curve for **4**‐Cs^+^ (initial addition of Li^+^) was significantly affected by the initial presence of Li^+^ in solution, as evidenced by a markedly different titration endpoint compared to the standard **4**‐Cs^+^ titration curve (see SI, Figure S66). In contrast, both titration curves for **4**‐Li^+^ were similar, whether in the absence or presence of an initial portion of Cs^+^, with nearly identical titration endpoints (see SI, Figure S65). These results indicate that the interactions between compound **4** and Li^+^ are stronger than those with Cs^+^, providing further evidence for the preferential binding of Li^+^ by receptor **4**. Next, as a general comparison, known sumanene receptors featured lower *K*_sv_ or apparent association constant (*K*_app_) values in comparison to herein reported sumanene receptors **4**–**5** (see comparison in Table 1). Thus, our studies on the receptor properties of **4**–**5** revealed that is it possible to detect selected metal cations with sumanene‐based receptors in solutions containing even 95 vol % of water with excellent interaction parameters (see comparison data in Table 1).

**Table 1:** Comparison of *K*_sv_ and *K*_app_ values (estimated with fluorescence spectroscopy) for sumanene‐based Cs^+^ and Li^+^ optical receptors, as well as solvents used for the analyses.

	*sumanene‐based Cs* ^*+*^ *receptors*				
Entry	Receptor	*K*_SV_	*K*_app_	Solvent	Ref.
1	**5**	4.68×10^10^	N/D	H_2_O : THF=95 : 5 v/v	This work
2	sumanenes monosubstituted with ferrocene (various linkers)	from 1.70×10^4^ to 4.50×10^5^	from 5.90×10^5^ to 8.70×10^5^	CHCl_3_ : CH_3_OH=1 : 1 v/v	[15,20]
3	sumanenes trisubstituted with ferrocene (various linkers)	N/D	from 2.88×10^4^ to 7.35×10^6^	CHCl_3_ : CH_3_OH=1 : 1 v/v or H_2_O : THF=1 : 1 v/v	[18,19,21,23]
4	sumanene tetrasubstituted with ferrocene	N/D	3.90×10^3^	H_2_O : THF=1 : 1 v/v	[22]
5	tetrasubstituted, push‐pull sumanene chromophore	N/D	1.20×10^5^	H_2_O : THF=1 : 1 v/v	[21]

	*sumanene‐based Li* ^*+*^ *receptors*				
Entry	Receptor	K_SV_	K_app_	Solvent	Ref.
6	**4**	4.54×10^8^	N/D	H_2_O : THF=95 : 5 v/v	This work
7	sumanene‐based bis(terpyridine)‐ ruthenium(II) complexes	from 0.73 to×10^3^ to 5.05×10^3^	N/D	H_2_O : CH_3_CN=1 : v/v	[24]

**Figure 6:**
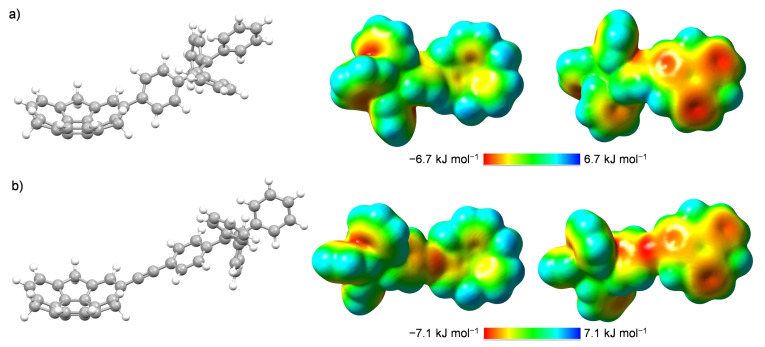
DFT‐optimized (B3LYP/6‐311++G(d,p)) structures of **4** (**a**) and **5** (**b**) together with ESP plots (views from two different perspectives).

Finally, the interesting structure‐receptor property relationship with **4**–**5** was further investigated with DFT computations (refer to SI, Section S6 for DFT computations details and full data). Sumanene skeleton plays a key role in terms of studied metal cations recognition phenomena by cation‐π interactions.[^25^, ^26^, ^27^] From the DFT optimized (B3LYP/6‐311++G(d,p)) structures of **4**–**5** it can be seen that the installation of ethynyl bridge between sumanene skeleton and 1,1,2,2‐tetraphenylethylene unit (compound **5**) provided the relatively long distance between these structural motifs (Figure 6). As a result, it could be hypothesized that there was a possibility for cation‐π interactions between the sumanene bowl in **5** and large Cs^+^ cations, similar to recognition features of known Cs^+^‐oriented sumanene receptors.[^15^, ^16^, ^17^, ^18^, ^19^, ^20^, ^21^, ^22^, ^23^] On the other hand, in compound **4** a sterically bulky 1,1,2,2‐tetraphenylethylene unit was located in the direct proximity of the sumanene skeleton. We believe this structural difference inhibited effective cation‐π interactions between **4** and large Cs^+^ cations, whereas boosted the molecular recognition process with small Li^+^ cations, what might stand for the observed changes in Li^+^/Cs^+^ cations binding preference between **4** and **5**.

**Figure 7:**
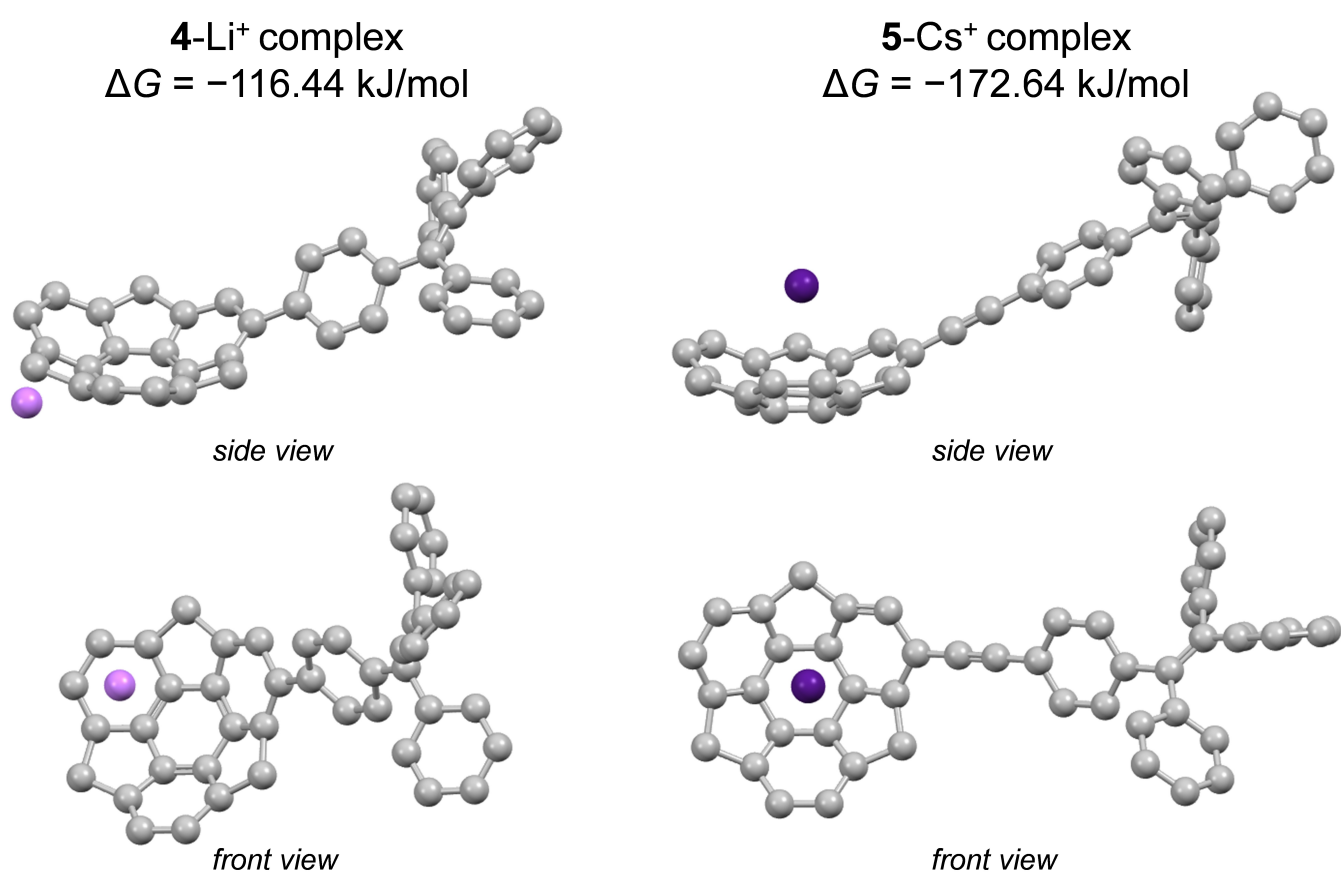
DFT‐optimized structures of **4**‐Li^+^ and **5**‐Cs^+^ complexes (most energetically favored) together with the interaction energy values (views from two different perspectives, hydrogen atoms were omitted for clarity of the image).

Apart from the steric effects, the electrostatic contribution could be also considered another important factor in terms of cation–π interactions. DFT computations on electrostatic surface potential (ESP) indicated a non‐uniform distribution of charge within the **4**–**5** (Figure 6). In both cases, more negative potential was found at the convex site of the sumanene bowl, suggesting that this site might play an important role in Cs^+^/Li^+^ binding with **4**–**5**. Further DFT studies were performed to estimate interaction energies (Δ*G*) for the **4**‐Li^+^ and **5**‐Cs^+^ systems (water was included as a solvent in those DFT computations). The 1 : 1 stoichiometry for each complex was subjected to calculation, not only considering the outcomes of spectrofluorimetric studies but also due to computationally expensive character of such DFT analyses with metallic species. Three different complex arrangements were considered, namely convex‐ or concave‐oriented ones with the inclusion of sumanene's central or peripheral six‐membered ring. Figure 7 presents the DFT‐computed structures of the most energetically favorable **4**‐Li^+^ (ωB97X‐D^[46]^/6‐31G^[47]^/IEFPCM(H_2_O)) and **5**‐Cs^+^ (ωB97X‐D/LANL2DZ^[48]^/IEFPCM(H_2_O)) complexes. Importantly, interaction energies for each convex‐ or concave‐oriented complex featured negative values (from −172.64 kJ mol^−1^ to −110.38 kJ mol^−1^; Figures S77–S78, SI). Therefore, it can be concluded that the formations of these systems were energetically favorable. Interestingly, Δ*G* values for convex‐ or concave‐oriented **4**‐Li^+^ systems featured similar values (from −116.44 kJ×mol^−1^ to −110.38 kJ×mol^−1^). On the other hand, concave‐oriented **5**‐Cs^+^ complex (Δ*G*=−172.64 kJ×mol^−1^) featured *ca*. 45 kJ×mol^−1^ lower Δ*G* value in comparison to convex‐oriented ones, suggesting this arrangement as the most thermodynamically prefered one. This conclusion could be considered in good accordance with the results of DFT studies on known sumanene‐based Cs^+^ complexes.[^18^, ^23^, ^25^] Additionally, according to our computations, Δ*G* values for **4**‐Li^+^ and **5**‐Cs^+^ complexes were lower in comparison to the values for **5**‐Li^+^ and **4**‐Cs^+^ systems, respectively, what further suggested that the formation of **4**‐Li^+^ and **5**‐Cs^+^ complexes was the most energetically favorable (see comparison data in Figures S79–S80, SI).

## Conclusions

In conclusion, this work introduced a new generation of sumanene receptors featuring AIE behavior, which may serve as effective metal cations optical probes. Despite the synthesized sumanene‐1,1,2,2‐tetraphenylethylne conjugates being water‐insoluble and composed of only carbon and hydrogen atoms, the detection process with these receptors could be tracked spectrofluorimetrically in aqueous solutions containing up to 95 vol % water. Additionally, for the first time in buckybowls science, by simple modification of the sumanene receptor structure (installation of an ethynyl bridge), it was possible to tune its properties from effective recognition of large Cs^+^ cations (*K*_SV_=4.68×10^10^ M^−1^; LOD=4.19 μM) to small Li^+^ cations (*K*_SV_=4.54×10^8^ M^−1^; LOD=4.83 μM). Judging from the fluorescence quenching efficiency parameter, the highest sensitivity toward Na^+^ or Li^+^ was concluded for the sumanene receptor featuring the absence or presence of an ethynyl bridge, respectively. We believe these achievements open new horizons in designing optical receptors based on bowl‐shaped molecules and provide further highlights on the enormous potential of carefully tuned sumanene derivatives as molecular receptors of different cationic species.

## Supporting Information

The authors have cited additional references within the Supporting Information (SI).[^49^, ^50^, ^51^, ^52^, ^53^, ^54^] SI contains the following information: Materials and methods, experimental procedures, compounds characterization data, data on: AIE studies, receptor studies and DFT computational details.

## Author Contributions

A.K. conceived the project, analyzed all the data, performed DFT computational studies, provided funding for research from the Polish side, conceptualized the manuscript and wrote its first draft, prepared most parts of SI, and corresponded with the Editor and Reviewers. J.S.C. performed all synthesis, characterization and receptor experiments under the supervision of A.K., analyzed the data, prepared selected images for the manuscript, wrote selected parts of the first draft of the manuscript, and prepared parts of SI. H.S. provided sumanene, funding for research from the Japanese side, and commented on the manuscript during the preparation of the final version.

## Conflict of Interests

There are no conflicts to declare. All authors have approved the final version of the manuscript.

1

## Supporting information

As a service to our authors and readers, this journal provides supporting information supplied by the authors. Such materials are peer reviewed and may be re‐organized for online delivery, but are not copy‐edited or typeset. Technical support issues arising from supporting information (other than missing files) should be addressed to the authors.

Supporting Information

## Data Availability

The data supporting this article have been included as part of the supplementary information.
