# Supplementary material for: A New Generation of Sumanene‐Based AIEgens for the Effective Recognition of Metal Cations in Solutions Containing 95 vol % of Water
Source: Chemistry. 2025 Apr 14;31(26):e202500705. doi: 10.1002/chem.202500705 (PMC12063049; doi:10.1002/chem.202500705)
Supplement: Supplementary file 1 — Supporting Information [file CHEM-31-e202500705-s001.pdf]

Chemistry–A European Journal

Supporting Information

A New Generation of Sumanene-Based AIEgens for the Effective Recognition of Metal Cations in Solutions Containing 95 vol% of Water

Jakub S. Cyniak, Hidehiro Sakurai, and Artur Kasprzak*

SUPPORTING INFORMATION (SI) FOR

A New Generation of Sumanene-Based AIEgens for the Effective Recognition of Metal Cations in Solutions Containing 95 vol% of Water

Jakub S. Cyniak,[a] Hidehiro Sakurai,[b,c] and Artur Kasprzak[a]*

[a] Faculty of Chemistry, Warsaw University of Technology, Noakowskiego Str. 3, 00-664 Warsaw, Poland

* Corresponding author e-mail: artur.kasprzak@pw.edu.pl (A.K.)

[b] Division of Applied Chemistry, Graduate School of Engineering, Osaka University, 2-1 Yamadaoka, Suita, 565-0871 Osaka, Japan

[c] Innovative Catalysis Science Division, Institute for Open and Transdisciplinary Research Initiatives (ICS-OTRI), Osaka University, Suita, Osaka 565-0871, Japan

Table of contents

S1. Experimental section 2

S1.1. Materials and methods 2

S1.2. Synthesis 2

S1.3. Aggregation-induced emission (AIE) studies – preparation of the samples 8

S1.4. Estimation of fluorescence quantum yield for 4-5 and their aggregates 8

S1.5. Receptor studies – titration experiments methodology 8

S2. NMR spectra 10

S3. HRMS spectra 22

S4. Photophysical and AIE studies 25

S5. Receptor studies 33

S6. DFT computations 50

S7. Supporting references 107

S1. Experimental section

S1.1. Materials and methods

Materials. Chemical reagents and solvents were of the higher possible purity and were commercially purchased and purified according to the standard methods, if necessary. Sumanene (**1**)[1], 2-bromosumanene (**10**)[2], 2-jodosumanene (**12**)[3], 4,4,5,5-tetramethyl-2-(5'-phenyl-[1,1':3',1''-terphenyl]-4-yl)-1,3,2-dioxaborolane (**14**)[4] and 2-([1,1':3',1''-terphenyl]-5'-yl)-4,4,5,5-tetramethyl-1,3,2-dioxaborolane (**15**)[5] were synthesized following the literature procedures. Thin layer chromatography (TLC) and preparative thin layer chromatography (PTLC; 2 mm) on SiO_2 were performed using Merck Silica gel 60 F254 plates. Thin layer chromatography (TLC) and column chromatography on Al_2O_3 were performed using aluminum oxide 90 neutral gel (CarlRoth).

The NMR experiments were carried out using a JEOL 600 MHz spectrometer (^{1}H NMR and {^{1}H}^{13}C NMR) equipped with a multinuclear z-gradient inverse probe head. The spectra were recorded at 297.15 K and standard 5 mm NMR tubes were used. ^{1}H NMR (δ_H) and {^{1}H}^{13}C NMR (δ_C) chemical shifts were reported in parts per million (ppm) relative to the solvent signal, *i.e.*, $CDCl_3$, δ_H (residual $CHCl_3$) 7.26 ppm, δ_C (residual $CHCl_3$) 77.16 ppm, DMSO-*d*$_6$, δ_H (residual DMSO) 2.50 ppm, δ_C (residual DMSO) 39.52 ppm, acetone-*d*$_6$, δ_H (residual acetone) 2.05 ppm, δ_C (residual acetone) 206.26 ppm. NMR spectra were analyzed with the MestReNova v12.0 software (Mestrelab Research S.L).

ESI-HRMS (TOF) measurements were performed with a Q-Exactive ThermoScientific spectrometer.

UV-vis spectra were recorded with a WVR UV-1600PC spectrometer, with the spectral resolution of 2 cm^{-1}. For the UV-Vis measurements, the wavelengths for the absorption maxima λ_{max} were reported in nm.

Fluorescence spectra were recorded with a HITACHI F-7100 FL spectrometer. Parameters for the liquid spectra acquisition: scan speed: 1200 nm/min, delay: 0.0 s, EX slit: 5.0 nm, EM slit: 5.0 nm. The wavelengths for the emission maxima (λ_{em}) were reported in nm. Parameters for the solid-state spectra acquisition: scan speed: 240 nm/min, delay: 0.0 s, EX slit: 5.0 nm, EM slit: 5.0 nm, PMT voltage: 400 V.

Dynamic light scattering (DLS) measurements were performed with Brookhaven Instruments Particle Size Analyser 90Plus.

Scanning Electron Microscopy (SEM) assays were performed using field emission scanning electron microscope Helios 5 PFIB (Thermo Scientific) with the use of SE (secondary electron) detector.

S1.2. Synthesis

The general synthesis scheme for compounds **4-9** is presented in **Scheme S1**. Experimental procedures are listed below.

S1.2.1. Synthesis of compounds 4, 6-8.

A solution of 2-bromosumanene (**10**; 10.0 mg, 0.029 mmol, 1 equiv.), tetrakis(triphenylphosphine)palladium(0) (10.0 mg; 0.0087 mmol, 0.3 equiv.) and boronic acid (**1** or **16** - synthesis of compounds **4** and **8** respectively; 2 equiv.) or pinacol ester (**14** or **15** - synthesis of compounds **6** and **7** respectively; 2 equiv.) in tetrahydrofuran (THF; 6 mL) was stirred under argon atmosphere. A water solution of potassium carbonate (2M) was added (0.5 mL) and the reaction mixture was refluxed under argon atmosphere for 24 hours. Distilled water (10 mL) was added, and the crude product was extracted with CH_2Cl_2 (3x20 mL). Organic layers were combined, washed with 1M HCl (3x20 mL) and water. After drying with $MgSO_4$ followed by filtration, volatiles were distilled off on a rotary evaporator. Finally, the product was purified using column chromatography (SiO_2 or Al_2O_3) and/or PTLC (SiO_2) (for purification process details see below).

Compound 4.

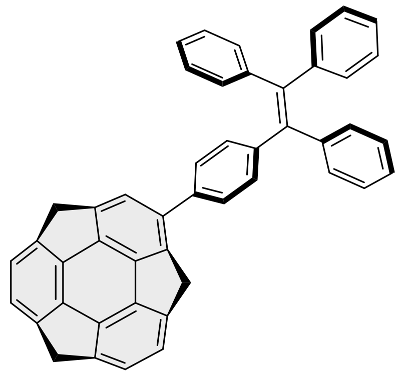

4

^{1}H NMR ($CDCl_3$, 600 MHz, ppm), δ_H 7.32-7.31 (m, 2H), 7.15-7.04 (m, 22 H), 4.83 (d, $^2J_{H\text{-}H}$ = 19.6 Hz, 1H), 4.77–4.65 (m, 2H), 3.47 (d, $^2J_{H\text{-}H}$ = 19.2 Hz, 1H), 3.41 (d, $^2J_{H\text{-}H}$ = 19.3 Hz, 1H), 3.28 (d, $^2J_{H\text{-}H}$ = 19.6 Hz, 1H). {^{1}H}^{13}C NMR ($CDCl_3$, 151 MHz, ppm), δ_C δ 150.1, 149.2x2, 149.1x2, 149.0x2, 148.8, 148.7, 148.6, 148.2, 146.3, 143.9x2, 143.7, 142.8, 141.4, 140.8x2, 138.1, 131.7, 131.6x2, 131.5, 127.9, 127.8x2, 126.7, 126.6x2, 123.5, 123.4x2, 123.0, 122.5, 42.8, 41.9x2. ESI-HRMS (TOF) m/z $[M+H]^+$ calcd. for $C_{47}H_{30}$ 594.23420 found 594.2340.

Purification process: First column chromatography (SiO_2, 1% $CHCl_3$/cyclohexane) to remove 2-bromosumanene (R_f = 0.58), after removal of 2-bromosumanene change of eluent to (15% $CHCl_3$/cyclohexane) for removal of **4** (R_f = 0.4). The collected fractions containing **4** were combined and volatiles were distilled off on a rotary evaporator. Then PTLC (SiO_2, 7% toluene/cyclohexane) (R_f = 0.25) was performed to obtain **4** as light yellow solid (10.9 mg; 63%).

Compound 6.

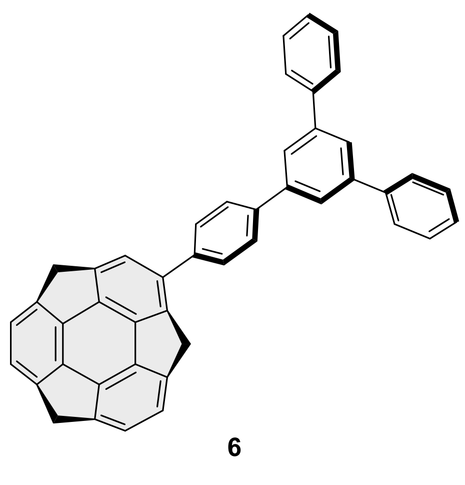

6

^{1}H NMR (DMSO-d_6, 600 MHz, ppm), δ_H 8.08-7.85 (m, 9H), 7.72 (s, 1H), 7.52-7.50 (m, 5 H), 7.43-7.42 (m, 3H), 7.23-7.13 (m, 4H), 5.14 (d, $^2J_{H\text{-}H}$ = 19.8 Hz, 1H), 4.80 (d, $^2J_{H\text{-}H}$ = 19.6 Hz, 1H), 4.71 (d, $^2J_{H\text{-}H}$ = 19.7 Hz, 1H), 3.67 (d, $^2J_{H\text{-}H}$ = 19.7 Hz, 1H), 3.51 (d, $^2J_{H\text{-}H}$ = 19.8 Hz, 1H), 3.44 (d, $^2J_{H\text{-}H}$ = 19.6 Hz, 1H). {^{1}H}^{13}C NMR (DMSO-d_6, 151 MHz, ppm), δ_C 150.3, 149.2, 149.1, 148.9, 148.7, 148.4x2, 148.2, 148.0, 147.8x2, 146.6, 141.7, 141.6, 141.2, 140.2, 140.1, 137.5, 129.0, 128.9, 127.7, 127.4, 127.2, 125.9, 124.9, 124.7, 124.5, 124.0, 123.8, 123.7, 123.3, 42.4, 41.5, 41.3.

ESI-HRMS (TOF) m/z [M+H]+ calcd. for $C_{45}H_{28}$ 568.2185 found 568.2182.
Purification process: First column chromatography (SiO_2, 1% $CHCl_3$/cyclohexane) to remove 2-bromosumanene (R_f = 0.58), after removal of 2-bromosumanene change of eluent to (15% $CHCl_3$/cyclohexane) for removal of **6** (R_f = 0.29). The collected fractions containing **6** were combined and volatiles were distilled off on a rotary evaporator. Then PTLC (SiO_2, 7% toluene/cyclohexane) (R_f = 0.3) was performed to obtain **6** as light yellow solid (8.0 mg; 47%).

Compound 7.

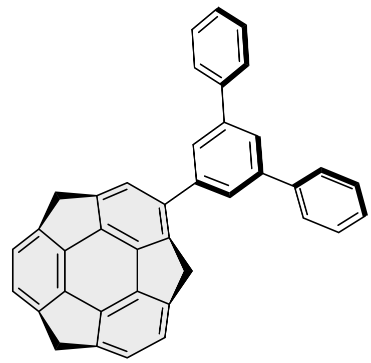

7

^{1}H NMR (acetone-d_6, 600 MHz, ppm), δ_H 7.93-7.89 (m, 3H), 7.86-7.84 (m, 4H), 7.69 (s, 1H), 7.53-7.50 (m, 4H), 7.43-7.40 (m, 2H), 7.22-7.15 (m, 4H), 5.13 (d, $^{2}J_{H-H}$ = 20.3 Hz, 1H), 4.81 (d, $^{2}J_{H-H}$ = 19.5 Hz, 1H), 4.73 (d, $^{2}J_{H-H}$ = 19.9 Hz, 1H), 3.66 (d, $^{2}J_{H-H}$ = 19.5 Hz, 1H), 3.51 (d, $^{2}J_{H-H}$ = 20.3 Hz, 1H), 3.47 (d, $^{2}J_{H-H}$ = 19.9 Hz, 1H). {^{1}H}^{13}C NMR (acetone-d_6, 151 MHz, ppm), δ_C 151.5, 150.4x2, 150.2, 150.0, 149.1, 149.7, 149.5, 149.4, 149.2, 147.7, 143.2, 142.9, 141.9, 139.2, 130.0, 128.6, 128.3, 127.2, 125.7, 124.7, 124.6x2, 124.2x3, 43.6, 42.4, 42.4, 42.3. ESI-HRMS (TOF) m/z [M+H]+ calcd. for $C_{45}H_{28}$ 492.1873 found 492.1870.
Purification process: First column chromatography (SiO_2, 1% $CHCl_3$/cyclohexane) to remove 2-bromosumanene (R_f = 0.58), after removal of 2-bromosumanene change of eluent to (15% $CHCl_3$/cyclohexane) for removal of **7** (R_f = 0.48). The collected fractions containing **7** were combined and volatiles were distilled off on a rotary evaporator. Then PTLC (SiO_2, 100% cyclohexane) (R_f = 0.3) was performed. The collected fractions containing **7** were combined and volatiles were distilled off on a rotary evaporator. Then column chromatography was performed (Al_2O_3, cyclohexane) (R_f = 0.31) to obtain **7** as light yellow solid (6.7 mg; 47%).

Compound 8.

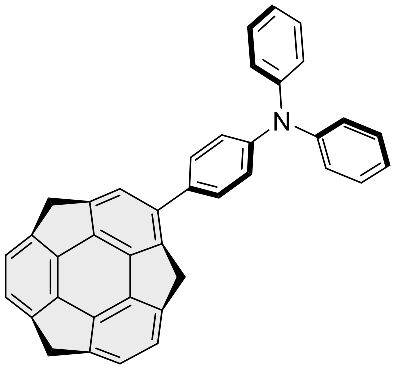

8

^{1}H NMR (acetone-d_6, 600 MHz, ppm), δ_H 7.62-7.61 (m, 2H), 7.47 (s, 1H), 7.35-7.32 (m, 4H), 7.18-7.15 (m, 4H), 7.13-7.07 (m, 8H), 5.00 (d, $^{2}J_{H-H}$ = 19.7 Hz, 1H), 4.77 (d, $^{2}J_{H-H}$ = 19.5 Hz, 1H), 4.71 (d, $^{2}J_{H-H}$ = 19.6 Hz, 1H), 3.58 (d, $^{2}J_{H-H}$ = 19.5 Hz, 1H), 3.50 (d, $^{2}J_{H-H}$ = 19.6 Hz, 1H), 3.39 (d, $^{2}J_{H-H}$ = 19.7 Hz, 1H). {^{1}H}^{13}C NMR (acetone-d_6, 151 MHz, ppm), δ_C 151.2, 150.2, 150.1, 150.0, 149.9, 149.7, 149.6x2, 149.3, 149.1, 148.6, 148.5, 148.1, 146.6, 138.8, 135.3, 130.4, 130.2x2, 125.4, 124.6, 124.4, 124.3, 124.2, 124.0, 123.0, 43.3, 42.2, 42.1. ESI-HRMS (TOF) m/z [M+H]+ calcd. for $C_{39}H_{25}N$ 507.1982 found 507.1979.
Purification process: First column chromatography (SiO_2, 1% $CHCl_3$/cyclohexane) to remove 2-bromosumanene (R_f = 0.58), after removal of 2-bromosumanene change of eluent to (15% $CHCl_3$/ 85% cyclohexane) for removal of **8** (R_f = 0.13). The collected fractions containing **8** were combined and volatiles were distilled off on a rotary evaporator. Then PTLC (SiO_2, 5%EtOAc/ cyclohexane) (R_f = 0.8) was performed. The collected fractions containing **8** were combined

and volatiles were distilled off on a rotary evaporator. Then column chromatography was performed (Al_2O_3, cyclohexane) (R_f = 0.12) to obtain **8** as light yellow solid (5.4 mg; 37%).

S1.2.2. Synthesis of compound 5.

A solution of 2-iodosumanene (**12**; 16.0 mg, 0.041 mmol, 1 equiv.), bis(triphenylphosphine)palladium(II) dichloride (8.6 mg; 0.012 mmol, 0.3 equiv.), (2-(4-ethynylphenyl)ethene-1,1,2-triyl)tribenzene (**13**; 22.1 mg, 0.062 mmol, 1.5 equiv.), copper(I) iodide (2.3 mg, 0.012 mmol, 0.3 equiv.) and triphenylphosphine (6.5 mg, 0.025 mmol, 0,6 equiv.) in triethylamine (TEA; 4 mL) and tetrahydrofuran (THF; 1 mL) was stirred under argon atmosphere at 80° for 24 hours. Then, a 2M hydrochloric acid (10 mL) was added, and the crude product was extracted with CH_2Cl_2 (3x20 mL). Organic layers were combined, washed with saturated solution of sodium bicarbonate and water. After drying with $MgSO_4$ followed by filtration, volatiles were distilled off on a rotary evaporator. Finally, the product was purified using column chromatography (SiO_2; 15% $CHCl_3$/cyclohexane) (R_f = 0.34) to obtain **5** as yellow solid (13.8 mg; 55%).

Compound 5.

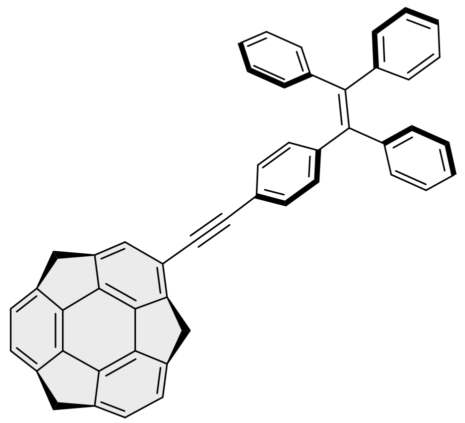

5

^{1}H NMR (DMSO-d_6, 600 MHz, ppm), δ_H 7.32-7.31 (m, 2H), 7.27 (s, 1H), 7.21-7.12 (m, 13 H), 7.01-6.97 (m, 8H), 4.77 (d, $^2J_{H\text{-}H}$ = 20.2 Hz, 1H), 4.71 (d, $^2J_{H\text{-}H}$ = 19.7 Hz, 2H), 3.63–3.51 (m, 3H). {^{1}H}^{13}C NMR (DMSO-d_6, 151 MHz, ppm), δ_C 150.9, 149.5, 149.1, 149.0x2, 148.5, 148.3, 148.2, 147.9, 147.5, 147.3, 143.7, 142.9, 142.7, 141.4, 139.8, 131.1, 130.9, 130.7x2, 130.6, 128.0, 127.9, 127.8, 126.8x2, 126.67, 124.4, 123.9x2, 120.4, 117.2, 90.9, 89.2, 41.4, 41.3, 41.2. ESI-HRMS (TOF) m/z [M+H]+ calcd. for $C_{49}H_{30}$ 618.2342 found 618.2340.

S1.2.3. Synthesis of compound **9**.

A solution of 2-iodosumanene (**12**; 6.9 mg, 0.018 mmol, 1.0 equiv.), *N*-phenylaniline (8.9 mg, 0.053 mmol, 3.0 equiv.), copper(I) iodide (2.3 mg, 0.012 mmol, 0.3 equiv.), potassium carbonate (4.9 mg, 0.035 mmol, 2.0 equiv.) and 18-crown-6 (1.0 mg, 0.0035 mmol, 0.2 equiv.) in 1,2-dichlorobenzene (5 mL) was refluxed under argon atmosphere for 24 hours. Then, distilled water (10 mL) was added, and the crude product was extracted with CH_2Cl_2 (3x20 mL). Organic layers were combined, washed with brine and water. After drying with $MgSO_4$ followed by filtration, volatiles were distilled off on a rotary evaporator. Finally, the product was purified first using column chromatography (SiO_2, 10% $CHCl_3$/cyclohexane) The collected fractions containing **9** were combined and volatiles were distilled off on a rotary evaporator. Then PTLC (SiO_2, 5%EtOAc/cyclohexane) (R_f = 0.55) was performed to obtain **9** as yellow solid (10.0 mg; 50%).

Compound 9.

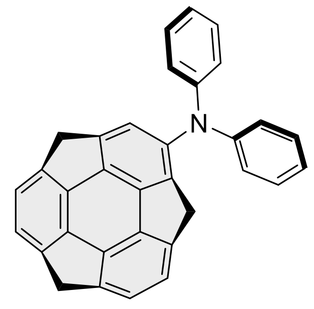

9

^{1}H NMR (acetone-d_6, 600 MHz, ppm), δ_H 7.29-7.27 (m, 4H), 7.19-7.16 (m, 1H), 7.13 (s, 2H), 7.08-7.02 (m, 7H), 6.74 (s, 1H), 4.71 (d, $^2J_{H-H}$ = 19.7 Hz, 1H), 4.63 (d, $^2J_{H-H}$ = 19.8 Hz, 1H), 3.86 (d, $^2J_{H-H}$ = 20.1 Hz, 1H), 3.53 (d, $^2J_{H-H}$ = 19.7 Hz, 1H), 3.36 (d, $^2J_{H-H}$ = 19.8 Hz, 1H), 3.18 (d, $^2J_{H-H}$ = 20.1 Hz, 1H). {^{1}H}^{13}C NMR (acetone-d_6, 151 MHz, ppm), δ_C 150.4, 150.1x2, 150.3, 149.4, 149.1, 149.0, 148.7, 147.7, 146.7, 146.1, 145.7, 144.9, 142.7, 130.4, 125.0, 124.9, 124.5, 124.4, 124.0, 123.7, 118.5, 42.2, 42.1, 42.0. ESI-HRMS (TOF) m/z [M+H]+ calcd. for $C_{33}H_{21}N$ 431.1669 found 431.1668.

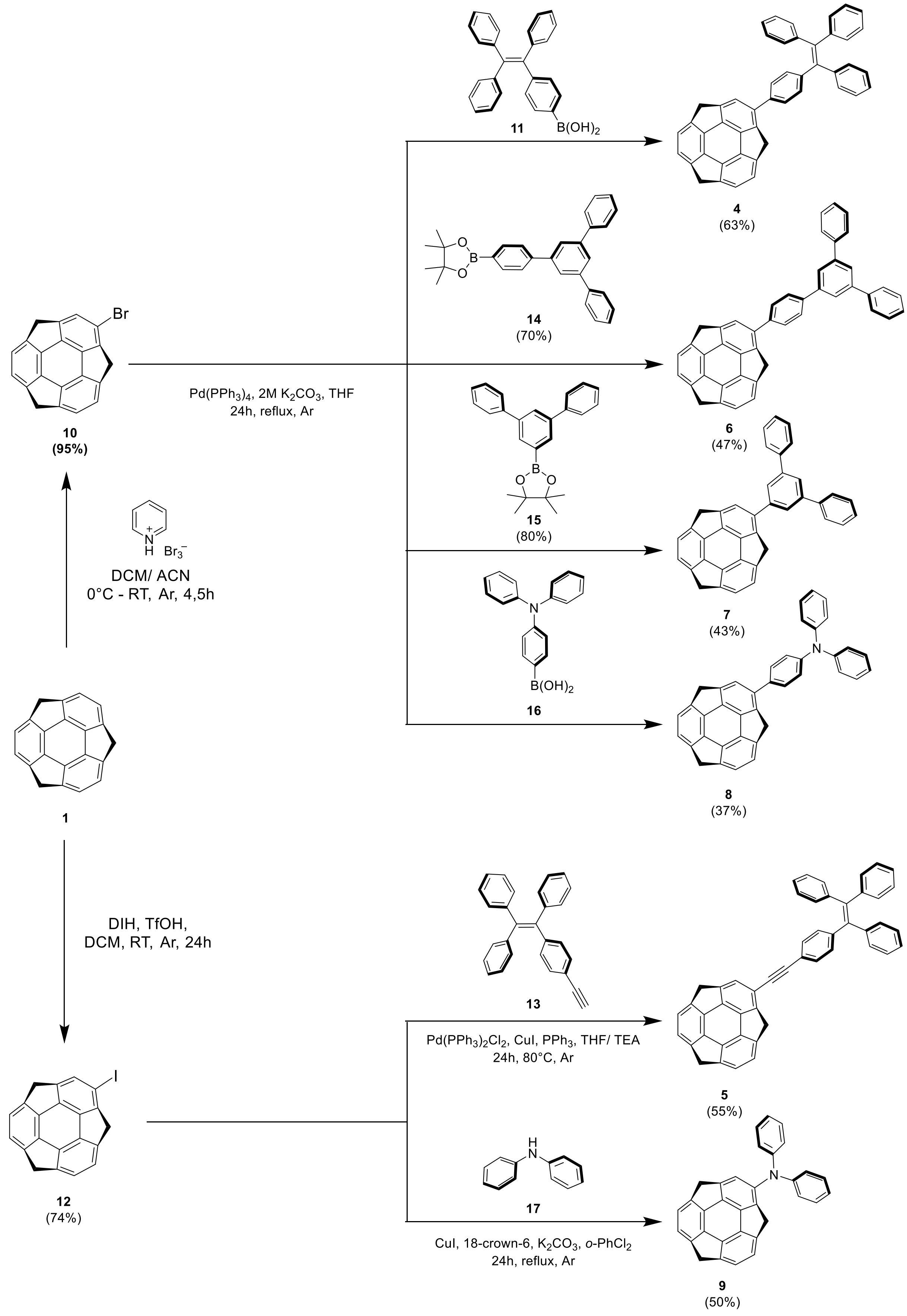

Scheme S1. General synthesis scheme of compounds **4-9**.

S1.3. Aggregation-induced emission (AIE) studies – preparation of the samples

The studies on the aggregation induced emission (AIE) behavior were performed employing measurements of the fluorescence spectra. The experiments were performed in the H_2O/THF solvent mixtures. Stock solutions of compounds **4**-**9** ($2\cdot10^{-3}$ M) in THF were diluted with proper volume of pure THF followed by addition of H_2O to reach given vol% of H_2O in the sample.

S1.4. Estimation of fluorescence quantum yield for 4-5 and their aggregates

The measurements for the estimation of fluorescence quantum yields (Φ_F) for **4-5** and their aggregates of were performed at room temperature according to the literature procedures.[6,7] Fluorescence quantum yields (Φ_F) were determined by comparison with quinine sulfate (QS) in 0.5M H_2SO_4 ($\Phi_{F,ref} = 0.5$)[8] as the standard. The measurements were performed with diluted solutions (absorbance for the highest wavelength A < 0.1 a.u.). The selected excitation wavelengths (λ_{ex}) were as follows: $C_{QS} = 2\cdot10^{-6}$ M; $C_4 = C_5 = 2\cdot10^{-6}$ M, $\lambda_{ex,4} = 320$ nm; $\lambda_{ex,5} = 344$ nm.

The following formula was used for the calculation of Φ_F:

$$\phi_{\mathrm{F}} = \phi_{\mathrm{F,ref}} \cdot \frac{F_{sample}}{F_{reference}} \cdot \frac{1-10^{-A_{\mathrm{ref}}}}{1-10^{-A_{\mathrm{sample}}}} \cdot \frac{n_{sample}^2}{n_{reference}^2}$$

where $\Phi_{F,ref}$ is the quantum yield for QS (0.551), F is the integrated area under the fluorescence spectra, A is the absorbance at the excitation wavelength, n is the refractive index of the solvent (1.346 for 0.5M H_2SO_4, 1.4072 for THF, for aggregates solution (THF/H_2O = 5:95 *vol*/*vol*) n value was taken as weighted arithmetic mean with weights equal to vol% of H_2O (n = 1.3329) and THF in the mixture). The calculated Φ_F for **4** and aggregates of **4**, were 0.0054 and 0.0382, respectively, whereas for **5** and aggregates of **5**, were 0.0096 and 0.1681, respectively.

S1.5. Receptor studies – titration experiments methodology

The anion binding experiments between compounds **4** and **5** (receptors) and cations (analytes; Li^+, Na^+, K^+, Cs^+ in form of PF_6^- salts and Rb^+ in form of BF_4^- salt) were performed employing the fluorescence spectra titration experiments. The experiments were performed in the H_2O/THF = 95:5 *v/v* system as follows. Stock solution of **4** or **5** ($2\cdot10^{-3}$ M) in THF was diluted with adequate volume of pure THF and H_2O to reach the final sample volume of 3 mL and the desired composition of solvents. The given cation was introduced to the mixture in the form of H_2O/THF = 95:5 *v/v* solutions. Each titration experiment consisted of 15 steps. First, fluorescence of a solution containing only a receptor (**4** or **5**) was measured, then solutions containing given cation were added in 14 consecutive steps to achieve the following proportions of cation to receptor: 0.1, 0.2, 0.3, 0.4, 0.5, 0.6, 0.7, 0.8, 0.9, 1.0, 2.0, 5.0, 10.0, 20.0 equiv. To ensure proper mixing, the contents of the cuvette were well-mixed using a magnetic stirrer (1200 rpm). For the additional experiments to confirm the 1:1 stoichiometry

(by Bindfit) of the dynamically formed **4**-Li^+ and **5**-Cs^+ complexes, additional respective titrations were performed for the host (**4** or **5**) concentrations in the sample of $2\cdot10^{-6}$ M and $5\cdot10^{-5}$ M. For the competitive binding experiments with receptor **4** featuring the Li^+ detection preference, the additional titrations were performed as follows. At first, the samples ($2\cdot10^{-5}$ M; final volume 3 mL) of aggregated **4** in H_2O/THF = 95:5 *v/v* solvent system were prepared. Then, 20 µL of the stock solution ($1.5\cdot10^{-2}$ M) of Li^+ or Cs^+ was added to introduce 5 equiv. of the given cation to the solution. As obtained solution was well mixed (1200 rpm) for several minutes and then titrated with the alternate cation (Cs^+ or Li^+, respectively) as noted above.

S2. NMR spectra

Compounds **4-9** featured characteristic signals coming from the sumanene skeleton, that is $H_{Ar}(C_{Ar})$, $H_{benzylic,exo}$ and $H_{benzylic,endo}$ ($C_{benzylic}$), as well as introduced polyaromatic units in the aromatic region. In the case of ^{1}H NMR spectra, signals from $H_{benzylic,exo}$ and $H_{benzylicen,endo}$ (multiplets or doublets with $^{2}J_{H-H}$=20.3-19.2 Hz) were found between 5.14-3.86 ppm and 3.69-3.18 ppm, respectively, whereas a singlet coming from H_{Ar} was found between 7.47-6.74 ppm. Due to the direct attachment of *N*,*N*-diphenylamino substituent to the sumanene skeleton in **9**, the H_{Ar} signal for **9** was the most shielded (δ_H=6.74 ppm) among **4-9**. In the case of {^{1}H}^{13}C NMR spectra analyses, the characteristic three singlets coming from $C_{benzylic}$ were found between 43.27-41.22 ppm. The expected number of signals in the {^{1}H}^{13}C NMR spectra of each compound also conformed to the anticipated values.

The comparison of the selected signals in the ^{1}H and {^{1}H}^{13}C NMR spectra of the sumanene derivatives **4-9** are presented in **Table S1** and **Table S2**, respectively.

All the ^{1}H, ^{1}H-^{1}H COSY and {^{1}H}^{13}C NMR spectra of **4-9** are presented below.

Table S1. Comparison of the selected signals in ^{1}H NMR spectra of compounds **4-9**.

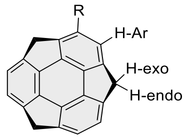

cpd.	^{1}H NMR Spectrum (600 MHz)				
	structure	solvent	d_{H-Ar} (s, 1H, ppm)	d_{H-exo} (ppm)	d_{H-endo} (ppm)
4	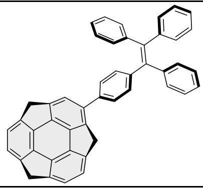	$CDCl_3$	N/D[a]	4.83<br>4.78–4.65	3.47<br>3.41<br>3.28
5	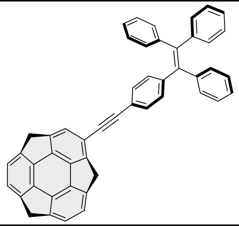	$(CD_3)_2SO$	7.27	4.77<br>4.71	3.69–3.47
6	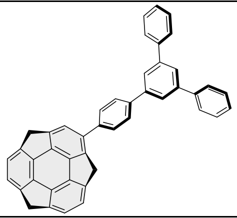	$(CD_3)_2SO$	7.22	5.14<br>4.80<br>4.71	3.67<br>3.51<br>3.44
7	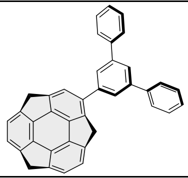	$(CD_3)_2CO$	7.69	5.13<br>4.81<br>4.73	3.66<br>3.51<br>3.47
8	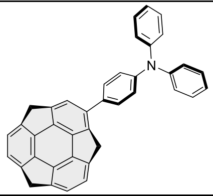	$(CD_3)_2CO$	7.47	5.00<br>4.77<br>4.71	3.58<br>3.50<br>3.39
9	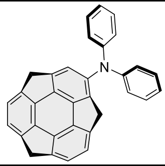	$(CD_3)_2CO$	6.74	4.71<br>4.63<br>3.86	3.53<br>3.36<br>3.18

[a] Included in a multiplet.

Table S2. Comparison of the signals for $C_{CH2\text{-benzylic}}$ in {^{1}H}^{13}C NMR spectra of compounds **4-9**.

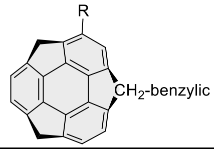

**cpd.**	**{^{1}H}^{13}C NMR Spectrum (151 MHz)**		
	**structure**	**solvent**	**d$_{CH2\text{-benzylic}}$ (ppm)**
**4**	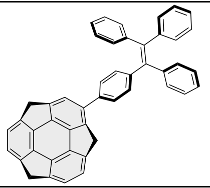	$CDCl_3$	42.8<br>41.9x2
**5**	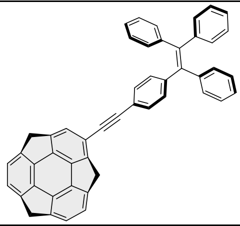	$(CD_3)_2SO$	41.4<br>41.3<br>41.2
**6**	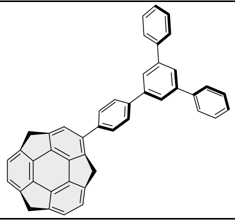	$(CD_3)_2SO$	42.4<br>41.5<br>41.3
**7**	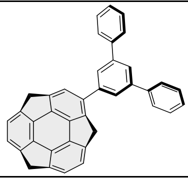	$(CD_3)_2CO$	42.4x2<br>42.3
**8**	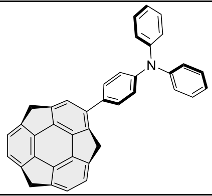	$(CD_3)_2CO$	43.3<br>42.2<br>42.1
**9**	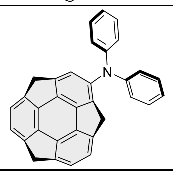	$(CD_3)_2CO$	42.2<br>42.1<br>42.0

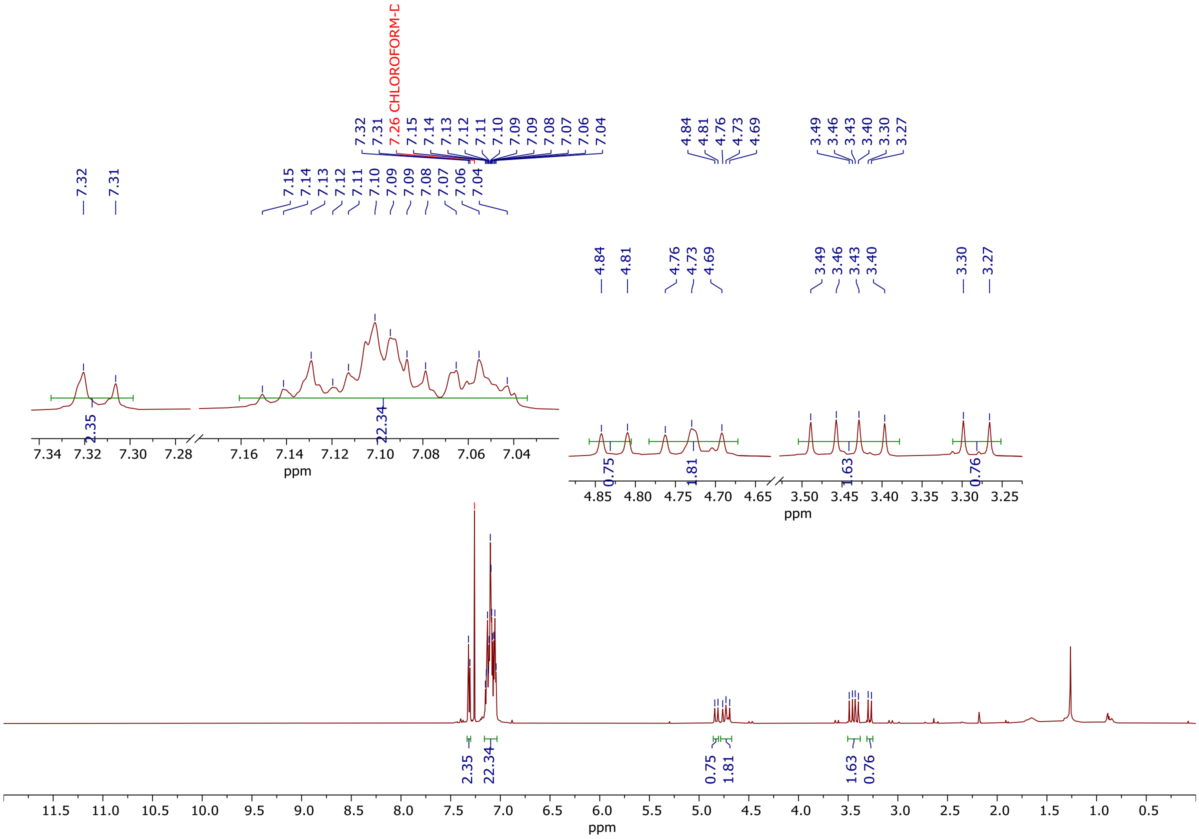

Fig. S1. ^{1}H NMR spectrum (600 MHz, $CDCl_3$) of compound **4**.

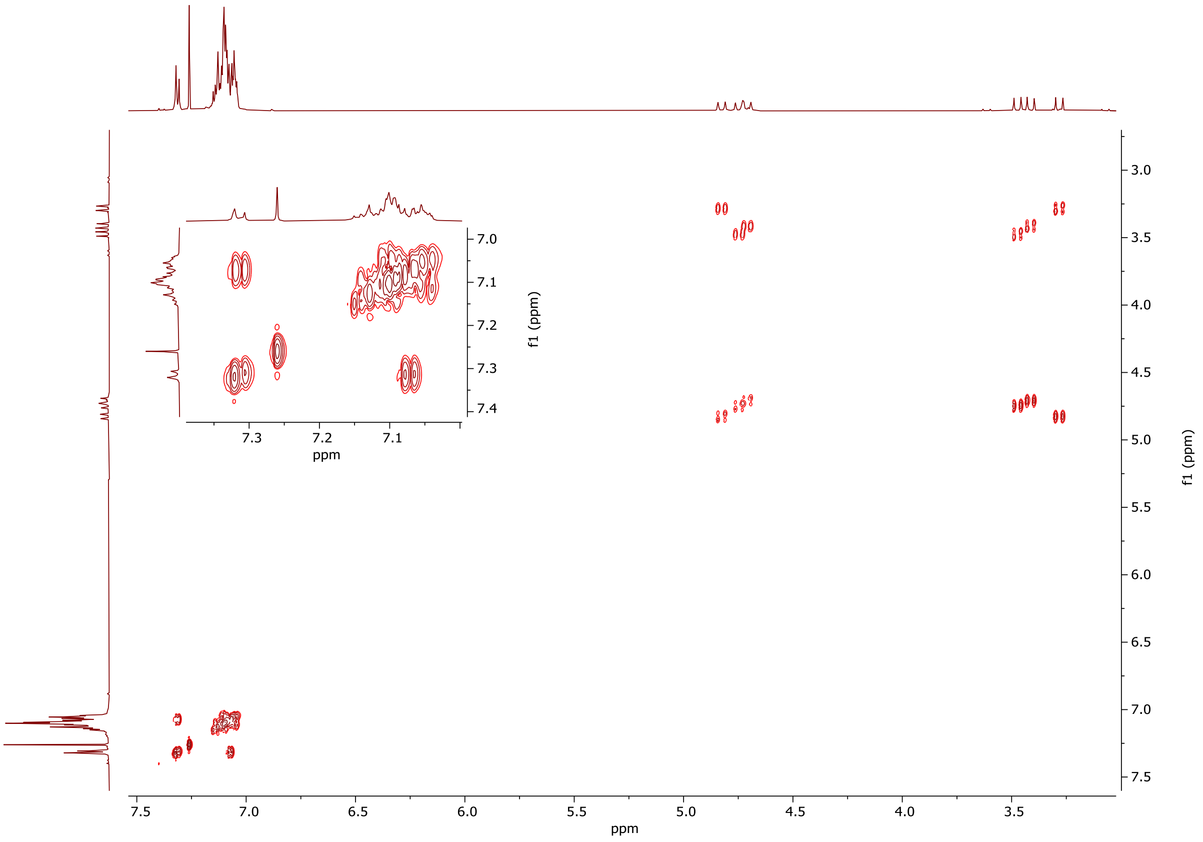

Fig. S2. ^{1}H-^{1}H COSY NMR spectrum (600 MHz, $CDCl_3$) of compound **4**.

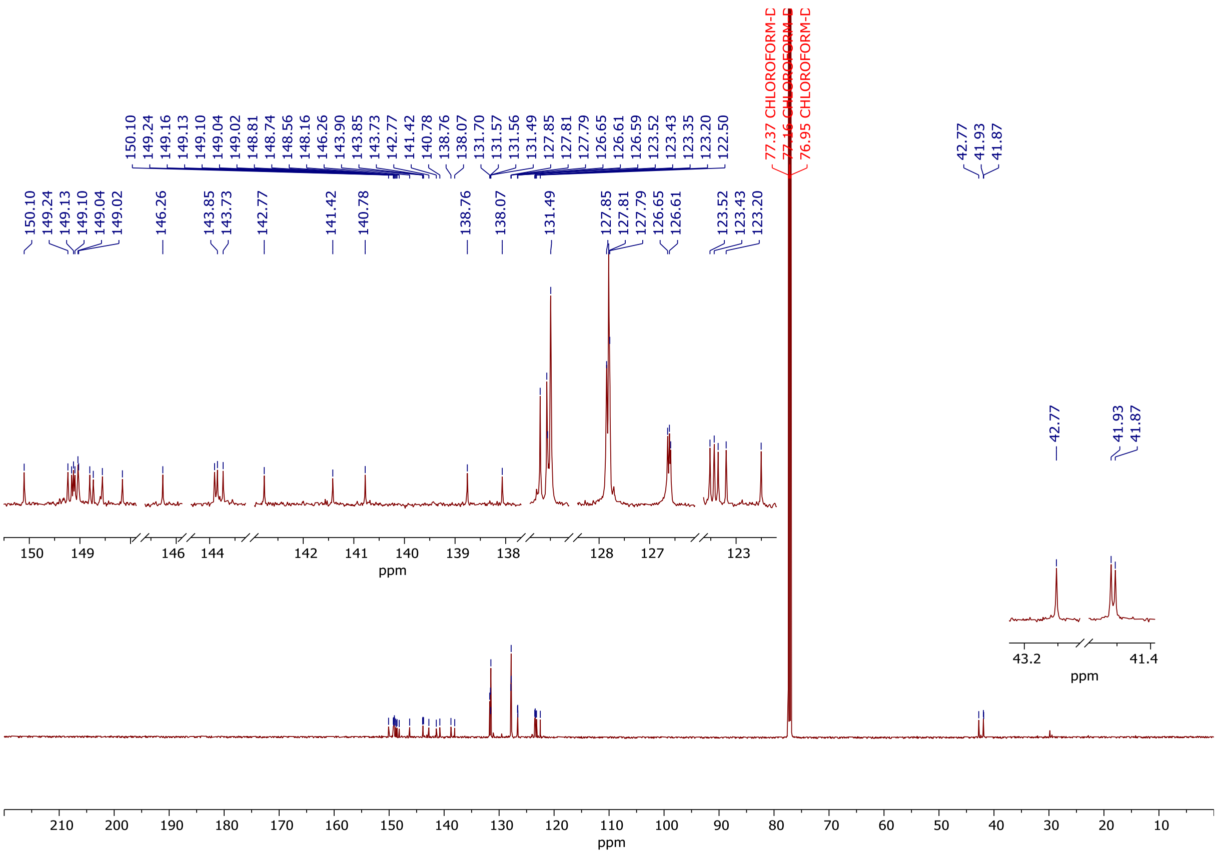

Fig. S3. {^{1}H}^{13}C NMR spectrum (151 MHz, $CDCl_3$) of compound **4**.

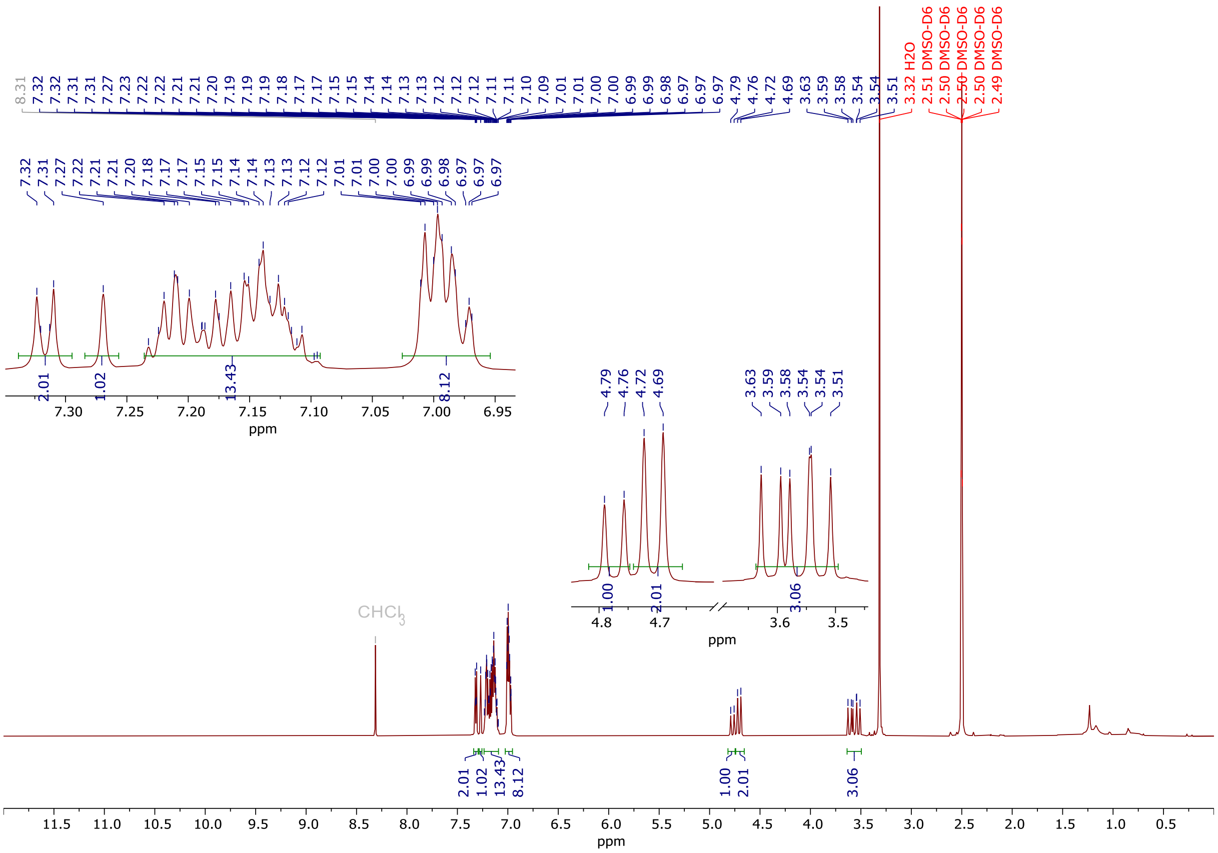

Fig. S4. ^{1}H NMR spectrum (600 MHz, DMSO-d_6) of compound **5**.

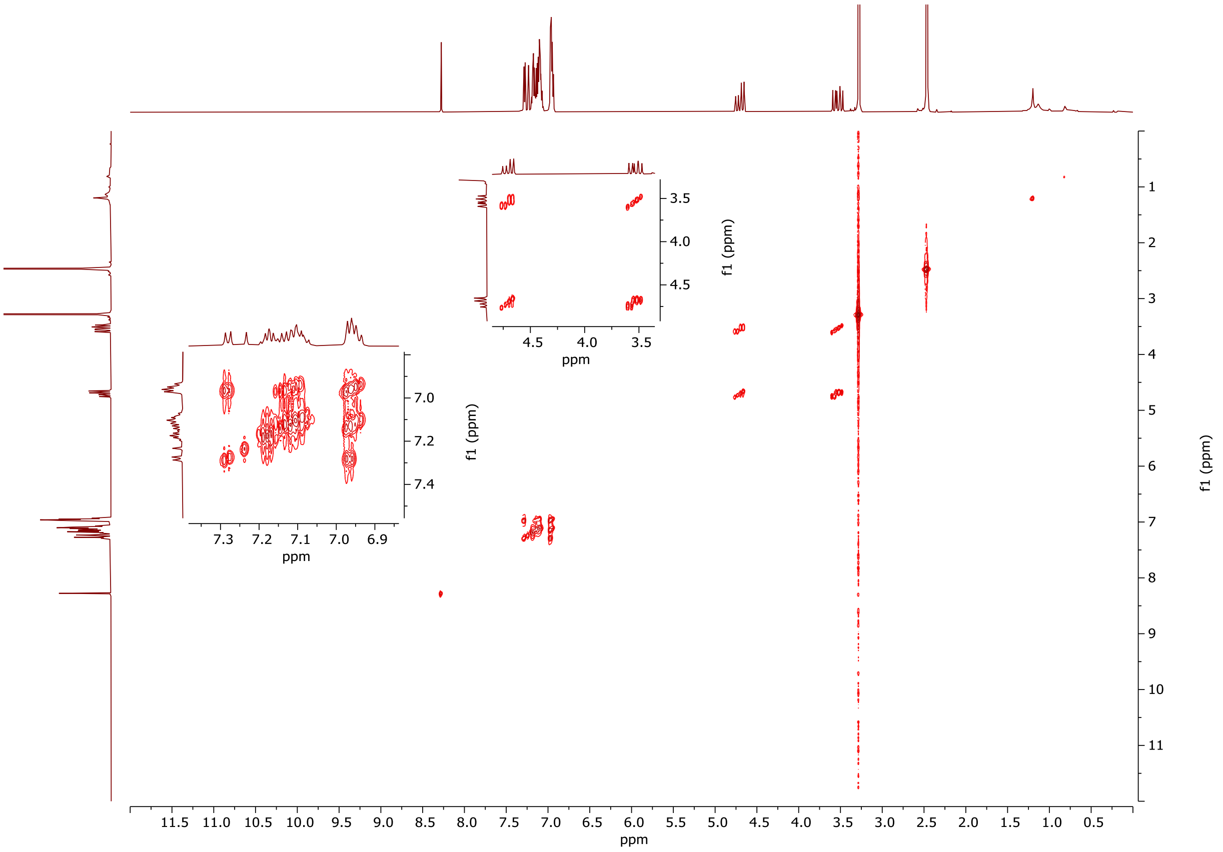

Fig. S5. ^{1}H-^{1}H COSY NMR spectrum (600 MHz, DMSO-d_6) of compound **5**.

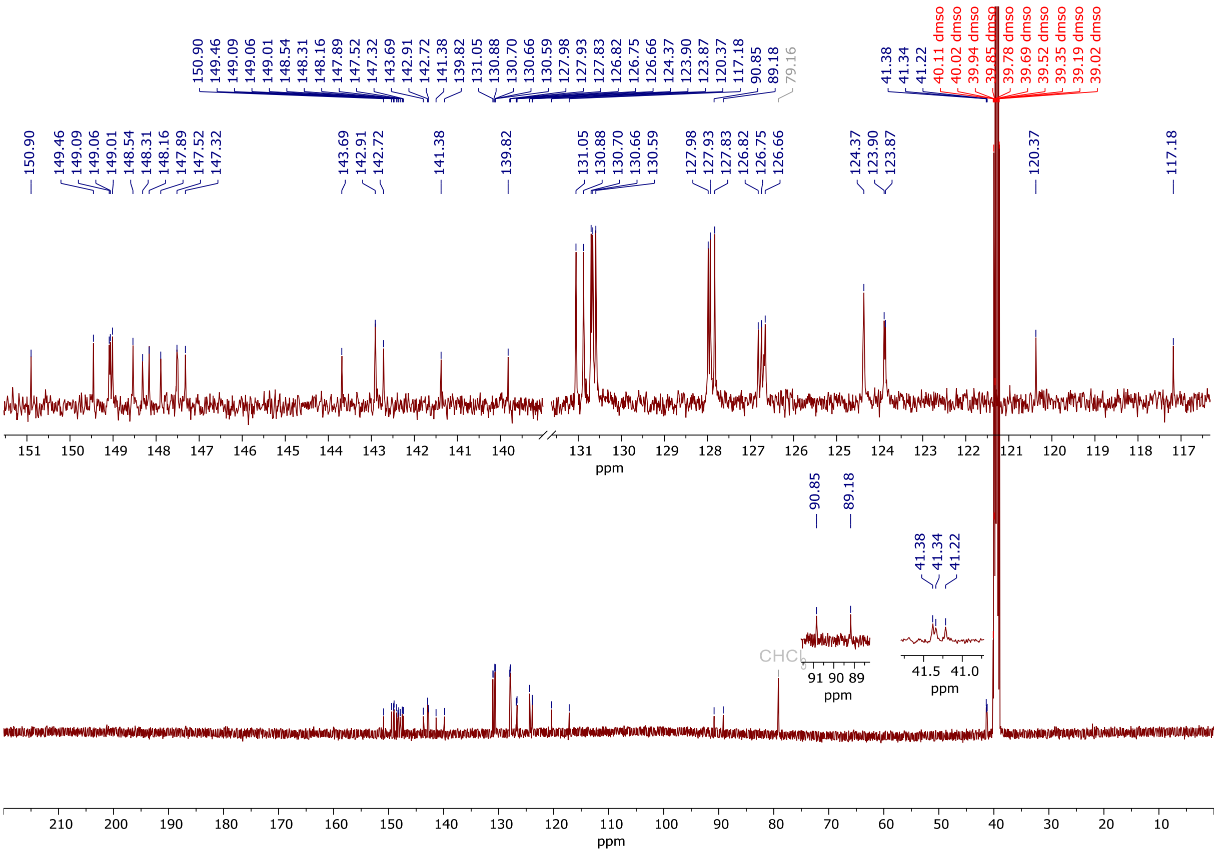

Fig. S6. {^{1}H}^{13}C NMR spectrum (151 MHz, DMSO-d_6) of compound **5**.

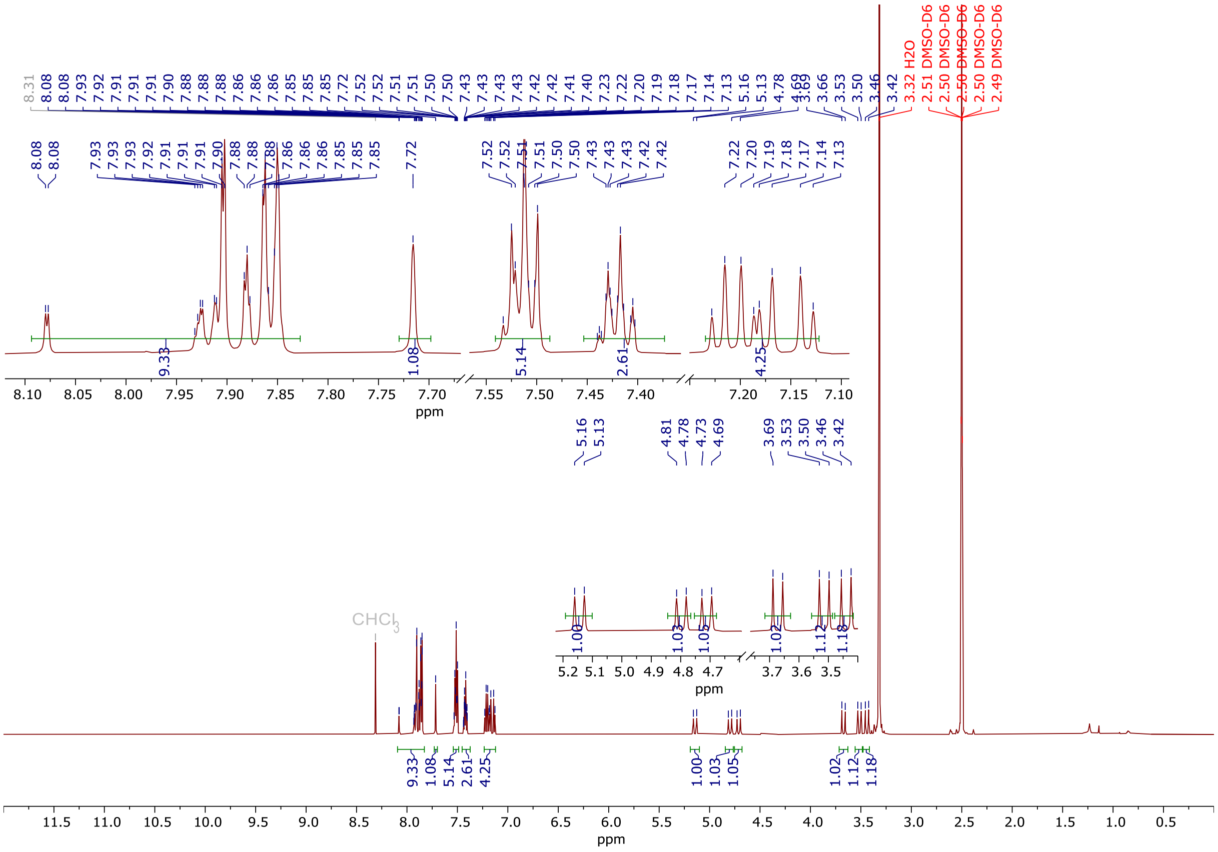

Fig. S7. ^{1}H NMR spectrum (600 MHz, DMSO-d_6) of compound **6**.

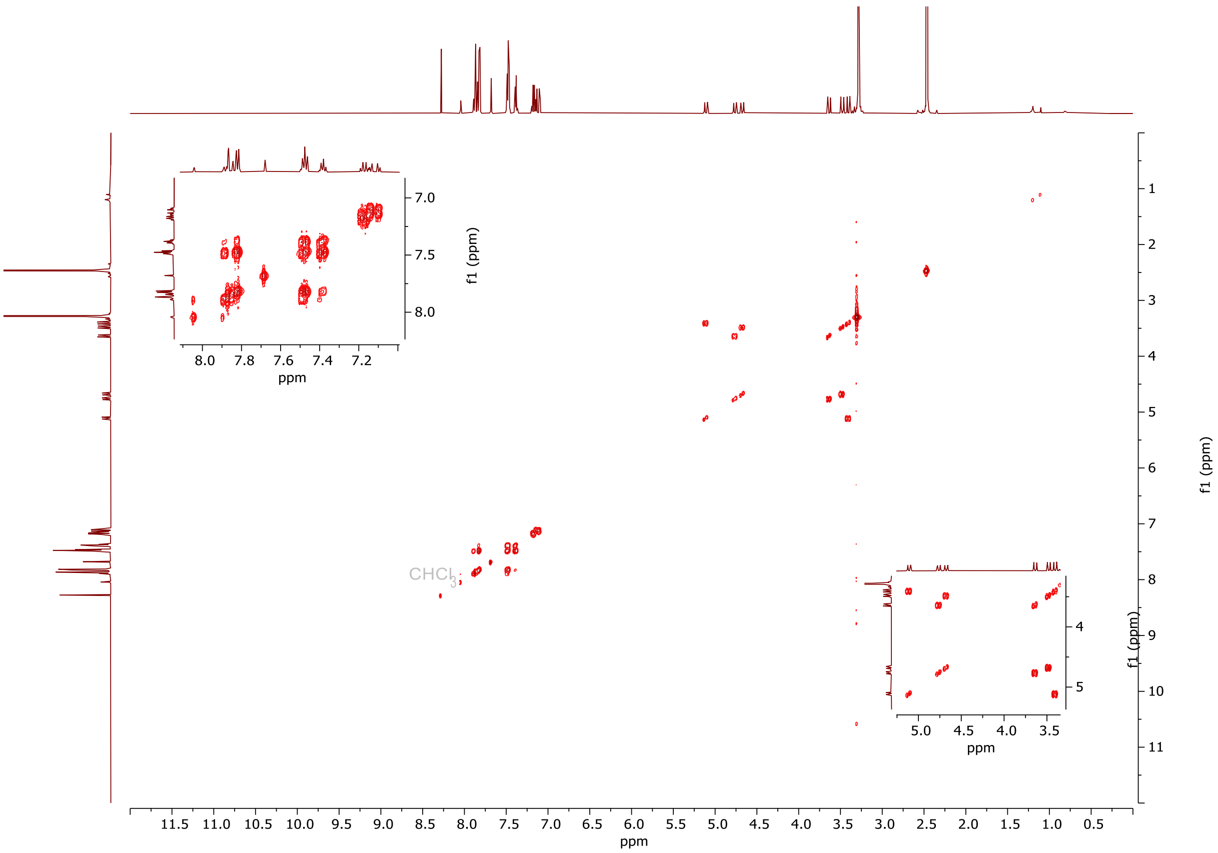

Fig. S8. ^{1}H-^{1}H COSY NMR spectrum (600 MHz, DMSO-d_6) of compound **6**.

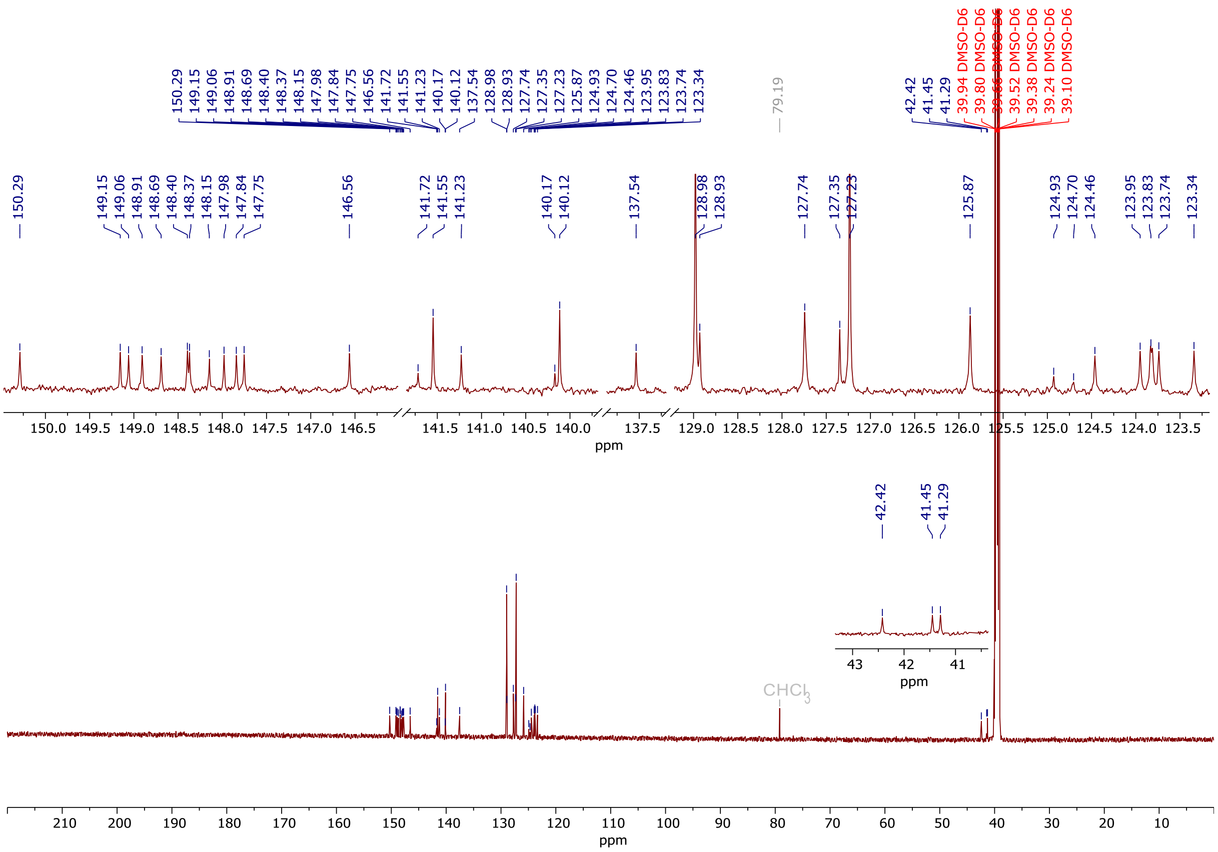

Fig. S9. {^{1}H}^{13}C NMR spectrum (151 MHz, DMSO-d_6) of compound **6**.

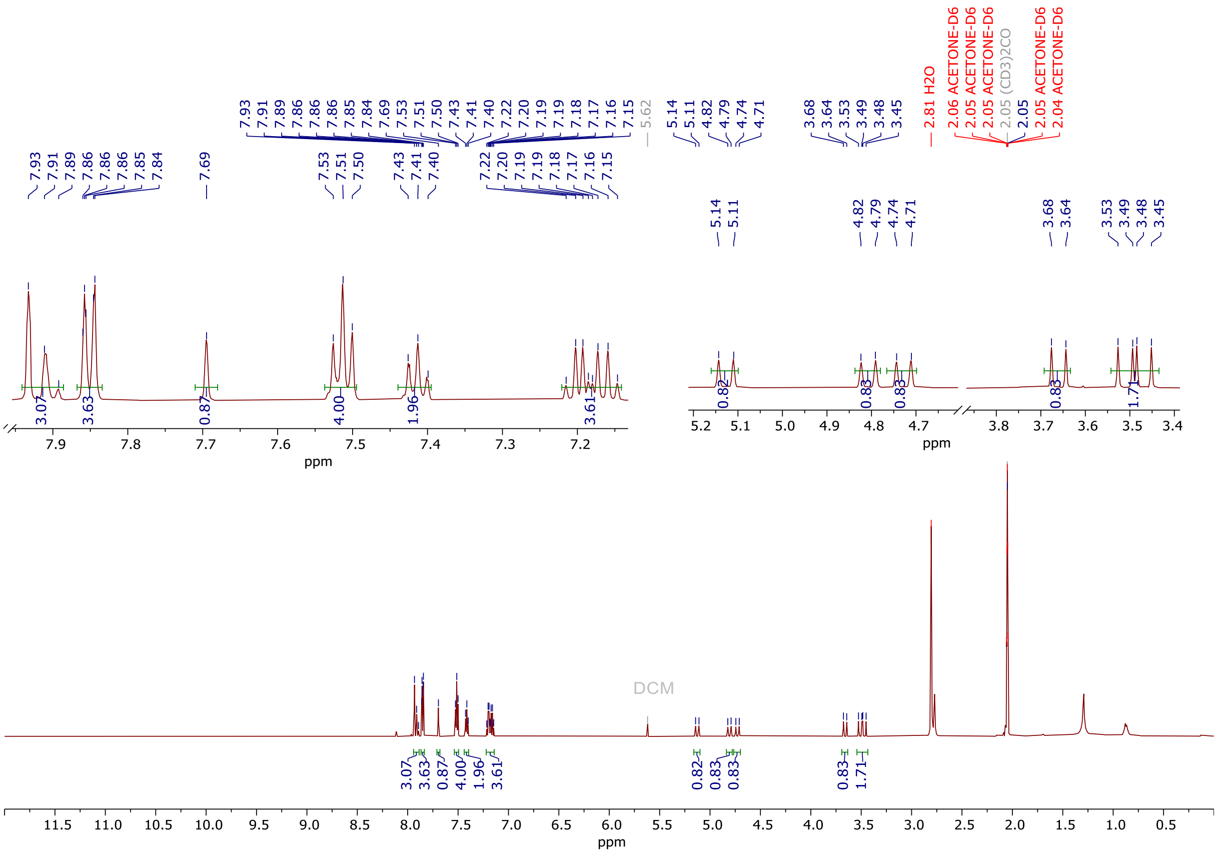

Fig. S10. ^{1}H NMR spectrum (600 MHz, acetone-d_6) of compound **7**.

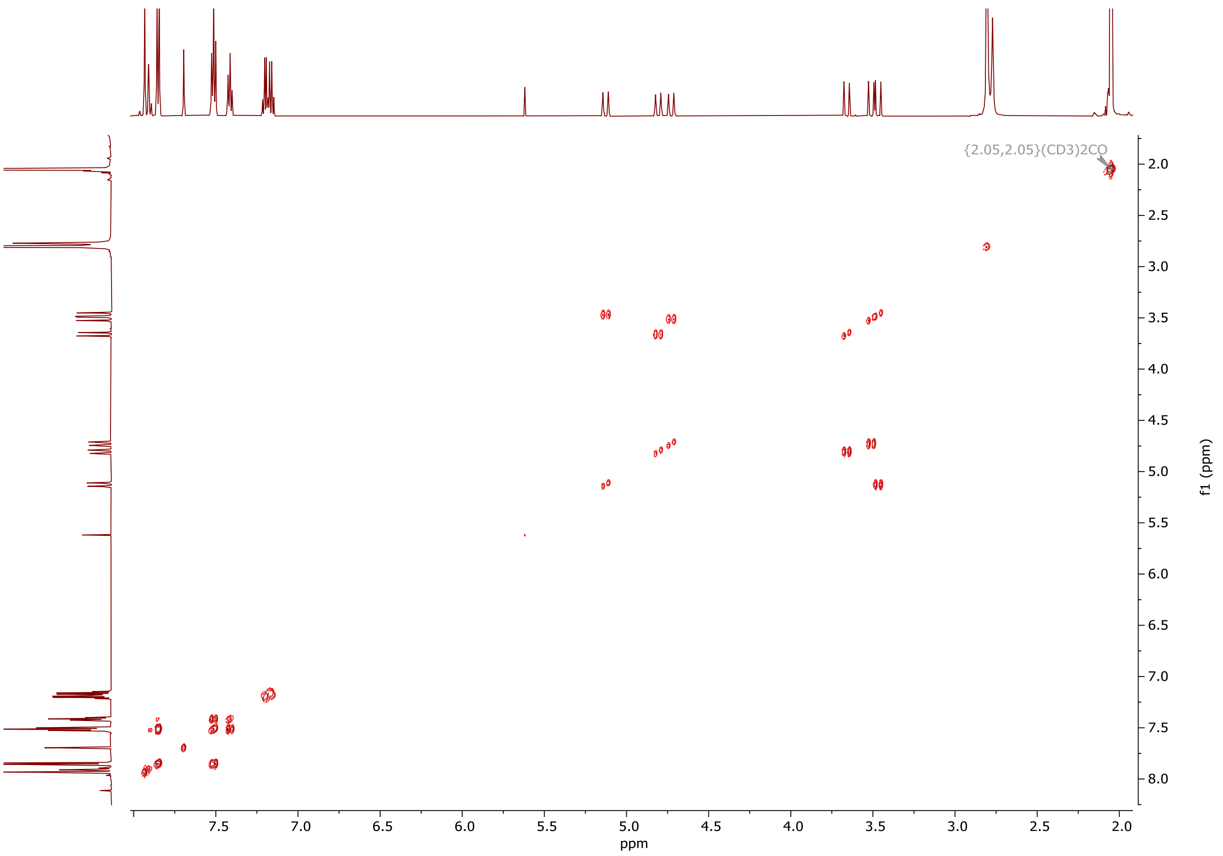

Fig. S11. ^1H-^1H COSY NMR spectrum (600 MHz, acetone-d_6) of compound **7**.

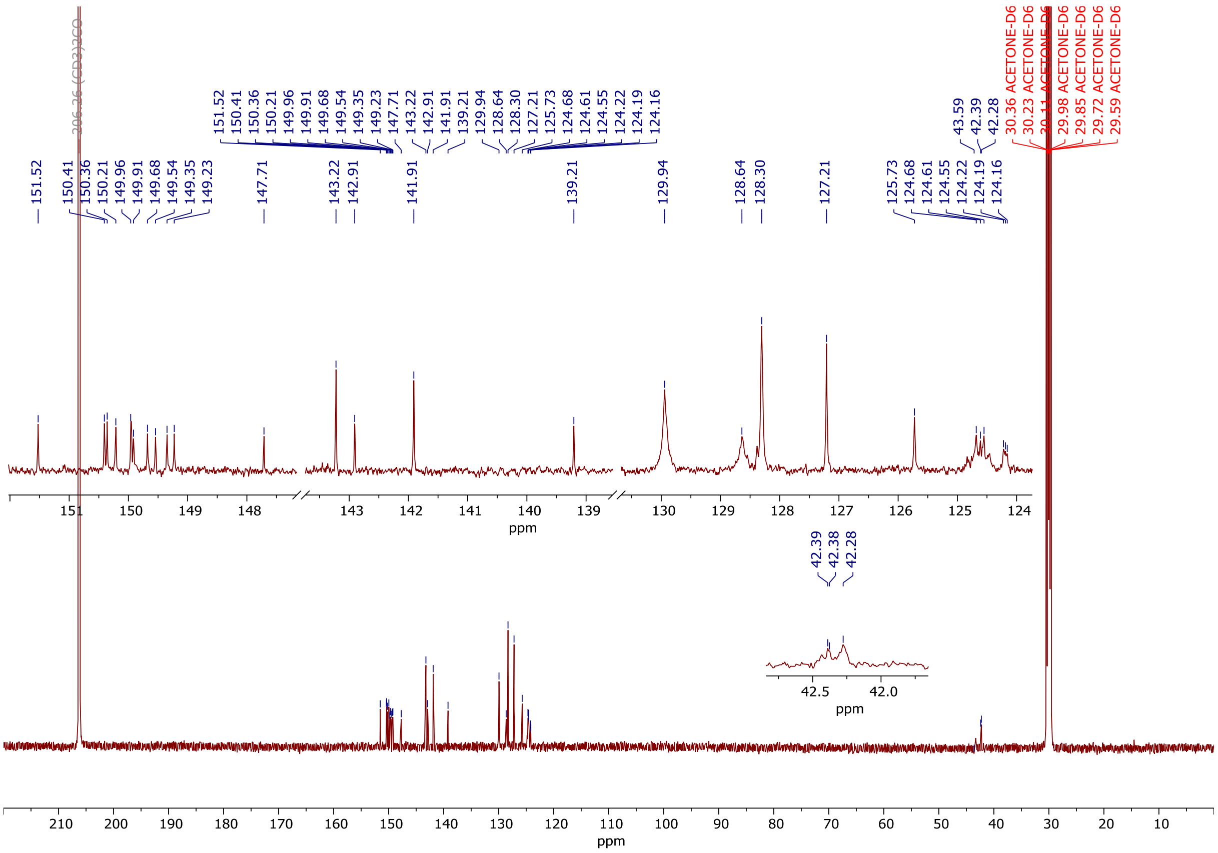

Fig. S12. {^1H}^{13}C NMR spectrum (151 MHz, acetone-d_6) of compound **7**.

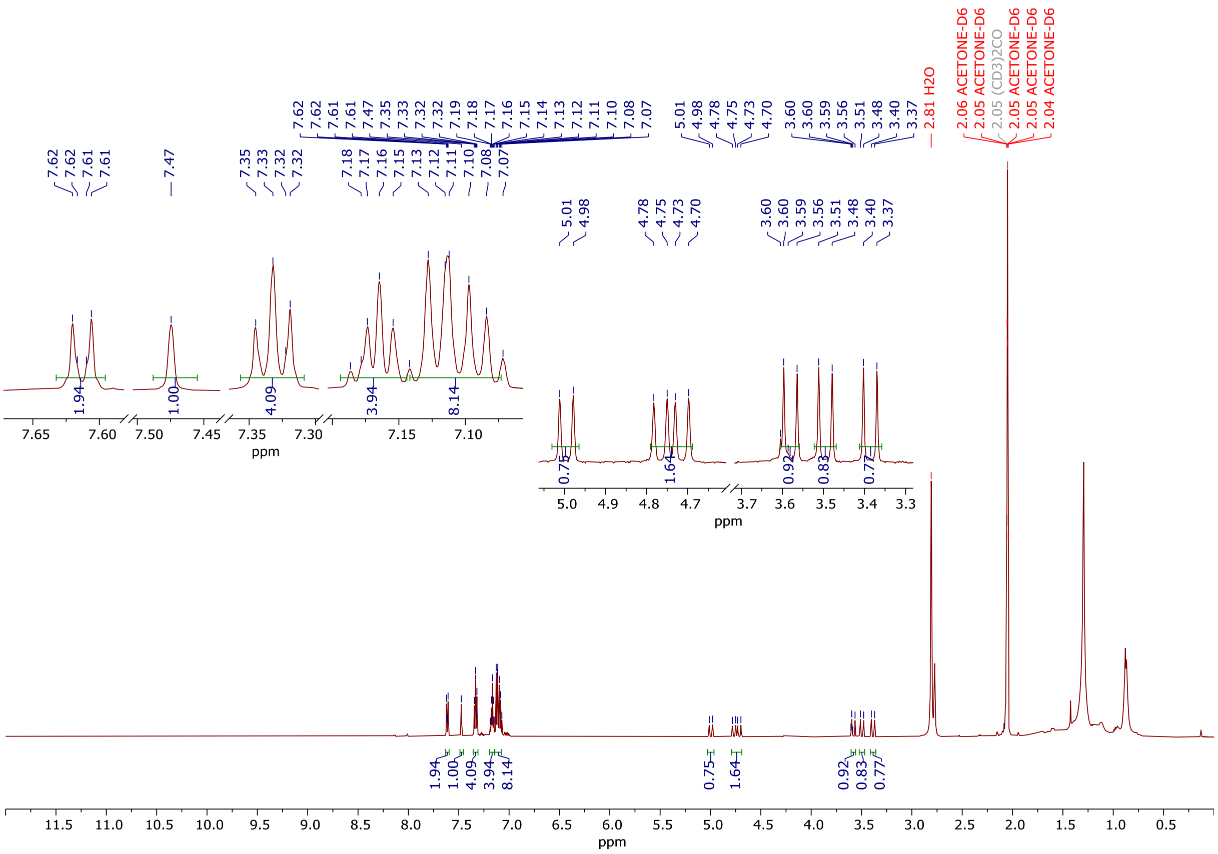

Fig. S13. ^{1}H NMR spectrum (600 MHz, acetone-d_6) of compound **8**.

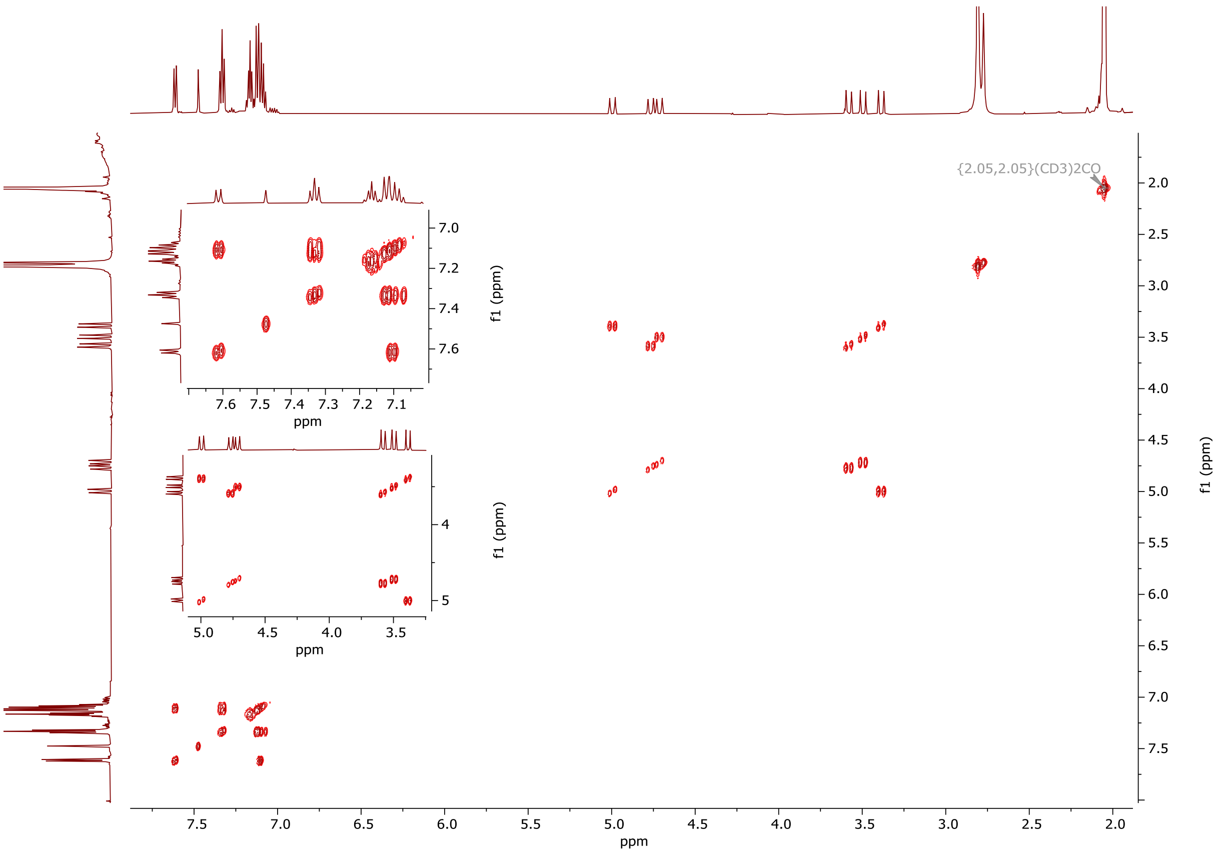

Fig. S14. ^{1}H-^{1}H COSY NMR spectrum (600 MHz, acetone-d_6) of compound **8**.

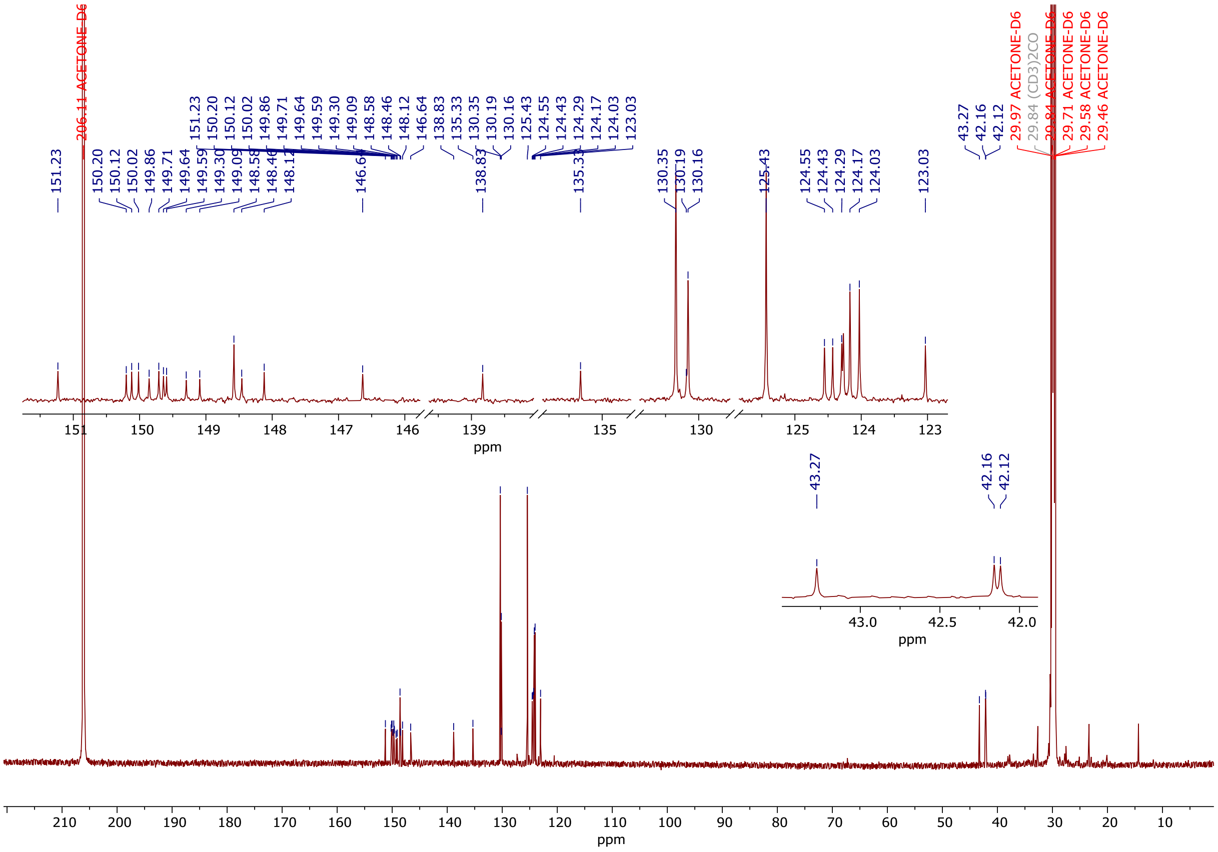

Fig. S15. {^{1}H}^{13}C NMR spectrum (151 MHz, acetone-d_6) of compound **8**.

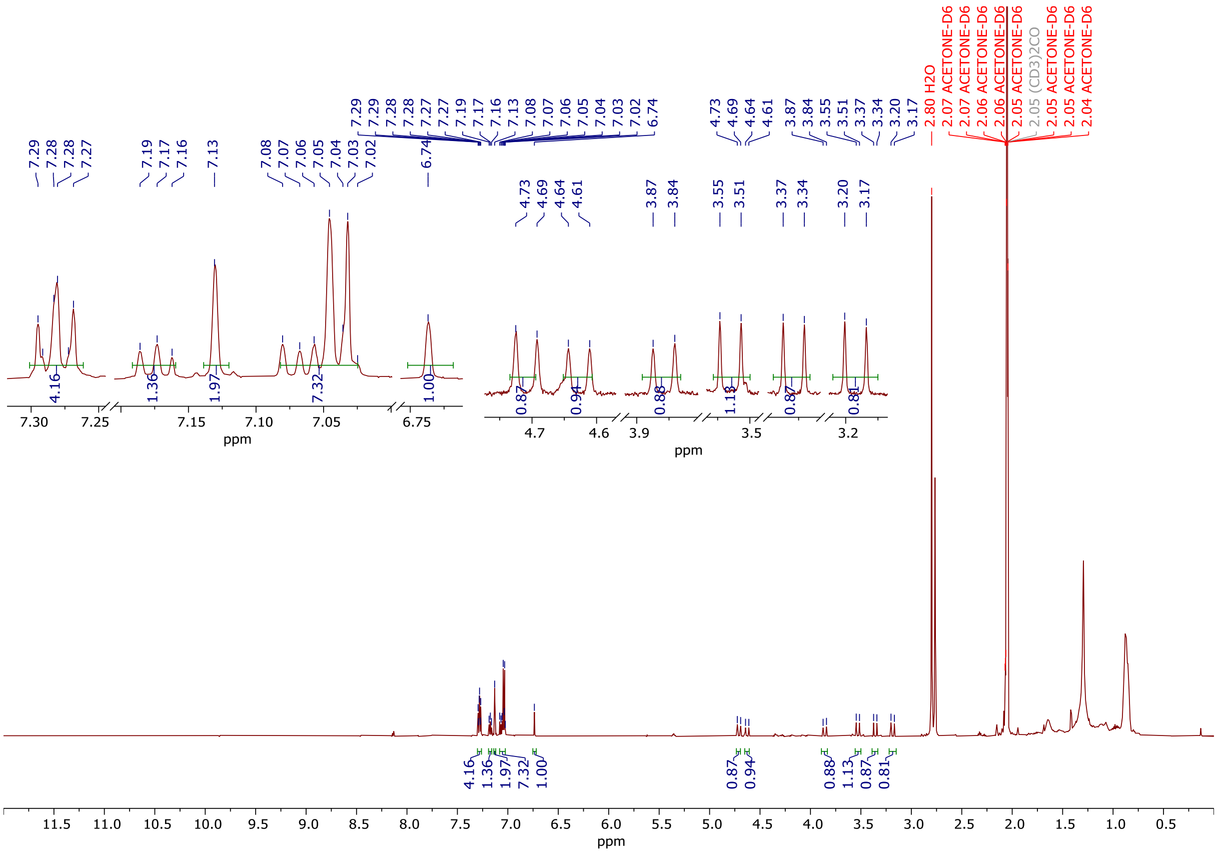

Fig. S16. ^{1}H NMR spectrum (600 MHz, acetone-d_6) of compound **9**.

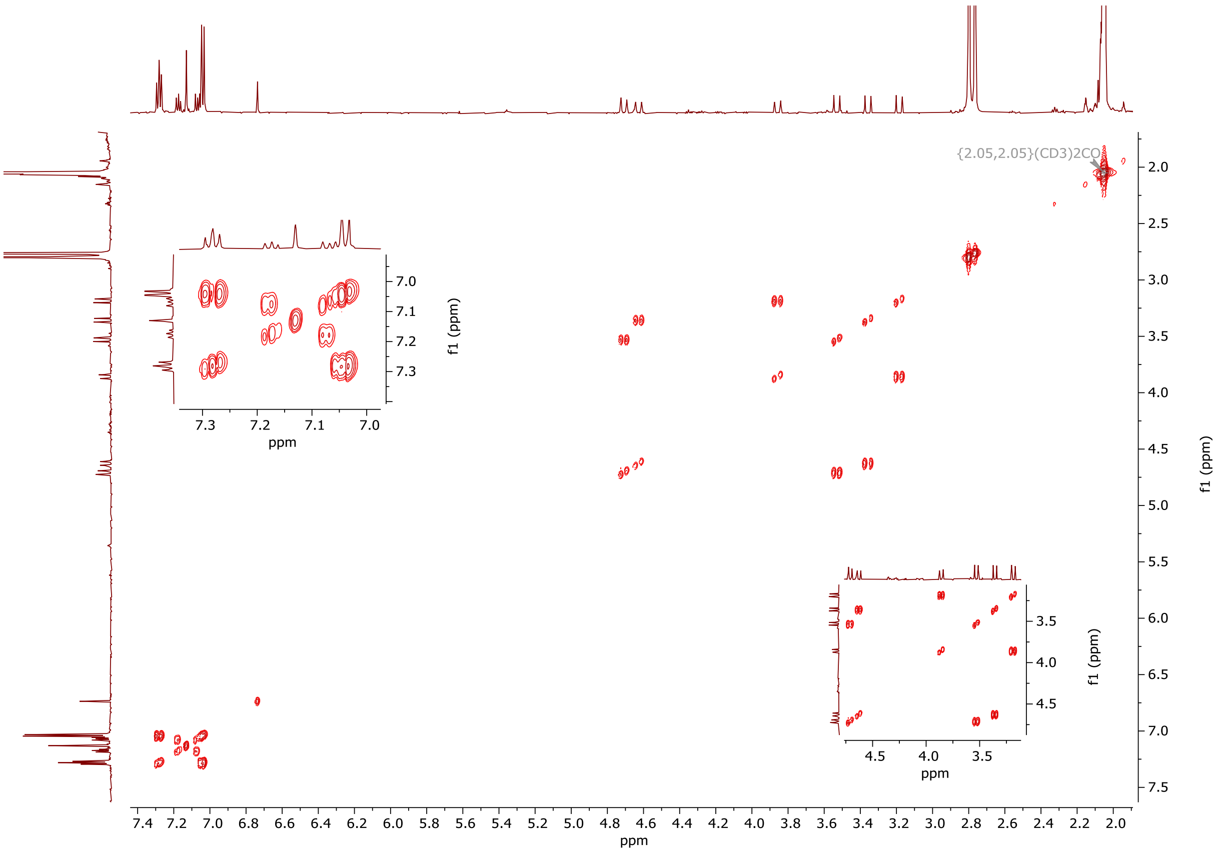

Fig. S17. ^{1}H-^{1}H COSY NMR spectrum (600 MHz, acetone-d_6) of compound **9**.

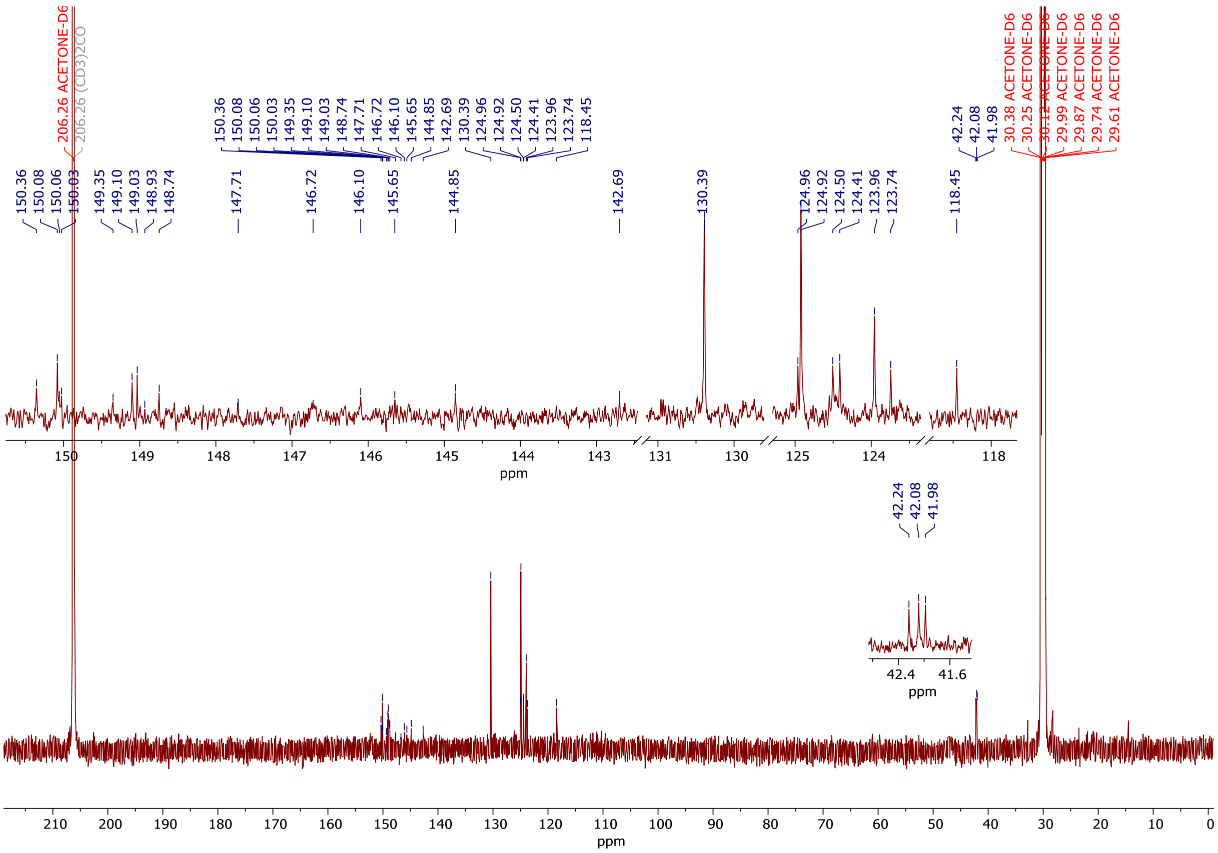

Fig. S18. {^{1}H}^{13}C NMR spectrum (151 MHz, acetone-d_6) of compound **9**.

S3. HRMS spectra

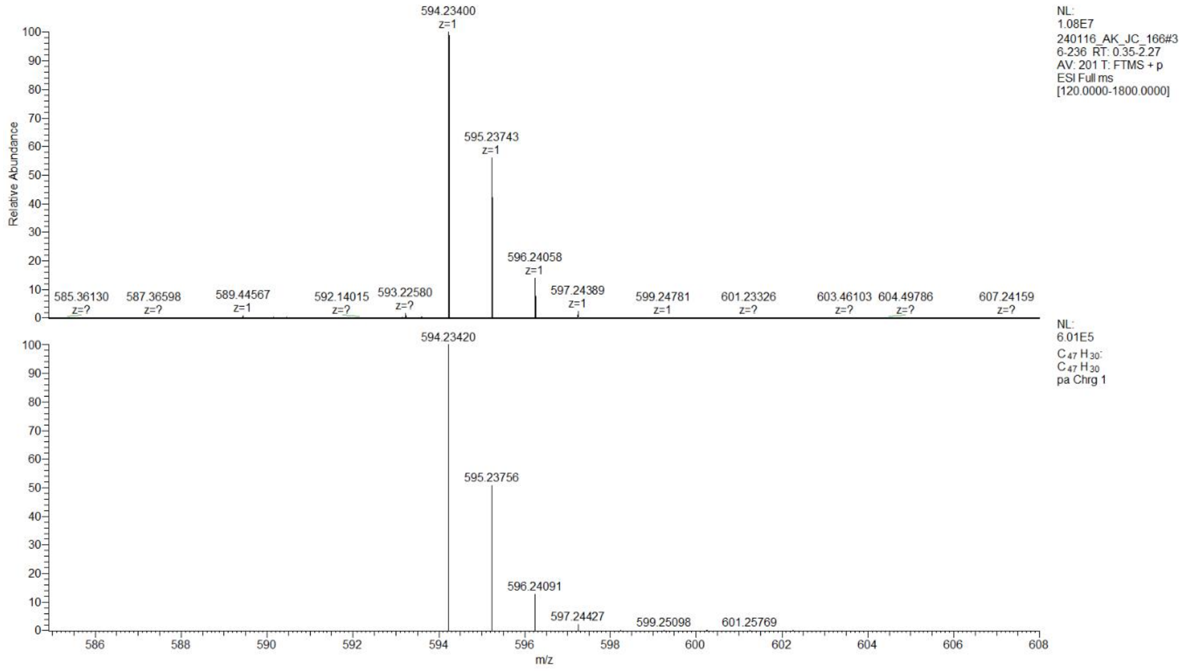

Fig. S19. ESI-HRMS (TOF) spectrum of compound **4**.

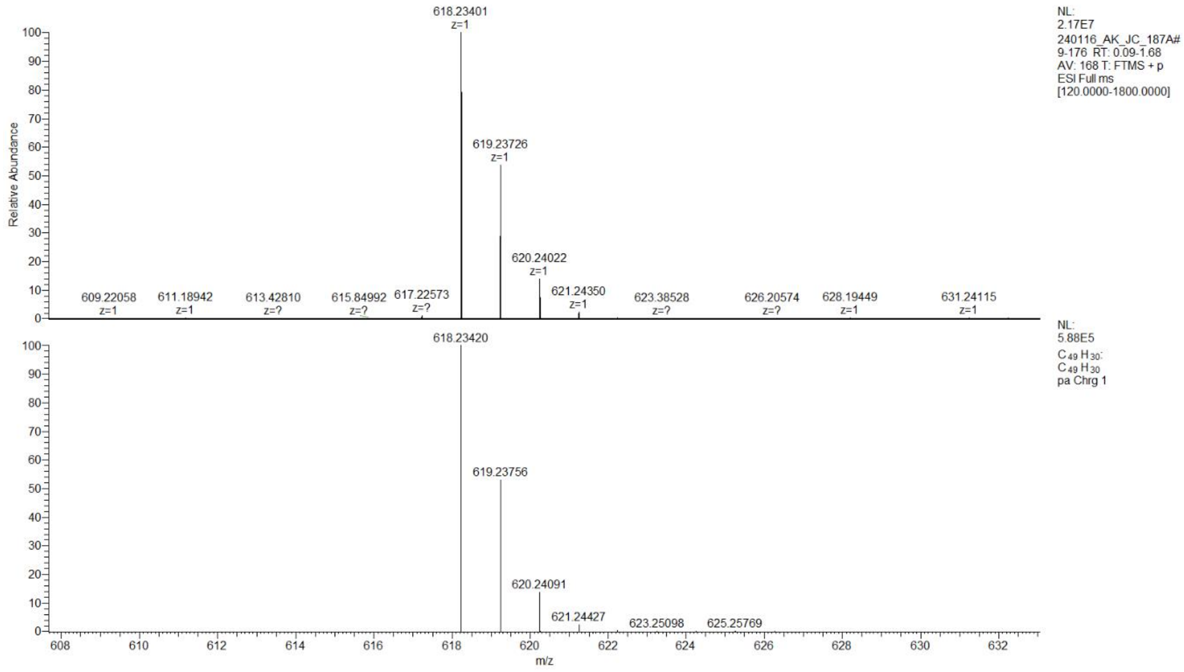

Fig. S20. ESI-HRMS (TOF) spectrum of compound **5**.

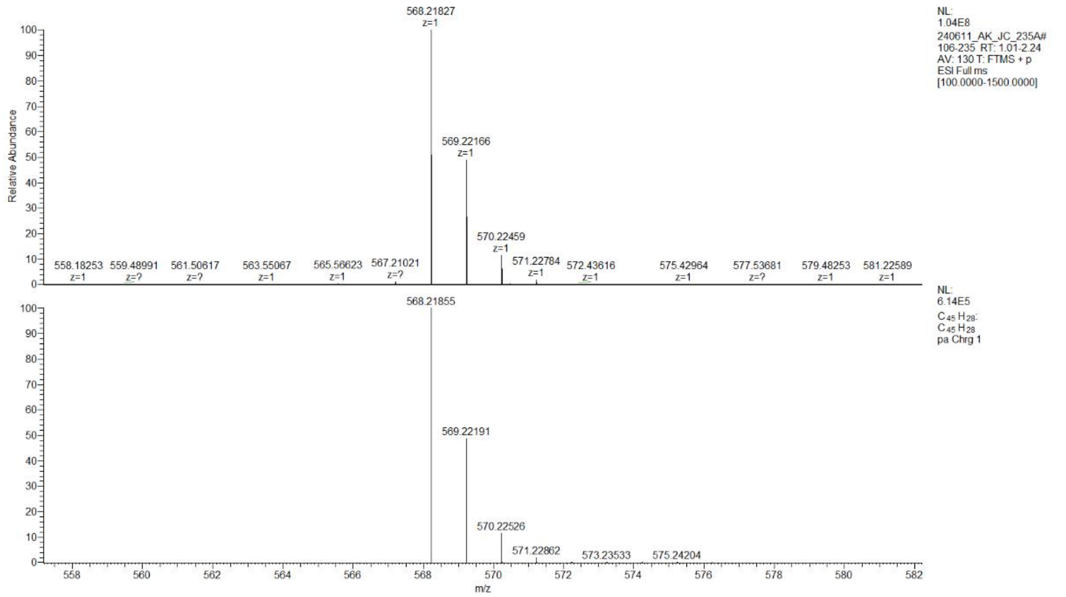

Fig. S21. ESI-HRMS (TOF) spectrum of compound **6**.

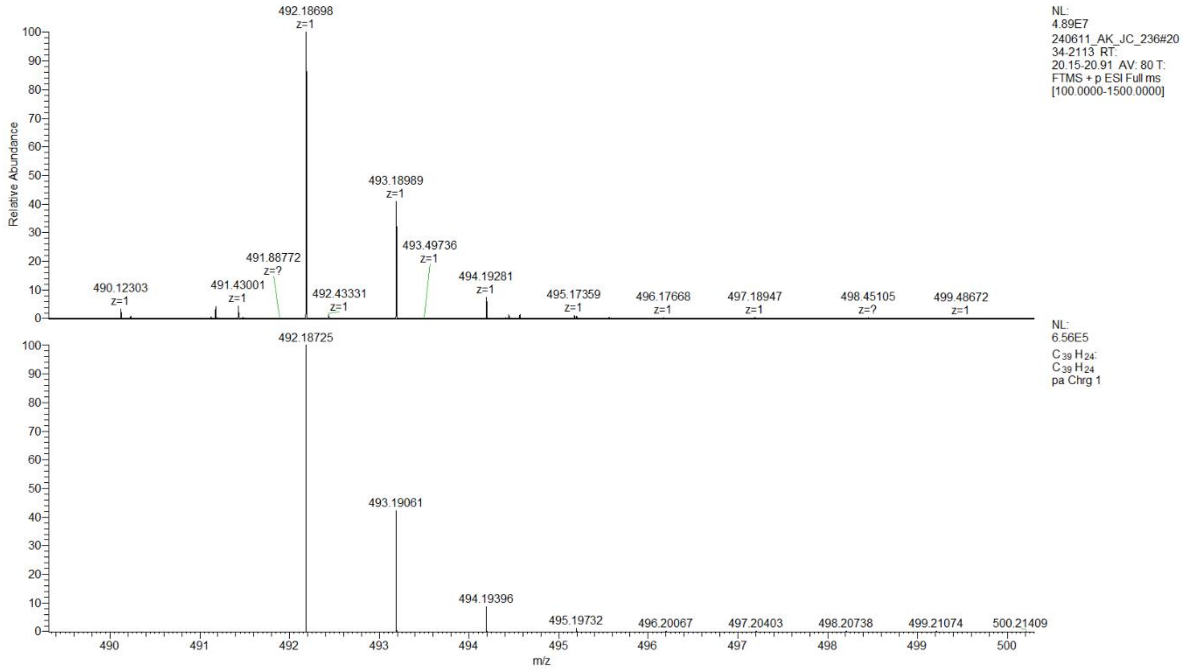

Fig. S22. ESI-HRMS (TOF) spectrum of compound **7**.

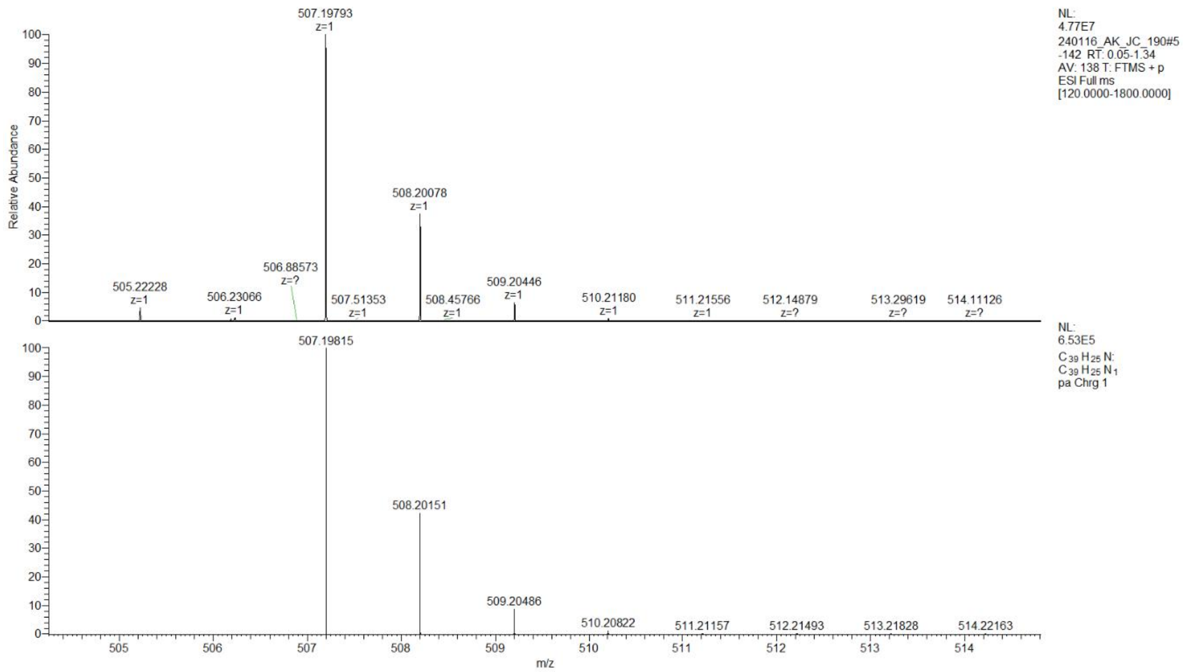

Fig. S23. ESI-HRMS (TOF) spectrum of compound **8**.

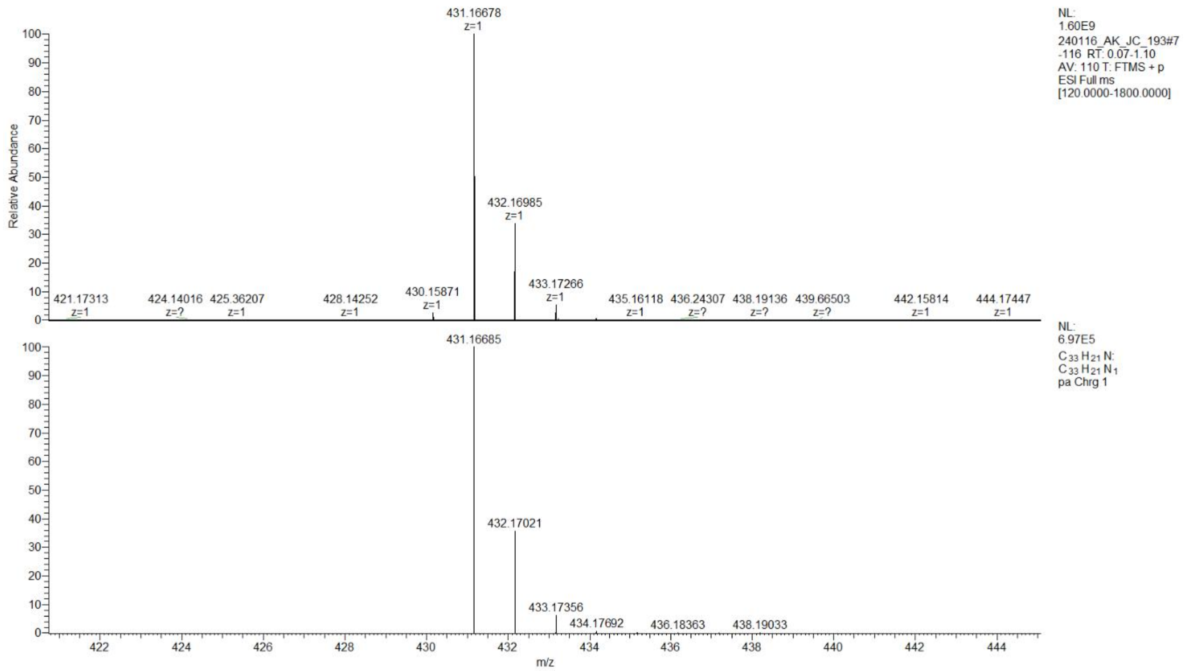

Fig. S24. ESI-HRMS (TOF) spectrum of compound **9**.

S4. Photophysical and AIE studies

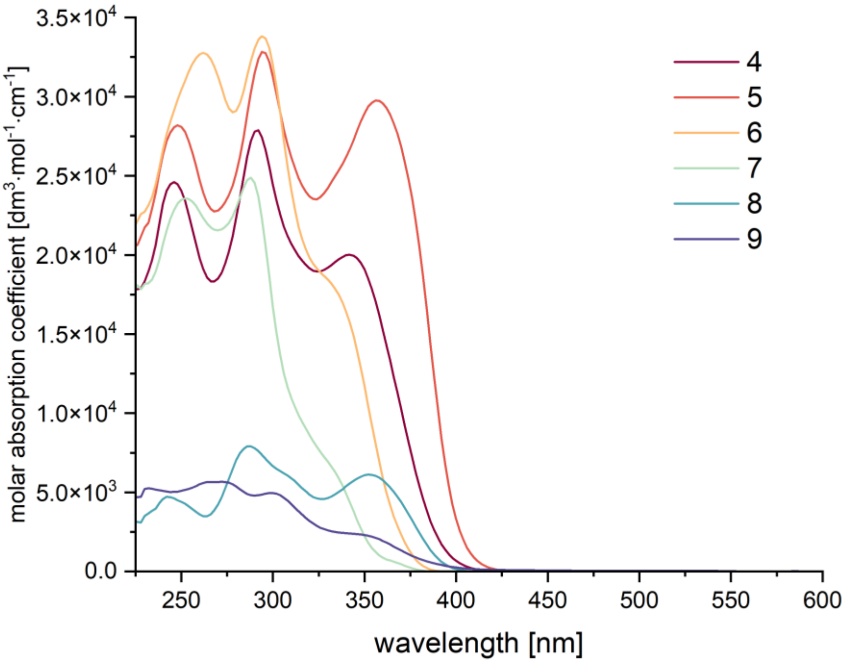

Fig. S25. UV-vis spectra of compounds **4–9** (THF, C = 2·10^{-5} M).

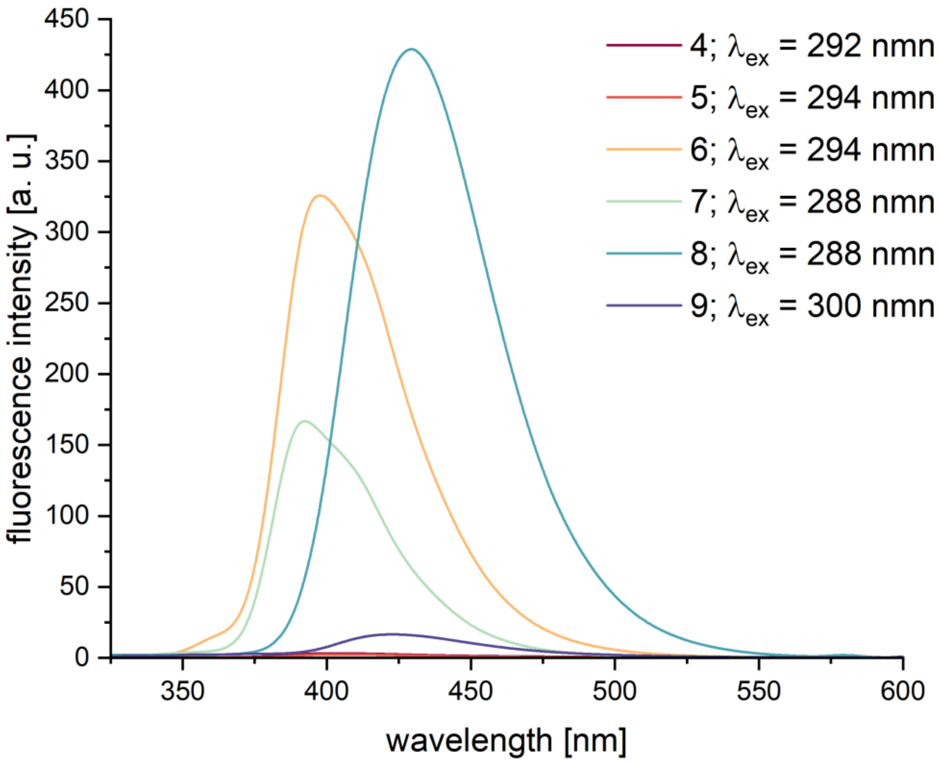

Fig. S26. Fluorescence spectra of compounds **4–9** (THF, C = 2·10^{-5} M, PMT voltage: 250 V).

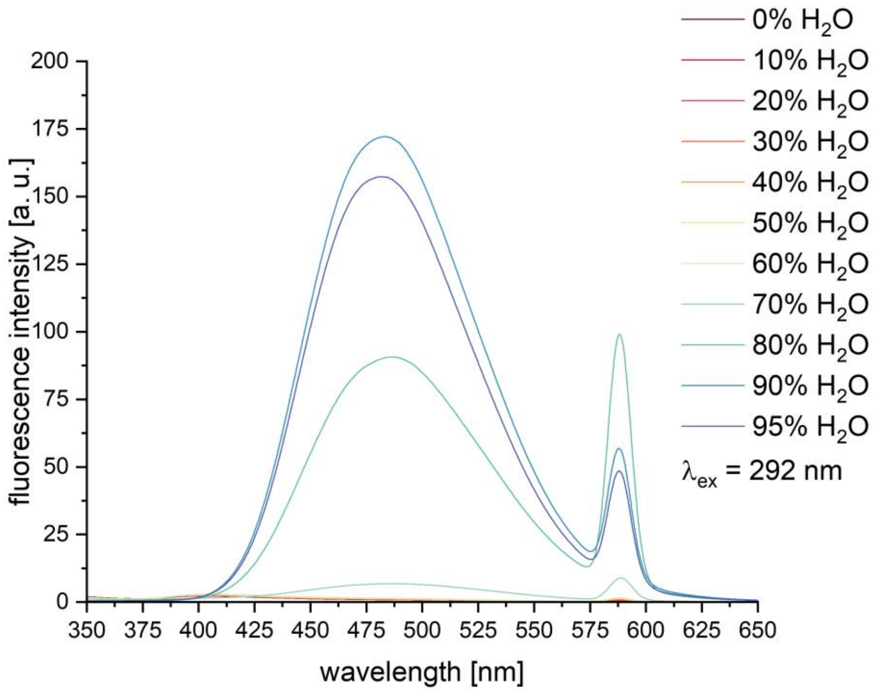

Fig. S27. Fluorescence spectra of compound **4** in H_2O/THF system containing different vol% of water in the sample ($C = 2 \cdot 10^{-5}$ M, PMT voltage: 250 V).

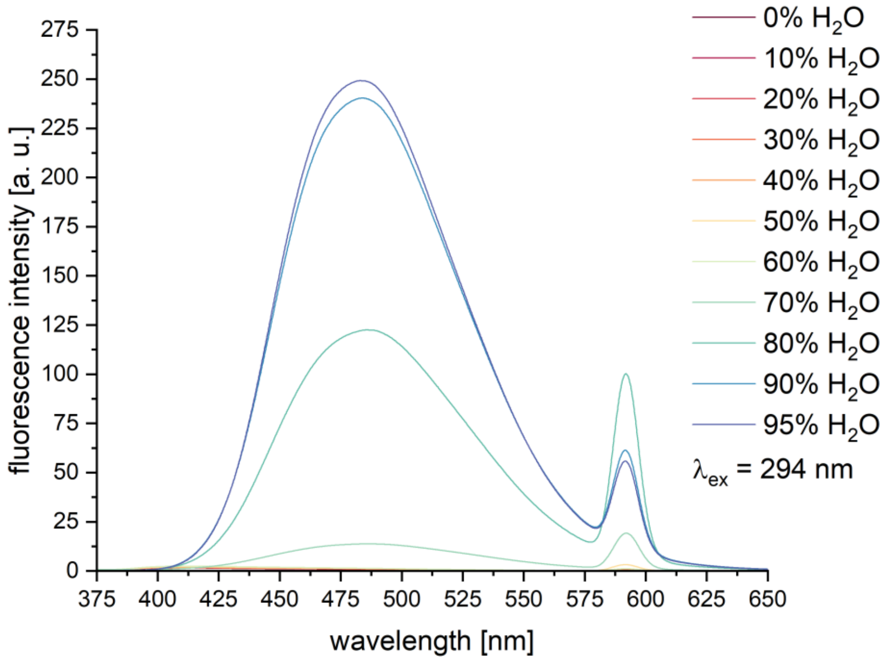

Fig. S28. Fluorescence spectra of compound **5** in H_2O/THF system containing different vol% of water in the sample ($C = 2 \cdot 10^{-5}$ M, PMT voltage: 250 V).

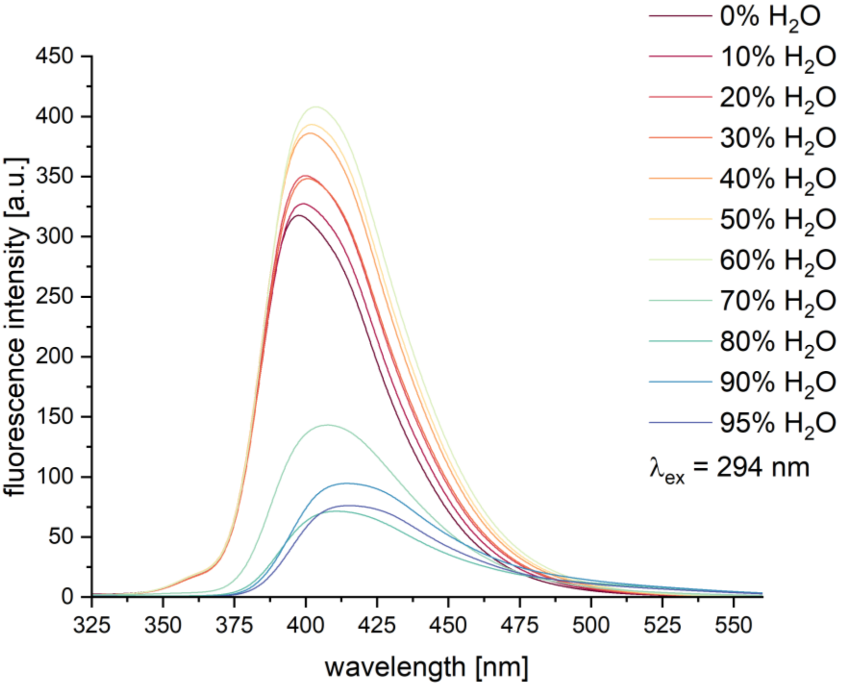

Fig. S29. Fluorescence spectra of compound **6** in H_2O/THF system containing different vol% of water in the sample (C = $2{\cdot}10^{-5}$ M, PMT voltage: 250 V).

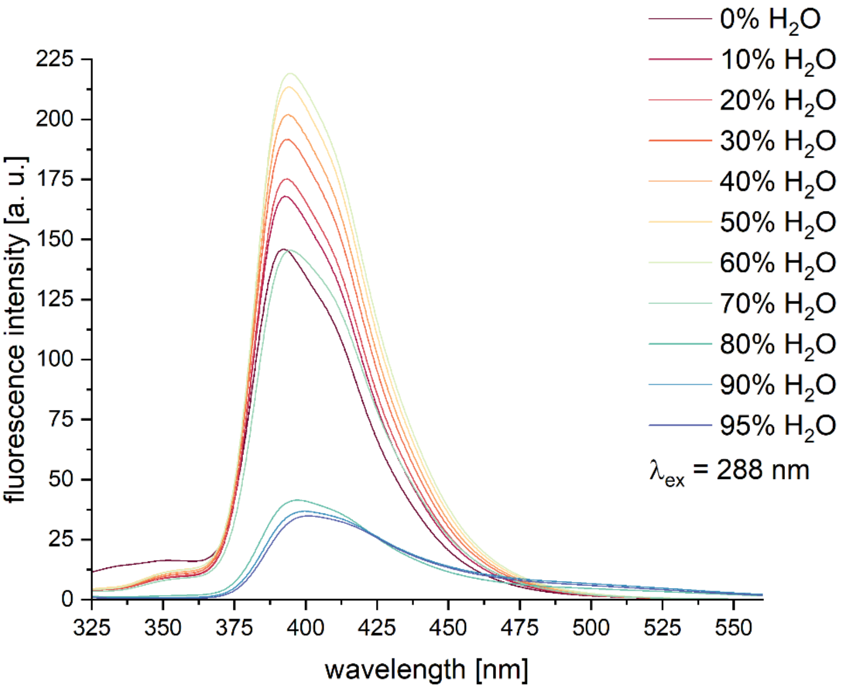

Fig. S30. Fluorescence spectra of compound **7** in H_2O/THF system containing different vol% of water in the sample (C = $2{\cdot}10^{-5}$ M, PMT voltage: 250 V).

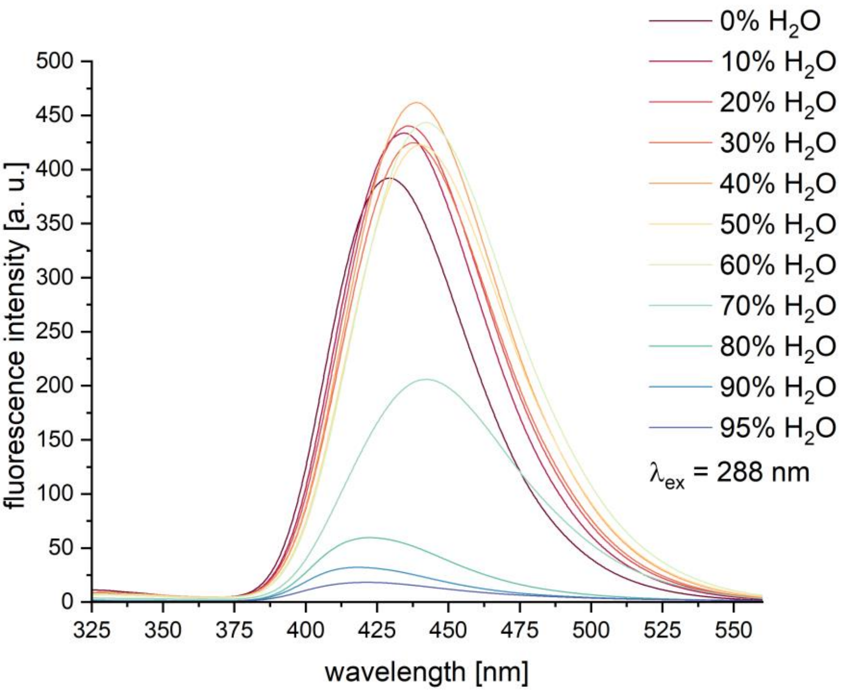

Fig. S31. Fluorescence spectra of compound **8** in H_2O/THF system containing different vol% of water in the sample (C = 2·10^{-5} M, PMT voltage: 250 V).

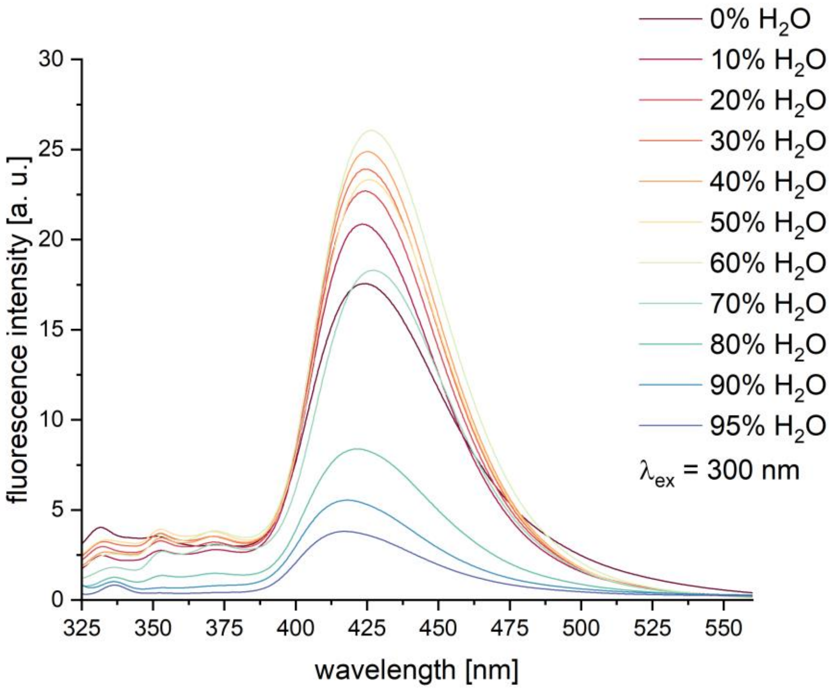

Fig. S32. Fluorescence spectra of compound **9** in H_2O/THF system containing different vol% of water in the sample (C = 2·10^{-5} M, PMT voltage: 250 V).

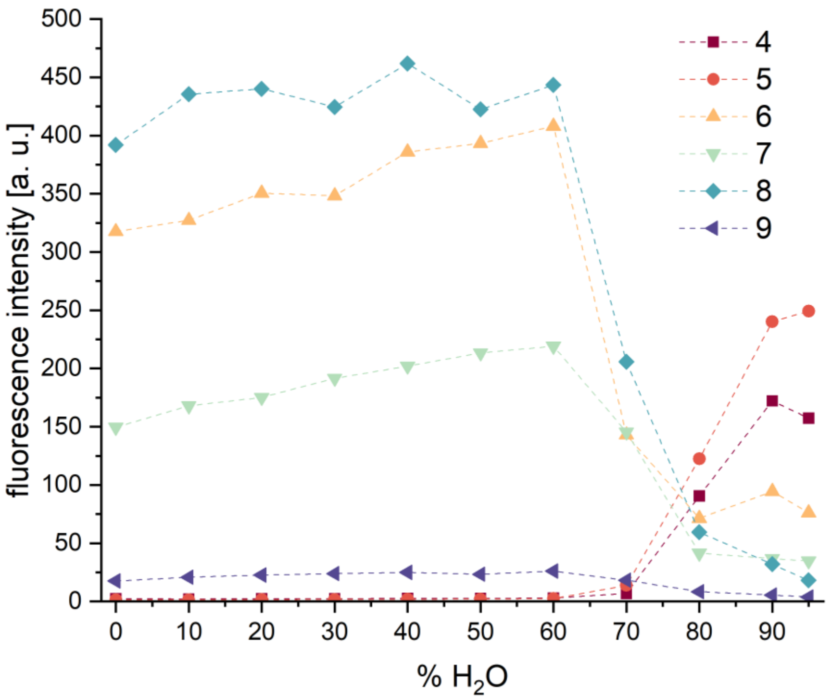

Fig. S33. Fluorescence intensity changes of compound **4-9** in H_2O/THF system containing different vol% of water in the sample (C = $2{\cdot}10^{-5}$ M; data for the maximum λ_{em}, PMT voltage: 250 V).

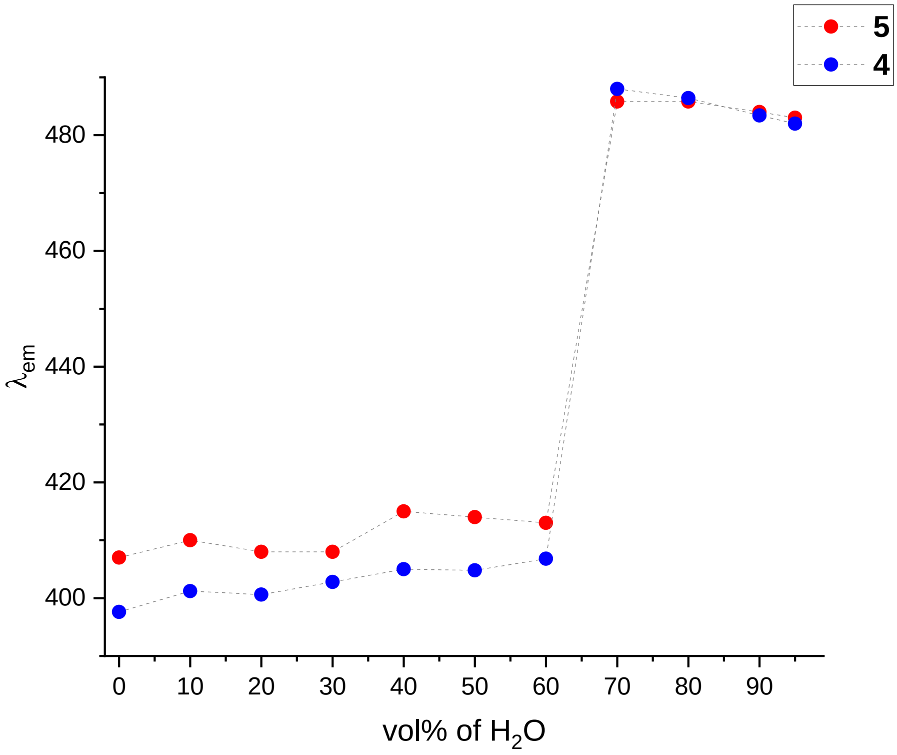

Fig. S34. The dependence of $\lambda_{em} = f(H_2O\ vol\%)$ for the AIE studies with **4-5**.

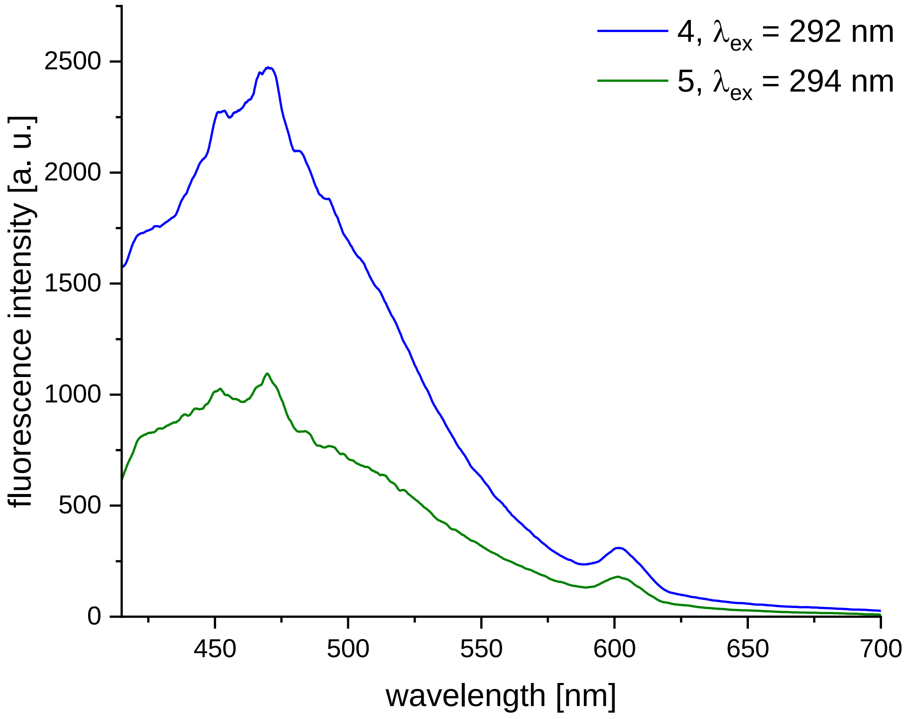

Fig. S35. Solid-state fluorescence spectra of **4-5**.

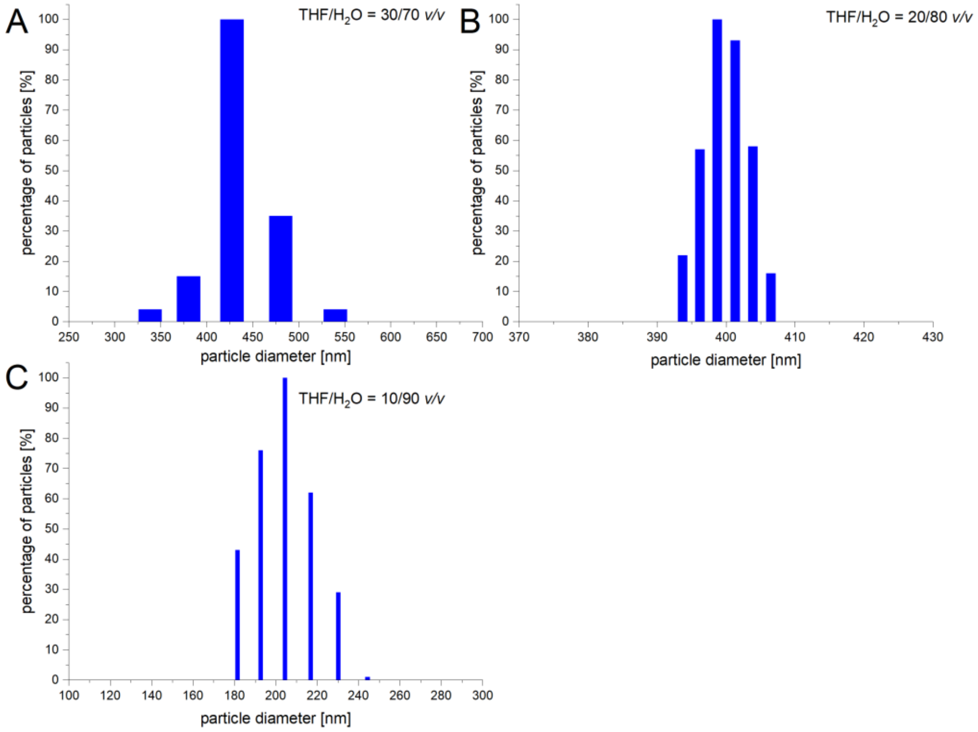

Fig. S36. Size distribution pattern of **4** in: A) H_2O/THF = 70/30 *v/v* system; B) H_2O/THF = 80/20 *v/v* system; C) H_2O/THF = 90/10 *v/v* system.

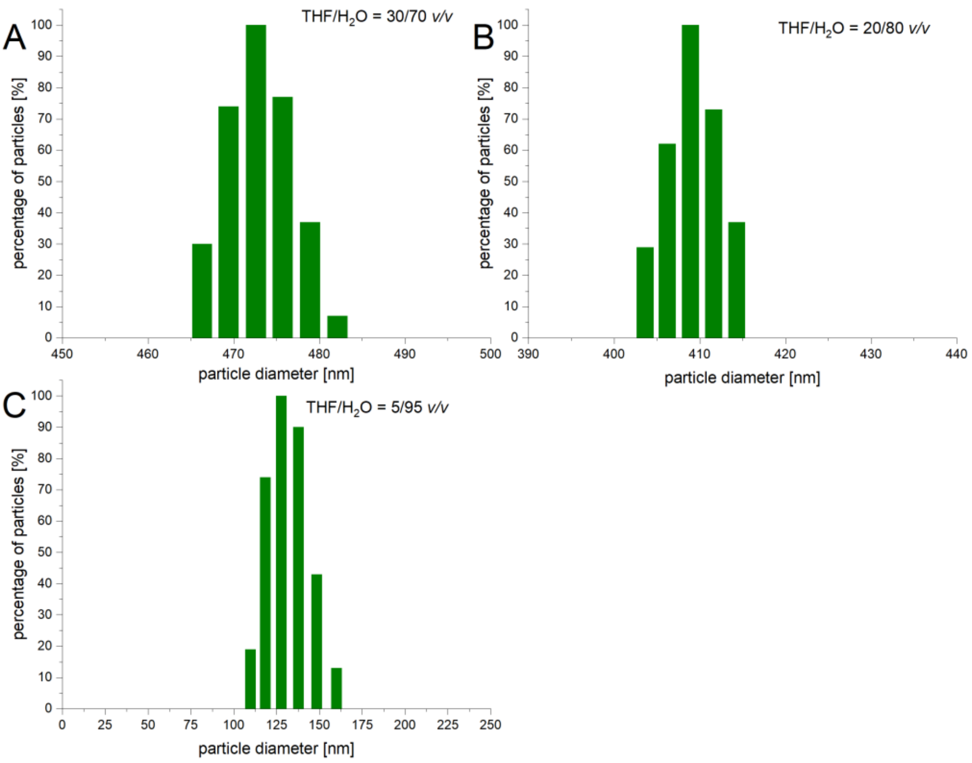

Fig. S37. Size distribution pattern of **5** in: A) H_2O/THF = 70/30 *v/v* system; B) H_2O/THF = 80/20 *v/v* system; C) H_2O/THF = 95/5 *v/v* system.

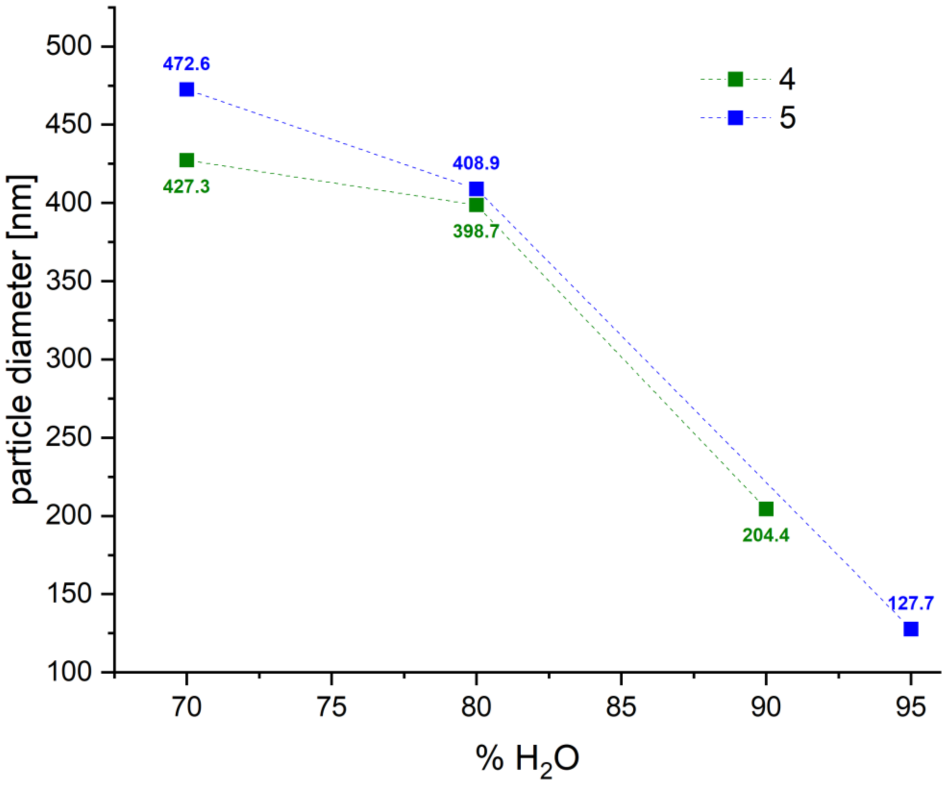

Fig. S38. Dependency of the particle size of compounds **4** and **5** on the water content (% vol) in the H_2O/THF system.

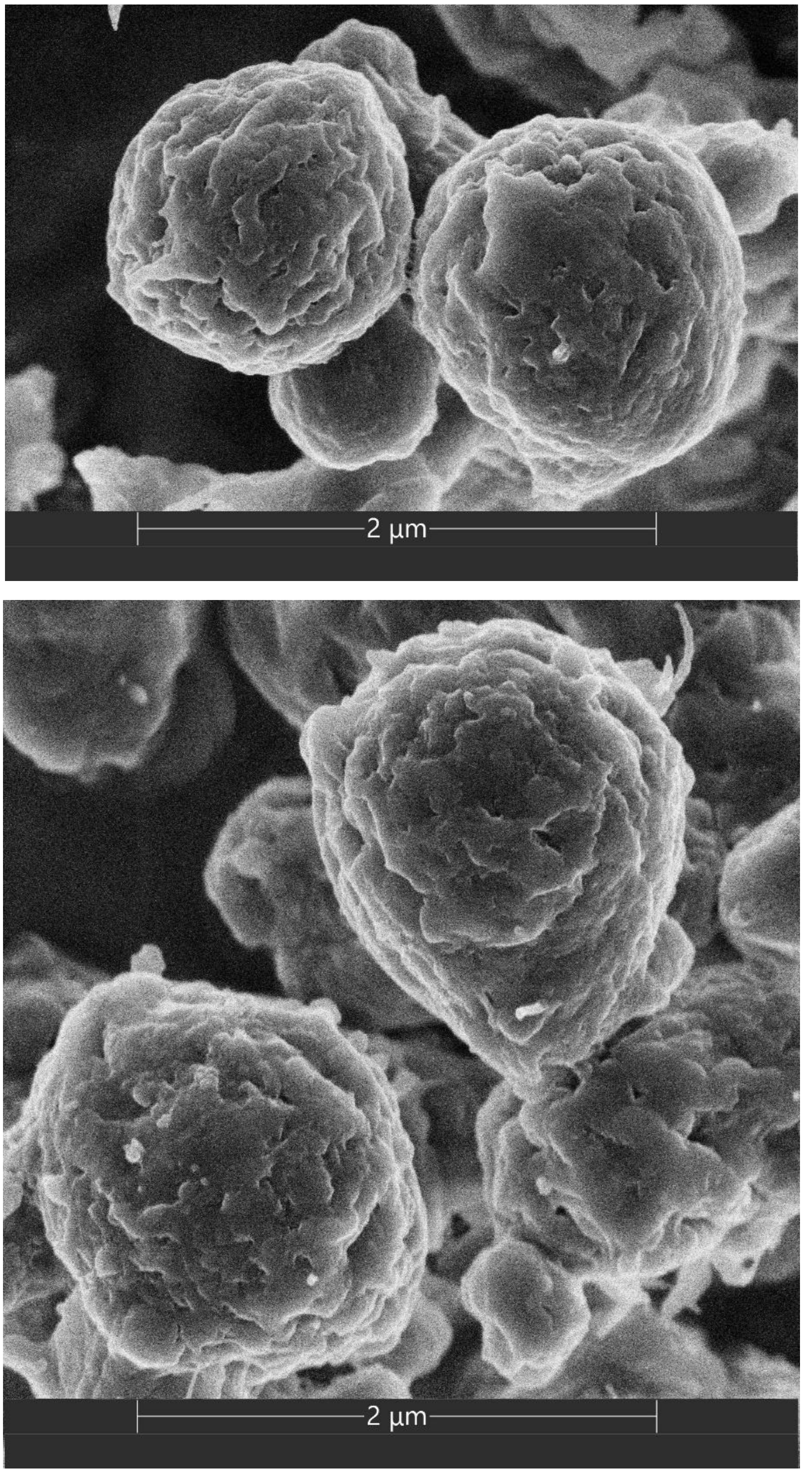

Fig. S39. SEM images of aggregated **4** (top) and **5** (bottom).

S5. Receptor studies

Stern-Volmer binding constant values (K_{sv}) were estimated using the double logarithmic Stern-Volmer method, given by the equation:

$$\log\left(\frac{I_0}{I} - 1\right) = \log(K_{sv}) + \log(\mathrm{C})$$

where I_0 and I are the fluorescence intensities of the receptor (compounds **4** or **5**) in the absence and presence of given cation, respectively, C is the concentration of a cation in solution. K_{sv} was taken as the value of $10^{\text{intercept}}$ of $\log\left(\frac{I_0}{I} - 1\right) = f\ (\log(C))$ linear plots.

The limit of detection (LOD) values were estimated from the linear plot of: $(I - I_{\min})/(I_{\max} - I_{\min}) = f\ (\log(C))$. At first, the *x* value for *y* =1 was calculated (value x(y=1)), and LOD was taken as $10^{\ x(y=1)}$.

The data for the estimation of K_{sv} for the studied systems were collected from emission maxima (for **4** and **5**: λ_{em} = 480 nm)

The stoichiometry of the complexes formed was estimated using Job's plot method, from the plot: (1-x)·ΔI = f (x). The x stands from the mole fraction of a cation. The expected stoichiometry was indicated by the maximum value in the plot.

For the interactions between **4** and Li^+, and **5** and Cs^+ non-linear (direct) data treatment using Bindfit (1:1 model) was also performed to estimate binding constant (K) values.[9–11]

All the spectra and plots are presented below.

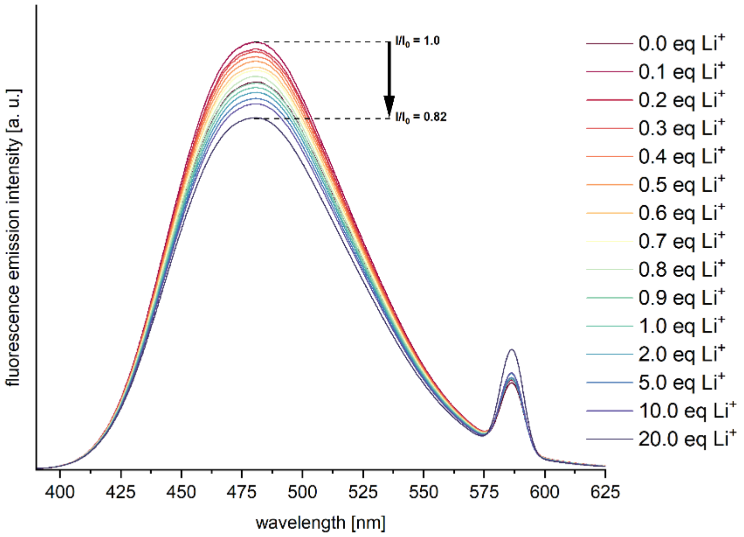

Fig. S40. Fluorescence spectra of aggregated **4** in the presence of various molar equivalents of Li^+ (λ_{ex} = 292 nm, C_4 = 2·10^{-5} M – aggregates, solvent = H_2O:THF = 95:5 v/v). The relative changes in the emission intensity are additionally graphically presented.

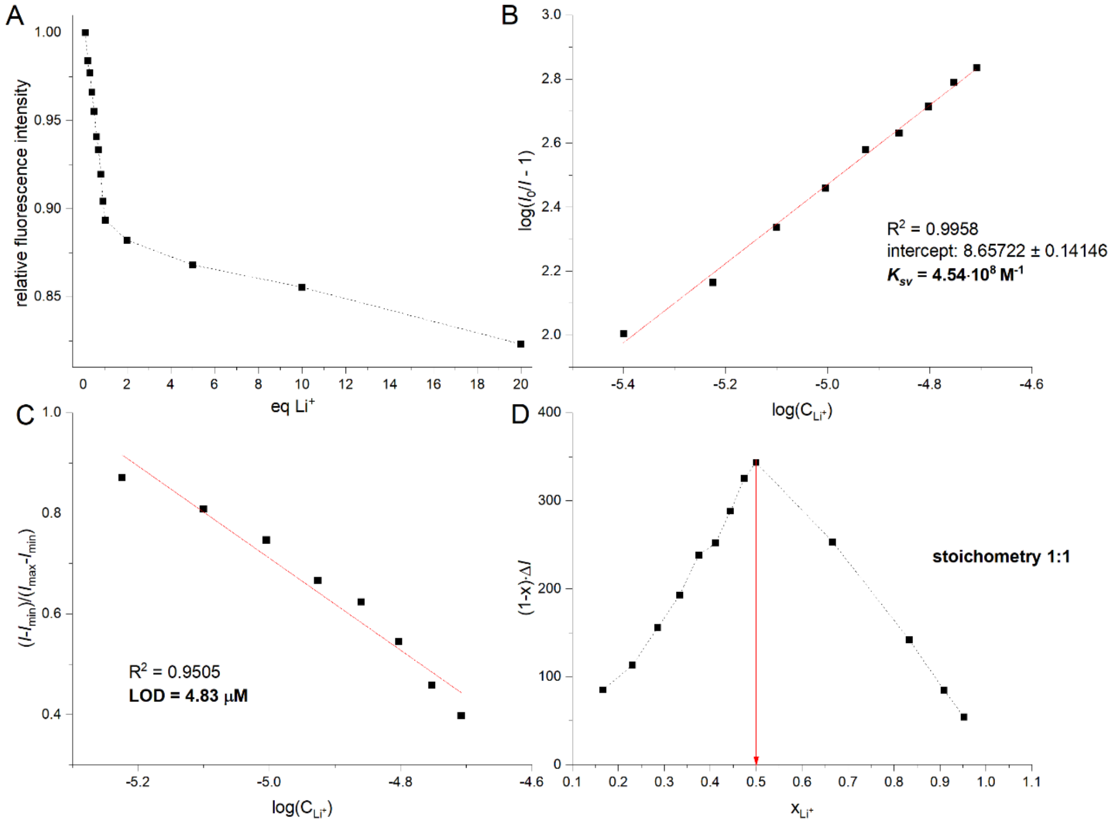

Fig. S41. Plots regarding interaction of **4** with Li^+: **A**) titration curve; **B**) Stern-Volmer plot; **C**) (I-I_{min})/(I_{max}-I_{min}) versus log(C_{cation}) plot; **D**) Job's plot (λ_{ex} = 292 nm, λ_{em} = 480 nm, C_4 = 2·10^{-5} M – aggregates, solvent = H_2O:THF = 95:5 v/v).

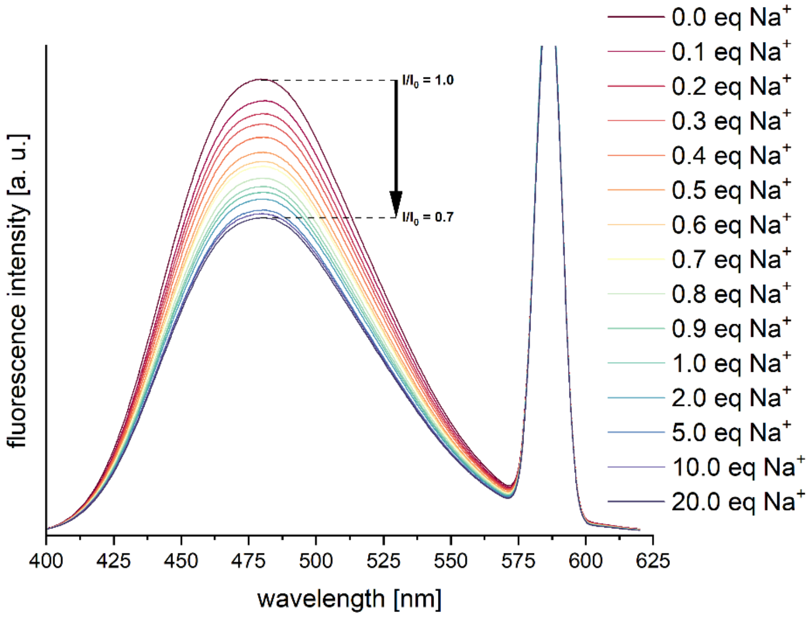

Fig. S42. Fluorescence spectra of aggregated **4** in the presence of various molar equivalents of Na^+ (λ_{ex} = 292 nm, C_4 = $2{\cdot}10^{-5}$ M – aggregates, solvent = H_2O:THF = 95:5 v/v). The relative changes in the emission intensity are additionally graphically presented.

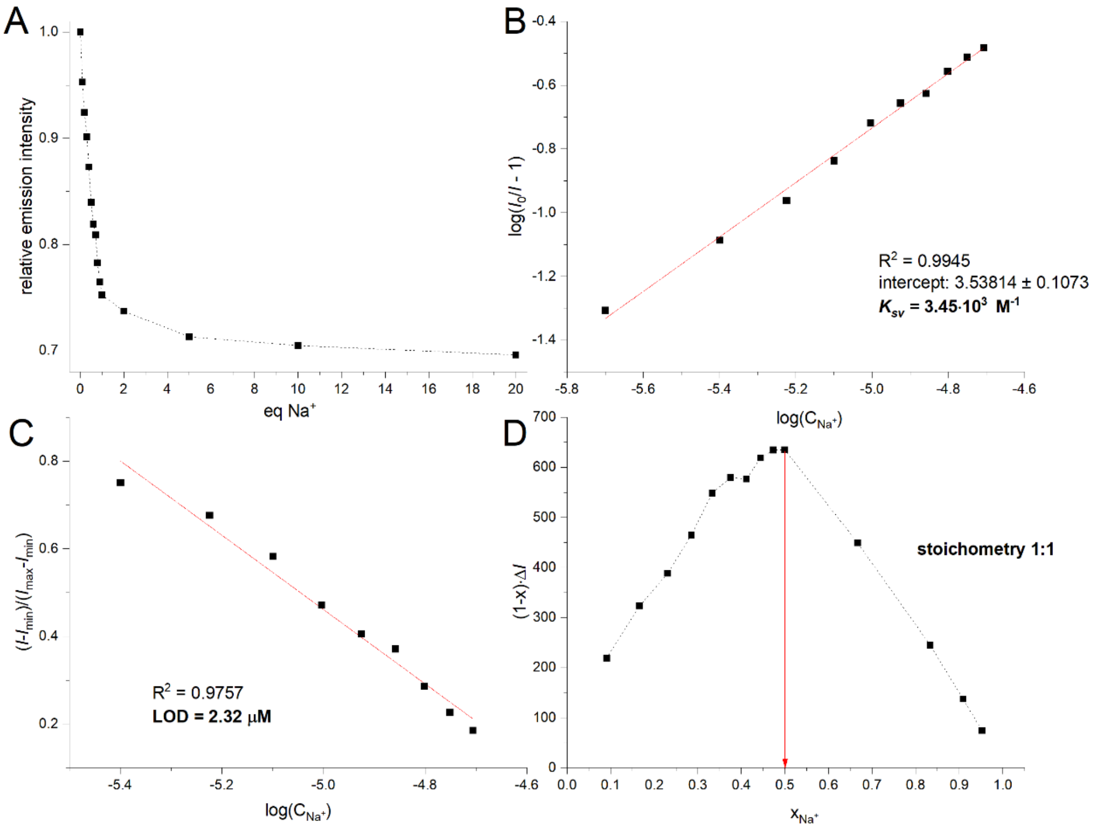

Fig. S43. Plots regarding interaction of **4** with Na^+: **A**) titration curve; **B**) Stern-Volmer plot; **C**) (I-I_{min})/(I_{max}-I_{min}) versus log(C_{cation}) plot; **D**) Job's plot (λ_{ex} = 292nm, λ_{em} = 480 nm, C_4 = $2{\cdot}10^{-5}$ M – aggregates, solvent = H_2O:THF = 95:5 v/v).

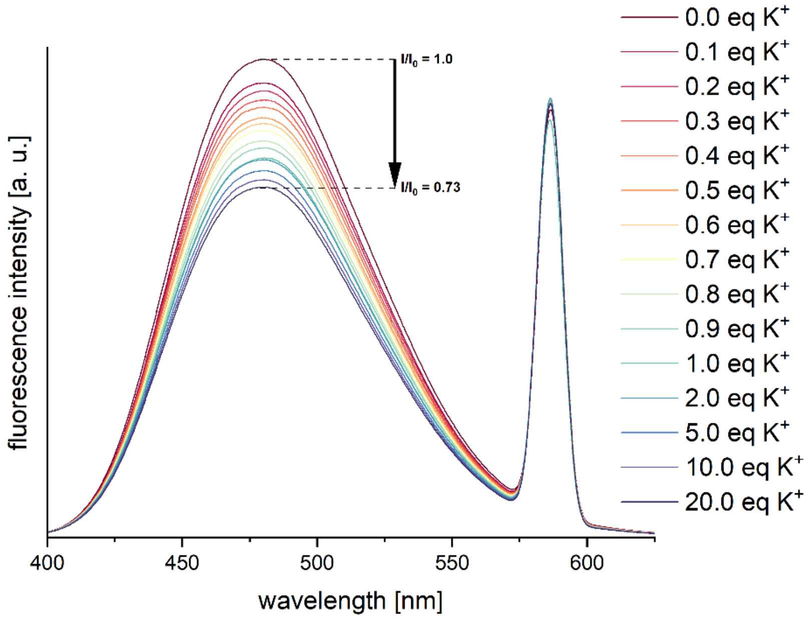

Fig. S44. Fluorescence spectra of aggregated **4** in the presence of various molar equivalents of K^+ (λ_{ex} = 292 nm, C_4 = 2·10^{-5} M – aggregates, solvent = H_2O:THF = 95:5 v/v). The relative changes in the emission intensity are additionally graphically presented.

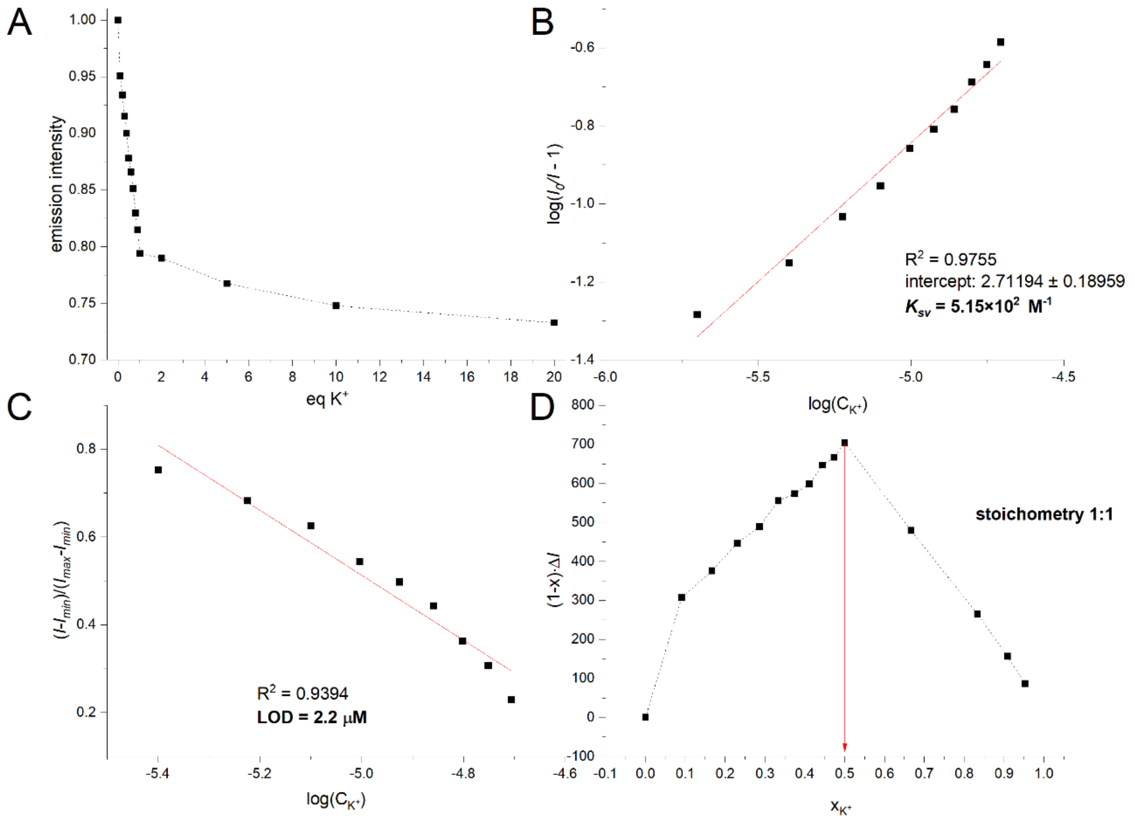

Fig. S45. Plots regarding interaction of **4** with K^+: **A**) titration curve; **B**) Stern-Volmer plot; **C**) (I-I_{min})/(I_{max}-I_{min}) versus log(C_{cation}) plot; **D**) Job's plot (λ_{ex} = 292 nm, λ_{em} = 480 nm, C_4 = 2·10^{-5} M – aggregates, solvent = H_2O:THF = 95:5 v/v).

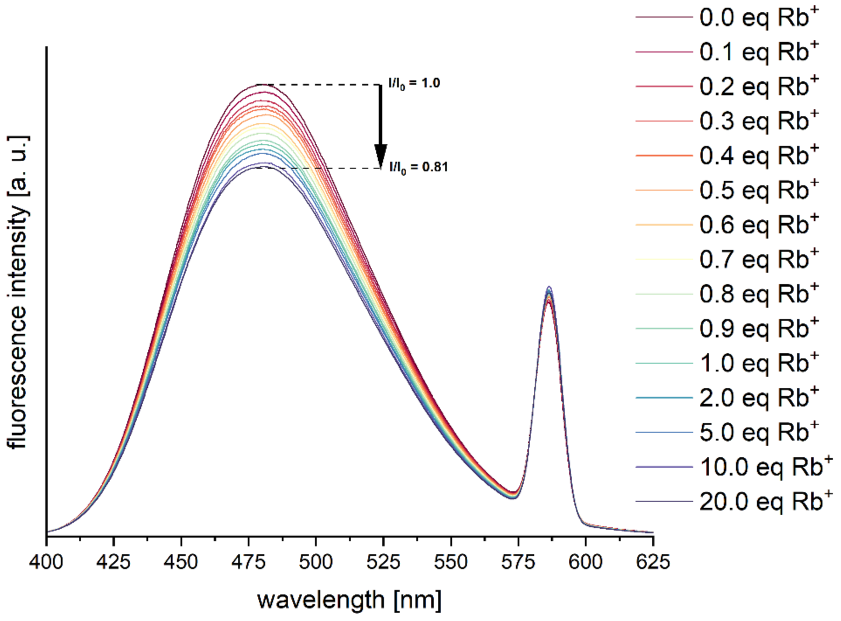

Fig. S46. Fluorescence spectra of aggregated **4** in the presence of various molar equivalents of Rb^+ (λ_{ex} = 292 nm, C_4 = 2·10^{-5} M – aggregates, solvent = H_2O:THF = 95:5 v/v). The relative changes in the emission intensity are additionally graphically presented.

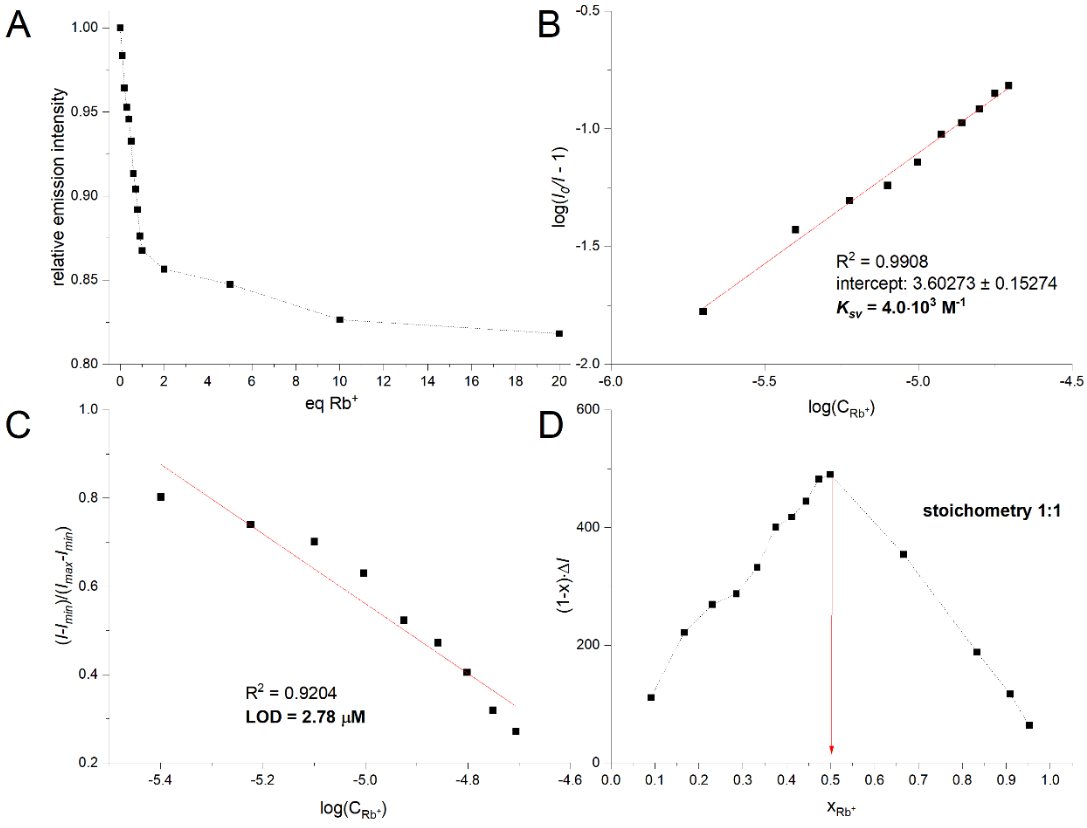

Fig. S47. Plots regarding interaction of **4** with Rb^+: **A**) titration curve; **B**) Stern-Volmer plot; **C**) $(I-I_{min})/(I_{max}-I_{min})$ versus log(C_{cation}) plot; **D**) Job's plot (λ_{ex} = 292 nm, λ_{em} = 480 nm, C_4 = 2·10^{-5} M – aggregates, solvent = H_2O:THF = 95:5 v/v).

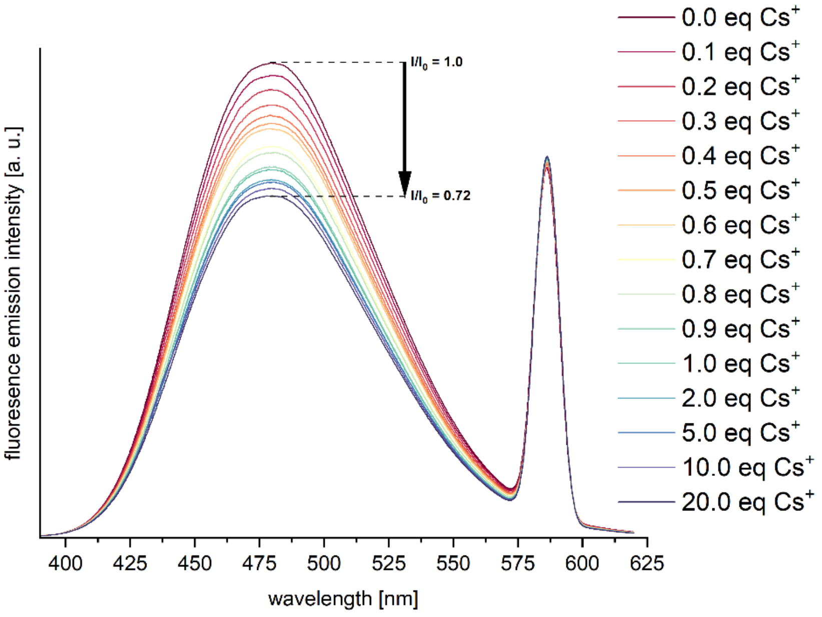

Fig. S48. Fluorescence spectra of aggregated **4** in the presence of various molar equivalents of Cs^+ (λ_{ex} = 292 nm, $C_4 = 2{\cdot}10^{-5}$ M – aggregates, solvent = H_2O:THF = 95:5 v/v). The relative changes in the emission intensity are additionally graphically presented.

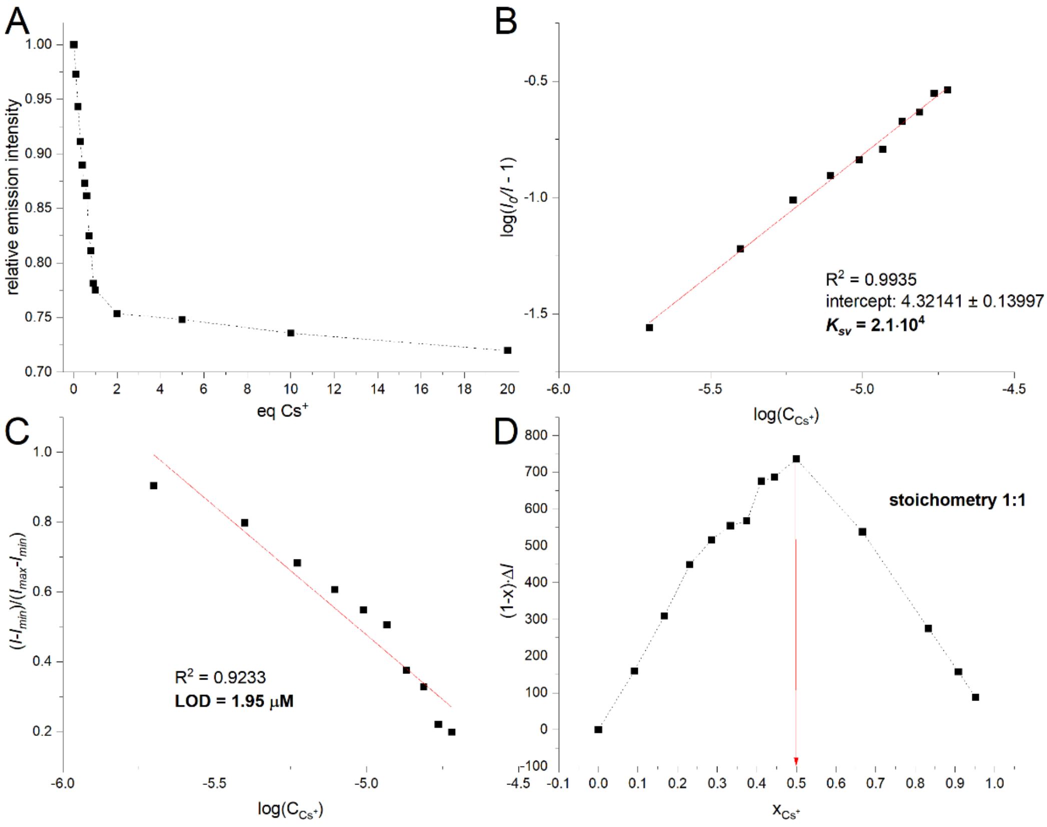

Fig. S49. Plots regarding interaction of **4** with Cs^+: **A**) titration curve; **B**) Stern-Volmer plot; **C**) $(I-I_{min})/(I_{max}-I_{min})$ versus log(C_{cation}) plot; **D**) Job's plot (λ_{ex} = 292 nm, λ_{em} = 480 nm, $C_4 = 2{\cdot}10^{-5}$ M – aggregates, solvent = H_2O:THF = 95:5 v/v).

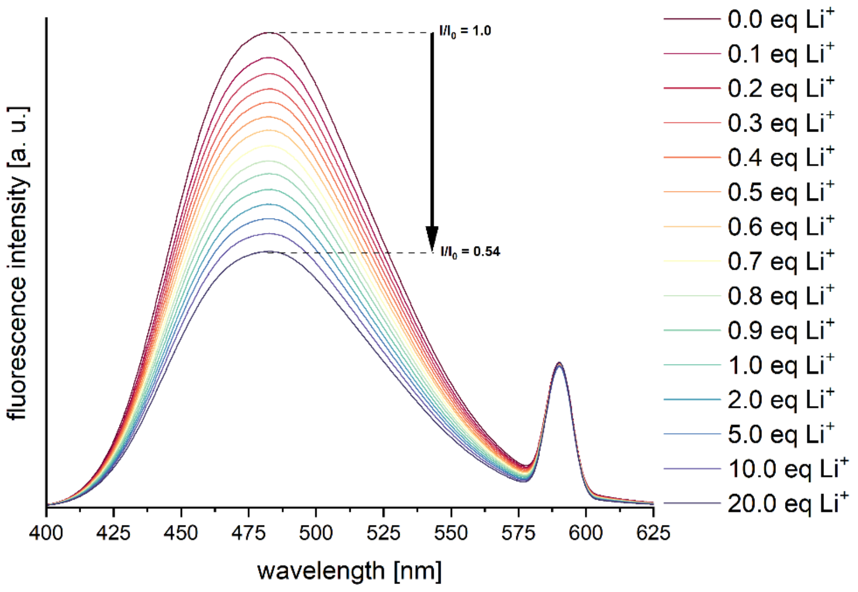

Fig. S50. Fluorescence spectra of aggregated **5** in the presence of various molar equivalents of Li^+ (λ_{ex} = 294 nm, $C_5 = 2{\cdot}10^{-5}$ M – aggregates, solvent = H_2O:THF = 95:5 v/v). The relative changes in the emission intensity are additionally graphically presented.

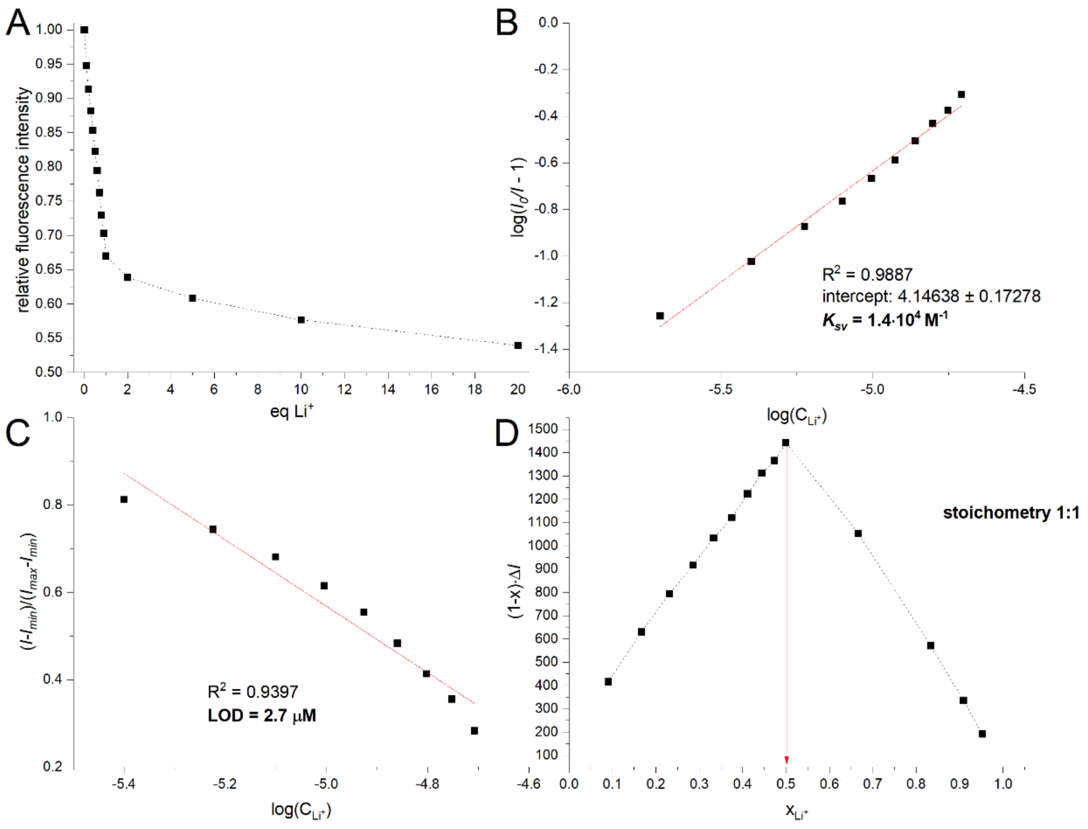

Fig. S51. Plots regarding interaction of **5** with Li^+: **A**) titration curve; **B**) Stern-Volmer plot; **C**) $(I-I_{min})/(I_{max}-I_{min})$ versus log(C_{cation}) plot; **D**) Job's plot (λ_{ex} = 294 nm, λ_{em} = 490 nm, $C_5 = 2{\cdot}10^{-5}$ M – aggregates, solvent = H_2O:THF = 95:5 v/v).

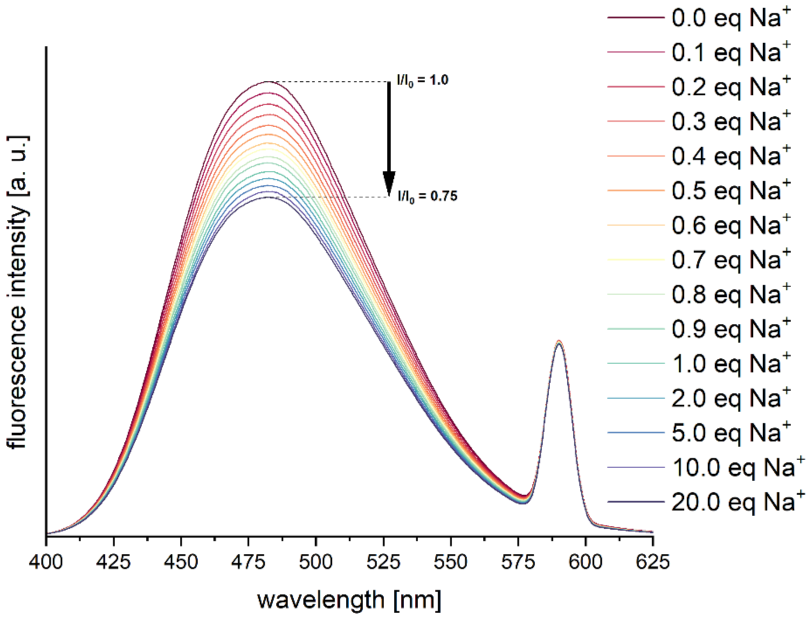

Fig. S52. Fluorescence spectra of aggregated **5** in the presence of various molar equivalents of Na^+ (λ_{ex} = 294 nm, $C_5 = 2{\cdot}10^{-5}$ M – aggregates, solvent = H_2O:THF = 95:5 v/v). The relative changes in the emission intensity are additionally graphically presented.

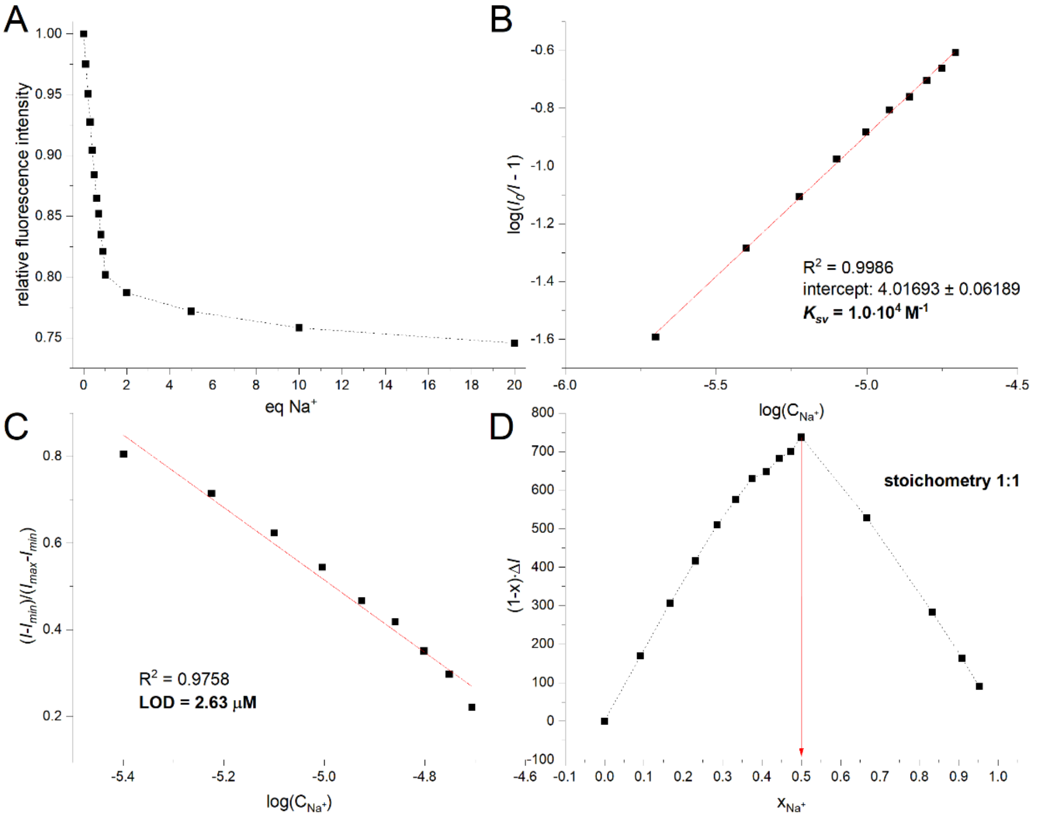

Fig. S53. Plots regarding interaction of **5** with Na^+: **A**) titration curve; **B**) Stern-Volmer plot; **C**) $(I\text{-}I_{min})/(I_{max}\text{-}I_{min})$ versus log(C_{cation}) plot; **D**) Job's plot (λ_{ex} = 294 nm, λ_{em} = 490 nm, $C_5 = 2{\cdot}10^{-5}$ M – aggregates, solvent = H_2O:THF = 95:5 v/v).

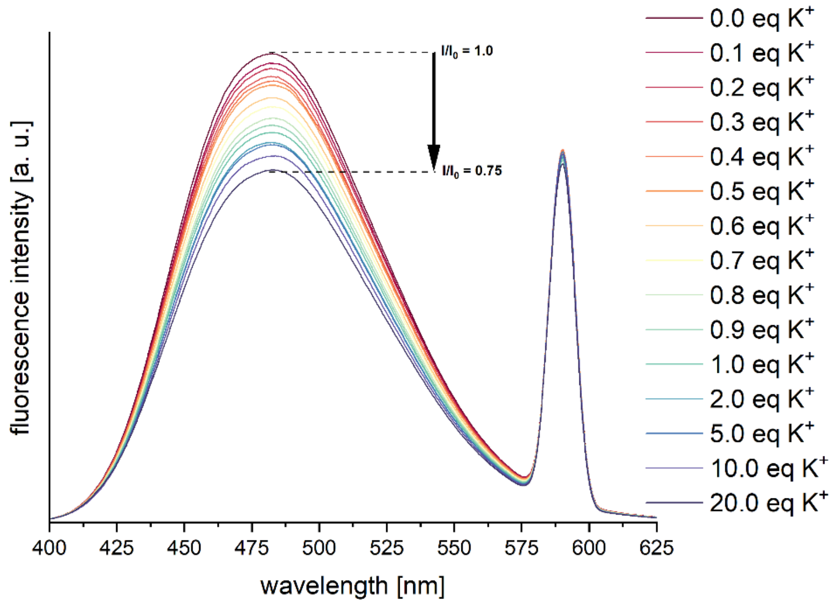

Fig. S54. Fluorescence spectra of aggregated **5** in the presence of various molar equivalents of K^+ (λ_{ex} = 294 nm, $C_5 = 2{\cdot}10^{-5}$ M – aggregates, solvent = H_2O:THF = 95:5 v/v). The relative changes in the emission intensity are additionally graphically presented.

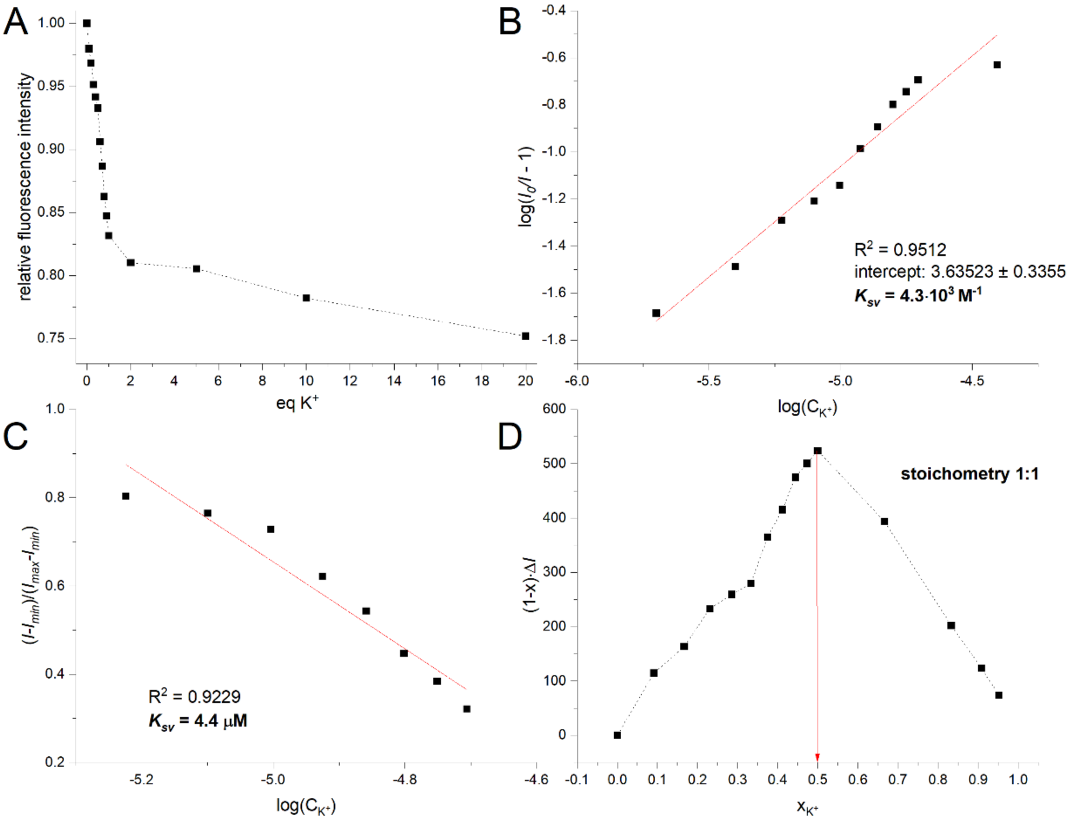

Fig. S55. Plots regarding interaction of **5** with K^+: **A**) titration curve; **B**) Stern-Volmer plot; **C**) $(I-I_{min})/(I_{max}-I_{min})$ versus log(C_{cation}) plot; **D**) Job's plot (λ_{ex} = 294 nm, λ_{em} = 490 nm, $C_5 = 2{\cdot}10^{-5}$ M – aggregates, solvent = H_2O:THF = 95:5 v/v).

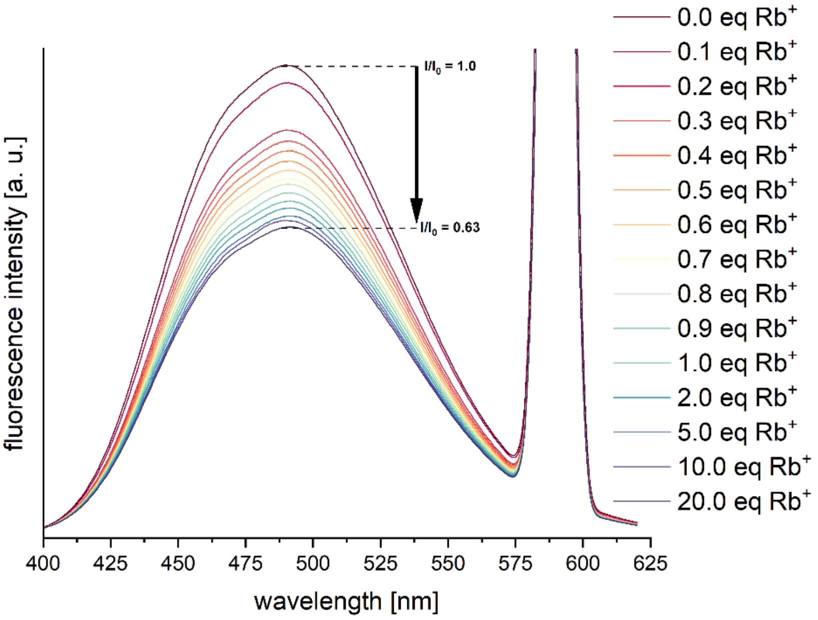

Fig. S56. Fluorescence spectra of aggregated **5** in the presence of various molar equivalents of Rb^+ (λ_{ex} = 294 nm, $C_5 = 2{\cdot}10^{-5}$ M – aggregates, solvent = H_2O:THF = 95:5 v/v). The relative changes in the emission intensity are additionally graphically presented.

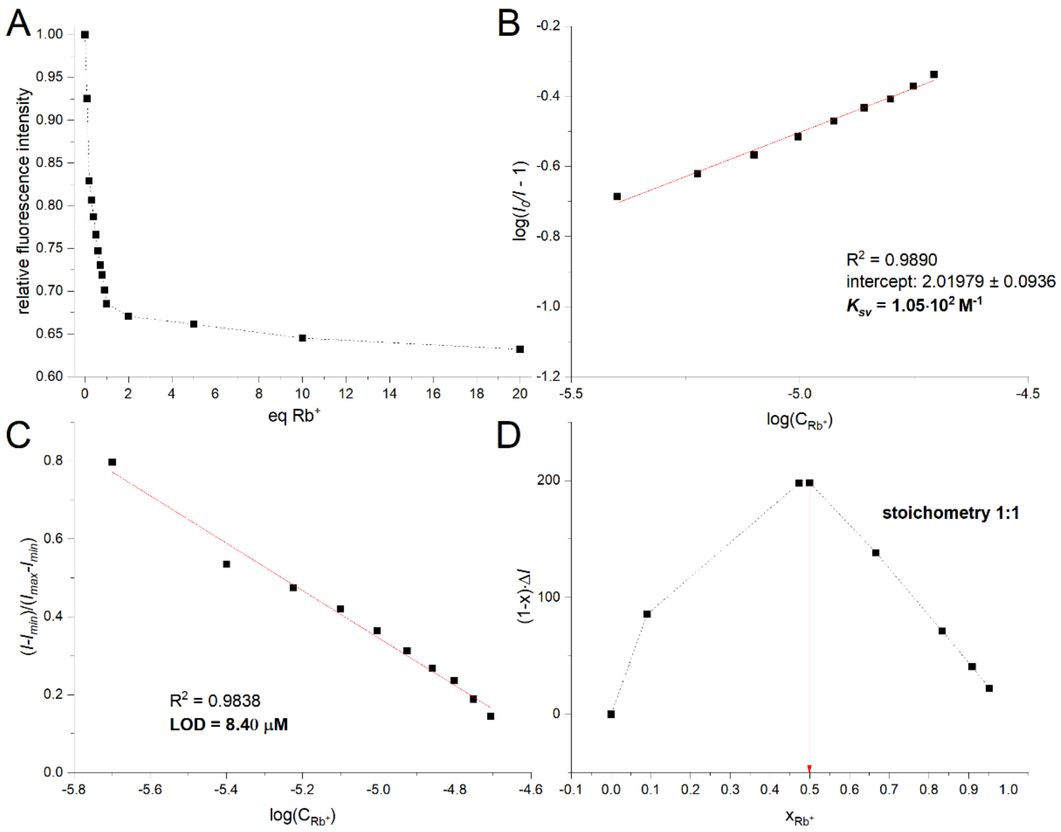

Fig. S57. Plots regarding interaction of **5** with Rb^+: **A**) titration curve; **B**) Stern-Volmer plot; **C**) $(I-I_{min})/(I_{max}-I_{min})$ versus log(C_{cation}) plot; **D**) Job's plot (λ_{ex} = 294 nm, λ_{em} = 490 nm, $C_5 = 2{\cdot}10^{-5}$ M – aggregates, solvent = H_2O:THF = 95:5 v/v).

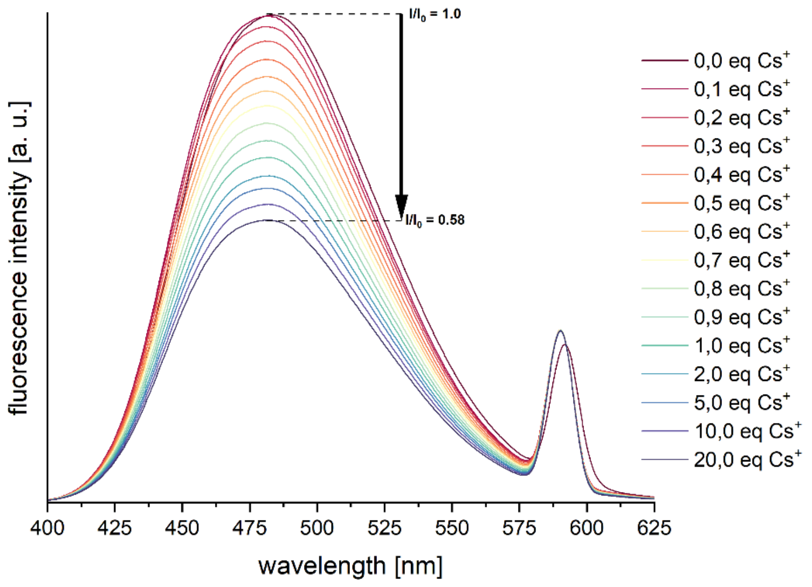

Fig. S58. Fluorescence spectra of aggregated **5** in the presence of various molar equivalents of Cs^+ (λ_{ex} = 294 nm, C_5 = 2·10^{-5} M – aggregates, solvent = H_2O:THF = 95:5 v/v). The relative changes in the emission intensity are additionally graphically presented.

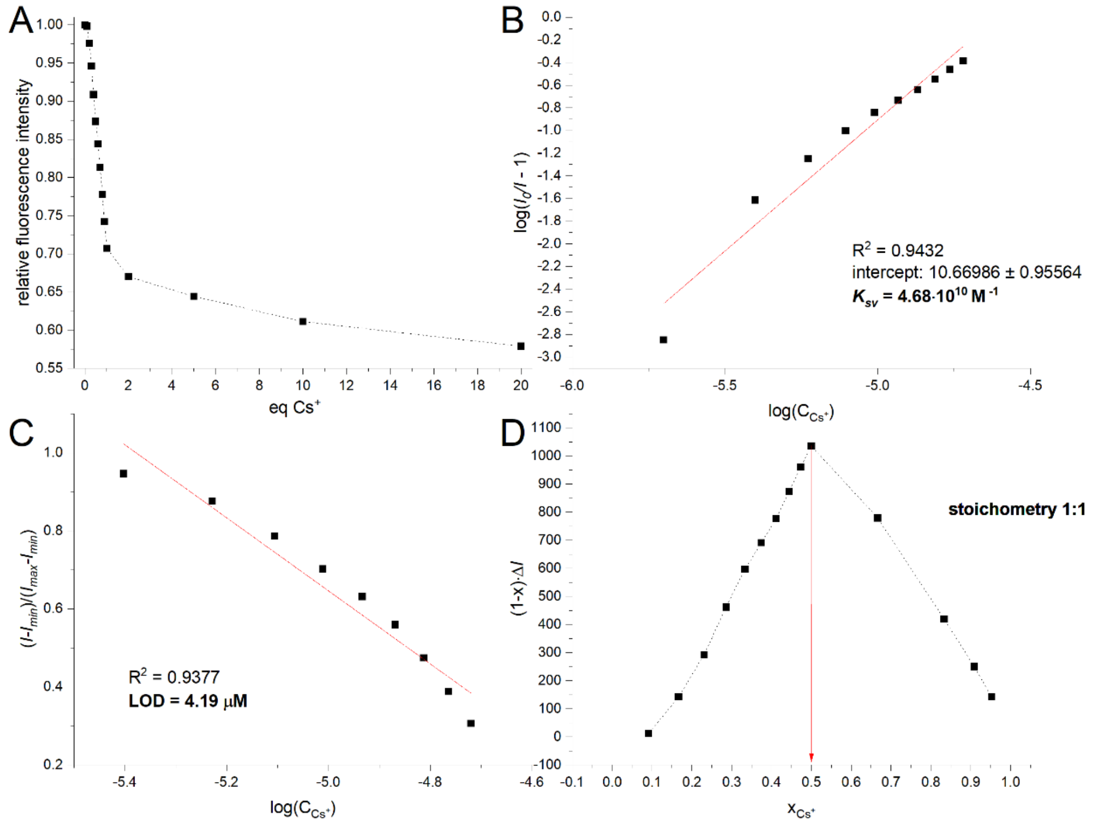

Fig. S59. Plots regarding interaction of **5** with Cs^+: **A**) titration curve; **B**) Stern-Volmer plot; **C**) (I-I_{min})/(I_{max}-I_{min}) versus log(C_{cation}) plot; **D**) Job's plot (λ_{ex} = 294 nm, λ_{em} = 490 nm, C_5 = 2·10^{-5} M – aggregates, solvent = H_2O:THF = 95:5 v/v).

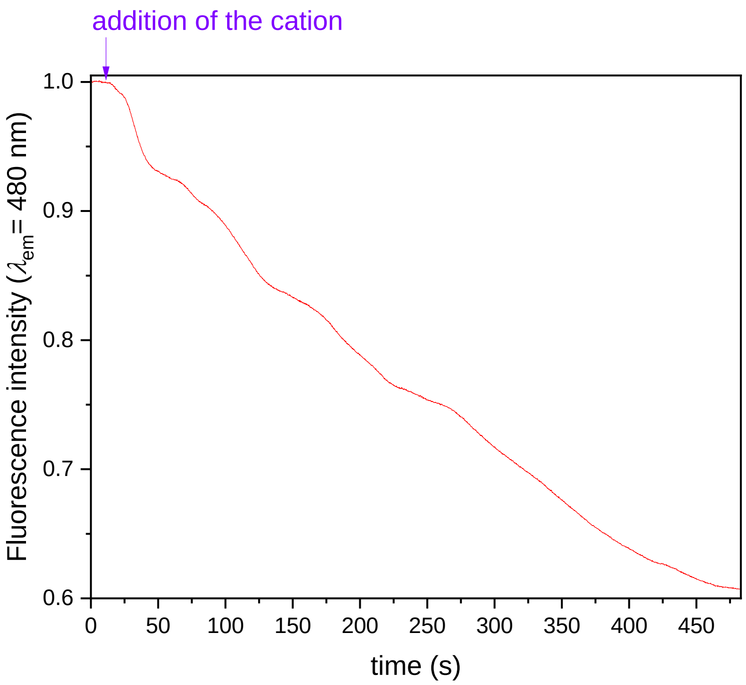

Fig. S60. Changes in the relative fluorescence intensity of aggregated **4** in the presence of 1 equiv. of Li^+ (λ_{ex} = 292 nm, λ_{em} = 480 nm, C_4 = $2 \cdot 10^{-5}$ M, solvent = H_2O:THF = 95:5 v/v).

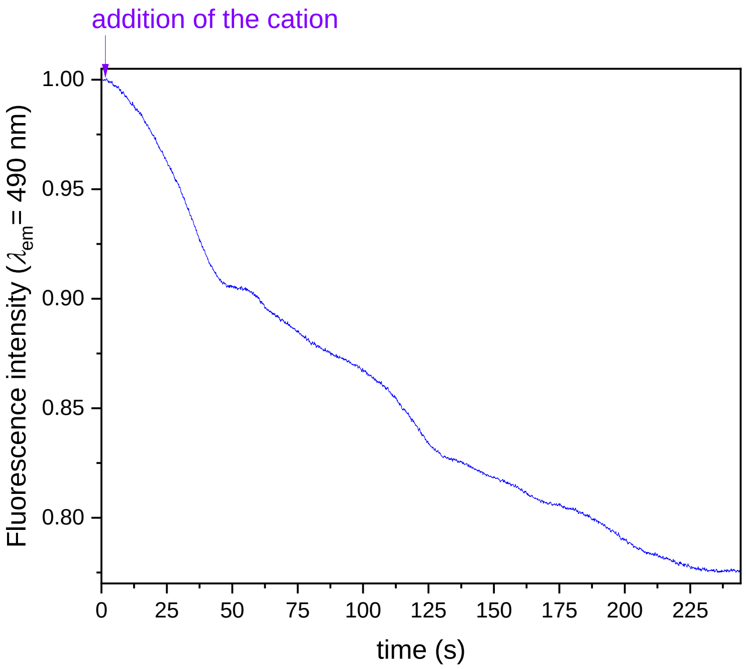

Fig. S61. Changes in the relative fluorescence intensity of aggregated **5** in the presence of 1 equiv. of Cs^+ (λ_{ex} = 294 nm, λ_{em} = 490 nm, C_4 = $2 \cdot 10^{-5}$ M, solvent = H_2O:THF = 95:5 v/v).

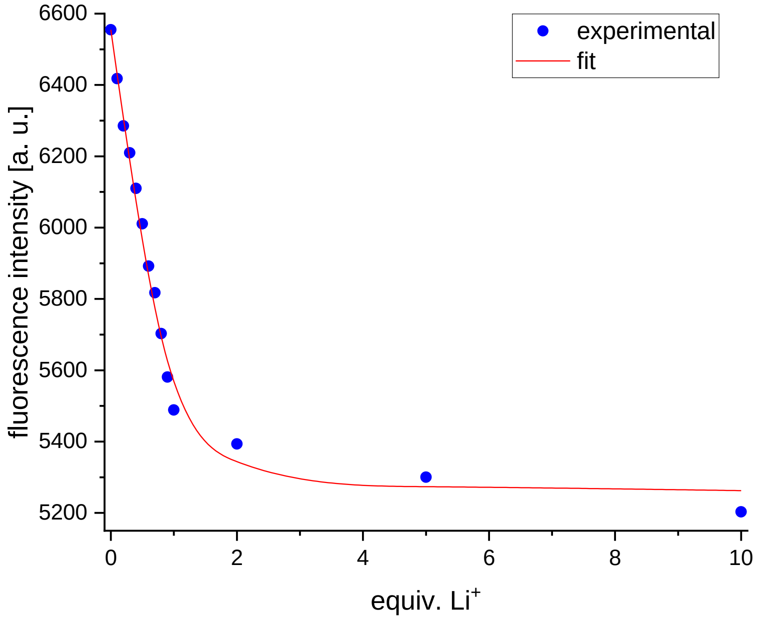

Fig. S62. Global fitting to the 1:1 model (Bindfit) regarding the interactions between **4** and Li^{+}. K = 5.52·10^{5} M^{-1} (±30%), covariance: 9.1·10^{-3}, Nelder-Mead method-algorithm.

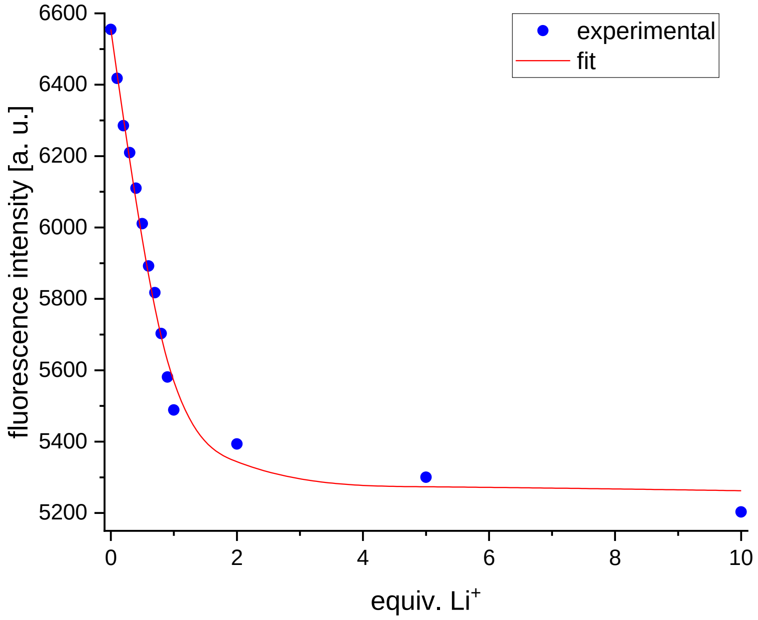

Fig. S63. Global fitting to the 1:1 model (Bindfit) regarding the interactions between **5** and Cs^{+}. K = 3.47·10^{5} M^{-1} (±38%), covariance: 1.7·10^{-2}, Nelder-Mead method-algorithm.

Table S3. Bindfit analyzes (Nelder-Mead method-algorithm) of the interactions between **4** and Li^+ from fluorescence titrations. Curve for global fitting to the 1:1 model (the best and most reliable fit) is presented in Fig. S52.

Stoichiom.	Mode	$K_{1:1}$	$K_{1:1}$ error (%)	$K_{1:2}$	$K_{1:2}$ error (%)	Covariance
**1:1**	**n.a.**	**$5.52·10^5$**	**30**	**n.a.**	**n.a.**	**$9.1·10^{-3}$**
1:2	n.a.	unreliable[a]	unreliable[a]	$1.90·10^4$	43	n.a.[b]
	non-cooperative	unreliable[a]	unreliable[a]	n.a.	n.a.	n.a.[b]
	additive	unreliable[a]	unreliable[a]	$2.10·10^3$	30	n.a.[b]
	statistical	unreliable[a]	unreliable[a]	n.a.	n.a.	n.a.[b]
2:1	n.a.	fit failed	fit failed	fit failed	fit failed	fit failed
	non-cooperative	$3.70·10^4$	10	n.a.	n.a.	$1.1·10^{-2}$
	additive	$4.48·10^5$	65	unreliable[a]	unreliable[a]	n.a.[b]
	statistical	$6.72·10^4$	15	n.a.	n.a.	$3.0·10^{-2}$

[a] Result labeled "unreliable" means very high *K* error value (>100-200%) or negative *K* (or *K* error) value, what is impossible. [b] Covariance value not given due to unreliable *K* and/or *K* error estimation as noted in point [a].

Table S4. Bindfit analyzes (Nelder-Mead method-algorithm) of the interactions between **5** and Cs^+ from fluorescence titrations. Curve for global fitting to the 1:1 model (the best and most reliable fit) is presented in Fig. S53.

Stoichiom.	Mode	$K_{1:1}$	$K_{1:1}$ error (%)	$K_{1:2}$	$K_{1:2}$ error (%)	Covariance
**1:1**	**n.a.**	**$3.47·10^5$**	**38**	**n.a.**	**n.a.**	**$1.7·10^{-2}$**
1:2	n.a.	unreliable[a]	unreliable[a]	unreliable[a]	unreliable[a]	n.a.[b]
	non-cooperative	unreliable[a]	unreliable[a]	n.a.	n.a.	n.a.[b]
	additive	unreliable[a]	unreliable[a]	$3.78·10^3$	45	n.a.[b]
	statistical	unreliable[a]	unreliable[a]	n.a.	n.a.	n.a.[b]
2:1	n.a.	fit failed	fit failed	fit failed	fit failed	fit failed
	non-cooperative	$2.56·10^4$	10	n.a.	n.a.	$3.7·10^{-2}$
	additive	$1.33·10^6$	55	unreliable[a]	unreliable[a]	n.a.[b]
	statistical	$6.02·10^4$	18	n.a.	n.a.	$4.0·10^{-2}$

[a] Result labeled "unreliable" means very high *K* error value (>100-200%) or negative *K* (or *K* error) value, what is impossible. [b] Covariance value not given due to unreliable *K* and/or *K* error estimation as noted in point [a].

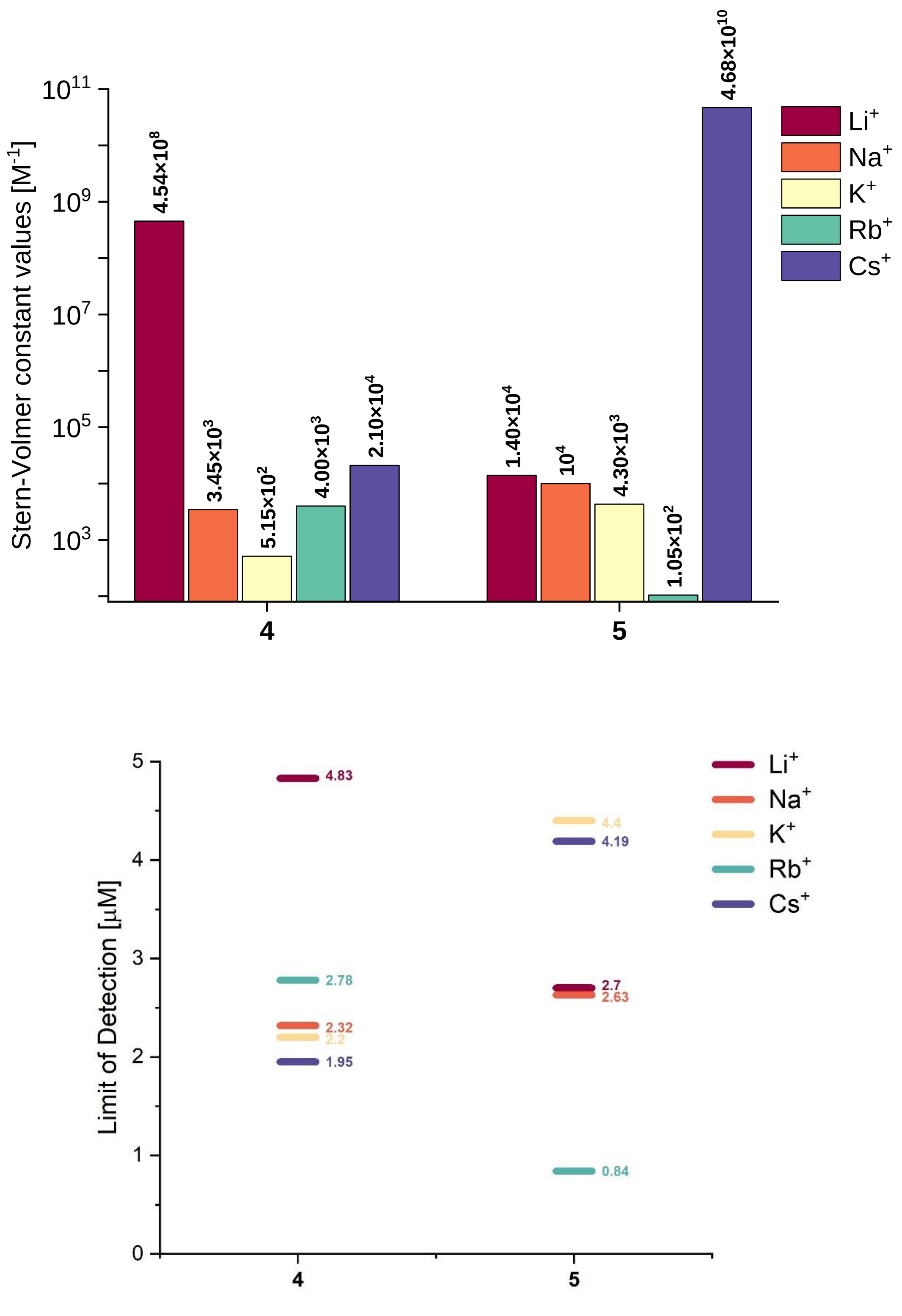

Fig. S64. Comparison of the *K*sv (top) and LOD (bottom) values for the interactions between **4** or **5** and tested metal cations.

Table S5. Bindfit analyzes (Nelder-Mead method-algorithm) for the additional studies on the stoichiometry of dynamically formed **4**-Li^+ complexes based on titrations with different host (**4**) concentrations (aggregates; solvent = H_2O:THF = 95:5 *v*/*v*). Global fitting curves to the 1:1 model (the best and most reliable fit) is presented on the left for respective titrations.

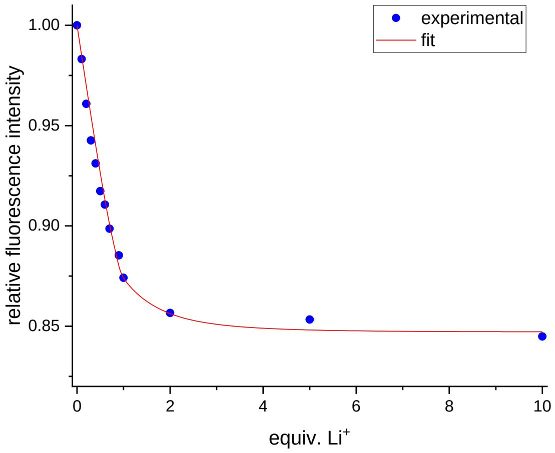

**4**-Li^+ (C_4 = 2·10^{-6} M)		
Stoichiom.	Mode	Covariance
**1:1**	**n.a.**	**1.5·10^{-2}**
1:2	n.a.	n.a.[a]
	non-cooperative	n.a.[a]
	additive	n.a.[a]
	statistical	n.a.[a]
2:1	n.a.	fit failed
	non-cooperative	3.2·10^{-1}
	additive	6.1·10^{-2}
	statistical	5.9·10^{-2}

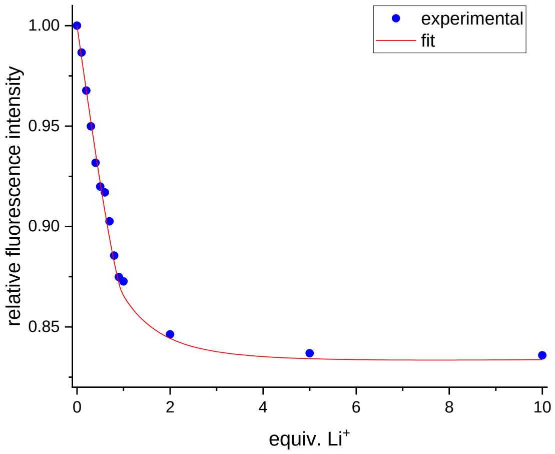

**4**-Li^+ (C_4 = 5·10^{-5} M)		
Stoichiom.	Mode	Covariance
**1:1**	**n.a.**	**7.0·10^{-3}**
1:2	n.a.	fit failed
	non-cooperative	n.a.[a]
	additive	5.7·10^{-2}
	statistical	n.a.[a]
2:1	n.a.	n.a.[a]
	non-cooperative	1.5·10^{-2}
	additive	n.a.[a]
	statistical	1.6·10^{-2}

[a] Covariance value not given due to very high *K* error value (>100-200%) or negative *K* (or *K* error) value, what is impossible.

Table S6. Bindfit analyzes (Nelder-Mead method-algorithm) for the additional studies on the stoichiometry of dynamically formed **5**-Cs^+ complexes based on titrations with different host (**5**) concentrations (aggregates; solvent = H_2O:THF = 95:5 *v*/*v*). Global fitting curves to the 1:1 model (the best and most reliable fit) is presented on the left for respective titrations.

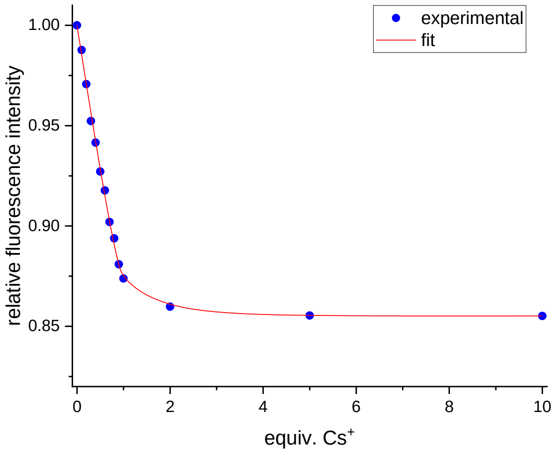

**5**-Cs^+ (C_5 = $2\cdot10^{-6}$ M)		
Stoichiom.	Mode	Covariance
**1:1**	**n.a.**	**$4.7\cdot10^{-3}$**
1:2	n.a.	fit failed
	non-cooperative	fit failed
	additive	n.a.[a]
	statistical	n.a.[a]
2:1	n.a.	fit failed
	non-cooperative	$3.7\cdot10^{-1}$
	additive	$2.8\cdot10^{-2}$
	statistical	$2.3\cdot10^{-2}$

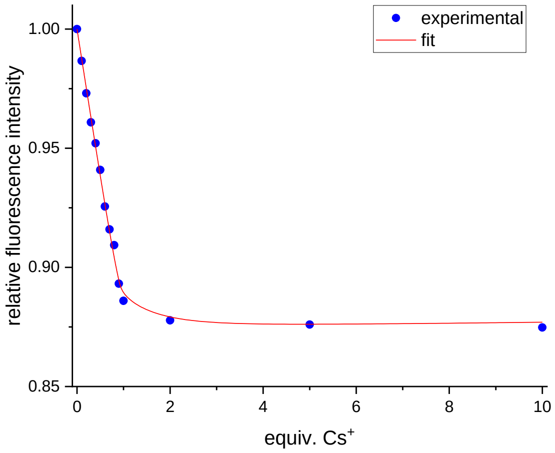

**5**-Cs^+ (C_5 = $5\cdot10^{-5}$ M)		
Stoichiom.	Mode	Covariance
**1:1**	**n.a.**	**$5.9\cdot10^{-3}$**
1:2	n.a.	n.a.[a]
	non-cooperative	n.a.[a]
	additive	n.a.[a]
	statistical	n.a.[a]
2:1	n.a.	fit failed
	non-cooperative	$2.1\cdot10^{-2}$
	additive	$2.3\cdot10^{-2}$
	statistical	$2.4\cdot10^{-2}$

[a] Covariance value not given due to very high *K* error value (>100-200%) or negative *K* (or *K* error) value, what is impossible

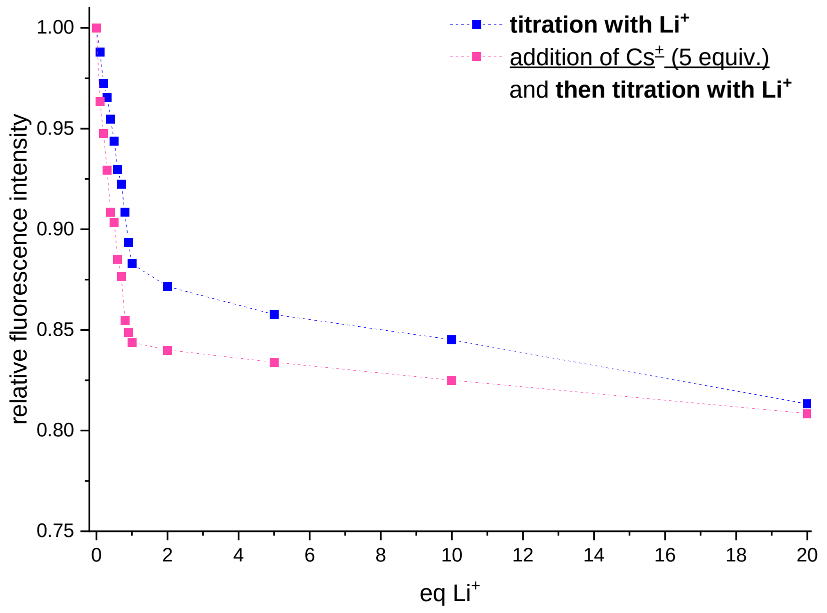

Fig. S65. Li^+ titration curve for receptor **4** in the presence or absence of the initial addition of Cs^+ (5 equiv.) to the solution. Conditions: λ_{ex} = 292 nm, λ_{em} = 480 nm, C_4 = $2{\cdot}10^{-5}$ M – aggregates, solvent = H_2O:THF = 95:5 *v*/*v*.

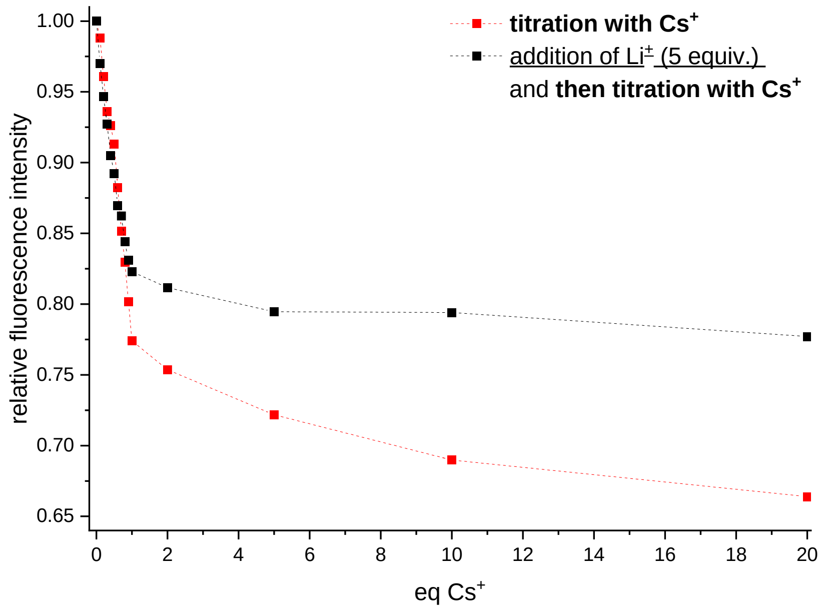

Fig. S66. Cs^+ titration curve for receptor **4** in the presence or absence of the initial addition of Li^+ (5 equiv.) to the solution. Conditions: λ_{ex} = 292 nm, λ_{em} = 480 nm, C_4 = $2{\cdot}10^{-5}$ M – aggregates, solvent = H_2O:THF = 95:5 *v*/*v*.

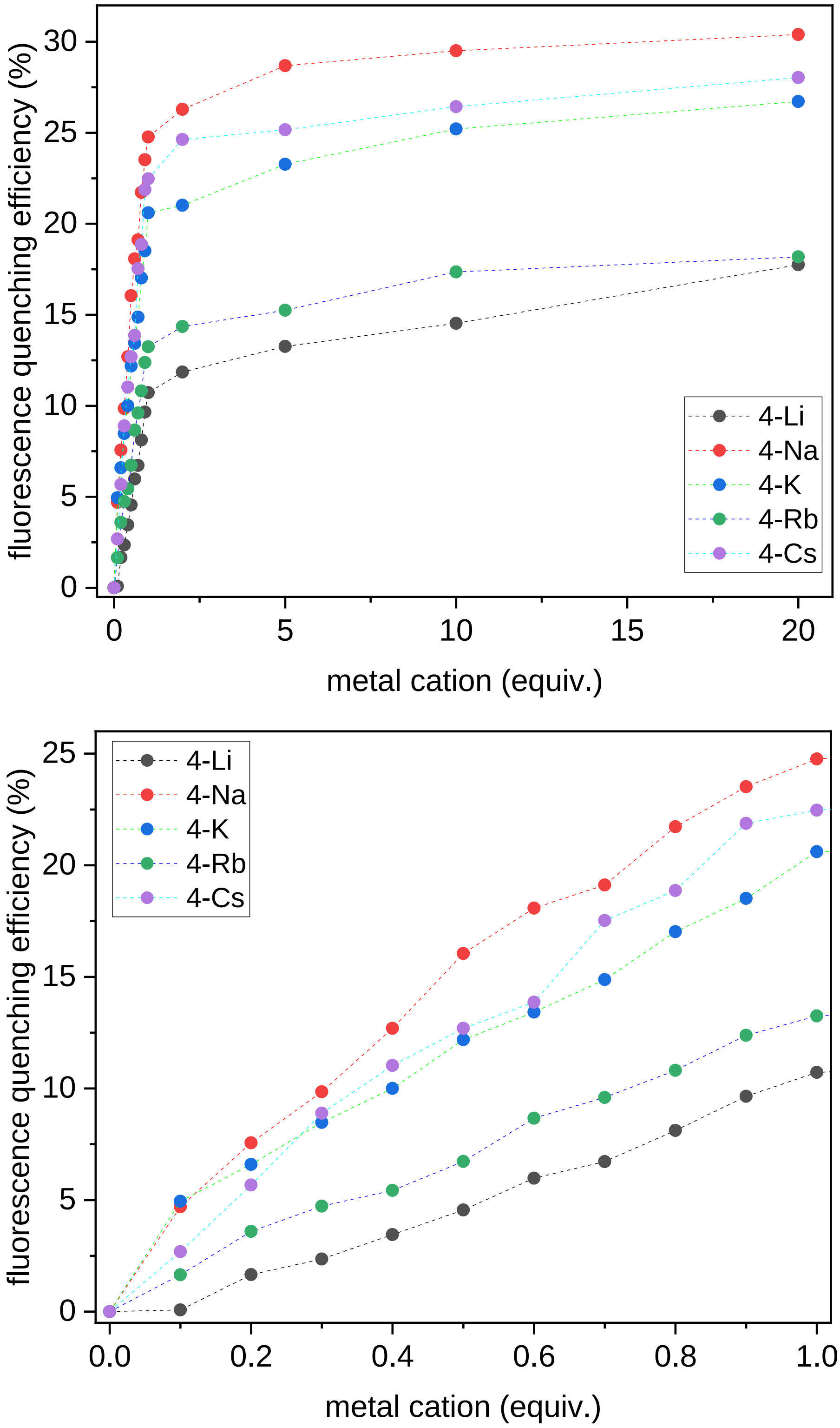

Fig. S67. Fluorescence quenching efficiency curves for the interactions between receptor **4** and tested alkali metal cations: (**top**) full metal concentration range, (**bottom**) inset for the range up to 1 equiv. of cation. Conditions: λ_{ex} = 292 nm, λ_{em} = 480 nm, C_4 = $2{\cdot}10^{-5}$ M – aggregates, solvent = H_2O:THF = 95:5 *v*/*v*.

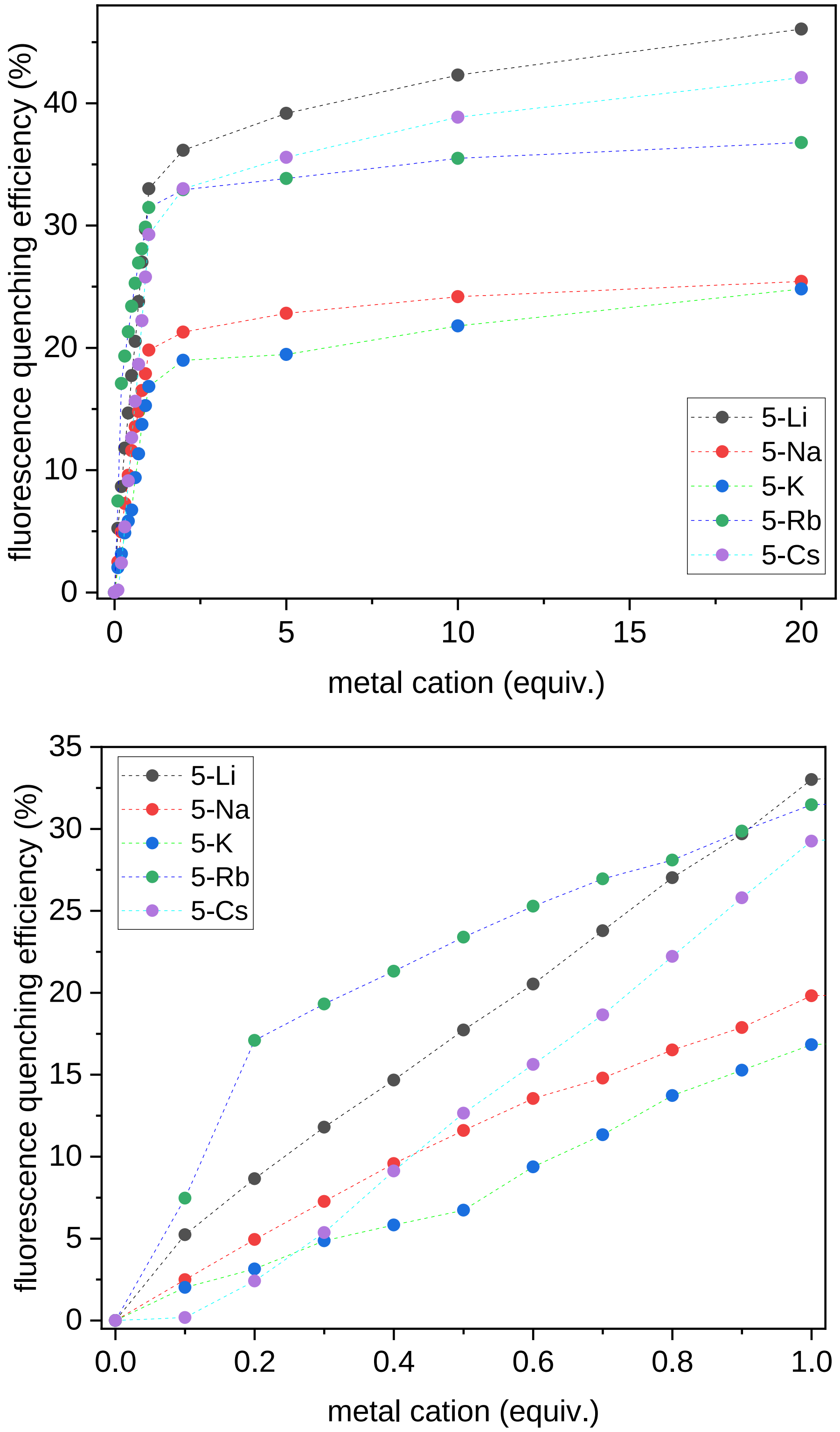

Fig. S68. Fluorescence quenching efficiency curves for the interactions between receptor **5** and tested alkali metal cations: (**top**) full metal concentration range, (**bottom**) inset for the range up to 1 equiv. of cation. Conditions: λ_{ex} = 292 nm, λ_{em} = 480 nm, C_5 = 2·10^{-5} M – aggregates, solvent = H_2O:THF = 95:5 *v*/*v*.

S6. DFT computations

Density functional theory (DFT) computations regarding structure optimization were performed with Gaussian software[12] with B3LYP functional[13] and 6-311++G(d,p) basis set[14]. The initial structure of sumanene was adopted from its crystal structure, which was further modified with Avogadro[15] and GaussView[16] software, and then subjected to calculation. After structure optimization, vibrational frequencies were calculated. All optimized compounds were stable geometric structures since no imaginary frequencies were detected.

Time-dependent-DFT (TD-DFT) computations for the UV-vis spectra calculations were performed at B3LYP/6-311++G(d,p) level of theory in a gas phase. The results were also compared by incorporating the solvent (THF, used for the experimental spectra) effect with Integral Equation Formalism Polarizable Continuum Model (IEFPCM)[17].

For the DFT-computations regarding interaction energy for **4**-Li^+ and **5**-Cs^+, ωB97X-D[18]/6-31G[19]/IEFPCM(H_2O incorporated as the solvent) and ωB97X-D/LANL2DZ[20]/IEFPCM(H_2O incorporated as the solvent) levels of theory were used, respectively. Similar comparative DFT computations were also performed for **5**-Li^+ and **4**-Cs^+ systems using above noted levels of theories. After structure optimization of the receptor (**4**, **5**), cation (Li/Cs) and complex, vibrational frequencies were calculated to provide thermal free energy correction to the electronic energy. Interaction energies (Gibbs free energies of association, ΔG) values were provided in kJ/mol. All optimized compounds were stable geometric structures since no imaginary frequencies were detected.

All data regarding DFT and TD-DFT computations are presented below.

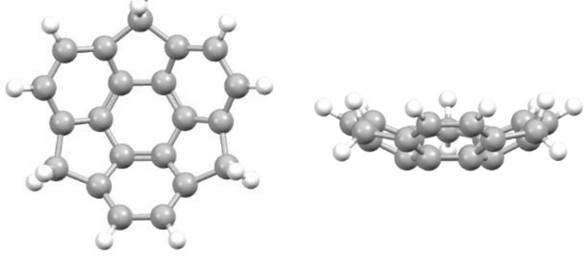

Fig. S69. DFT optimized (B3LYP/6-311++G(d,p)) structure of sumanene **1** (views from two different perspectives are presented).

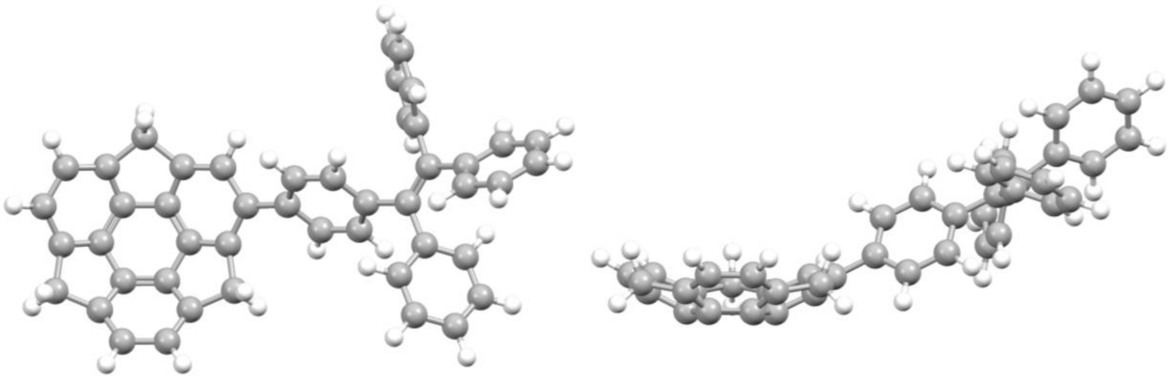

Fig. S70. DFT optimized (B3LYP/6-311++G(d,p)) structure of **4** (views from two different perspectives are presented).

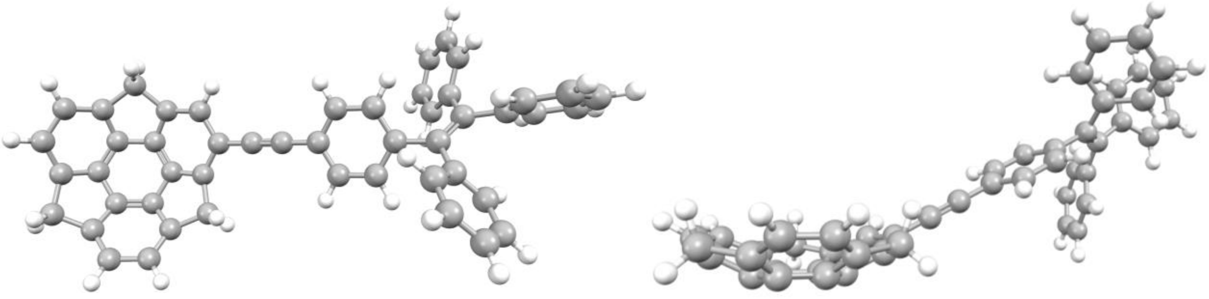

Fig. S71. DFT optimized (B3LYP/6-311++G(d,p)) structure of **5** (views from two different perspectives are presented).

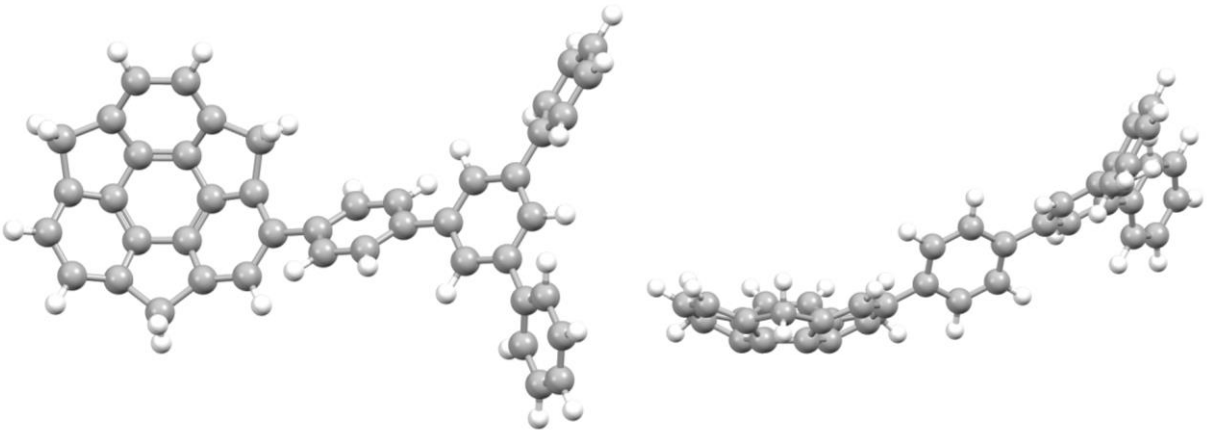

Fig. S72. DFT optimized (B3LYP/6-311++G(d,p)) structure of **6** (views from two different perspectives are presented).

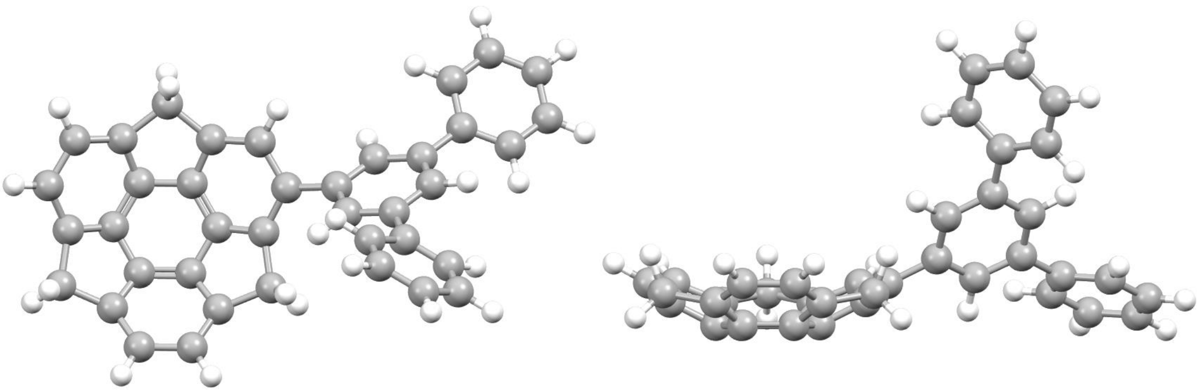

Fig. S73. DFT optimized (B3LYP/6-311++G(d,p)) structure of **7** (views from two different perspectives are presented).

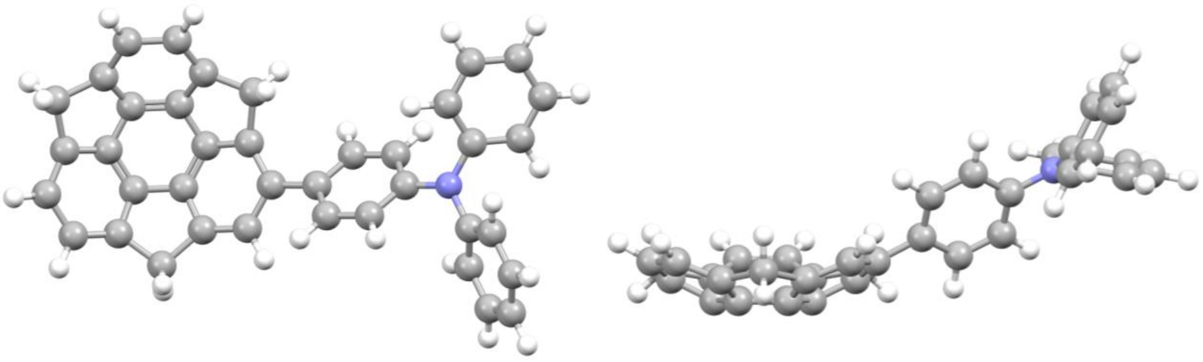

Fig. S74. DFT optimized (B3LYP/6-311++G(d,p)) structure of **8** (views from two different perspectives are presented).

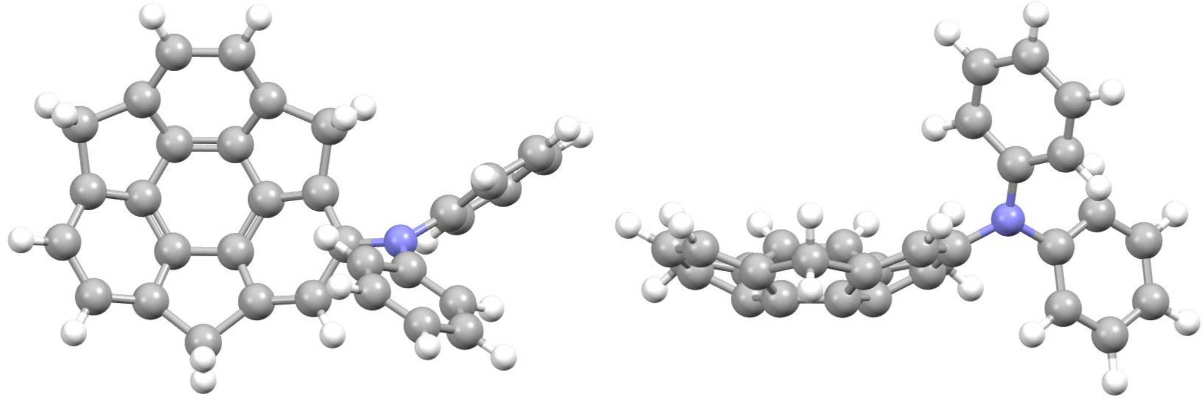

Fig. S75. DFT optimized (B3LYP/6-311++G(d,p)) structure of **9** (views from two different perspectives are presented).

a)

HOMO ΔE = 3.80 eV LUMO

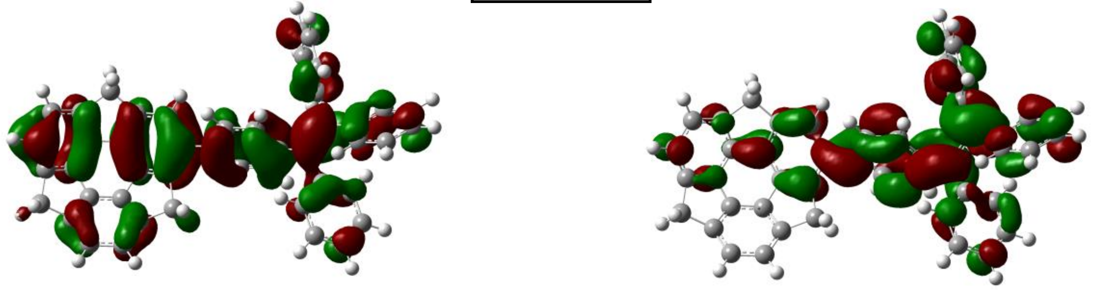

parameter	experimental	Calculated (gas phase, B3LYP/6-311++G(d,p))	Calculated (THF, B3LYP/ 6-311++G(d,p)/IEFPCM)
λ_{max} (nm)	341	347	347
ε ($dm^3 \cdot mol^{-1} \cdot cm^{-1}$)	20000	6537	9348

b)

HOMO ΔE = 3.45 eV LUMO

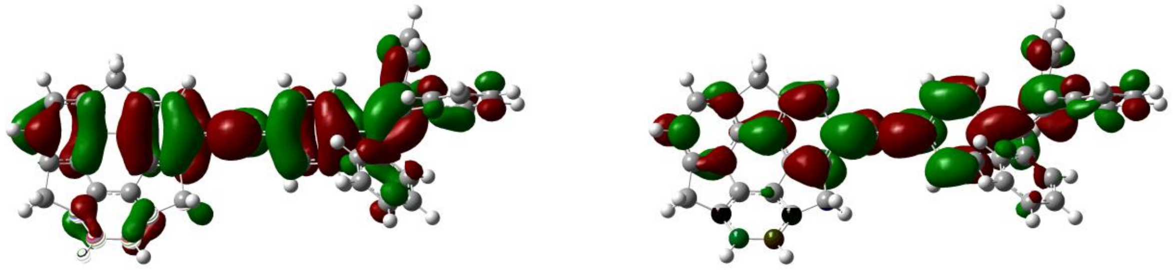

parameter	experimental	Calculated (gas phase, B3LYP/6-311++G(d,p))	Calculated (THF, B3LYP/ 6-311++G(d,p)/IEFPCM)
λ_{max} (nm)	358	362	365
ε ($dm^3 \cdot mol^{-1} \cdot cm^{-1}$)	29700	17190	39763

Fig. S76. Calculated HOMO and LUMO orbitals, HOMO-LUMO gaps (ΔE) and UV-vis absorption bands for **4** (**a**) and **5** (**b**) based on TD-DFT computations.

Table S5. Comparison of bowl depths for DFT optimized (B3LYP/6-311++G(d,p)) **4-9** structures.

cpd.	structure	bowl depth (Å)	bowl depth graph
**1**	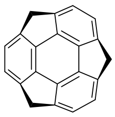	1.144	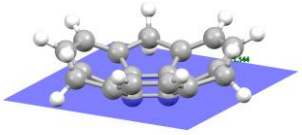
**4**	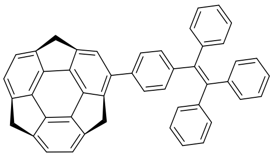	1.143	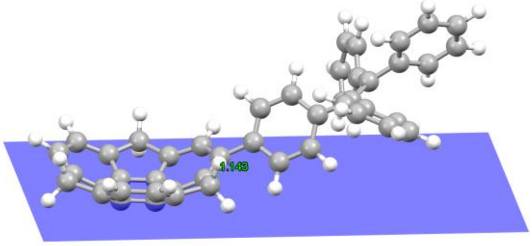
**5**	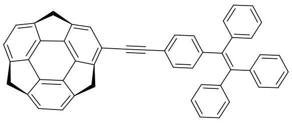	1.144	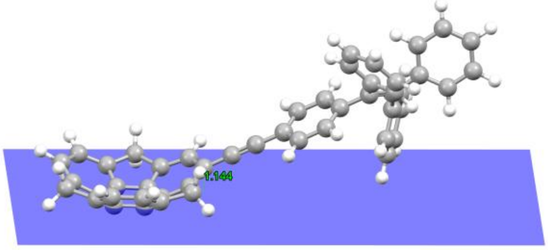
**6**	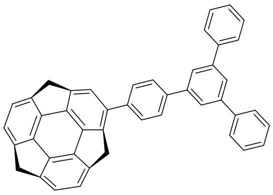	1.150	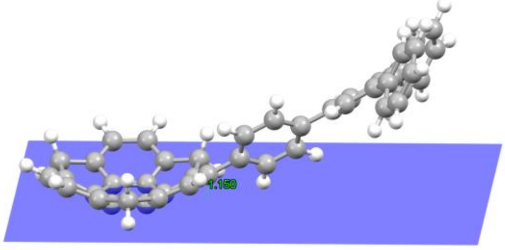
**7**	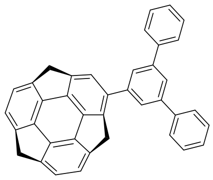	1.122	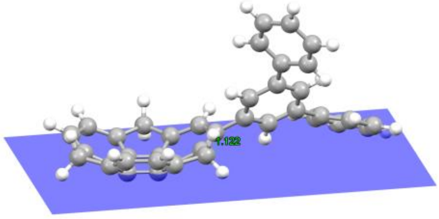
**8**	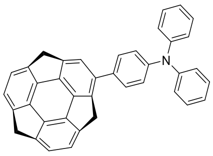	1.150	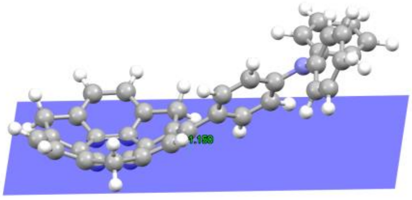
**9**	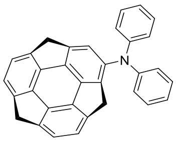	1.150	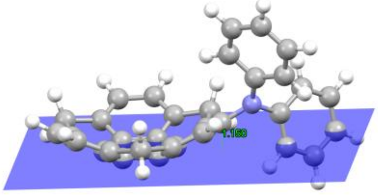

4-Li$^+$ complex, arrangement 1 (concave)
Δ*G* = −110.38 kJ/mol

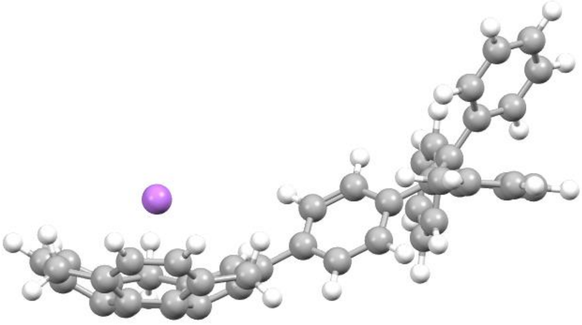
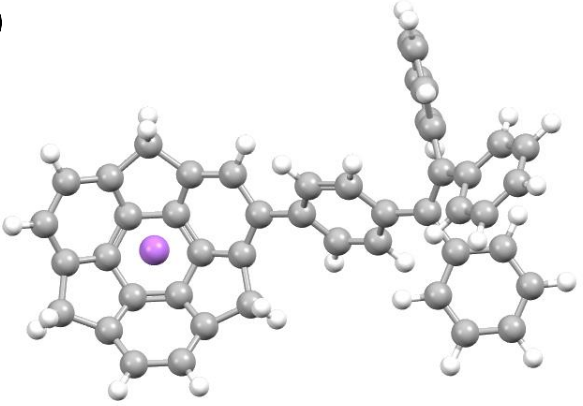

4-Li$^+$ complex, arrangement 2 (convex)
Δ*G* = −113.36 kJ/mol

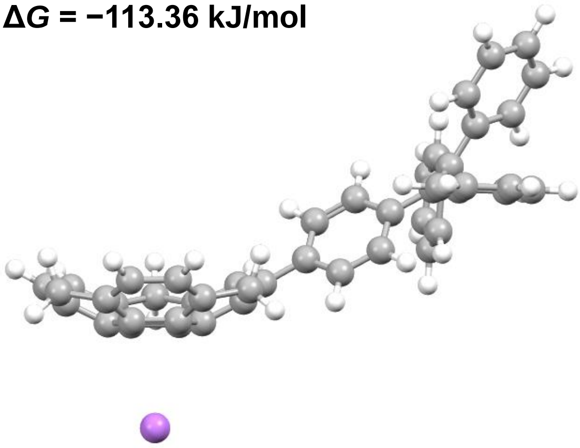
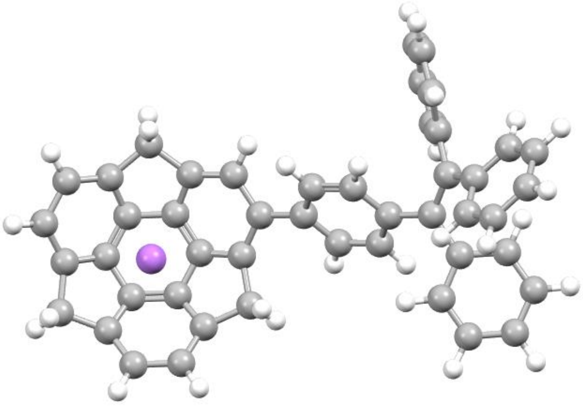

4-Li$^+$ complex, arrangement 3 (convex)
Δ*G* = −116.44 kJ/mol

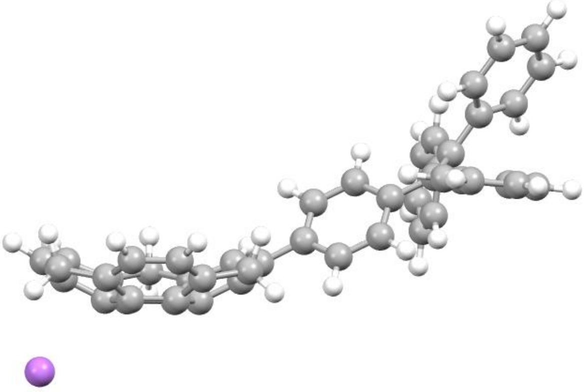
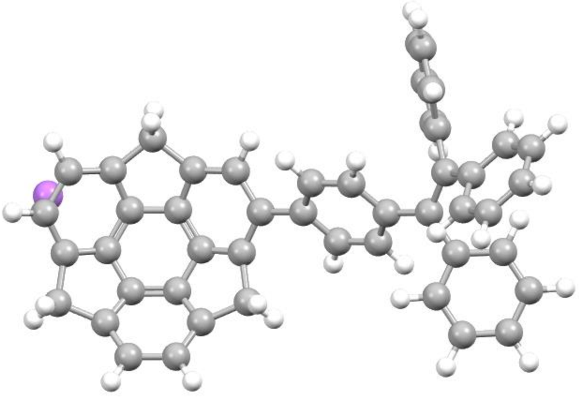

Fig. S77. DFT optimized (ωB97X-D/6-31G/IEFPCM(H_2O)) structures of **4**-Li$^+$ complexes (views from two perspectives), together with their interaction energies.

5-Cs⁺ complex, arrangement 1 (concave)
Δ*G* = −172.64 kJ/mol

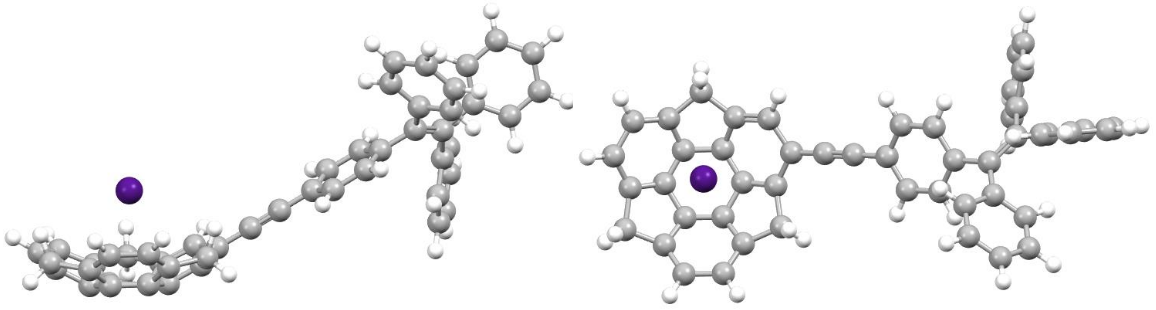

5-Cs⁺ complex, arrangement 2 (convex)
Δ*G* = −129.60 kJ/mol

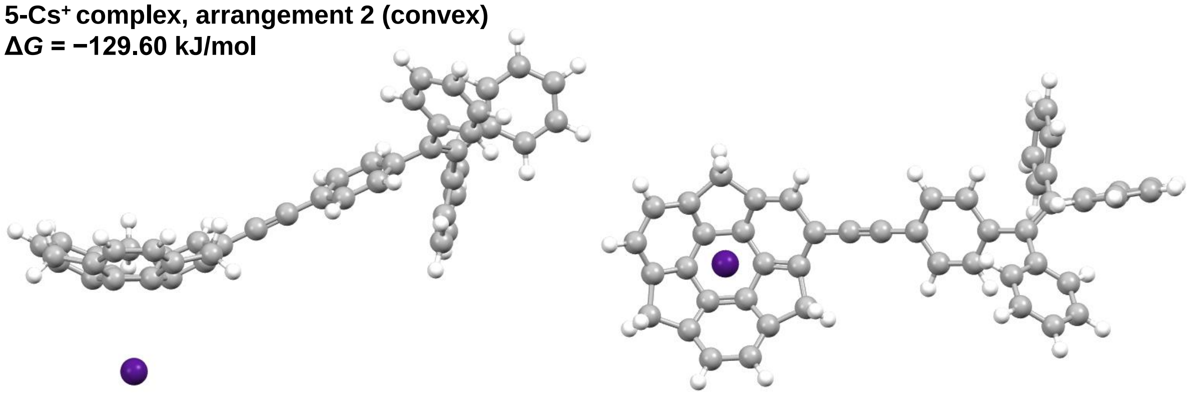

5-Cs⁺ complex, arrangement 3 (convex)
Δ*G* = −127.07 kJ/mol

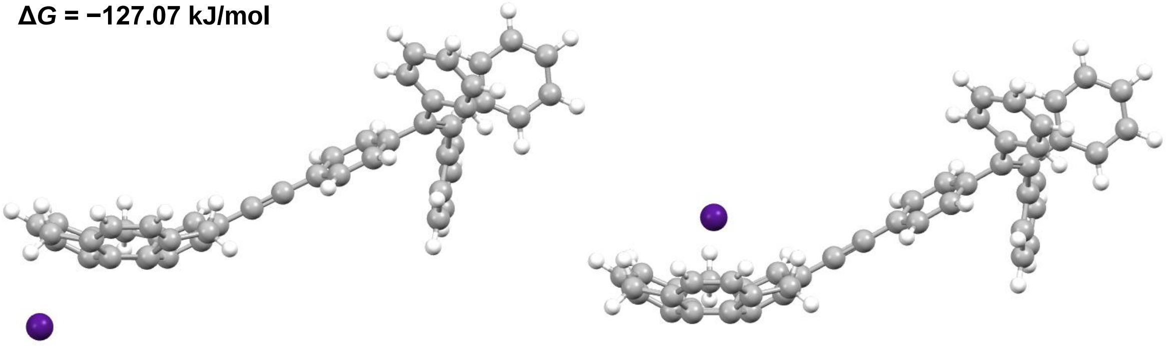

Fig. S78. DFT optimized (ωB97X-D/LANL2DZ/IEFPCM(H_2O)) structures of **5**-Cs^+ complexes (views from two perspectives), together with their interaction energies.

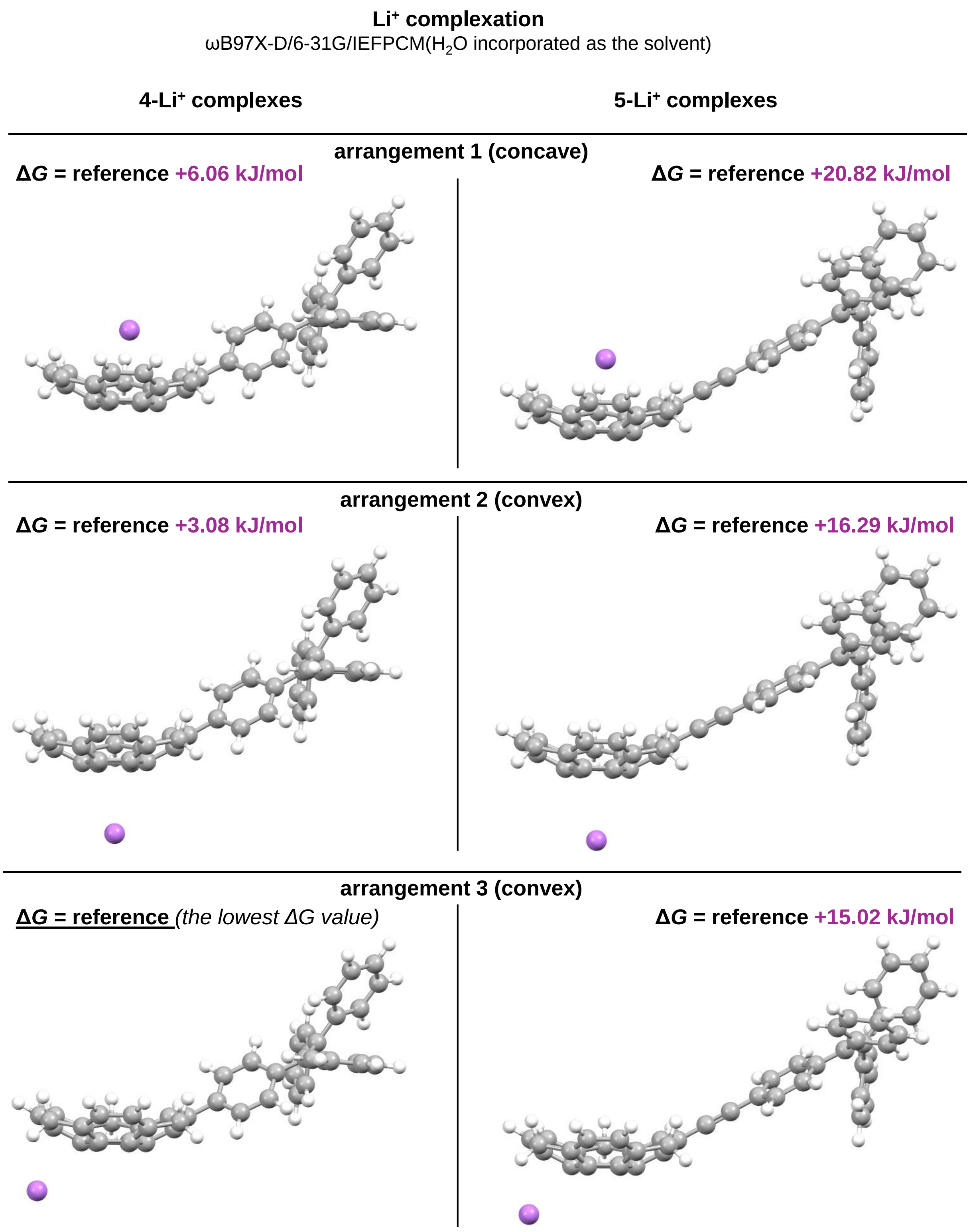

Fig. S79. Graphical comparison of interaction energies (Δ*G*) for **4**-Li^+ and **5**-Li^+ complexes (DFT-optimized; ωB97X-D/6-31G/IEFPCM(H_2O); a view from one perspective is presented for image clarity). Reference Δ*G* value was taken for the system featuring the lowest value Δ*G* in series (**4**-Li^+, arrangement 3, convex).

Cs^+ complexation

ωB97X-D/LANL2DZ/IEFPCM(H2O incorporated as the solvent)

4-Cs^+ complexes | **5-Cs^+ complexes**

arrangement 1 (concave)

Δ*G* = reference +9.77 kJ/mol

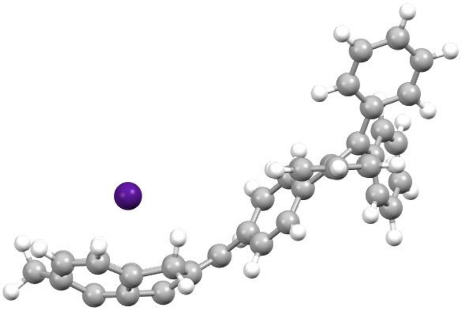

Δ*G* = reference *(the lowest ΔG value)*

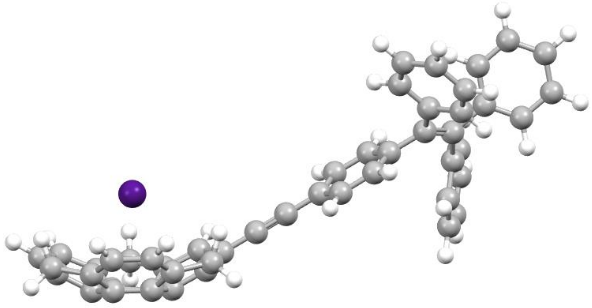

arrangement 2 (convex)

Δ*G* = reference +50.82 kJ/mol

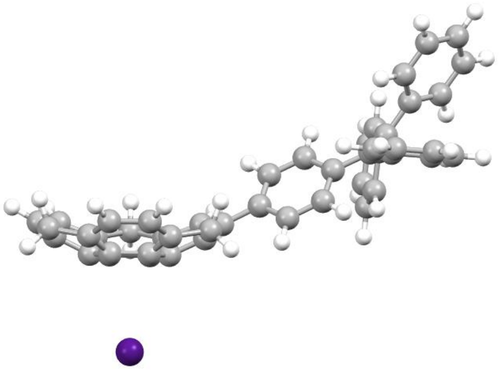

Δ*G* = reference +43.04 kJ/mol

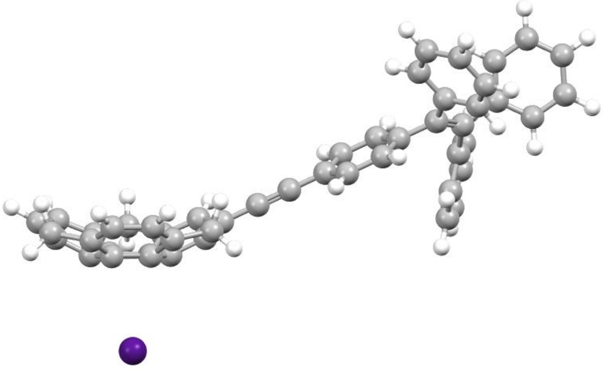

arrangement 3 (convex)

Δ*G* = reference +53.28 kJ/mol

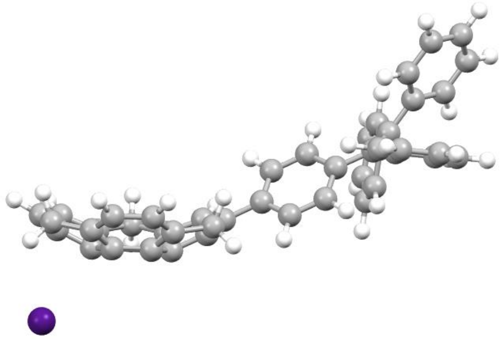

Δ*G* = reference +45.57 kJ/mol

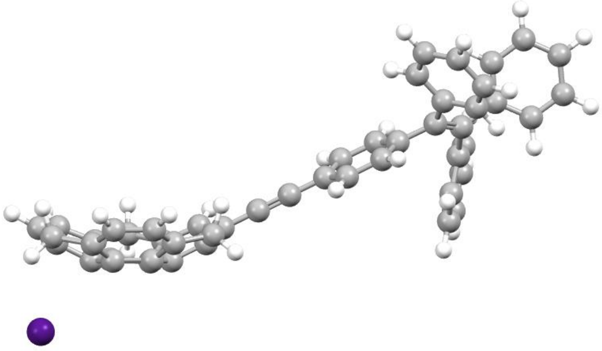

Fig. S80. Graphical comparison of interaction energies (Δ*G*) for **4**-Cs^+ and **5**-Cs^+ complexes (DFT-optimized; ωB97X-D/LANL2DZ/IEFPCM(H_2O); a view from one perspective is presented for image clarity). Reference Δ*G* value was taken for the system featuring the lowest Δ*G* value in series (**5**-Cs^+, arrangement 1, concave).

Table S6. Atomic coordinates for the DFT optimized (B3LYP/6-311++G(d,p)) structure of sumanene **1**.

	x	*y*	*z*
C	3.2908000000	0.7886000000	0.4480000000
C	-1.1064000000	3.1975000000	0.4489000000
C	3.3229000000	-0.6407000000	0.4477000000
C	2.2610000000	-1.3875000000	-0.0691000000
C	1.2421000000	-0.6650000000	-0.6954000000
C	1.7112000000	-2.8167000000	0.1862000000
C	0.0711000000	2.6515000000	-0.0684000000
C	-0.9624000000	-3.2438000000	0.4488000000
C	1.5836000000	2.8904000000	0.1860000000
C	-2.3283000000	2.4554000000	0.4486000000
C	-2.3862000000	1.1588000000	-0.0688000000
C	2.1967000000	1.4872000000	-0.0689000000
C	1.2113000000	0.7198000000	-0.6954000000
C	0.1896000000	-2.6457000000	-0.0685000000
C	0.0177000000	-1.4088000000	-0.6953000000
C	-0.0451000000	1.4080000000	-0.6952000000
C	-2.2162000000	-2.5569000000	0.4489000000
C	-3.2953000000	-0.0736000000	0.1854000000
C	-1.2289000000	0.6889000000	-0.6953000000
C	-2.3322000000	-1.2642000000	-0.0687000000
C	-1.1971000000	-0.7433000000	-0.6954000000
H	4.0830000000	1.3079000000	0.9782000000
H	-1.0950000000	4.1446000000	0.9792000000
H	4.1376000000	-1.1241000000	0.9775000000
H	1.9150000000	-3.1516000000	1.2062000000
H	2.1589000000	-3.5541000000	-0.4906000000
H	-0.9086000000	-4.1894000000	0.9791000000
H	1.7724000000	3.2345000000	1.2059000000
H	1.9977000000	3.6469000000	-0.4911000000
H	-3.1742000000	2.8817000000	0.9787000000
H	-3.0420000000	-3.0205000000	0.9793000000
H	-4.1566000000	-0.0928000000	-0.4929000000
H	-3.6890000000	-0.0822000000	1.2047000000

Table S. Atomic coordinates for the DFT optimized (B3LYP/6-311++G(d,p)) structure of sumanene **4**.

	x	*y*	*z*
C	8.0075000000	-1.3757000000	1.5451000000
C	6.2632000000	3.2464000000	0.6828000000
C	7.1781000000	-2.5106000000	1.2881000000
C	6.1453000000	-2.4534000000	0.3497000000
C	6.0587000000	-1.2810000000	-0.4049000000
C	4.8052000000	-3.2139000000	0.1580000000
C	6.9688000000	2.0637000000	0.4503000000
C	2.5312000000	-1.8684000000	-0.4783000000
C	8.1160000000	1.3189000000	1.1851000000
C	4.9387000000	3.4275000000	0.1797000000
C	4.3042000000	2.4276000000	-0.5612000000
C	7.8133000000	-0.1702000000	0.8668000000
C	6.8624000000	-0.1815000000	-0.1559000000
C	3.8979000000	-2.1384000000	-0.4990000000
C	4.7322000000	-1.0974000000	-0.9094000000
C	6.3620000000	1.1375000000	-0.4011000000
C	2.0073000000	-0.5524000000	-0.7333000000
C	2.8258000000	2.0455000000	-0.8292000000
C	5.0780000000	1.3130000000	-0.8892000000
C	2.8958000000	0.5032000000	-1.0173000000
C	4.2459000000	0.1760000000	-1.1518000000
C	0.5548000000	-0.3066000000	-0.5564000000
C	-0.1790000000	-0.9039000000	0.4805000000
C	-1.5400000000	-0.6710000000	0.6275000000
C	-2.2319000000	0.1747000000	-0.2516000000
C	-1.4944000000	0.7954000000	-1.2706000000
C	-0.1361000000	0.5509000000	-1.4262000000
C	-3.6885000000	0.4552000000	-0.0858000000
C	-4.6134000000	-0.5295000000	0.0859000000
C	-4.0529000000	1.9046000000	-0.1266000000
C	-6.0256000000	-0.2498000000	0.4870000000
C	-4.2982000000	-1.9766000000	-0.1156000000
C	-3.6755000000	-2.4303000000	-1.2872000000
C	-3.4145000000	-3.7836000000	-1.4796000000
C	-3.7671000000	-4.7128000000	-0.5029000000
C	-4.3961000000	-4.2780000000	0.6626000000
C	-4.6696000000	-2.9264000000	0.8481000000
C	-3.3507000000	2.8366000000	0.6524000000
C	-3.6706000000	4.1901000000	0.6095000000
C	-4.6841000000	4.6447000000	-0.2325000000
C	-5.3740000000	3.7336000000	-1.0295000000

C	-5.0632000000	2.3781000000	-0.9755000000
C	-6.3237000000	0.5605000000	1.5918000000
C	-7.6417000000	0.7897000000	1.9751000000
C	-8.6911000000	0.2169000000	1.2596000000
C	-8.4103000000	-0.5990000000	0.1649000000
C	-7.0919000000	-0.8393000000	-0.2091000000
H	8.7055000000	-1.4418000000	2.3737000000
H	6.6518000000	4.0026000000	1.3575000000
H	7.2938000000	-3.3737000000	1.9361000000
H	4.3968000000	-3.5592000000	1.1108000000
H	4.9227000000	-4.0965000000	-0.4817000000
H	1.8234000000	-2.6299000000	-0.1689000000
H	8.0986000000	1.5128000000	2.2604000000
H	9.1035000000	1.6237000000	0.8187000000
H	4.3958000000	4.3108000000	0.5009000000
H	2.4297000000	2.5562000000	-1.7142000000
H	2.1755000000	2.3138000000	0.0071000000
H	0.3284000000	-1.5377000000	1.1986000000
H	-2.0757000000	-1.1412000000	1.4431000000
H	-1.9972000000	1.4659000000	-1.9583000000
H	0.3974000000	1.0127000000	-2.2491000000
H	-3.3978000000	-1.7145000000	-2.0513000000
H	-2.9367000000	-4.1126000000	-2.3958000000
H	-3.5612000000	-5.7668000000	-0.6518000000
H	-4.6799000000	-4.9935000000	1.4265000000
H	-5.1722000000	-2.5982000000	1.7509000000
H	-2.5525000000	2.4925000000	1.3003000000
H	-3.1247000000	4.8915000000	1.2309000000
H	-4.9288000000	5.7003000000	-0.2722000000
H	-6.1561000000	4.0783000000	-1.6969000000
H	-5.6054000000	1.6760000000	-1.5972000000
H	-5.5139000000	1.0103000000	2.1533000000
H	-7.8486000000	1.4159000000	2.8359000000
H	-9.7180000000	0.3983000000	1.5564000000
H	-9.2194000000	-1.0539000000	-0.3958000000
H	-6.8818000000	-1.4865000000	-1.0531000000

Table S8. Atomic coordinates for the DFT optimized (B3LYP/6-311++G(d,p)) structure of sumanene **5**.

	x	*y*	*z*
C	9.4191000000	-0.4633000000	1.9548000000
C	7.5075000000	3.1984000000	-0.8863000000
C	8.6320000000	-1.6177000000	2.2584000000
C	7.5984000000	-2.0299000000	1.4141000000
C	7.4679000000	-1.3344000000	0.2085000000
C	6.2902000000	-2.8412000000	1.6132000000
C	8.2569000000	2.0623000000	-0.5695000000
C	3.9668000000	-2.0114000000	0.4848000000
C	9.4309000000	1.7711000000	0.4033000000
C	6.1750000000	3.0884000000	-1.3915000000
C	5.5765000000	1.8407000000	-1.5860000000
C	9.1808000000	0.2925000000	0.8038000000
C	8.2305000000	-0.2153000000	-0.0854000000
C	5.3406000000	-2.2156000000	0.5562000000
C	6.1353000000	-1.4445000000	-0.2992000000
C	7.6837000000	0.8309000000	-0.8967000000
C	3.3998000000	-0.9626000000	-0.3336000000
C	4.1109000000	1.3318000000	-1.6098000000
C	6.3928000000	0.7246000000	-1.3876000000
C	4.2476000000	-0.1154000000	-1.0776000000
C	5.6041000000	-0.4329000000	-1.0823000000
C	2.0036000000	-0.7120000000	-0.2537000000
C	0.8118000000	-0.4968000000	-0.1947000000
C	-0.5858000000	-0.2509000000	-0.1257000000
C	-1.4186000000	-1.0354000000	0.6941000000
C	-2.7839000000	-0.7975000000	0.7523000000
C	-3.3776000000	0.2324000000	0.0056000000
C	-2.5391000000	1.0305000000	-0.7900000000
C	-1.1754000000	0.7895000000	-0.8684000000
C	-4.8399000000	0.5158000000	0.0898000000
C	-5.7873000000	-0.4567000000	-0.0242000000
C	-5.1851000000	1.9548000000	0.3048000000
C	-7.2268000000	-0.2218000000	0.3007000000
C	-5.4702000000	-1.8411000000	-0.4892000000
C	-4.5542000000	2.6951000000	1.3162000000
C	-4.8560000000	4.0398000000	1.5130000000
C	-5.7783000000	4.6800000000	0.6857000000
C	-6.3951000000	3.9629000000	-0.3383000000
C	-6.1029000000	2.6143000000	-0.5251000000
C	-7.6139000000	0.3564000000	1.5184000000
C	-8.9590000000	0.5427000000	1.8260000000

C	-9.9466000000	0.1588000000	0.9202000000
C	-9.5772000000	-0.4266000000	-0.2905000000
C	-8.2326000000	-0.6260000000	-0.5906000000
C	-4.7471000000	-2.0644000000	-1.6700000000
C	-4.4814000000	-3.3576000000	-2.1123000000
C	-4.9302000000	-4.4561000000	-1.3808000000
C	-5.6599000000	-4.2497000000	-0.2104000000
C	-5.9375000000	-2.9564000000	0.2231000000
H	10.1187000000	-0.1207000000	2.7106000000
H	7.8682000000	4.1909000000	-0.6353000000
H	8.7795000000	-2.0846000000	3.2270000000
H	5.8953000000	-2.7321000000	2.6260000000
H	6.4433000000	-3.9128000000	1.4396000000
H	3.2820000000	-2.5435000000	1.1360000000
H	9.4100000000	2.4341000000	1.2717000000
H	10.4058000000	1.9043000000	-0.0804000000
H	5.6004000000	4.0035000000	-1.4942000000
H	3.6784000000	1.3593000000	-2.6169000000
H	3.4600000000	1.9307000000	-0.9679000000
H	-0.9812000000	-1.8312000000	1.2851000000
H	-3.4034000000	-1.4157000000	1.3905000000
H	-2.9669000000	1.8470000000	-1.3606000000
H	-0.5504000000	1.4070000000	-1.5025000000
H	-3.8255000000	2.2093000000	1.9556000000
H	-4.3670000000	4.5896000000	2.3097000000
H	-6.0083000000	5.7292000000	0.8333000000
H	-7.1043000000	4.4542000000	-0.9953000000
H	-6.5876000000	2.0644000000	-1.3228000000
H	-6.8534000000	0.6584000000	2.2283000000
H	-9.2356000000	0.9866000000	2.7761000000
H	-10.9940000000	0.3069000000	1.1583000000
H	-10.3376000000	-0.7338000000	-1.0002000000
H	-7.9556000000	-1.0957000000	-1.5277000000
H	-4.3944000000	-1.2164000000	-2.2447000000
H	-3.9246000000	-3.5067000000	-3.0308000000
H	-4.7202000000	-5.4632000000	-1.7232000000
H	-6.0184000000	-5.0975000000	0.3632000000
H	-6.5181000000	-2.8056000000	1.1264000000

Table S9. Atomic coordinates for the DFT optimized (B3LYP/6-311++G(d,p)) structure of sumanene **6**.

	x	*y*	*z*
C	-8.3835000000	-1.4820000000	1.3360000000
C	-6.3339000000	3.0931000000	1.3244000000
C	-7.6388000000	-2.5991000000	0.8442000000
C	-6.6195000000	-2.4305000000	-0.0962000000
C	-6.4633000000	-1.1432000000	-0.6173000000
C	-5.3393000000	-3.2268000000	-0.4665000000
C	-7.1245000000	2.0270000000	0.8877000000
C	-2.9857000000	-1.9365000000	-0.8901000000
C	-8.3105000000	1.2325000000	1.4975000000
C	-5.0063000000	3.2799000000	0.8293000000
C	-4.4547000000	2.4025000000	-0.1080000000
C	-8.1166000000	-0.1839000000	0.8930000000
C	-7.1845000000	-0.0612000000	-0.1406000000
C	-4.3685000000	-2.1071000000	-0.9293000000
C	-5.1339000000	-0.9536000000	-1.1132000000
C	-6.5966000000	1.2448000000	-0.1428000000
C	-2.3754000000	-0.6331000000	-0.9031000000
C	-3.0106000000	1.9825000000	-0.4834000000
C	-5.3100000000	1.4263000000	-0.6235000000
C	-3.1913000000	0.5136000000	-0.9600000000
C	-4.5632000000	0.3081000000	-1.1206000000
C	-0.9073000000	-0.5196000000	-0.7163000000
C	-0.2070000000	-1.3385000000	0.1831000000
C	1.1680000000	-1.2193000000	0.3464000000
C	1.9080000000	-0.2747000000	-0.3802000000
C	1.2091000000	0.5461000000	-1.2767000000
C	-0.1662000000	0.4265000000	-1.4399000000
C	3.3772000000	-0.1480000000	-0.2057000000
C	3.9899000000	1.1111000000	-0.1847000000
C	5.3709000000	1.2468000000	-0.0002000000
C	6.1430000000	0.0906000000	0.1650000000
C	5.5609000000	-1.1825000000	0.1459000000
C	4.1776000000	-1.2848000000	-0.0400000000
C	6.3968000000	-2.4021000000	0.3002000000
C	6.0044000000	2.5913000000	0.0207000000
C	6.1656000000	-3.5394000000	-0.4877000000
C	6.9480000000	-4.6820000000	-0.3418000000
C	7.9790000000	-4.7126000000	0.5958000000
C	8.2205000000	-3.5898000000	1.3858000000
C	7.4380000000	-2.4474000000	1.2391000000
C	7.0119000000	2.8992000000	0.9468000000

C	7.6056000000	4.1586000000	0.9667000000
C	7.2046000000	5.1384000000	0.0600000000
C	6.2046000000	4.8471000000	-0.8664000000
C	5.6114000000	3.5874000000	-0.8854000000
H	-9.0709000000	-1.6599000000	2.1570000000
H	-6.6582000000	3.7281000000	2.1428000000
H	-7.8039000000	-3.5605000000	1.3203000000
H	-5.5281000000	-3.9578000000	-1.2616000000
H	-4.9432000000	-3.7780000000	0.3897000000
H	-2.3295000000	-2.7884000000	-0.7462000000
H	-9.2788000000	1.6662000000	1.2208000000
H	-8.2664000000	1.2154000000	2.5892000000
H	-4.3980000000	4.0455000000	1.3004000000
H	-2.3312000000	2.0430000000	0.3705000000
H	-2.5932000000	2.6261000000	-1.2662000000
H	-0.7498000000	-2.0558000000	0.7881000000
H	1.6703000000	-1.8457000000	1.0749000000
H	1.7531000000	1.2655000000	-1.8784000000
H	-0.6719000000	1.0528000000	-2.1660000000
H	3.3766000000	2.0010000000	-0.2635000000
H	7.2187000000	0.1807000000	0.2602000000
H	3.7166000000	-2.2653000000	-0.0567000000
H	5.3825000000	-3.5191000000	-1.2373000000
H	6.7576000000	-5.5465000000	-0.9683000000
H	8.5883000000	-5.6020000000	0.7094000000
H	9.0146000000	-3.6056000000	2.1242000000
H	7.6205000000	-1.5885000000	1.8751000000
H	7.3155000000	2.1530000000	1.6723000000
H	8.3765000000	4.3774000000	1.6973000000
H	7.6667000000	6.1190000000	0.0750000000
H	5.8916000000	5.5990000000	-1.5824000000
H	4.8520000000	3.3647000000	-1.6264000000

Table S10. Atomic coordinates for the DFT optimized (B3LYP/6-311++G(d,p)) structure of sumanene **7**.

	x	*y*	*z*
C	6.3471000000	-0.8597000000	1.7010000000
C	4.7411000000	2.5007000000	-1.6652000000
C	5.4358000000	-1.8155000000	2.2497000000
C	4.2904000000	-2.2069000000	1.5513000000
C	4.1608000000	-1.7132000000	0.2508000000
C	2.9018000000	-2.7691000000	1.9618000000
C	5.3471000000	1.3392000000	-1.1786000000
C	0.6268000000	-1.8173000000	0.8121000000
C	6.5427000000	1.0486000000	-0.2312000000
C	3.3714000000	2.5053000000	-2.0756000000
C	2.5908000000	1.3486000000	-2.0004000000
C	6.1237000000	-0.2838000000	0.4474000000
C	5.0435000000	-0.7881000000	-0.2807000000
C	1.9665000000	-2.2028000000	0.8587000000
C	2.7880000000	-1.7060000000	-0.1545000000
C	4.5831000000	0.1711000000	-1.2389000000
C	0.1452000000	-0.8426000000	-0.1310000000
C	1.0714000000	1.0681000000	-1.8646000000
C	3.2545000000	0.1776000000	-1.6301000000
C	1.0399000000	-0.2525000000	-1.0414000000
C	2.3384000000	-0.7676000000	-1.0663000000
C	-1.2802000000	-0.4139000000	-0.0157000000
C	-1.6215000000	0.9100000000	0.2788000000
C	-2.9594000000	1.3120000000	0.3772000000
C	-3.9633000000	0.3577000000	0.1739000000
C	-3.6534000000	-0.9768000000	-0.1137000000
C	-2.3051000000	-1.3473000000	-0.2030000000
C	-4.7312000000	-1.9804000000	-0.3142000000
C	-3.3047000000	2.7194000000	0.7088000000
C	-4.6157000000	-3.2770000000	0.2087000000
C	-5.6253000000	-4.2173000000	0.0211000000
C	-6.7735000000	-3.8818000000	-0.6946000000
C	-6.9022000000	-2.5975000000	-1.2210000000
C	-5.8921000000	-1.6578000000	-1.0326000000
C	-4.3592000000	3.3751000000	0.0563000000
C	-4.6820000000	4.6942000000	0.3641000000
C	-3.9570000000	5.3871000000	1.3323000000
C	-2.9068000000	4.7483000000	1.9897000000
C	-2.5843000000	3.4293000000	1.6810000000
H	7.1460000000	-0.5030000000	2.3435000000
H	5.2554000000	3.4558000000	-1.6251000000

H	5.5961000000	-2.1284000000	3.2766000000
H	2.6050000000	-2.4281000000	2.9567000000
H	2.8944000000	-3.8653000000	1.9817000000
H	-0.0699000000	-2.1363000000	1.5808000000
H	6.6818000000	1.8485000000	0.5003000000
H	7.4857000000	0.9481000000	-0.7814000000
H	2.9257000000	3.4630000000	-2.3250000000
H	0.5898000000	0.9410000000	-2.8416000000
H	0.5537000000	1.8873000000	-1.3648000000
H	-0.8363000000	1.6390000000	0.4378000000
H	-5.0007000000	0.6468000000	0.2940000000
H	-2.0460000000	-2.3681000000	-0.4591000000
H	-3.7376000000	-3.5430000000	0.7861000000
H	-5.5184000000	-5.2111000000	0.4416000000
H	-7.5596000000	-4.6139000000	-0.8412000000
H	-7.7870000000	-2.3287000000	-1.7874000000
H	-5.9930000000	-0.6699000000	-1.4676000000
H	-4.9154000000	2.8540000000	-0.7148000000
H	-5.4958000000	5.1841000000	-0.1592000000
H	-4.2084000000	6.4139000000	1.5726000000
H	-2.3424000000	5.2748000000	2.7515000000
H	-1.7813000000	2.9355000000	2.2165000000

Table S11. Atomic coordinates for the DFT optimized (B3LYP/6-311++G(d,p)) structure of sumanene **8**.

	x	*y*	*z*
C	-7.1313000000	-1.4544000000	1.2340000000
C	-5.1533000000	3.1466000000	1.0145000000
C	-6.3488000000	-2.5867000000	0.8466000000
C	-5.2928000000	-2.4597000000	-0.0592000000
C	-5.1343000000	-1.2055000000	-0.6551000000
C	-3.9858000000	-3.2544000000	-0.3241000000
C	-5.9081000000	2.0409000000	0.6142000000
C	-1.6365000000	-1.9515000000	-0.7333000000
C	-7.1071000000	1.2657000000	1.2233000000
C	-3.8088000000	3.3253000000	0.5643000000
C	-3.2042000000	2.4003000000	-0.2910000000
C	-6.8658000000	-0.1826000000	0.7198000000
C	-5.8924000000	-0.1089000000	-0.2798000000
C	-3.0136000000	-2.1490000000	-0.8176000000
C	-3.7884000000	-1.0240000000	-1.1077000000
C	-5.3246000000	1.2046000000	-0.3410000000
C	-1.0446000000	-0.6408000000	-0.8046000000
C	-1.7393000000	1.9837000000	-0.5787000000
C	-4.0215000000	1.3782000000	-0.7785000000
C	-1.8763000000	0.4846000000	-0.9685000000
C	-3.2369000000	0.2446000000	-1.1721000000
N	4.5969000000	-0.0247000000	0.0654000000
C	3.2026000000	-0.1776000000	-0.1451000000
C	5.4128000000	-1.1666000000	0.2935000000
C	5.1834000000	1.2704000000	0.0438000000
C	6.4020000000	1.4891000000	-0.6137000000
C	6.9772000000	2.7563000000	-0.6249000000
C	6.3437000000	3.8287000000	0.0022000000
C	5.1276000000	3.6161000000	0.6508000000
C	4.5535000000	2.3487000000	0.6808000000
C	2.5322000000	0.5872000000	-1.1096000000
C	1.1654000000	0.4363000000	-1.3062000000
C	0.4116000000	-0.4895000000	-0.5683000000
C	1.0957000000	-1.2521000000	0.3921000000
C	2.4590000000	-1.0979000000	0.6076000000
C	5.2343000000	-2.3330000000	-0.4635000000
C	6.0306000000	-3.4505000000	-0.2312000000
C	7.0268000000	-3.4215000000	0.7443000000
C	7.2125000000	-2.2598000000	1.4930000000
C	6.4100000000	-1.1432000000	1.2785000000
H	-7.8504000000	-1.5922000000	2.0354000000

H	-5.5222000000	3.8263000000	1.7762000000
H	-6.5191000000	-3.5188000000	1.3762000000
H	-4.1294000000	-4.0379000000	-1.0776000000
H	-3.6178000000	-3.7428000000	0.5814000000
H	-0.9745000000	-2.7813000000	-0.5092000000
H	-8.0690000000	1.6635000000	0.8787000000
H	-7.1101000000	1.3188000000	2.3148000000
H	-3.2330000000	4.1302000000	1.0102000000
H	-1.0982000000	2.1092000000	0.2976000000
H	-1.2987000000	2.5854000000	-1.3818000000
H	6.8944000000	0.6628000000	-1.1119000000
H	7.9199000000	2.9075000000	-1.1393000000
H	6.7913000000	4.8156000000	-0.0139000000
H	4.6273000000	4.4385000000	1.1502000000
H	3.6144000000	2.1879000000	1.1964000000
H	3.0888000000	1.2950000000	-1.7118000000
H	0.6781000000	1.0208000000	-2.0783000000
H	0.5461000000	-1.9528000000	1.0103000000
H	2.9540000000	-1.6871000000	1.3701000000
H	4.4701000000	-2.3586000000	-1.2308000000
H	5.8797000000	-4.3438000000	-0.8273000000
H	7.6494000000	-4.2913000000	0.9181000000
H	7.9786000000	-2.2242000000	2.2598000000
H	6.5531000000	-0.2475000000	1.8707000000

Table S12. Atomic coordinates for the DFT optimized (B3LYP/6-311++G(d,p)) structure of sumanene **9**.

	x	y	z
C	5.2291000000	1.6611000000	0.0049000000
C	3.3974000000	-2.5236000000	2.0697000000
C	4.3628000000	2.5358000000	-0.7230000000
C	3.1986000000	2.0593000000	-1.3303000000
C	3.0036000000	0.6758000000	-1.2975000000
C	1.8407000000	2.6976000000	-1.7310000000
C	4.0529000000	-1.7223000000	1.1313000000
C	-0.4984000000	1.4130000000	-1.2222000000
C	5.2964000000	-0.7940000000	1.1744000000
C	2.0108000000	-2.8413000000	1.9307000000
C	1.2643000000	-2.3616000000	0.8511000000
C	4.9395000000	0.3003000000	0.1328000000
C	3.8427000000	-0.1714000000	-0.5930000000
C	0.8491000000	1.5145000000	-1.5637000000
C	1.6167000000	0.3534000000	-1.4431000000
C	3.3170000000	-1.3657000000	-0.0018000000
C	-1.0321000000	0.2175000000	-0.6269000000
C	-0.2401000000	-2.0630000000	0.6212000000
C	1.9736000000	-1.6758000000	-0.1379000000
C	-0.1996000000	-0.8877000000	-0.3863000000
C	1.1057000000	-0.8004000000	-0.8711000000
N	-2.3789000000	0.2139000000	-0.1653000000
C	-2.8998000000	1.3455000000	0.5216000000
C	-3.2163000000	-0.8960000000	-0.4530000000
C	-4.1678000000	-1.3333000000	0.4812000000
C	-4.9873000000	-2.4207000000	0.1959000000
C	-4.8676000000	-3.1039000000	-1.0142000000
C	-3.9175000000	-2.6778000000	-1.9411000000
C	-3.1031000000	-1.5814000000	-1.6714000000
C	-4.1655000000	1.8578000000	0.2054000000
C	-4.6664000000	2.9637000000	0.8863000000
C	-3.9120000000	3.5873000000	1.8795000000
C	-2.6482000000	3.0858000000	2.1901000000
C	-2.1467000000	1.9711000000	1.5248000000
H	6.0451000000	2.1119000000	0.5609000000
H	3.8893000000	-2.8170000000	2.9917000000
H	4.5715000000	3.6001000000	-0.6774000000
H	1.8549000000	3.0779000000	-2.7592000000
H	1.5793000000	3.5368000000	-1.0819000000
H	-1.1548000000	2.2743000000	-1.2733000000
H	6.2153000000	-1.3290000000	0.9068000000

H	5.4502000000	-0.3700000000	2.1698000000
H	1.5287000000	-3.3571000000	2.7551000000
H	-0.7455000000	-1.7775000000	1.5473000000
H	-0.7813000000	-2.9286000000	0.2216000000
H	-4.2620000000	-0.8174000000	1.4289000000
H	-5.7156000000	-2.7431000000	0.9320000000
H	-5.5036000000	-3.9543000000	-1.2303000000
H	-3.8151000000	-3.1928000000	-2.8901000000
H	-2.3768000000	-1.2518000000	-2.4038000000
H	-4.7529000000	1.3852000000	-0.5724000000
H	-5.6473000000	3.3474000000	0.6280000000
H	-4.3029000000	4.4515000000	2.4037000000
H	-2.0523000000	3.5560000000	2.9647000000
H	-1.1671000000	1.5816000000	1.7749000000

Table S13. Atomic coordinates for the DFT optimized (ωB97X-D/6-31G/IEFPCM(H_2O)) structure of the **4**-Li^+ complex, arrangement 1 (concave).

	x	*y*	*z*
C	7.8815580000	-1.4035400000	1.6199450000
C	6.1320110000	3.2330970000	0.9391670000
C	7.0730630000	-2.5302710000	1.2643760000
C	6.0914590000	-2.4297830000	0.2730800000
C	6.0371190000	-1.2218940000	-0.4351170000
C	4.7690790000	-3.1915670000	-0.0157390000
C	6.8639070000	2.0748850000	0.6569330000
C	2.5138940000	-1.8294000000	-0.6599790000
C	7.9873790000	1.3030990000	1.4022460000
C	4.8254510000	3.4299310000	0.3902330000
C	4.2426030000	2.4708870000	-0.4451980000
C	7.7118410000	-0.1678900000	0.9864930000
C	6.8173000000	-0.1341290000	-0.0908920000
C	3.8847460000	-2.0864500000	-0.6561560000
C	4.7284110000	-1.0220960000	-0.9969340000
C	6.3113350000	1.1985750000	-0.2858120000
C	1.9844970000	-0.5027590000	-0.8598570000
C	2.7799170000	2.0946670000	-0.7957620000
C	5.0499980000	1.3898300000	-0.8201270000
C	2.8796690000	0.5679000000	-1.0719290000
C	4.2387370000	0.2571730000	-1.1888740000
C	0.5354810000	-0.2596160000	-0.6938740000
C	-0.2534600000	-0.9938550000	0.2160050000
C	-1.6089320000	-0.7472190000	0.3656380000
C	-2.2876210000	0.2501350000	-0.3889370000
C	-1.4785330000	0.9846890000	-1.2989840000
C	-0.1203210000	0.7393700000	-1.4411190000
C	-3.7206950000	0.4655820000	-0.2480380000
C	-4.5995270000	-0.5986200000	0.1459810000
C	-4.2680250000	1.8064220000	-0.5330040000
C	-5.7734780000	-0.3209340000	0.9862060000
C	-4.3551820000	-1.9802030000	-0.2909680000
C	-3.7401050000	-2.2568790000	-1.5379690000
C	-3.4745430000	-3.5585210000	-1.9535370000
C	-3.8175800000	-4.6499400000	-1.1465590000
C	-4.4306410000	-4.4057760000	0.0875350000
C	-4.6896690000	-3.1027570000	0.5075990000
C	-3.5757500000	2.9950570000	-0.2018240000
C	-4.1173040000	4.2536340000	-0.4575990000
C	-5.3796600000	4.3786940000	-1.0476140000
C	-6.0866210000	3.2176650000	-1.3797260000

C	-5.5405160000	1.9604930000	-1.1315570000
C	-5.7487130000	0.7105440000	1.9575200000
C	-6.8620570000	1.0114070000	2.7378610000
C	-8.0519930000	0.2893580000	2.5915340000
C	-8.1016590000	-0.7394640000	1.6440170000
C	-6.9905970000	-1.0352140000	0.8571910000
H	8.5347330000	-1.5009330000	2.4814830000
H	6.4819170000	3.9437070000	1.6815070000
H	7.1549920000	-3.4241250000	1.8750110000
H	4.3211150000	-3.5842880000	0.9016350000
H	4.9224500000	-4.0406910000	-0.6933460000
H	1.8107780000	-2.6147270000	-0.4004430000
H	7.9159510000	1.4353140000	2.4857910000
H	8.9874780000	1.6366210000	1.0985780000
H	4.2502930000	4.2797000000	0.7445860000
H	2.4120580000	2.6562010000	-1.6632180000
H	2.0934870000	2.3013210000	0.0317800000
H	0.2128150000	-1.7469840000	0.8455450000
H	-2.1754550000	-1.3189110000	1.0924800000
H	-1.9419390000	1.7430780000	-1.9214170000
H	0.4440640000	1.3069920000	-2.1757910000
H	-3.4752260000	-1.4226970000	-2.1791080000
H	-3.0057990000	-3.7259980000	-2.9191880000
H	-3.6126410000	-5.6646190000	-1.4714800000
H	-4.6981680000	-5.2377090000	0.7328870000
H	-5.1477930000	-2.9398020000	1.4780060000
H	-2.6035410000	2.9207890000	0.2755970000
H	-3.5570530000	5.1429650000	-0.1837020000
H	-5.8031600000	5.3583120000	-1.2428990000
H	-7.0655890000	3.2943100000	-1.8440930000
H	-6.0966330000	1.0679240000	-1.3990210000
H	-4.8307040000	1.2734740000	2.0900130000
H	-6.7996500000	1.8080270000	3.4738710000
H	-8.9188040000	0.5217720000	3.2012660000
H	-9.0180520000	-1.3069300000	1.5088360000
H	-7.0613120000	-1.8236330000	0.1145830000
Li	4.2239150000	0.0585380000	2.2149990000

Table S14. Atomic coordinates for the DFT optimized (ωB97X-D/6-31G/IEFPCM(H_2O)) structure of the **4**-Li^+ complex, arrangement 2 (convex).

	x	*y*	*z*
C	-7.8624940000	-1.4086040000	-1.7528790000
C	-6.1157500000	3.2410700000	-1.0436930000
C	-7.0552730000	-2.5362560000	-1.3963520000
C	-6.0695580000	-2.4362710000	-0.4085920000
C	-6.0144690000	-1.2268430000	0.2955470000
C	-4.7443570000	-3.1960210000	-0.1179920000
C	-6.8444590000	2.0758980000	-0.7809620000
C	-2.4926690000	-1.8351380000	0.5621750000
C	-7.9610130000	1.3031340000	-1.5385750000
C	-4.8129150000	3.4378640000	-0.4854110000
C	-4.2275590000	2.4718160000	0.3405700000
C	-7.6900480000	-0.1705890000	-1.1239700000
C	-6.7940110000	-0.1387590000	-0.0486970000
C	-3.8634850000	-2.0932770000	0.5349350000
C	-4.7092480000	-1.0277630000	0.8635810000
C	-6.2908490000	1.1930890000	0.1542740000
C	-1.9661890000	-0.5080880000	0.7706260000
C	-2.7649380000	2.0935390000	0.6962770000
C	-5.0331150000	1.3836910000	0.6963460000
C	-2.8634940000	0.5642040000	0.9674940000
C	-4.2228290000	0.2511040000	1.0634110000
C	-0.5144900000	-0.2659730000	0.6281010000
C	0.2884940000	-1.0004350000	-0.2689150000
C	1.6459930000	-0.7533090000	-0.3976750000
C	2.3119940000	0.2463610000	0.3647140000
C	1.4891070000	0.9809280000	1.2619860000
C	0.1291340000	0.7342590000	1.3841510000
C	3.7466250000	0.4653890000	0.2424410000
C	4.6333550000	-0.5971560000	-0.1383060000
C	4.2861300000	1.8087350000	0.5292050000
C	5.8152740000	-0.3176680000	-0.9668240000
C	4.3882520000	-1.9781680000	0.2991320000
C	3.7567680000	-2.2537140000	1.5382820000
C	3.4904350000	-3.5551380000	1.9539820000
C	3.8487900000	-4.6476880000	1.1551820000
C	4.4780750000	-4.4046950000	-0.0709950000
C	4.7380030000	-3.1019750000	-0.4913120000
C	3.5935250000	2.9941470000	0.1867470000
C	4.1281170000	4.2552860000	0.4443460000
C	5.3834980000	4.3865780000	1.0478500000
C	6.0907300000	3.2289580000	1.3911750000

C	5.5516530000	1.9691660000	1.1409910000
C	5.7961920000	0.7097980000	-1.9425090000
C	6.9162580000	1.0123350000	-2.7124990000
C	8.1077850000	0.2961960000	-2.5506890000
C	8.1521280000	-0.7283350000	-1.5982950000
C	7.0341790000	-1.0257430000	-0.8218440000
H	-8.5152680000	-1.5073450000	-2.6145070000
H	-6.4629980000	3.9580890000	-1.7810280000
H	-7.1392600000	-3.4297150000	-2.0071840000
H	-4.2912140000	-3.5768790000	-1.0379410000
H	-4.8969320000	-4.0528230000	0.5498780000
H	-1.7845700000	-2.6191670000	0.3125340000
H	-7.8750000000	1.4359970000	-2.6210630000
H	-8.9648370000	1.6366480000	-1.2478520000
H	-4.2403530000	4.2936200000	-0.8292410000
H	-2.3985200000	2.6536160000	1.5651300000
H	-2.0775130000	2.3011220000	-0.1302960000
H	-0.1686350000	-1.7517390000	-0.9069990000
H	2.2242860000	-1.3250440000	-1.1152290000
H	1.9423580000	1.7412600000	1.8895680000
H	-0.4467640000	1.3025280000	2.1093760000
H	3.4799060000	-1.4187130000	2.1732810000
H	3.0089110000	-3.7215730000	2.9135370000
H	3.6431580000	-5.6621970000	1.4802080000
H	4.7575560000	-5.2374430000	-0.7102080000
H	5.2087160000	-2.9402430000	-1.4558670000
H	2.6270150000	2.9151500000	-0.3013040000
H	3.5679840000	5.1418190000	0.1612460000
H	5.8015790000	5.3682540000	1.2445230000
H	7.0644100000	3.3103650000	1.8657790000
H	6.1079900000	1.0793380000	1.4170560000
H	4.8770090000	1.2679140000	-2.0867910000
H	6.8579680000	1.8055010000	-3.4525670000
H	8.9798490000	0.5298420000	-3.1524260000
H	9.0695920000	-1.2910940000	-1.4511430000
H	7.1006780000	-1.8106800000	-0.0751650000
Li	-6.7936000000	0.0759860000	3.3851610000

Table S15. Atomic coordinates for the DFT optimized (ωB97X-D/6-31G/IEFPCM(H_2O)) structure of the **4**-Li^+ complex, arrangement 3 (convex).

	x	*y*	*z*
C	7.9010580000	-1.3224720000	1.6503680000
C	6.0903980000	3.3151550000	1.0092690000
C	7.0972810000	-2.4564030000	1.3030960000
C	6.0900030000	-2.3592590000	0.3351940000
C	6.0070940000	-1.1446970000	-0.3580080000
C	4.7640820000	-3.1280380000	0.0696660000
C	6.8252320000	2.1584080000	0.7252580000
C	2.4864120000	-1.7861680000	-0.5661170000
C	7.9648150000	1.3922960000	1.4547550000
C	4.7758020000	3.5027730000	0.4765560000
C	4.1839930000	2.5356050000	-0.3441730000
C	7.7032820000	-0.0813710000	1.0322450000
C	6.7830020000	-0.0506250000	-0.0229990000
C	3.8607960000	-2.0301210000	-0.5605540000
C	4.6897290000	-0.9550080000	-0.8981870000
C	6.2626090000	1.2770320000	-0.2050000000
C	1.9443270000	-0.4640920000	-0.7620540000
C	2.7190940000	2.1456220000	-0.6784030000
C	4.9931090000	1.4578160000	-0.7204000000
C	2.8284380000	0.6190660000	-0.9615460000
C	4.1881120000	0.3201400000	-1.0798900000
C	0.4909010000	-0.2367220000	-0.6138570000
C	-0.3033860000	-0.9744320000	0.2878880000
C	-1.6638810000	-0.7415250000	0.4153740000
C	-2.3414590000	0.2443920000	-0.3551760000
C	-1.5264230000	0.9823740000	-1.2570930000
C	-0.1637770000	0.7514260000	-1.3762800000
C	-3.7787670000	0.4482820000	-0.2394670000
C	-4.6566510000	-0.6176650000	0.1512890000
C	-4.3312130000	1.7815780000	-0.5507000000
C	-5.8534020000	-0.3387410000	0.9578360000
C	-4.3929140000	-2.0032260000	-0.2614210000
C	-3.7525470000	-2.2926360000	-1.4925230000
C	-3.4711370000	-3.5981490000	-1.8850240000
C	-3.8224880000	-4.6805730000	-1.0695650000
C	-4.4600640000	-4.4236180000	0.1494730000
C	-4.7350530000	-3.1166770000	0.5464350000
C	-3.6567830000	2.9791670000	-0.2151270000
C	-4.2020450000	4.2305820000	-0.4966380000
C	-5.4505930000	4.3390700000	-1.1185420000
C	-6.1400880000	3.1689480000	-1.4552740000

C	-5.5901050000	1.9188560000	-1.1808990000
C	-5.8673480000	0.7128170000	1.9079000000
C	-7.0048100000	1.0151050000	2.6520660000
C	-8.1815150000	0.2749270000	2.4897890000
C	-8.1927720000	-0.7746730000	1.5638870000
C	-7.0576380000	-1.0720320000	0.8129930000
H	8.5699760000	-1.4190620000	2.4997400000
H	6.4450930000	4.0314840000	1.7437280000
H	7.2003200000	-3.3517450000	1.9081630000
H	4.3342200000	-3.5147060000	0.9983780000
H	4.9085430000	-3.9819460000	-0.6034830000
H	1.7891050000	-2.5784910000	-0.3118350000
H	7.8973940000	1.5136960000	2.5399140000
H	8.9590050000	1.7403440000	1.1488150000
H	4.2022240000	4.3518360000	0.8350280000
H	2.3334530000	2.7078460000	-1.5374650000
H	2.0431820000	2.3409450000	0.1605500000
H	0.1627320000	-1.7172640000	0.9295790000
H	-2.2353200000	-1.3149510000	1.1370530000
H	-1.9880770000	1.7322680000	-1.8909570000
H	0.4062070000	1.3212880000	-2.1049400000
H	-3.4806860000	-1.4652230000	-2.1395380000
H	-2.9831320000	-3.7759120000	-2.8392400000
H	-3.6051270000	-5.6982720000	-1.3764850000
H	-4.7342610000	-5.2483560000	0.8012270000
H	-5.2127280000	-2.9426870000	1.5055060000
H	-2.6957360000	2.9174310000	0.2860610000
H	-3.6557680000	5.1272420000	-0.2185000000
H	-5.8770760000	5.3131000000	-1.3343060000
H	-7.1081440000	3.2327780000	-1.9438450000
H	-6.1326980000	1.0190240000	-1.4517890000
H	-4.9604080000	1.2903670000	2.0529100000
H	-6.9718640000	1.8276990000	3.3723880000
H	-9.0671170000	0.5089080000	3.0712790000
H	-9.0978390000	-1.3573240000	1.4172660000
H	-7.0968210000	-1.8780660000	0.0869800000
Li	9.1116960000	-2.0891170000	-1.4469020000

Table S16. Atomic coordinates for the DFT optimized (ωB97X-D/6-31G/IEFPCM(H_2O)) structure of **4**.

	x	*y*	*z*
C	7.9843210000	-1.3001080000	1.6051940000
C	6.1888750000	3.2825990000	0.6190650000
C	7.1719030000	-2.4534430000	1.3633090000
C	6.1486300000	-2.4286020000	0.4090090000
C	6.0612390000	-1.2745410000	-0.3783500000
C	4.8180890000	-3.2119190000	0.2250190000
C	6.9127190000	2.1016780000	0.4186900000
C	2.5344320000	-1.9101560000	-0.4679850000
C	8.0601260000	1.3895050000	1.1894490000
C	4.8658850000	3.4353140000	0.0957740000
C	4.2551130000	2.4083410000	-0.6328200000
C	7.7811290000	-0.1114260000	0.8948110000
C	6.8454030000	-0.1612050000	-0.1447700000
C	3.9074820000	-2.1624850000	-0.4723550000
C	4.7331630000	-1.1190090000	-0.9039860000
C	6.3294390000	1.1506200000	-0.4265140000
C	2.0036370000	-0.6036690000	-0.7536490000
C	2.7813180000	2.0012490000	-0.9050510000
C	5.0522300000	1.2983570000	-0.9332900000
C	2.8799230000	0.4557320000	-1.0581800000
C	4.2362910000	0.1397850000	-1.1808250000
C	0.5506500000	-0.3523630000	-0.5741440000
C	-0.1780760000	-0.9481360000	0.4683450000
C	-1.5379110000	-0.6965610000	0.6243750000
C	-2.2182020000	0.1605010000	-0.2536750000
C	-1.4864050000	0.7777190000	-1.2788030000
C	-0.1284970000	0.5180600000	-1.4419020000
C	-3.6722960000	0.4488310000	-0.0820990000
C	-4.5967720000	-0.5334950000	0.0799060000
C	-4.0339340000	1.8980800000	-0.1028610000
C	-6.0095350000	-0.2500410000	0.4730130000
C	-4.2764570000	-1.9785240000	-0.1211980000
C	-3.6376420000	-2.4217640000	-1.2894490000
C	-3.3539920000	-3.7748190000	-1.4751440000
C	-3.7073890000	-4.7071480000	-0.4963140000
C	-4.3563010000	-4.2785520000	0.6646930000
C	-4.6462870000	-2.9261530000	0.8459900000
C	-3.3226970000	2.8158850000	0.6859470000
C	-3.6472430000	4.1724390000	0.6669030000
C	-4.6775730000	4.6355340000	-0.1556940000
C	-5.3781520000	3.7334260000	-0.9604360000

C	-5.0578380000	2.3760200000	-0.9349800000
C	-6.3026550000	0.5551890000	1.5842550000
C	-7.6246650000	0.8014240000	1.9551130000
C	-8.6751140000	0.2439760000	1.2216450000
C	-8.3944100000	-0.5700750000	0.1210410000
C	-7.0719080000	-0.8221210000	-0.2442040000
H	8.6702110000	-1.3335790000	2.4458810000
H	6.5600900000	4.0542270000	1.2862360000
H	7.2850540000	-3.2998640000	2.0334450000
H	4.4018940000	-3.5262430000	1.1865760000
H	4.9500910000	-4.1140530000	-0.3848130000
H	1.8368390000	-2.6736410000	-0.1375000000
H	8.0135770000	1.6019390000	2.2616230000
H	9.0507790000	1.7025650000	0.8374220000
H	4.3032220000	4.3144650000	0.3936950000
H	2.3820030000	2.4862230000	-1.8039270000
H	2.1236860000	2.2741260000	-0.0736050000
H	0.3270340000	-1.5921120000	1.1804420000
H	-2.0809320000	-1.1627970000	1.4391240000
H	-1.9896260000	1.4559400000	-1.9601770000
H	0.4105210000	0.9809430000	-2.2619080000
H	-3.3611420000	-1.6995250000	-2.0500430000
H	-2.8612760000	-4.1014350000	-2.3845450000
H	-3.4851200000	-5.7589070000	-0.6397770000
H	-4.6376660000	-4.9969100000	1.4270760000
H	-5.1575440000	-2.5971260000	1.7451040000
H	-2.5164060000	2.4604940000	1.3197220000
H	-3.0959540000	4.8672060000	1.2912820000
H	-4.9277260000	5.6906400000	-0.1742110000
H	-6.1708400000	4.0870480000	-1.6108610000
H	-5.6036330000	1.6774990000	-1.5600290000
H	-5.4888060000	0.9896210000	2.1547310000
H	-7.8344060000	1.4242120000	2.8180170000
H	-9.7029990000	0.4368470000	1.5087960000
H	-9.2044870000	-1.0097480000	-0.4507340000
H	-6.8567220000	-1.4617440000	-1.0942740000

Table S17. Atomic coordinates for the DFT optimized (ωB97X-D/LANL2DZ/IEFPCM(H_2O)) structure of the **5**-Cs^+ complex, arrangement 1 (concave).

	x	*y*	*z*
C	-8.6511100000	-0.6374430000	-1.3854460000
C	-6.6486070000	2.8674430000	1.5817270000
C	-7.8634330000	-1.7835620000	-1.7623250000
C	-6.8320150000	-2.2643290000	-0.9425990000
C	-6.6703800000	-1.6495510000	0.3225840000
C	-5.5421120000	-3.0867000000	-1.2152310000
C	-7.4155150000	1.7365130000	1.2493890000
C	-3.1920570000	-2.2713640000	-0.1866510000
C	-8.6159000000	1.5008450000	0.2918180000
C	-5.2955270000	2.7278390000	2.0442350000
C	-4.7045760000	1.4581070000	2.1743660000
C	-8.3855620000	0.0373240000	-0.1793960000
C	-7.4367790000	-0.5394680000	0.6822050000
C	-4.5555740000	-2.5127220000	-0.1582990000
C	-5.3470510000	-1.8448660000	0.8351120000
C	-6.8455240000	0.4803500000	1.5227150000
C	-2.5448900000	-1.2967750000	0.7222730000
C	-3.2407390000	0.9464800000	2.1192360000
C	-5.5380280000	0.3435790000	1.9690530000
C	-3.4069950000	-0.4963800000	1.5708750000
C	-4.7537890000	-0.8254940000	1.6168620000
C	-1.1757440000	-1.0291710000	0.6063740000
C	0.0343290000	-0.7672460000	0.5012150000
C	1.4115180000	-0.4629340000	0.3881760000
C	2.2528790000	-1.1527960000	-0.5340320000
C	3.6091230000	-0.8487570000	-0.6337860000
C	4.2035350000	0.1520310000	0.1687770000
C	3.3675660000	0.8567240000	1.0645180000
C	2.0110000000	0.5570710000	1.1846810000
C	5.6521990000	0.4802760000	0.0562050000
C	6.6259420000	-0.4782300000	-0.0057930000
C	5.9819870000	1.9413330000	0.0158850000
C	8.0352970000	-0.1629550000	-0.3984150000
C	6.3592070000	-1.9143230000	0.3209250000
C	5.2929890000	2.8062270000	-0.8567250000
C	5.5947900000	4.1751180000	-0.8954160000
C	6.5805210000	4.7043750000	-0.0451190000
C	7.2569050000	3.8535460000	0.8451690000
C	6.9585120000	2.4841720000	0.8742230000
C	8.3120790000	0.5863080000	-1.5597780000
C	9.6331570000	0.8710980000	-1.9319100000

C	10.7032190000	0.4074750000	-1.1482530000
C	10.4389290000	-0.3532650000	0.0033710000
C	9.1162550000	-0.6422180000	0.3683410000
C	5.6901510000	-2.2702490000	1.5095320000
C	5.4564390000	-3.6153420000	1.8253800000
C	5.8911230000	-4.6310180000	0.9565430000
C	6.5720620000	-4.2877510000	-0.2233740000
C	6.8106740000	-2.9405780000	-0.5323540000
H	-9.3490710000	-0.2331590000	-2.1116390000
H	-7.0131310000	3.8675760000	1.3688770000
H	-8.0046040000	-2.1866230000	-2.7612260000
H	-5.1776230000	-2.9587020000	-2.2385200000
H	-5.7135290000	-4.1595860000	-1.0596540000
H	-2.5691920000	-2.6966440000	-0.9699440000
H	-8.6037220000	2.2010350000	-0.5483360000
H	-9.5784240000	1.6198420000	0.8033010000
H	-4.7019960000	3.6297880000	2.1578000000
H	-2.7534160000	0.9720080000	3.1020260000
H	-2.6297930000	1.5596810000	1.4468440000
H	1.8224960000	-1.9211210000	-1.1686480000
H	4.2231370000	-1.3872540000	-1.3490810000
H	3.7936720000	1.6424240000	1.6821980000
H	1.3952950000	1.1049660000	1.8912000000
H	4.5260710000	2.4024870000	-1.5108170000
H	5.0641980000	4.8257760000	-1.5827100000
H	6.8123820000	5.7639370000	-0.0708530000
H	8.0100000000	4.2557790000	1.5147200000
H	7.4817360000	1.8302990000	1.5646040000
H	7.4890870000	0.9434000000	-2.1704350000
H	9.8277660000	1.4479150000	-2.8300860000
H	11.7261670000	0.6289650000	-1.4338710000
H	11.2589800000	-0.7194250000	0.6122410000
H	8.9189530000	-1.2342640000	1.2570060000
H	5.3536090000	-1.4891260000	2.1835130000
H	4.9412440000	-3.8714880000	2.7453090000
H	5.7084350000	-5.6726750000	1.1987440000
H	6.9157910000	-5.0650910000	-0.8978520000
H	7.3417990000	-2.6821980000	-1.4435910000
Cs	-4.8665310000	0.9150730000	-1.6029510000

Table S18. Atomic coordinates for the DFT optimized (ωB97X-D/LANL2DZ/IEFPCM(H_2O)) structure of the **5**-Cs^+ complex, arrangement 2 (convex).

	x	*y*	*z*
C	8.1750000000	0.7994530000	2.9943330000
C	6.2835700000	3.7933840000	-0.6025240000
C	7.3941810000	-0.3154710000	3.4739690000
C	6.4156440000	-0.9246020000	2.6733240000
C	6.3203550000	-0.4731240000	1.3407650000
C	5.1314480000	-1.7587710000	2.9566030000
C	7.0484310000	2.7624240000	-0.0259260000
C	2.8418710000	-1.2533780000	1.6017090000
C	8.1852010000	2.7069450000	1.0372200000
C	4.9732660000	3.5325430000	-1.1377370000
C	4.4179450000	2.2398450000	-1.0994320000
C	7.9623430000	1.3149680000	1.7020470000
C	7.0772150000	0.6010240000	0.8810220000
C	4.2139320000	-1.3889730000	1.7513180000
C	5.0530400000	-0.7960360000	0.7612870000
C	6.5231340000	1.4662580000	-0.1328480000
C	2.2417620000	-0.4442370000	0.5124040000
C	2.9712730000	1.6672470000	-1.0908170000
C	5.2615590000	1.2139800000	-0.6478680000
C	3.1389570000	0.3185980000	-0.3340640000
C	4.4941080000	0.0753910000	-0.1949540000
C	0.8526970000	-0.3013630000	0.4303240000
C	-0.3820180000	-0.1605960000	0.3531190000
C	-1.7800680000	-0.0026150000	0.2554970000
C	-2.6729190000	-0.6715330000	1.1501700000
C	-4.0517820000	-0.5164350000	1.0392680000
C	-4.6295590000	0.3085120000	0.0438620000
C	-3.7487710000	0.9958250000	-0.8253850000
C	-2.3667590000	0.8433950000	-0.7366710000
C	-6.1028000000	0.4747270000	-0.0714730000
C	-6.9807460000	-0.5716040000	0.0281890000
C	-6.5802900000	1.8756080000	-0.3083600000
C	-8.4526990000	-0.3665160000	0.2032230000
C	-6.5383820000	-1.9996700000	-0.0402620000
C	-6.1127560000	2.9361900000	0.4921130000
C	-6.5542280000	4.2489940000	0.2726880000
C	-7.4588360000	4.5243890000	-0.7669060000
C	-7.9129660000	3.4767100000	-1.5856210000
C	-7.4758650000	2.1644650000	-1.3569310000
C	-8.9499080000	0.5108010000	1.1884280000
C	-10.3293910000	0.6943110000	1.3565390000

C	-11.2390970000	-0.0014810000	0.5426070000
C	-10.7549970000	-0.8907060000	-0.4319860000
C	-9.3744950000	-1.0762500000	-0.5927530000
C	-5.6880050000	-2.4415870000	-1.0743950000
C	-5.2900920000	-3.7828680000	-1.1522090000
C	-5.7388360000	-4.7100780000	-0.1958100000
C	-6.5980910000	-4.2835470000	0.8305250000
C	-6.9997480000	-2.9414340000	0.9008680000
H	8.8262430000	1.3058720000	3.7003680000
H	6.6074410000	4.8281450000	-0.5404930000
H	7.4992150000	-0.5878690000	4.5207400000
H	4.6764330000	-1.4961670000	3.9158460000
H	5.3490370000	-2.8344670000	2.9815100000
H	2.1573190000	-1.6337370000	2.3563980000
H	8.0930690000	3.5180930000	1.7649600000
H	9.1791310000	2.7894640000	0.5808920000
H	4.3777800000	4.3849640000	-1.4514740000
H	2.5655880000	1.5412250000	-2.1028810000
H	2.2881600000	2.3355400000	-0.5546330000
H	-2.2608500000	-1.3040660000	1.9304200000
H	-4.7013560000	-1.0336630000	1.7389190000
H	-4.1607130000	1.6496710000	-1.5893890000
H	-1.7177700000	1.3732100000	-1.4273040000
H	-5.4086290000	2.7282340000	1.2920540000
H	-6.1946820000	5.0527140000	0.9067630000
H	-7.7985950000	5.5400590000	-0.9405080000
H	-8.6011040000	3.6821390000	-2.3989930000
H	-7.8275550000	1.3578730000	-1.9923200000
H	-8.2517510000	1.0474470000	1.8227480000
H	-10.6935860000	1.3728440000	2.1209690000
H	-12.3070230000	0.1409520000	0.6703890000
H	-11.4497230000	-1.4361450000	-1.0622690000
H	-9.0075030000	-1.7677080000	-1.3453800000
H	-5.3394710000	-1.7291050000	-1.8150710000
H	-4.6365900000	-4.1055110000	-1.9561320000
H	-5.4295180000	-5.7483890000	-0.2537310000
H	-6.9536890000	-4.9926200000	1.5709310000
H	-7.6676900000	-2.6195810000	1.6943080000
Cs	7.1183890000	-1.7126930000	-2.0427010000

Table S19. Atomic coordinates for the DFT optimized (ωB97X-D/LANL2DZ/IEFPCM(H_2O)) structure of the **5**-Cs^+ complex, arrangement 3 (convex).

	x	*y*	*z*
C	7.9323900000	0.6259390000	2.2223320000
C	5.7187060000	4.1646140000	-0.6177920000
C	7.2214050000	-0.5953880000	2.5169620000
C	6.2117920000	-1.0784070000	1.6687630000
C	6.0194660000	-0.3853710000	0.4541440000
C	4.9808420000	-2.0126680000	1.8489810000
C	6.5541200000	3.0779880000	-0.2982630000
C	2.5968030000	-1.3615120000	0.7422380000
C	7.7523890000	2.8739240000	0.6756880000
C	4.3890250000	3.9493770000	-1.1230020000
C	3.8862030000	2.6481670000	-1.3129360000
C	7.6142330000	1.3748280000	1.0721450000
C	6.7082670000	0.7918480000	0.1736490000
C	3.9804470000	-1.4600800000	0.7883080000
C	4.7349500000	-0.6501430000	-0.1096470000
C	6.0720630000	1.8035110000	-0.6323200000
C	1.9067440000	-0.3827210000	-0.1300400000
C	2.4628600000	2.0206550000	-1.3359770000
C	4.7927130000	1.5937240000	-1.1223080000
C	2.7242860000	0.5615980000	-0.8660160000
C	4.0943950000	0.3555460000	-0.8565100000
C	0.5121560000	-0.2792170000	-0.0989050000
C	-0.7286960000	-0.1696980000	-0.0731290000
C	-2.1306680000	-0.0404870000	-0.0492220000
C	-2.9501460000	-0.8971020000	0.7539750000
C	-4.3349290000	-0.7646760000	0.7653320000
C	-4.9962790000	0.2207180000	-0.0098350000
C	-4.1877130000	1.0887630000	-0.7846960000
C	-2.8013630000	0.9637050000	-0.8181090000
C	-6.4746240000	0.3645570000	0.0064370000
C	-7.3313810000	-0.7048920000	-0.0216320000
C	-6.9892570000	1.7718220000	0.0533550000
C	-8.7880570000	-0.5701400000	0.2924340000
C	-6.8798960000	-2.0851430000	-0.3820810000
C	-6.4877640000	2.6874000000	0.9990060000
C	-6.9642600000	4.0054960000	1.0440950000
C	-7.9396810000	4.4346350000	0.1279000000
C	-8.4293840000	3.5353370000	-0.8341630000
C	-7.9566010000	2.2159740000	-0.8695680000
C	-9.2210190000	0.1104870000	1.4489300000
C	-10.5860910000	0.2284670000	1.7448170000

C	-11.5461430000	-0.3378060000	0.8893400000
C	-11.1257790000	-1.0325370000	-0.2576510000
C	-9.7591220000	-1.1532540000	-0.5469650000
C	-6.1038510000	-2.3094920000	-1.5378310000
C	-5.6987220000	-3.6036370000	-1.8904120000
C	-6.0656290000	-4.7014480000	-1.0926690000
C	-6.8506810000	-4.4912470000	0.0531970000
C	-7.2600180000	-3.1948520000	0.3988900000
H	8.6158940000	1.0108450000	2.9723850000
H	6.0074550000	5.1815070000	-0.3687000000
H	7.4080270000	-1.0652820000	3.4783080000
H	4.5757900000	-1.9625920000	2.8635000000
H	5.2408450000	-3.0606560000	1.6512610000
H	1.9732980000	-1.9121080000	1.4427390000
H	7.6769980000	3.5275080000	1.5491670000
H	8.7151750000	3.0825210000	0.1935790000
H	3.7427060000	4.8159550000	-1.2282130000
H	1.9987660000	2.0728500000	-2.3292550000
H	1.7923250000	2.5419160000	-0.6433930000
H	-2.4744710000	-1.6554670000	1.3684760000
H	-4.9245730000	-1.4260390000	1.3930310000
H	-4.6622490000	1.8654950000	-1.3784980000
H	-2.2113870000	1.6364350000	-1.4333060000
H	-5.7293790000	2.3611950000	1.7042480000
H	-6.5771570000	4.6945690000	1.7875160000
H	-8.3067070000	5.4551770000	0.1588770000
H	-9.1728680000	3.8616510000	-1.5539500000
H	-8.3360040000	1.5242460000	-1.6150000000
H	-8.4838890000	0.5454330000	2.1161610000
H	-10.8997960000	0.7550710000	2.6402110000
H	-12.6031260000	-0.2459150000	1.1162220000
H	-11.8592100000	-1.4773830000	-0.9222220000
H	-9.4417340000	-1.6941430000	-1.4335120000
H	-5.8183270000	-1.4645010000	-2.1561260000
H	-5.1031910000	-3.7573470000	-2.7844110000
H	-5.7505900000	-5.7037510000	-1.3634220000
H	-7.1428520000	-5.3325300000	0.6731800000
H	-7.8704480000	-3.0404490000	1.2836850000
Cs	9.2718130000	-1.6432220000	-0.5017820000

Table S20. Atomic coordinates for the DFT optimized (ωB97X-D/LANL2DZ/IEFPCM(H_2O)) structure of **5**.

	x	*y*	*z*
C	9.4833100000	-0.3144160000	1.9472850000
C	7.4759520000	3.1832910000	-1.0859440000
C	8.7112270000	-1.4700560000	2.3243640000
C	7.6601220000	-1.9360350000	1.5147500000
C	7.5085870000	-1.3000690000	0.2726530000
C	6.3611240000	-2.7550910000	1.7739600000
C	8.2446060000	2.0694740000	-0.7025490000
C	4.0026520000	-2.0230120000	0.6165540000
C	9.4365020000	1.8489560000	0.2753740000
C	6.1303360000	3.0261740000	-1.5734770000
C	5.5425420000	1.7533060000	-1.6828970000
C	9.2110900000	0.3855320000	0.7579960000
C	8.2508970000	-0.1870570000	-0.0902750000
C	5.3902380000	-2.1979530000	0.6921100000
C	6.1648260000	-1.4558030000	-0.2151170000
C	7.6779890000	0.8105850000	-0.9554340000
C	3.4189310000	-1.0211590000	-0.2534930000
C	4.0785920000	1.2223350000	-1.6636330000
C	6.3833790000	0.6594670000	-1.4267000000
C	4.2450090000	-0.1946790000	-1.0490530000
C	5.6118170000	-0.4952130000	-1.0464700000
C	2.0051410000	-0.7768150000	-0.1775490000
C	0.8044730000	-0.5544690000	-0.1262030000
C	-0.6070630000	-0.2908030000	-0.0690490000
C	-1.4531060000	-1.0723710000	0.7478440000
C	-2.8250590000	-0.8133500000	0.7956010000
C	-3.3933880000	0.2273170000	0.0341530000
C	-2.5425100000	1.0198970000	-0.7610230000
C	-1.1702310000	0.7623270000	-0.8229110000
C	-4.8591870000	0.5162220000	0.1001590000
C	-5.8009550000	-0.4598640000	-0.0443060000
C	-5.2171240000	1.9507800000	0.3342850000
C	-7.2460010000	-0.2321330000	0.2714770000
C	-5.4598470000	-1.8335650000	-0.5311780000
C	-4.5949930000	2.6821120000	1.3649860000
C	-4.9216630000	4.0278970000	1.5851770000
C	-5.8646060000	4.6681540000	0.7635560000
C	-6.4735030000	3.9525430000	-0.2810510000
C	-6.1507610000	2.6052180000	-0.4933790000
C	-7.6344360000	0.3365380000	1.5007010000
C	-8.9892200000	0.5339020000	1.8009050000

C	-9.9786350000	0.1637940000	0.8745700000
C	-9.6014310000	-0.4166680000	-0.3481640000
C	-8.2455650000	-0.6211500000	-0.6415830000
C	-4.7061980000	-2.0146270000	-1.7077440000
C	-4.3975470000	-3.3020310000	-2.1681840000
C	-4.8400830000	-4.4304670000	-1.4578480000
C	-5.6049760000	-4.2598110000	-0.2915240000
C	-5.9196450000	-2.9706930000	0.1614440000
H	10.1919640000	0.0756300000	2.6714350000
H	7.8292300000	4.1927770000	-0.8992900000
H	8.8795610000	-1.8880870000	3.3120430000
H	5.9757790000	-2.5887850000	2.7833470000
H	6.5258550000	-3.8327190000	1.6595560000
H	3.3337580000	-2.5368920000	1.2996780000
H	9.4066450000	2.5552340000	1.1093940000
H	10.4050270000	1.9723220000	-0.2231670000
H	5.5418070000	3.9253850000	-1.7272020000
H	3.6364250000	1.1865540000	-2.6661370000
H	3.4298590000	1.8496920000	-1.0453590000
H	-1.0306170000	-1.8749280000	1.3425210000
H	-3.4637520000	-1.4184910000	1.4299480000
H	-2.9605780000	1.8363180000	-1.3411640000
H	-0.5296180000	1.3726410000	-1.4502470000
H	-3.8620540000	2.1939240000	2.0002910000
H	-4.4432740000	4.5744390000	2.3910380000
H	-6.1155080000	5.7103430000	0.9303120000
H	-7.1930270000	4.4429480000	-0.9281990000
H	-6.6211250000	2.0567070000	-1.3030670000
H	-6.8734400000	0.6210760000	2.2201080000
H	-9.2724110000	0.9706470000	2.7527880000
H	-11.0274410000	0.3186030000	1.1047380000
H	-10.3593100000	-0.7096760000	-1.0670220000
H	-7.9599050000	-1.0757740000	-1.5853750000
H	-4.3658500000	-1.1457040000	-2.2617200000
H	-3.8180470000	-3.4249720000	-3.0770530000
H	-4.5992190000	-5.4273610000	-1.8115370000
H	-5.9549280000	-5.1260490000	0.2599770000
H	-6.5154930000	-2.8445580000	1.0603600000

Table S21. Atomic coordinates for the DFT optimized (ωB97X-D/LANL2DZ/IEFPCM(H_2O)) structure of **4**.

	x	*y*	*z*
C	8.0355170000	-1.2866690000	1.6159680000
C	6.2450260000	3.3061650000	0.5428320000
C	7.2099100000	-2.4475410000	1.4047270000
C	6.1666810000	-2.4342530000	0.4618480000
C	6.0762110000	-1.2888380000	-0.3445900000
C	4.8289960000	-3.2157690000	0.3014440000
C	6.9611130000	2.1082770000	0.3692890000
C	2.5363590000	-1.9133770000	-0.4028900000
C	8.1160080000	1.4050910000	1.1422360000
C	4.9101740000	3.4553870000	0.0245680000
C	4.2815020000	2.4078240000	-0.6717020000
C	7.8246810000	-0.1031820000	0.8858380000
C	6.8704010000	-0.1715220000	-0.1410960000
C	3.9143920000	-2.1734570000	-0.4060440000
C	4.7425560000	-1.1372180000	-0.8629870000
C	6.3576870000	1.1385600000	-0.4467560000
C	2.0064010000	-0.6053360000	-0.7142800000
C	2.8022110000	2.0013690000	-0.9301690000
C	5.0727970000	1.2824490000	-0.9469000000
C	2.8892350000	0.4489660000	-1.0437130000
C	4.2472660000	0.1215390000	-1.1627990000
C	0.5493860000	-0.3472080000	-0.5397390000
C	-0.1947510000	-0.9516850000	0.4947570000
C	-1.5617520000	-0.6967030000	0.6401010000
C	-2.2350350000	0.1714010000	-0.2412850000
C	-1.4887400000	0.7984310000	-1.2565150000
C	-0.1230760000	0.5366350000	-1.4083480000
C	-3.6944780000	0.4549070000	-0.0801260000
C	-4.6171560000	-0.5382730000	0.0721750000
C	-4.0706800000	1.9037640000	-0.0984930000
C	-6.0285200000	-0.2636530000	0.4880830000
C	-4.2881550000	-1.9793670000	-0.1642860000
C	-3.6439480000	-2.3872350000	-1.3491960000
C	-3.3473360000	-3.7386140000	-1.5734610000
C	-3.6930480000	-4.7057010000	-0.6146330000
C	-4.3492150000	-4.3104840000	0.5632950000
C	-4.6518340000	-2.9586270000	0.7807150000
C	-3.3665230000	2.8338260000	0.6911330000
C	-3.7105060000	4.1931310000	0.6739560000
C	-4.7547750000	4.6459710000	-0.1498880000
C	-5.4474860000	3.7289020000	-0.9581120000

C	-5.1066490000	2.3696190000	-0.9321550000
C	-6.3055520000	0.5348680000	1.6153980000
C	-7.6276730000	0.7786040000	2.0119770000
C	-8.6957310000	0.2247910000	1.2863420000
C	-8.4290230000	-0.5844970000	0.1690700000
C	-7.1049450000	-0.8333210000	-0.2198080000
H	8.7356400000	-1.3107460000	2.4454870000
H	6.6287780000	4.0953040000	1.1822190000
H	7.3323640000	-3.2835960000	2.0865260000
H	4.4209440000	-3.5166280000	1.2700690000
H	4.9542300000	-4.1252220000	-0.2977440000
H	1.8385510000	-2.6702120000	-0.0569770000
H	8.0878850000	1.6433460000	2.2089440000
H	9.1021130000	1.7025620000	0.7666580000
H	4.3586820000	4.3487660000	0.3011020000
H	2.4094830000	2.4634300000	-1.8432610000
H	2.1458330000	2.3032590000	-0.1083890000
H	0.2988820000	-1.6041810000	1.2077910000
H	-2.1123910000	-1.1665750000	1.4483630000
H	-1.9823590000	1.4821030000	-1.9401290000
H	0.4228600000	1.0062600000	-2.2204960000
H	-3.3774440000	-1.6436750000	-2.0933680000
H	-2.8523260000	-4.0367270000	-2.4916870000
H	-3.4615500000	-5.7516740000	-0.7858520000
H	-4.6242770000	-5.0514200000	1.3065250000
H	-5.1635890000	-2.6595540000	1.6904870000
H	-2.5549830000	2.4904590000	1.3256270000
H	-3.1670990000	4.8952730000	1.2974600000
H	-5.0194960000	5.6979090000	-0.1671910000
H	-6.2463310000	4.0720520000	-1.6070720000
H	-5.6423480000	1.6649520000	-1.5599610000
H	-5.4834220000	0.9621520000	2.1803740000
H	-7.8243650000	1.3939340000	2.8836110000
H	-9.7195310000	0.4148750000	1.5907700000
H	-9.2477840000	-1.0199630000	-0.3940080000
H	-6.9044980000	-1.4641780000	-1.0805560000

Table S22. Atomic coordinates for the DFT optimized (ωB97X-D/LANL2DZ/IEFPCM(H_2O)) structure of the **4**-Cs^+ complex, arrangement 1 (concave).

	x	*y*	*z*
C	-7.3217720000	1.3833220000	1.0093460000
C	-5.5311820000	-3.2490420000	0.3086100000
C	-6.5150000000	2.5252710000	0.6679470000
C	-5.5197840000	2.4401370000	-0.3201230000
C	-5.4496190000	1.2321840000	-1.0381420000
C	-4.1992400000	3.2147510000	-0.5900620000
C	-6.2704850000	-2.0882080000	0.0251590000
C	-1.9190720000	1.8626740000	-1.1767620000
C	-7.4068470000	-1.3283390000	0.7651230000
C	-4.2109520000	-3.4353980000	-0.2308000000
C	-3.6229650000	-2.4630400000	-1.0576450000
C	-7.1371930000	0.1488670000	0.3638760000
C	-6.2280120000	0.1321250000	-0.7088450000
C	-3.2952040000	2.1172990000	-1.2179360000
C	-4.1285380000	1.0446970000	-1.5857380000
C	-5.7092210000	-1.1990530000	-0.9089200000
C	-1.3751970000	0.5331980000	-1.3687820000
C	-2.1571330000	-2.0735580000	-1.3849720000
C	-4.4363420000	-1.3792320000	-1.4329740000
C	-2.2602810000	-0.5410570000	-1.6353550000
C	-3.6242450000	-0.2343260000	-1.7800060000
C	0.0615140000	0.2867240000	-1.1134050000
C	0.7906210000	1.0185160000	-0.1441870000
C	2.1376160000	0.7649900000	0.0991670000
C	2.8655700000	-0.2394780000	-0.6108460000
C	2.1141200000	-0.9743970000	-1.5782320000
C	0.7647710000	-0.7212670000	-1.8155990000
C	4.2878510000	-0.4617610000	-0.3749120000
C	5.1447450000	0.6010920000	0.0805480000
C	4.8520440000	-1.8077820000	-0.6292190000
C	6.2719910000	0.3153940000	0.9869670000
C	4.9275630000	1.9894030000	-0.3660070000
C	4.3975100000	2.2729460000	-1.6555120000
C	4.1619930000	3.5836310000	-2.0857300000
C	4.4501090000	4.6770980000	-1.2469270000
C	4.9776880000	4.4231620000	0.0332100000
C	5.2090830000	3.1106210000	0.4632860000
C	4.1364390000	-2.9992560000	-0.3391770000
C	4.6932440000	-4.2653930000	-0.5614720000
C	5.9954580000	-4.3935310000	-1.0790910000
C	6.7246790000	-3.2263120000	-1.3718090000

C	6.1619720000	-1.9624890000	-1.1535880000
C	6.1928150000	-0.7285130000	1.9500020000
C	7.2656620000	-1.0377030000	2.7944460000
C	8.4689530000	-0.3112890000	2.7203490000
C	8.5701440000	0.7313240000	1.7797220000
C	7.4976130000	1.0356140000	0.9325930000
H	-7.9792620000	1.4671440000	1.8685560000
H	-5.8823330000	-3.9635860000	1.0459720000
H	-6.6054770000	3.4115160000	1.2877230000
H	-3.7686780000	3.6167350000	0.3309070000
H	-4.3535340000	4.0564630000	-1.2747770000
H	-1.2323260000	2.6473100000	-0.8759560000
H	-7.3469710000	-1.4692060000	1.8476020000
H	-8.3995380000	-1.6645240000	0.4446680000
H	-3.6322690000	-4.2804810000	0.1277090000
H	-1.7841900000	-2.6125130000	-2.2632220000
H	-1.4752440000	-2.2984020000	-0.5588160000
H	0.2848820000	1.7698620000	0.4572820000
H	2.6538870000	1.3325080000	0.8668520000
H	2.6150360000	-1.7363140000	-2.1673670000
H	0.2509960000	-1.2880830000	-2.5878210000
H	4.1765730000	1.4418180000	-2.3185550000
H	3.7611390000	3.7563810000	-3.0807320000
H	4.2688460000	5.6940900000	-1.5797910000
H	5.2001420000	5.2513780000	0.7003680000
H	5.5993250000	2.9429220000	1.4628830000
H	3.1375470000	-2.9251430000	0.0812080000
H	4.1167050000	-5.1540960000	-0.3207930000
H	6.4290360000	-5.3739270000	-1.2485720000
H	7.7292110000	-3.3034730000	-1.7781360000
H	6.7339910000	-1.0703080000	-1.3905610000
H	5.2685330000	-1.2929400000	2.0283160000
H	7.1624480000	-1.8405700000	3.5191790000
H	9.3011780000	-0.5489780000	3.3751870000
H	9.4922830000	1.3003810000	1.7005890000
H	7.6093730000	1.8318320000	0.2024560000
Cs	-3.4936770000	-0.0299180000	1.9198580000

Table S23. Atomic coordinates for the DFT optimized (ωB97X-D/LANL2DZ/IEFPCM(H_2O)) structure of the **4**-Cs^+ complex, arrangement 2 (convex).

	x	*y*	*z*
C	-6.6845120000	1.3821130000	3.0583120000
C	-5.0455810000	-3.2874080000	2.0396060000
C	-5.8968080000	2.5222150000	2.6617410000
C	-4.9880880000	2.4430810000	1.5917740000
C	-5.0010570000	1.2439370000	0.8632480000
C	-3.6876920000	3.2041250000	1.1932220000
C	-5.7793150000	-2.0961620000	1.8981320000
C	-1.5120620000	1.8459030000	0.2495930000
C	-6.8216500000	-1.3374490000	2.7731640000
C	-3.7935550000	-3.4769910000	1.3529740000
C	-3.2632330000	-2.4772680000	0.5182540000
C	-6.5704050000	0.1511610000	2.3879420000
C	-5.7589290000	0.1484640000	1.2437420000
C	-2.8757230000	2.1129380000	0.4333920000
C	-3.7557830000	1.0534350000	0.1698200000
C	-5.2883630000	-1.1807260000	0.9552770000
C	-1.0162560000	0.5165850000	-0.0521460000
C	-1.8372600000	-2.0968140000	0.0266860000
C	-4.0842730000	-1.3631620000	0.2936720000
C	-1.9395020000	-0.5532690000	-0.1763940000
C	-3.2991700000	-0.2247550000	-0.1113150000
C	0.4454140000	0.2697280000	-0.0876770000
C	1.3627070000	1.0123870000	0.6950810000
C	2.7320700000	0.7605740000	0.6510710000
C	3.2955690000	-0.2529950000	-0.1824320000
C	2.3594110000	-0.9947310000	-0.9637920000
C	0.9892820000	-0.7426750000	-0.9143000000
C	4.7379850000	-0.4780870000	-0.2430780000
C	5.6681080000	0.5931520000	0.0029450000
C	5.2359070000	-1.8318540000	-0.5744310000
C	6.9557180000	0.3311670000	0.6696350000
C	5.3572200000	1.9703210000	-0.4234440000
C	4.5844940000	2.2217630000	-1.5910770000
C	4.2590270000	3.5213930000	-1.9961230000
C	4.6947260000	4.6350660000	-1.2531670000
C	5.4627340000	4.4128700000	-0.0944860000
C	5.7842010000	3.1115440000	0.3108850000
C	4.5826200000	-3.0128790000	-0.1311490000
C	5.0815920000	-4.2873750000	-0.4290010000
C	6.2622970000	-4.4368200000	-1.1801460000
C	6.9283890000	-3.2811300000	-1.6282130000

C	6.4230890000	-2.0087640000	-1.3336560000
C	7.0847640000	-0.7007150000	1.6410640000
C	8.3081210000	-0.9859430000	2.2586320000
C	9.4630630000	-0.2470590000	1.9394540000
C	9.3605550000	0.7838100000	0.9858850000
C	8.1372600000	1.0638200000	0.3649470000
H	-7.2683650000	1.4669460000	3.9696540000
H	-5.3382840000	-4.0371390000	2.7682260000
H	-5.9318130000	3.4020890000	3.2968530000
H	-3.1487170000	3.5647940000	2.0733170000
H	-3.8996290000	4.0736700000	0.5600740000
H	-0.7771400000	2.6226710000	0.4376990000
H	-6.6501680000	-1.5094840000	3.8390800000
H	-7.8486990000	-1.6509120000	2.5522870000
H	-3.2118690000	-4.3591030000	1.6023200000
H	-1.5770310000	-2.6245110000	-0.8981590000
H	-1.0696560000	-2.3468750000	0.7655260000
H	0.9970490000	1.7731510000	1.3797920000
H	3.3972950000	1.3378600000	1.2853180000
H	2.7259180000	-1.7622380000	-1.6386000000
H	0.3274390000	-1.3154810000	-1.5586010000
H	4.2471970000	1.3752260000	-2.1815910000
H	3.6716310000	3.6695620000	-2.8981370000
H	4.4437640000	5.6433900000	-1.5665900000
H	5.8028130000	5.2571200000	0.4989100000
H	6.3619680000	2.9681810000	1.2193200000
H	3.6821200000	-2.9234610000	0.4695250000
H	4.5565330000	-5.1663720000	-0.0657660000
H	6.6516510000	-5.4238740000	-1.4083260000
H	7.8380230000	-3.3740980000	-2.2149070000
H	6.9449180000	-1.1265110000	-1.6923090000
H	6.2025720000	-1.2741460000	1.9089700000
H	8.3616850000	-1.7799550000	2.9984260000
H	10.4118120000	-0.4662570000	2.4187860000
H	10.2403910000	1.3627620000	0.7189450000
H	8.0909420000	1.8516500000	-0.3812470000
Cs	-6.1486730000	0.0187680000	-2.4989740000

Table S24. Atomic coordinates for the DFT optimized (ωB97X-D/LANL2DZ/IEFPCM(H_2O)) structure of the **4**-Cs^+ complex, arrangement 3 (convex).

	x	*y*	*z*
C	6.5772910000	-0.2249580000	2.4183220000
C	4.4619210000	4.1651240000	1.0838730000
C	5.8751840000	-1.4610370000	2.1790160000
C	4.8991840000	-1.5591990000	1.1702580000
C	4.7643090000	-0.4411340000	0.3316610000
C	3.6588280000	-2.4659110000	0.9203410000
C	5.2943490000	3.0343060000	1.0009950000
C	1.3224030000	-1.3985500000	-0.0072200000
C	6.4464630000	2.4569010000	1.8771090000
C	3.1633870000	4.1775400000	0.4622800000
C	2.6860450000	3.0611410000	-0.2482590000
C	6.3073100000	0.9232330000	1.6503230000
C	5.4391870000	0.7467490000	0.5616960000
C	2.7134350000	-1.5279630000	0.1116910000
C	3.4784120000	-0.4300020000	-0.3081380000
C	4.8424100000	1.9957710000	0.1729290000
C	0.6941360000	-0.1525700000	-0.3990170000
C	1.2755950000	2.5145860000	-0.6107850000
C	3.5938670000	2.0073810000	-0.4287240000
C	1.5094960000	0.9738710000	-0.6818240000
C	2.8957280000	0.7716530000	-0.6794480000
C	-0.7840560000	-0.0347080000	-0.3659530000
C	-1.5863090000	-0.7644550000	0.5446840000
C	-2.9737530000	-0.6388820000	0.5589360000
C	-3.6714610000	0.2235530000	-0.3403750000
C	-2.8499910000	0.9561910000	-1.2490770000
C	-1.4613420000	0.8318450000	-1.2570170000
C	-5.1298310000	0.3115460000	-0.3380880000
C	-5.9441720000	-0.7988280000	0.0828370000
C	-5.7701460000	1.5677110000	-0.7880620000
C	-7.2132450000	-0.5651730000	0.7960650000
C	-5.5338180000	-2.1860480000	-0.1993680000
C	-4.7859340000	-2.5097870000	-1.3659010000
C	-4.3629170000	-3.8166360000	-1.6339800000
C	-4.6722140000	-4.8672760000	-0.7493010000
C	-5.4143090000	-4.5739100000	0.4105880000
C	-5.8331000000	-3.2649550000	0.6791780000
C	-5.2165280000	2.8463930000	-0.5123790000
C	-5.8501350000	4.0271590000	-0.9203170000
C	-7.0709110000	3.9803570000	-1.6189960000
C	-7.6396510000	2.7246890000	-1.9018450000

C	-7.0000850000	1.5463450000	-1.4975400000
C	-7.3714060000	0.5489220000	1.6666670000
C	-8.5773800000	0.8030310000	2.3306680000
C	-9.6836830000	-0.0504630000	2.1603860000
C	-9.5522350000	-1.1612730000	1.3054060000
C	-8.3465930000	-1.4107080000	0.6381840000
H	7.2242510000	-0.1741380000	3.2881030000
H	4.7221780000	5.0018140000	1.7249150000
H	6.0329340000	-2.2727640000	2.8818690000
H	3.2014430000	-2.7860150000	1.8602080000
H	3.9205220000	-3.3701770000	0.3584240000
H	0.6726480000	-2.2123690000	0.3001530000
H	6.3198810000	2.7179510000	2.9310980000
H	7.4282790000	2.8298660000	1.5627940000
H	2.5144110000	5.0230560000	0.6690980000
H	0.9156390000	2.9281160000	-1.5599810000
H	0.5311270000	2.7655970000	0.1511690000
H	-1.1158890000	-1.4119760000	1.2801010000
H	-3.5489830000	-1.1990420000	1.2892520000
H	-3.3206330000	1.6111510000	-1.9759540000
H	-0.8887760000	1.3878990000	-1.9948020000
H	-4.5458560000	-1.7144410000	-2.0650060000
H	-3.7981390000	-4.0210270000	-2.5394730000
H	-4.3454390000	-5.8813120000	-0.9564570000
H	-5.6579280000	-5.3674740000	1.1116830000
H	-6.3874300000	-3.0641280000	1.5913090000
H	-4.2863060000	2.9089450000	0.0448760000
H	-5.3984380000	4.9865580000	-0.6841330000
H	-7.5642090000	4.8948300000	-1.9326010000
H	-8.5781600000	2.6659940000	-2.4459410000
H	-7.4473990000	0.5842880000	-1.7292780000
H	-6.5249860000	1.2118760000	1.8187840000
H	-8.6540430000	1.6624660000	2.9910220000
H	-10.6185700000	0.1439520000	2.6762710000
H	-10.3961850000	-1.8281280000	1.1518450000
H	-8.2793140000	-2.2637150000	-0.0306620000
Cs	8.0409270000	-1.2928560000	-0.9789740000

Table S25. Atomic coordinates for the DFT optimized (ωB97X-D/6-31G/IEFPCM(H_2O)) structure of **5**.

	x	*y*	*z*
C	9.4335430000	-0.2946270000	1.9357630000
C	7.4058250000	3.1852340000	-1.0606800000
C	8.6767530000	-1.4527900000	2.3023800000
C	7.6431130000	-1.9274120000	1.4872240000
C	7.4895980000	-1.2951440000	0.2477840000
C	6.3530110000	-2.7562750000	1.7440370000
C	8.1847310000	2.0809950000	-0.6959820000
C	3.9998740000	-2.0399060000	0.5932290000
C	9.3734550000	1.8681820000	0.2833230000
C	6.0705560000	3.0192800000	-1.5479440000
C	5.5018500000	1.7469570000	-1.6758010000
C	9.1639600000	0.4003650000	0.7511330000
C	8.2195720000	-0.1759900000	-0.1058100000
C	5.3821250000	-2.2070320000	0.6614330000
C	6.1497930000	-1.4602670000	-0.2416380000
C	7.6389600000	0.8203660000	-0.9639110000
C	3.4092110000	-1.0431160000	-0.2680770000
C	4.0450620000	1.2047280000	-1.6601300000
C	6.3503710000	0.6603160000	-1.4360880000
C	4.2261280000	-0.2142450000	-1.0616740000
C	5.5913900000	-0.5041500000	-1.0673960000
C	2.0019090000	-0.8026490000	-0.1843120000
C	0.8081920000	-0.5752380000	-0.1275120000
C	-0.5956610000	-0.3083400000	-0.0649960000
C	-1.4387960000	-1.0862450000	0.7492920000
C	-2.8033770000	-0.8249370000	0.8016230000
C	-3.3687940000	0.2144660000	0.0467040000
C	-2.5207520000	1.0033720000	-0.7464260000
C	-1.1563090000	0.7438150000	-0.8121950000
C	-4.8299240000	0.5104920000	0.1123250000
C	-5.7766480000	-0.4524750000	-0.0359160000
C	-5.1707110000	1.9455110000	0.3495290000
C	-7.2231830000	-0.2072410000	0.2428030000
C	-5.4483100000	-1.8357640000	-0.4937880000
C	-4.5564570000	2.6550970000	1.3932570000
C	-4.8616200000	3.9978100000	1.6167340000
C	-5.7734250000	4.6569440000	0.7880160000
C	-6.3751590000	3.9651950000	-0.2662980000
C	-6.0750130000	2.6204660000	-0.4844180000
C	-7.6389690000	0.3724730000	1.4512500000
C	-8.9935840000	0.5815690000	1.7102370000

C	-9.9543600000	0.2123710000	0.7653580000
C	-9.5520900000	-0.3771130000	-0.4360630000
C	-8.1977500000	-0.5934390000	-0.6906460000
C	-4.6933780000	-2.0513250000	-1.6567940000
C	-4.4018730000	-3.3465280000	-2.0851470000
C	-4.8629050000	-4.4467720000	-1.3579660000
C	-5.6272740000	-4.2434760000	-0.2058320000
C	-5.9252910000	-2.9481040000	0.2171210000
H	10.1301130000	0.1045190000	2.6662900000
H	7.7462560000	4.1955040000	-0.8565990000
H	8.8395930000	-1.8699530000	3.2910690000
H	5.9630450000	-2.5890730000	2.7521470000
H	6.5250750000	-3.8338270000	1.6339790000
H	3.3368140000	-2.5562520000	1.2796390000
H	9.3282260000	2.5661240000	1.1244930000
H	10.3439300000	2.0108620000	-0.2075770000
H	5.4692980000	3.9121580000	-1.6869190000
H	3.5980910000	1.1777970000	-2.6614010000
H	3.3926060000	1.8183340000	-1.0310400000
H	-1.0162690000	-1.8907120000	1.3406140000
H	-3.4422090000	-1.4325190000	1.4325100000
H	-2.9387420000	1.8229040000	-1.3213460000
H	-0.5155410000	1.3529450000	-1.4397480000
H	-3.8421000000	2.1479250000	2.0340790000
H	-4.3874740000	4.5292180000	2.4347710000
H	-6.0080700000	5.7019280000	0.9585630000
H	-7.0747560000	4.4733970000	-0.9210540000
H	-6.5439700000	2.0850390000	-1.3029360000
H	-6.8948100000	0.6606460000	2.1858550000
H	-9.2989370000	1.0283040000	2.6502510000
H	-11.0074140000	0.3766600000	0.9659720000
H	-10.2923340000	-0.6700710000	-1.1726680000
H	-7.8887550000	-1.0595750000	-1.6208310000
H	-4.3343030000	-1.1985350000	-2.2229100000
H	-3.8189210000	-3.4965120000	-2.9874040000
H	-4.6343590000	-5.4537440000	-1.6895020000
H	-5.9922750000	-5.0928960000	0.3613100000
H	-6.5260090000	-2.7930360000	1.1077020000

Table S26. Atomic coordinates for the DFT optimized (ωB97X-D/6-31G/IEFPCM(H_2O)) structure of the **5**-Li^+ complex, arrangement 1 (concave).

	x	*y*	*z*
C	9.2825180000	-0.6087910000	2.0479670000
C	7.3783900000	3.2843620000	-0.4541660000
C	8.5190200000	-1.8117180000	2.1874220000
C	7.5421200000	-2.1590380000	1.2485150000
C	7.4492420000	-1.3448020000	0.1123640000
C	6.2522950000	-3.0230490000	1.3022260000
C	8.1535690000	2.1387610000	-0.2440580000
C	3.9542810000	-2.1265630000	0.1809300000
C	9.2952490000	1.7775640000	0.7457600000
C	6.0698720000	3.1941800000	-1.0259470000
C	5.5274510000	1.9582370000	-1.3940350000
C	9.0733170000	0.2562460000	0.9685290000
C	8.1862780000	-0.1831700000	-0.0220380000
C	5.3323270000	-2.3091070000	0.2738410000
C	6.1397950000	-1.4387030000	-0.4724300000
C	7.6395200000	0.9353830000	-0.7432420000
C	3.3910190000	-1.0057510000	-0.5385040000
C	4.0782830000	1.4197330000	-1.5368460000
C	6.3767760000	0.8483570000	-1.2998510000
C	4.2442140000	-0.0750400000	-1.1664450000
C	5.6105680000	-0.3633790000	-1.1614050000
C	1.9866390000	-0.7679330000	-0.4628060000
C	0.7886260000	-0.5374590000	-0.3955010000
C	-0.6031110000	-0.2700160000	-0.3106620000
C	-1.4891730000	-1.1718130000	0.3289730000
C	-2.8416080000	-0.9085740000	0.4155190000
C	-3.4253530000	0.2801630000	-0.1239520000
C	-2.5134530000	1.1786040000	-0.7601540000
C	-1.1601020000	0.9147340000	-0.8522020000
C	-4.8460260000	0.5319700000	-0.0471130000
C	-5.8058480000	-0.5241980000	0.0619950000
C	-5.3205940000	1.9375150000	-0.1052450000
C	-7.0554350000	-0.3187130000	0.8133180000
C	-5.5932900000	-1.8220110000	-0.5973300000
C	-4.6668700000	2.9815570000	0.5832220000
C	-5.1405280000	4.2923840000	0.5397590000
C	-6.2922040000	4.6053940000	-0.1891100000
C	-6.9578930000	3.5856940000	-0.8769370000
C	-6.4771810000	2.2779470000	-0.8380150000
C	-7.0852550000	0.4966850000	1.9699920000
C	-8.2659440000	0.7304850000	2.6711400000

C	-9.4685030000	0.1501440000	2.2533100000
C	-9.4639360000	-0.6685160000	1.1183800000
C	-8.2844650000	-0.8955000000	0.4122200000
C	-4.8893830000	-1.9157270000	-1.8226410000
C	-4.6687230000	-3.1392300000	-2.4487370000
C	-5.1466400000	-4.3268070000	-1.8821020000
C	-5.8486880000	-4.2613890000	-0.6738110000
C	-6.0647480000	-3.0371890000	-0.0436570000
H	9.9325540000	-0.3244060000	2.8694260000
H	7.6965780000	4.2478480000	-0.0685060000
H	8.6298000000	-2.3769240000	3.1075270000
H	5.8103370000	-3.0268750000	2.3026370000
H	6.4441390000	-4.0670900000	1.0256590000
H	3.2679160000	-2.7369910000	0.7589790000
H	9.2090170000	2.3382270000	1.6810160000
H	10.2863800000	1.9921010000	0.3273190000
H	5.4625790000	4.0936860000	-1.0438420000
H	3.6802710000	1.5556930000	-2.5501110000
H	3.3896610000	1.9233430000	-0.8507780000
H	-1.0883020000	-2.0801150000	0.7699830000
H	-3.4862670000	-1.6155300000	0.9251370000
H	-2.8993960000	2.0903270000	-1.2035310000
H	-0.5068950000	1.6213860000	-1.3562870000
H	-3.7827100000	2.7515350000	1.1697050000
H	-4.6168150000	5.0707440000	1.0865420000
H	-6.6643460000	5.6240710000	-0.2193420000
H	-7.8504730000	3.8131350000	-1.4520460000
H	-6.9987660000	1.4928080000	-1.3758860000
H	-6.1584400000	0.9448670000	2.3124480000
H	-8.2473950000	1.3605660000	3.5556570000
H	-10.3879070000	0.3295550000	2.8006440000
H	-10.3886800000	-1.1229800000	0.7749340000
H	-8.3077410000	-1.5188820000	-0.4760080000
H	-4.5186900000	-1.0029580000	-2.2771610000
H	-4.1289620000	-3.1681860000	-3.3908890000
H	-4.9759500000	-5.2801190000	-2.3710490000
H	-6.2216900000	-5.1720820000	-0.2142020000
H	-6.5986710000	-3.0122360000	0.9010620000
Li	5.5467990000	0.8946660000	2.0007710000

Table S27. Atomic coordinates for the DFT optimized (ωB97X-D/6-31G/IEFPCM(H_2O)) structure of the **5**-Li^+ complex, arrangement 2 (convex).

	x	*y*	*z*
C	9.3303940000	-0.5834250000	2.0888810000
C	7.3924840000	3.3345500000	-0.3777050000
C	8.5613550000	-1.7807180000	2.2460380000
C	7.5528590000	-2.1165920000	1.3363600000
C	7.4360870000	-1.2967370000	0.2069260000
C	6.2539820000	-2.9669950000	1.4230830000
C	8.1601940000	2.1830350000	-0.1704800000
C	3.9362240000	-2.0656680000	0.3322280000
C	9.3199550000	1.8123280000	0.7965770000
C	6.0716630000	3.2541430000	-0.9231630000
C	5.5059630000	2.0212180000	-1.2670460000
C	9.0970860000	0.2898660000	1.0205620000
C	8.1784500000	-0.1398850000	0.0555770000
C	5.3155120000	-2.2498790000	0.4115650000
C	6.1117250000	-1.3784280000	-0.3435260000
C	7.6218660000	0.9829240000	-0.6498230000
C	3.3654110000	-0.9435670000	-0.3799820000
C	4.0490210000	1.4906100000	-1.3779880000
C	6.3468950000	0.9053840000	-1.1781280000
C	4.2115570000	-0.0070800000	-1.0105260000
C	5.5758920000	-0.2992380000	-1.0205260000
C	1.9597560000	-0.7144180000	-0.3045400000
C	0.7596110000	-0.4920580000	-0.2521410000
C	-0.6353280000	-0.2335170000	-0.1930810000
C	-1.5287550000	-1.1412960000	0.4271250000
C	-2.8851180000	-0.8887860000	0.4808110000
C	-3.4653280000	0.2938310000	-0.0750850000
C	-2.5458900000	1.1995600000	-0.6892370000
C	-1.1885530000	0.9465690000	-0.7482390000
C	-4.8900320000	0.5323790000	-0.0351700000
C	-5.8416860000	-0.5336910000	0.0489060000
C	-5.3762220000	1.9330300000	-0.1061880000
C	-7.1102050000	-0.3427340000	0.7713590000
C	-5.6001490000	-1.8272820000	-0.6086740000
C	-4.7483310000	2.9842100000	0.5955700000
C	-5.2324380000	4.2906580000	0.5381820000
C	-6.3693150000	4.5921640000	-0.2183130000
C	-7.0095260000	3.5652930000	-0.9195800000
C	-6.5182730000	2.2619710000	-0.8667110000
C	-7.1750780000	0.4706170000	1.9281300000
C	-8.3737300000	0.6909080000	2.6026240000

C	-9.5603400000	0.0985190000	2.1569780000
C	-9.5212510000	-0.7183720000	1.0214260000
C	-8.3237830000	-0.9318390000	0.3418300000
C	-4.8670160000	-1.9098000000	-1.8176260000
C	-4.6196030000	-3.1288880000	-2.4423360000
C	-5.0982470000	-4.3231960000	-1.8905900000
C	-5.8281570000	-4.2689850000	-0.6983600000
C	-6.0712480000	-3.0491540000	-0.0695530000
H	10.0039730000	-0.3073830000	2.8940170000
H	7.7267360000	4.2980240000	-0.0059940000
H	8.6931100000	-2.3479540000	3.1620750000
H	5.8350610000	-2.9560530000	2.4334340000
H	6.4273780000	-4.0156580000	1.1523580000
H	3.2546010000	-2.6766860000	0.9151460000
H	9.2500560000	2.3687070000	1.7358720000
H	10.3044200000	2.0266560000	0.3628510000
H	5.4749320000	4.1607150000	-0.9354030000
H	3.6278030000	1.6309480000	-2.3811230000
H	3.3812570000	1.9981370000	-0.6743450000
H	-1.1312470000	-2.0458170000	0.8787930000
H	-3.5364060000	-1.5999840000	0.9759560000
H	-2.9285800000	2.1077620000	-1.1425790000
H	-0.5290250000	1.6580100000	-1.2371060000
H	-3.8761610000	2.7631550000	1.2030890000
H	-4.7286250000	5.0746420000	1.0955430000
H	-6.7495540000	5.6074650000	-0.2595010000
H	-7.8901810000	3.7837460000	-1.5161860000
H	-7.0197240000	1.4712820000	-1.4155300000
H	-6.2610270000	0.9274690000	2.2926930000
H	-8.3818040000	1.3197770000	3.4881660000
H	-10.4937650000	0.2673660000	2.6835440000
H	-10.4330780000	-1.1821990000	0.6565680000
H	-8.3203280000	-1.5544040000	-0.5472870000
H	-4.4953880000	-0.9916170000	-2.2604460000
H	-4.0580930000	-3.1492430000	-3.3719390000
H	-4.9066600000	-5.2730840000	-2.3784830000
H	-6.2019180000	-5.1850010000	-0.2500680000
H	-6.6264240000	-3.0330110000	0.8630160000
Li	8.0688760000	-1.3183340000	-3.1985450000

Table S28. Atomic coordinates for the DFT optimized (ωB97X-D/6-31G/IEFPCM(H_2O)) structure of the **5**-Li^+ complex, arrangement 3 (convex).

	x	*y*	*z*
C	9.2721430000	-0.7679890000	1.9894040000
C	7.3220180000	3.4319170000	0.0558300000
C	8.5176230000	-1.9851100000	1.9640640000
C	7.5296360000	-2.2027730000	0.9962110000
C	7.4194380000	-1.2349590000	-0.0107990000
C	6.2399920000	-3.0697730000	0.9429870000
C	8.1014590000	2.2709780000	0.1139330000
C	3.9289500000	-2.0492570000	-0.0476960000
C	9.2497210000	1.7831740000	1.0412460000
C	6.0118210000	3.4134570000	-0.5187940000
C	5.4687510000	2.2337570000	-1.0405810000
C	9.0453730000	0.2418460000	1.0461080000
C	8.1482020000	-0.0598300000	0.0137760000
C	5.3092770000	-2.2287550000	0.0245870000
C	6.1057790000	-1.2543510000	-0.5915790000
C	7.5880430000	1.1440450000	-0.5382550000
C	3.3547070000	-0.8457370000	-0.6065160000
C	4.0211490000	1.7080950000	-1.2494030000
C	6.3228520000	1.1262110000	-1.0937940000
C	4.1982520000	0.1761030000	-1.0911700000
C	5.5661420000	-0.0971980000	-1.1201690000
C	1.9462640000	-0.6399840000	-0.5150940000
C	0.7441450000	-0.4353950000	-0.4390110000
C	-0.6527810000	-0.1978160000	-0.3492560000
C	-1.5108590000	-1.0919840000	0.3373720000
C	-2.8686160000	-0.8581800000	0.4258670000
C	-3.4874180000	0.2889000000	-0.1629280000
C	-2.6032660000	1.1800540000	-0.8470220000
C	-1.2440110000	0.9476730000	-0.9367920000
C	-4.9133550000	0.5077910000	-0.0858450000
C	-5.8461180000	-0.5627680000	0.0965170000
C	-5.4255030000	1.8949230000	-0.2218500000
C	-7.0903260000	-0.3419130000	0.8526700000
C	-5.6099770000	-1.8929600000	-0.4852790000
C	-4.7973590000	2.9937500000	0.4018920000
C	-5.3056300000	4.2869530000	0.2844500000
C	-6.4674350000	4.5266300000	-0.4561130000
C	-7.1082790000	3.4516610000	-1.0804470000
C	-6.5929490000	2.1614300000	-0.9677180000
C	-7.1237990000	0.5392440000	1.9600980000
C	-8.2996560000	0.7850150000	2.6652440000

C	-9.4930510000	0.1515300000	2.3018500000
C	-9.4846890000	-0.7313590000	1.2161460000
C	-8.3103060000	-0.9705720000	0.5054870000
C	-4.9176320000	-2.0453360000	-1.7113640000
C	-4.6725790000	-3.2995480000	-2.2631560000
C	-5.1136950000	-4.4610470000	-1.6181730000
C	-5.8045920000	-4.3379560000	-0.4079230000
C	-6.0449240000	-3.0827430000	0.1478800000
H	9.9248000000	-0.5964220000	2.8395030000
H	7.6376450000	4.3376650000	0.5639650000
H	8.6382050000	-2.6707650000	2.7968120000
H	5.8058320000	-3.2020780000	1.9381730000
H	6.4299700000	-4.0692640000	0.5333720000
H	3.2475680000	-2.7416420000	0.4361190000
H	9.1555880000	2.2011580000	2.0478040000
H	10.2382250000	2.0679160000	0.6606750000
H	5.4028270000	4.3060440000	-0.4158920000
H	3.6139670000	1.9820680000	-2.2305170000
H	3.3354910000	2.1049500000	-0.4937650000
H	-1.0837200000	-1.9694050000	0.8147470000
H	-3.4905510000	-1.5566970000	0.9738310000
H	-3.0152000000	2.0599820000	-1.3293390000
H	-0.6129380000	1.6476560000	-1.4770660000
H	-3.9051420000	2.8207450000	0.9958240000
H	-4.8009130000	5.1093550000	0.7824630000
H	-6.8662880000	5.5317820000	-0.5439830000
H	-8.0082930000	3.6218540000	-1.6637110000
H	-7.0949830000	1.3326430000	-1.4565640000
H	-6.2036540000	1.0290360000	2.2607800000
H	-8.2840470000	1.4661500000	3.5111270000
H	-10.4084720000	0.3401030000	2.8527730000
H	-10.4025940000	-1.2275470000	0.9147220000
H	-8.3306850000	-1.6456450000	-0.3442240000
H	-4.5755260000	-1.1537480000	-2.2262980000
H	-4.1424470000	-3.3736840000	-3.2082900000
H	-4.9237510000	-5.4384480000	-2.0489840000
H	-6.1493050000	-5.2272230000	0.1118060000
H	-6.5685120000	-3.0126130000	1.0960730000
Li	10.5664380000	-2.3576600000	-0.7510900000

S7. Supporting references

(1) Sakurai, H.; Daiko, T.; Hirao, T. A Synthesis of Sumanene, a Fullerene Fragment. *Science* **2003**, *301* (5641), 1878–1878. https://doi.org/10.1126/science.1088290.

(2) Kasprzak, A.; Gajda-Walczak, A.; Kowalczyk, A.; Wagner, B.; Nowicka, A. M.; Nishimoto, M.; Koszytkowska-Stawińska, M.; Sakurai, H. Application of Monoferrocenylsumanenes Derived from Sonogashira Cross-Coupling or *Click Chemistry* Reactions in Highly Sensitive and Selective Cesium Cation Electrochemical Sensors. *J. Org. Chem.* **2023**, *88*, 4199–4208. https://doi.org/10.1021/acs.joc.2c02767.

(3) Shrestha, B. B.; Higashibayashi, S.; Sakurai, H. Columnar/Herringbone Dual Crystal Packing of Pyrenylsumanene and Its Photophysical Properties. *Beilstein J. Org. Chem.* **2014**, *10*, 841–847. https://doi.org/10.3762/bjoc.10.80.

(4) Guo, R.; Ye, S.; Wang, Y.; Duan, Y.; Di, K.; Wang, L. Exploiting Asymmetric Anthracene-Based Multifunctional Materials Based on a “Bulky Peripheral Modification” Strategy for Constructing Simplified Efficient Deep-Blue Fluorescent OLEDs. *J. Mater. Chem. C* **2021**, *9* (38), 13392–13401. https://doi.org/10.1039/D1TC02081A.

(5) Jiang, W.; Duan, L.; Qiao, J.; Zhang, D.; Dong, G.; Wang, L.; Qiu, Y. Novel Star-Shaped Host Materials for Highly Efficient Solution-Processed Phosphorescent Organic Light-Emitting Diodes. *J. Mater. Chem.* **2010**, *20* (29), 6131. https://doi.org/10.1039/c0jm00692k.

(6) Brouwer, A. M. Standards for Photoluminescence Quantum Yield Measurements in Solution (IUPAC Technical Report). *Pure Appl. Chem.* **2011**, *83* (12), 2213–2228. https://doi.org/10.1351/PAC-REP-10-09-31.

(7) Würth, C.; Grabolle, M.; Pauli, J.; Spieles, M.; Resch-Genger, U. Relative and Absolute Determination of Fluorescence Quantum Yields of Transparent Samples. *Nat. Protoc.* **2013**, *8* (8), 1535–1550. https://doi.org/10.1038/nprot.2013.087.

(8) Brouwer, A. M. Standards for Photoluminescence Quantum Yield Measurements in Solution (IUPAC Technical Report). *Pure Appl. Chem.* **2011**, *83* (12), 2213–2228. https://doi.org/10.1351/PAC-REP-10-09-31.

(9) http://supramolecular.org/.

(10) Thordarson, P. Determining Association Constants from Titration Experiments in Supramolecular Chemistry. *Chem. Soc. Rev.* **2011**, *40* (3), 1305–1323. https://doi.org/10.1039/C0CS00062K.

(11) Brynn Hibbert, D.; Thordarson, P. The Death of the Job Plot, Transparency, Open Science and Online Tools, Uncertainty Estimation Methods and Other Developments in Supramolecular Chemistry Data Analysis. *Chem. Commun.* **2016**, *52* (87), 12792–12805. https://doi.org/10.1039/C6CC03888C.

(12) Frisch, M. J.; Trucks, G. W.; Schlegel, H. B.; Scuseria, G. E.; Robb, M. A.; Cheeseman, J. R.; Scalmani, G.; Barone, V.; Petersson, G. A.; Nakatsuji, H.; Li, X.; Caricato, M.; Marenich, A. V.; Bloino, J.; Janesko, B. G.; Gomperts, R.; Mennucci, B.; Hratchian, H. P.; Ortiz, J. V.; Izmaylov, A. F.; Sonnenberg, L.; Williams-Young, D.; Ding, F.; Lipparini, F.; Egidi, F.; Goings, J.; Peng, B.; Petrone, A.; Henderson, T.; Ranasinghe, D.; Zakrzewski, V. G.; Gao, J.; Rega, N.; Zheng, G.; Liang, W.; Hada, M.; Ehara, M.; Toyota, K.; Fukuda, R.; Hasegawa, J.; Ishida, M.; Nakajima, T.; Honda, Y.; Kitao, O.; Nakai, H.; Vreven, T.; Throssell, K.; Montgomery, Jr., J. A.; Peralta, J. E.; Ogliaro, F.; Bearpark, M. J.; Heyd, J. J.; Brothers, E. N.; Kudin, K. N.; Staroverov, V. N.; Keith, T. A.; Kobayashi, R.; Normand, J.; Raghavachari, K.; Rendell, A. P.; Burant, J. C.; Iyengar, S. S.; Tomasi, J.; Cossi, M.; Millam, J. M.; Klene, M.; Adamo, C.;

Cammi, R.; Ochterski, J. W.; Martin, R. L.; Morokuma, K.; Farkas, O.; Foresman, J. B.; Fox, D. J. Gaussian 16, Revision C.01, Gaussian, Inc., Wallingford CT; **2016**.

(13) Becke, A. D. Density-Functional Thermochemistry. III. The Role of Exact Exchange. *The Journal of Chemical Physics* **1993**, *98* (7), 5648–5652. https://doi.org/10.1063/1.464913.

(14) Krishnan, R.; Binkley, J. S.; Seeger, R.; Pople, J. A. Self-Consistent Molecular Orbital Methods. XX. A Basis Set for Correlated Wave Functions. *J. Chem. Phys.* **1980**, *72* (1), 650–654. https://doi.org/10.1063/1.438955.

(15) Hanwell, M. D.; Curtis, D. E.; Lonie, D. C.; Vandermeersch, T.; Zurek, E.; Hutchison, G. R. Avogadro: An Advanced Semantic Chemical Editor, Visualization, and Analysis Platform. *J. Cheminform.* **2012**, *4* (1), 17. https://doi.org/10.1186/1758-2946-4-17.

(16) Dennington, R.; Keith, T. A.; Millam, J. M. GaussView, Version 6.1, Semichem Inc., Shawnee Mission, KS; **2016**.

(17) Cancès, E.; Mennucci, B.; Tomasi, J. A New Integral Equation Formalism for the Polarizable Continuum Model: Theoretical Background and Applications to Isotropic and Anisotropic Dielectrics. *J. Chem. Phys.* **1997**, *107* (8), 3032–3041. https://doi.org/10.1063/1.474659.

(18) Chai, J.-D.; Head-Gordon, M. Long-Range Corrected Hybrid Density Functionals with Damped Atom–Atom Dispersion Corrections. *Phys. Chem. Chem. Phys.* **2008**, *10* (44), 6615. https://doi.org/10.1039/b810189b.

(19) Dill, J. D.; Pople, J. A. Self-Consistent Molecular Orbital Methods. XV. Extended Gaussian-Type Basis Sets for Lithium, Beryllium, and Boron. *J. Chem. Phys.* **1975**, *62* (7), 2921–2923. https://doi.org/10.1063/1.430801.

(20) Hay, P. J.; Wadt, W. R. *Ab Initio* Effective Core Potentials for Molecular Calculations. Potentials for the Transition Metal Atoms Sc to Hg. *J. Chem. Phys.* **1985**, *82* (1), 270–283. https://doi.org/10.1063/1.448799.
